# A comparison of one‐stage vs two‐stage individual patient data meta‐analysis methods: A simulation study

**DOI:** 10.1002/jrsm.1303

**Published:** 2018-06-21

**Authors:** Evangelos Kontopantelis

**Affiliations:** ^1^ Faculty of Biology, Medicine and Health University of Manchester Manchester UK; ^2^ NIHR School for Primary Care Research University of Manchester Manchester UK

**Keywords:** individual patient data, IPD, meta‐analysis, one‐stage, one‐step, two‐stage, two‐step

## Abstract

**Background:**

Individual patient data (IPD) meta‐analysis allows for the exploration of heterogeneity and can identify subgroups that most benefit from an intervention (or exposure), much more successfully than meta‐analysis of aggregate data. One‐stage or two‐stage IPD meta‐analysis is possible, with the former using mixed‐effects regression models and the latter obtaining study estimates through simpler regression models before aggregating using standard meta‐analysis methodology. However, a comprehensive comparison of the two methods, in practice, is lacking.

**Methods:**

We generated 1000 datasets for each of many simulation scenarios covering different IPD sizes and different between‐study variance (heterogeneity) assumptions at various levels (intercept and exposure). Numerous simulation settings of different assumptions were also used, while we evaluated performance both on main effects and interaction effects. Performance was assessed on mean bias, mean error, coverage, and power.

**Results:**

Fully specified one‐stage models (random study intercept or fixed study‐specific intercept; random exposure effect; and fixed study‐specific effects for covariate) were the best performers overall, especially when investigating interactions. For main effects, performance was almost identical across models unless intercept heterogeneity was present, in which case the fully specified one‐stage and the two‐stage models performed better. For interaction effects, differences across models were greater with the two‐stage model consistently outperformed by the two fully specified one‐stage models.

**Conclusions:**

A fully specified one‐stage model should be preferred (accounting for potential exposure, intercept, and, possibly, interaction heterogeneity), especially when investigating interactions. If non‐convergence is encountered with a random study intercept, the fixed study‐specific intercept one‐stage model should be used instead.

**What is already known?**

- One‐stage and two‐stage approaches to IPD are known, and software to perform them is routinely available.
- Some comparative advantages are known, but very little is known about how they compare in terms of performance on key metrics.

**What is new?**

- A comprehensive and consistent comparison shows that a fully specified one‐stage model should be preferred, especially when investigating interaction terms
- The fully specified one‐stage model should include random study intercept or fixed study‐specific intercept; random exposure effect; and fixed study‐specific effects for a covariate.
- The random study intercept model may not converge, and in that case, the fixed study‐specific intercept model should be used instead.

**Potential impact for RSM readers outside the author's field:**

- Individual patient data meta‐analysis is on the rise, and these findings should provide guidance when conducting such analyses.

## 1 INTRODUCTION

Exploring heterogeneity between studies and identifying the subgroup or subgroups that benefit the most from a successful intervention or exposure should be a key aim of every systematic review and meta‐analysis. Traditionally, this has been achieved through meta‐regressions, which use published results and are easy to perform, but also tend to be underpowered and prone to ecological bias and confounding.^1^, ^2^ Individual patient data (IPD) meta‐analysis provides a potential solution to these problems, along with great modelling flexibility and numerous other advantages, like dealing with missing data at the patient level.^3^ However, an important disadvantage of IPD meta‐analysis is the need for access to the original patient‐level data. Thus, there are logistical challenges that need to be overcome; analyses methods are more complex, while only a subset of the identified datasets are usually obtained since it is common for the authors of the original studies to refuse to share data. Necessarily, IPD analyses can only focus on a self‐selected subsample of the identified relevant studies, which, however, tend to be more recent and of higher quality.

Meta‐analysts wishing to perform an IPD analysis face a choice between one‐stage and two‐stage approaches. The one‐stage approach uses mixed‐effects multilevel regressions to model within‐ and between‐study variances (ie, heterogeneity) and quantify the effect or association of interest in a single analytical model. The association of interest may be a main or an interaction effect, while the regression model can be linear, logistic, Poisson, or other, depending on the outcome type. An alternative IPD approach involves modelling in two stages. In the first step, study‐level estimates (main or interaction effects/associations) are obtained using simple regression models. In the second step, the common methods of meta‐analysis summary results are utilised to obtain an overall estimate across all studies. Usually, a random‐effects DerSimonian‐Laird model is used in the second step,^4^ which has been shown to be more conservative and better performing in the presence of heterogeneity,^5^ even when the normality assumption for the effect(s) is violated.^6^

Various modelling choices are possible within the 1‐stage IPD approach, under different assumptions for the intercept (random, fixed common, or fixed study specific) and similarly for the exposure effect and any covariates, including any baseline information. It is known that the correct specification of the 1‐stage model is critical,^7^, ^8^ and guides and software to aid researchers in this are available.^9^, ^10^, ^11^ Fewer modelling options are available for two‐stage analysis, and they are mainly around the choice of the second step model, fixed effect, or one of the numerous random‐effects options.^12^, ^13^ A well‐developed implementation of a two‐stage analysis is available in Stata,^14^ with the less flexible *ipdmeta* command, which can only analyse a continuous outcome, available in R software.^15^ One‐stage analyses rely on widely used mixed‐effects models and are considered more flexible,^16^ but they are more challenging in both conducting and communicating the findings, especially regarding visualisation with the hallmark forest plot, although software solutions are available.^10^ These challenges with the one‐stage approach drive meta‐analysts to the two‐stage approach, which is more prevalent,^17^ although other challenges like power calculations,^18^ or multiple imputation,^19^, ^20^, ^21^ are common across both approaches.

An algebraic comparison of one‐stage and two‐stage IPD demonstrated that in the presence of covariates, which is typically the case, the two approaches completely agree only in scenarios of unrealistically simplifying assumptions.^22^ Often, results are found to be similar in real datasets, but 1 stage is recommended as a more exact statistical approach that accounts for parameter correlation, although estimation issues affect both approaches (unstable two‐stage estimates and convergence issues for fully specified 1‐stage models).^23^ However, it has recently been suggested that one‐stage analyses of interactions can lead to *deluded* evaluations in certain scenarios, namely, when the covariate of interest is grossly imbalanced across trials (for example, when assessing the interaction between gender and the intervention of interest, but some studies have only enrolled men).^24^ But it has also been argued that most differences between the two approaches arise because of different modelling assumptions, rather than the choice of one‐stage or two‐stage itself.^11^

Nevertheless, practical recommendations on the modelling choice between one‐stage and two‐stage meta‐analyses are lacking, based on performance metrics in real‐world settings. Asymptotically, the methods may well be equivalent or very close,^11^ although it has been shown that in the common scenario when covariates are present, the two approaches coincide only under extreme simplifying assumptions, which are somewhat unrealistic in practice.^22^ Nevertheless, little is known about practical performance of these methods in widely used models, especially when numbers are small, or when the focus is on interaction terms, which are typically the primary outcome in IPD. The aim of the paper is to comprehensively evaluate one‐stage vs two‐stage IPD across numerous scenarios and provide practical recommendations for these widely used methods.

## 2 METHODS

### 2.1 One‐stage

As previously mentioned, mixed‐effects models for all types of outcomes are relevant in the IPD context. We will focus on linear models and a continuous outcome for simplicity (reasonable computation time and fewer convergence issues), both in the notation and the simulation set‐up to be described in a later section. Detailed descriptions of such models for various outcomes have been described elsewhere.^9^, ^25^, ^26^ The simplest specification assumes a common intercept across studies, fixed effect for the covariate (assuming one is present and for which we wish to adjust the models), and a random effect for the exposure, where we use gammas and betas to describe fixed and random effects, respectively:

(1) $$Y_{\mathrm{ij}}=\gamma_{0}+\beta_{1j}\cdot {\text{group}}_{\mathrm{ij}}+\gamma_{2}\cdot x_{\mathrm{ij}}+\varepsilon_{\mathrm{ij}},$$

(2) $$\beta_{1j}=\gamma_{1}+u_{1j}.$$

Where error *ε* and centred random‐effects component *u* are usually assumed to be normally distributed:

(3) $$\varepsilon_{\mathrm{ij}}\sim N\left(0,\sigma_{j}^{2}\right),$$

(4) $$u_{1j}\sim N\left(0,\tau_{1}^{2}\right),$$

where *i* is the patient, *j* the study, *Y* the continuous outcome, *γ*_0_ the fixed common intercept, *β*_1*j*_ the random exposure effect for study *j*, *γ*_1_ the mean exposure effect, *group* the binary exposure variable (ie, 0 = control and 1 = treatment), *γ*_2_ the fixed covariate effect, *x* the covariate, $\tau_{1}^{2}$ the between‐study variance for the exposure, and $\sigma_{j}^{2}$ the within‐study variance for study *j*.

An alternative specification is generally recommended, since the common intercept and fixed covariate assumptions may not be easy to justify, which involves study‐varying fixed intercept and fixed covariate effects^9^:

(5) $$Y_{\mathrm{ij}}=\gamma_{0j}+\beta_{1j}\cdot {\text{group}}_{\mathrm{ij}}+\gamma_{2j}\cdot x_{\mathrm{ij}}+\varepsilon_{\mathrm{ij}},$$

where *β*_1*j*_ is as in (2), *γ*_0*j*_ the fixed intercept for study *j*, and *γ*_2*j*_ the fixed covariate effect for study *j*. A more complex specification involves random effects for the study intercepts:

(6) $$Y_{\mathrm{ij}}=\beta_{0j}+\beta_{1j}\cdot {\text{group}}_{\mathrm{ij}}+\gamma_{2j}\cdot x_{\mathrm{ij}}+\varepsilon_{\mathrm{ij}},$$

(7) $$\beta_{0j}=\gamma_{0}+u_{0j},$$

where we assume a non‐zero correlation *ρ* between the exposure and intercept random effects:

(8) $$u_{0j}\sim N\left(0,\tau_{0}^{2}\right),$$

(9) $$\mathrm{cov}\left(u_{0j},u_{1j}\right)=\rho \cdot \tau_{0}\cdot \tau_{1},$$

where $\tau_{0}^{2}$ is the between‐study variance for the intercept. Additional random effects can be added, in this context for the covariate, although convergence issues may limit the practical usefulness of such models.^10^ Finally, in some cases, the focus may be on interactions, and in such a scenario, we would expand (6) to

(10) $$Y_{\mathrm{ij}}=\beta_{0j}+\beta_{1j}\cdot {\text{group}}_{\mathrm{ij}}+\gamma_{2j}\cdot x_{\mathrm{ij}}+\gamma_{3}\cdot {\text{group}}_{\mathrm{ij}}\cdot x_{\mathrm{ij}}+\varepsilon_{\mathrm{ij}}.$$

Estimation of all these multilevel models is achieved using maximum likelihood or restricted maximum likelihood algorithms.

### 2.2 Two‐stage

In the two‐stage approach, study‐varying fixed effects are necessarily used for all parameters of interest, intercept, exposure, and covariate:

(11) $$Y_{\mathrm{ij}}=\gamma_{0j}+\gamma_{1j}\cdot {\text{group}}_{\mathrm{ij}}+\gamma_{2j}\cdot x_{\mathrm{ij}}+\varepsilon_{\mathrm{ij}}.$$

For each study *j*, *γ*_1*j*_ is estimated using a simple regression model. The aim of two‐stage meta‐analysis of aggregate results is to pool the estimates of the study exposure effects *γ*_1*j*_ and estimate the overall true mean effect *θ*. Under a random‐effects model that allows *γ*_1*j*_ to vary across studies to within‐ and between‐study variances, we have

(12) $$\gamma_{1j}=\theta_{j}+e_{j},$$

(13) $$e_{j}\sim N\left(0,\sigma_{j}^{2}\right).$$

And

(14) $$\theta_{j}=\theta +\varepsilon_{j},$$

(15) $$\varepsilon_{j}\sim N\left(0,\tau^{2}\right),$$

where *τ*^2^ is the between‐study variance. Numerous estimators exist for *τ*^2^,^12^, ^13^ with the one most commonly used proposed by DerSimonian and Laird^4^:

(16) $${\hat{\tau }}_{\mathrm{DL}}^{2}=\frac{Q_{\hat{w}}-\left(k-1\right)}{\sum_{j=1}^{k}{\hat{w}}_{j}-\sum_{j=1}^{k}{\hat{w}}_{j}^{2}/\sum_{j=1}^{k}{\hat{w}}_{j}},$$

(17) $$Q_{\hat{w}}=\sum_{j=1}^{k}{\hat{w}}_{j}{\left({\hat{\gamma }}_{1j}-{\hat{\gamma }}_{1}\right)}^{2},$$

where ${\hat{w}}_{j}=1/\sigma_{j}^{2}$, *k* is the number of studies, and ${\hat{\gamma }}_{1}=\frac{\sum_{j=1}^{k}{\hat{w}}_{j}{\overset{´}{Y}}_{j}}{\sum_{j=1}^{k}{\hat{w}}_{j}}$ is the overall exposure effect estimate under a fixed‐effect approach with ${\overset{´}{Y}}_{j}=\theta +e_{j}$.

### 2.3 Data generation

Various data scenarios were generated using the *ipdpower* command in Stata, which allows modelling between‐study variability and thus can generate IPD with different levels of statistical heterogeneity for the parameters of interest.^18^ Some aspects of the data generation design were the same across all simulation scenarios, in which we specified numerous permutations for the number of studies, the (mean) number of patients within a study, the between‐study variance (heterogeneity) for the intercept, and the between‐study variance (heterogeneity) for the exposure. The simulated sizes for patients and studies are displayed in Table 1, and each of these was repeated for different combinations of intercept and exposure heterogeneity (as measured by *I* ^2^, a ratio of within‐ and between‐study variability, with up to 25% considered modest, 25% to 50% moderate, and over 50% substantial heterogeneity): 0% and 0%; 50% and 0%; 0% and 50%; 33% and 33%; 50% and 50%; and 67% and 67%.

**Table 1:** Lower and higher level simulation sizes

Total Number of Patients	Number of Studies	Mean Number of Patients in Each Study^a^
5000	10	500
2000	4	500
1000	2	500
5000	20	250
2000	8	250
1000	4	250
5000	50	100
2000	20	100
1000	10	100

a Drawn from a uniform distribution within the *ipdpower* command (more information on the process is available in the *ipdpower* help file).

#### 2.3.1 Simulation setting 1

The data generation model followed what was described in Section 2.1 and Equation (10) with a continuous outcome *Y*, a binary exposure *group*, a continuous covariate *x*, and an interaction term between the exposure and the covariate. The hypothesised parameters were *γ*_0_ = 1 (intercept), *γ*_1_ = 0.5 (exposure), *γ*_2_ = 0.3 (covariate), and *γ*_3_ = 0 (interaction). The design was balanced with a 0.5 exposure probability, while the distribution of the continuous covariate did not vary across studies. The standard deviation *σ* for the residual error *ε* was set to 1 across all studies (the within‐study variance), against which the between‐study variances *τ*_0_ and *τ*_1_ were set to produce data at various heterogeneity levels using *I*^2^ = *τ*^2^/(*τ*^2^ + *σ*^2^).

#### 2.3.2 Simulation setting 2

Here, we introduced a small‐study effect scenario, by dropping a set percentage of small studies with low estimates for the exposure (*γ*_1_ ≈ 0 or *γ*_1_ < 0). This was meant to serve as a proxy for publication bias, arguably the biggest threat to conducting meta‐analysis, and it is known that the weighting of studies in two‐stage meta‐analysis under a fixed‐effect or random‐effects model is important in this context. Random‐effects meta‐analyses use inverse‐variance weighting, under which the weights assigned to smaller studies, the ones that are the most affected by publication bias, are larger compared with weights assigned under the fixed‐effect model. Thus, evaluating whether two‐stage performance deteriorates in the presence of publication bias, compared with the one‐stage approach, seems relevant—especially since detecting and accounting for publication bias can be challenging. Within each simulation generated by ipdpower, smaller studies have greater variability in the key parameter of interest *β*_1*j*_, so the simulation set‐up is suitable for this investigation. Studies were ranked on size and effect size, with 20% of the studies ranked the lowest on both parameters (small size and effect) being dropped.

#### 2.3.3 Simulation setting 3

The standard deviation *σ* of the residual error *ε* was set to vary across studies. The mean of *σ* was set to 1 with a standard deviation was 0.5, allowing perhaps a more realistic variation of within‐study variance across studies.

#### 2.3.4 Simulation setting 4

Here, we introduced moderate levels of skewness (skew = 1; kurtosis = 4) in the distribution of all random‐effects components, which previously followed normal distributions (Equations (4) and (8)).

#### 2.3.5 Simulation setting 5

Including an interaction term between the binary exposure and continuous covariate, which would be the focus of the investigation, the hypothesised parameters in this setting were *γ*_0_ = 1 (intercept), *γ*_1_ = 1 (exposure), *γ*_2_ = 0.5 (covariate), and *γ*_3_ = 0.4 (interaction). This setting allows us to evaluate performance on quantifying effect heterogeneity (interaction) rather than the main effect. It should be noted that Equation (10) is known to be problematic in decomposing within‐ and between‐study interactions, and centring the continuous covariate at the study level is essential.^7^ However, in the simulation, deviations from zero for the means of the generated covariate at the study level are very small and down to statistical error, since they are generated from *N*(0, 1).

#### 2.3.6 Simulation setting 6

Including a binary rather than a continuous covariate, with a balanced 0.5 probability, this allows for the investigation of a binary by binary interaction. The hypothesised parameters are as in simulation setting 4.

#### 2.3.7 Simulation setting 7

Here, we used the same set‐up as for simulation 6, but also allowed the levels of the binary covariate to vary greatly across studies from 0% to 100%, this does happen in practice (for example, studies enrolling only men or only women), although it was more common in the past. The focus in again on the interaction tem.

#### 2.3.8 Simulation setting 8

We used the same settings as in simulation setting 6, but included heterogeneity for both the covariate (*I* ^2^ = 50%) and the interaction term (*I* ^2^ = 50%), effectively, this scenario includes between‐study heterogeneity for each component (intercept, exposure, covariate, and interaction term), to evaluate whether modelling approaches can practically separate between the various heterogeneity levels in estimating the interaction effect.

### 2.4 Analysis

The simulated datasets were analysed in Stata using the *mixed* command, for one‐stage mixed‐effects modelling, and the *ipdmetan* command, for two‐stage modelling. Three mixed‐effects models of an increasing order of complexity were used, which have been practically described in this context elsewhere, and call on the built‐in *mixed* command for multilevel mixed‐effects linear regression that was fitted through the default maximum likelihood^10^: (*a*) fixed common intercept, random exposure effect, and fixed effect for the covariate (Equation (1)); (*b*) fixed study‐specific intercepts, random exposure effect, and fixed study‐specific effects for the covariate (Equation (5)); and (*c*) random study intercept, random exposure effect, and fixed study‐specific effects for the covariate (Equation (6)). A fourth regression model (*d*) was called through *ipdmetan*, pooling study results using a restricted maximum likelihood random‐effects meta‐analysis model. Interaction terms were only included in the models analysing data from simulation settings 5 to 7. For simulation setting 8 alone, we included more complex models that included random effects for the interaction: (*e*) fixed study‐specific intercepts, random exposure effect, fixed study‐specific effects for the covariate, and random interaction effect; (*f*) random study intercept, random exposure effect, fixed study‐specific effects for the covariate, and random interaction effect; and (*g*) random study intercept, random exposure effect, random covariate effect, and random interaction effect.

### 2.5 Performance measures

We evaluated the performance of the 4 models in all scenarios and simulation settings previously described, over 1000 iterations. The estimates of interest were the exposure effect *γ*_1_ (main effect) in simulation settings 1 to 4 and the interaction effect *γ*_3_ in simulation settings 5 to 7. To allow for a comprehensive comparison, performance was assessed on a range of metrics: mean error, mean bias, coverage probability, and power. Mean error is an aggregate of the absolute difference in the estimate to the true parameter expressed as $\frac{1}{1000}\sum_{i=1}^{1000}|z-{\hat{z}}_{i}|$ where *z* is the true association of interest. Mean bias is an aggregate of the difference in the estimate to the true parameter, or $\frac{1}{1000}\sum_{i=1}^{1000}\left(z-{\hat{z}}_{i}\right)$. The coverage probability is the proportion of 95% confidence intervals for the estimate that contain the true parameter across the 1000 iterations, which theoretically should be close to 95%. To balance the fact that methods that return very wide confidence intervals will score highly on coverage, we also calculated power (although potentially problematic in the presence of bias). Over the 1000 iterations, we calculated power as a function to detect the probability for each effect estimate to be statistically different to zero (models 1‐4) or 0.2 (models 5‐7). We varied the hypothesis to allow for meaningful levels of obtained power that help discriminate methods.

## 3 RESULTS

**Figure 1:**
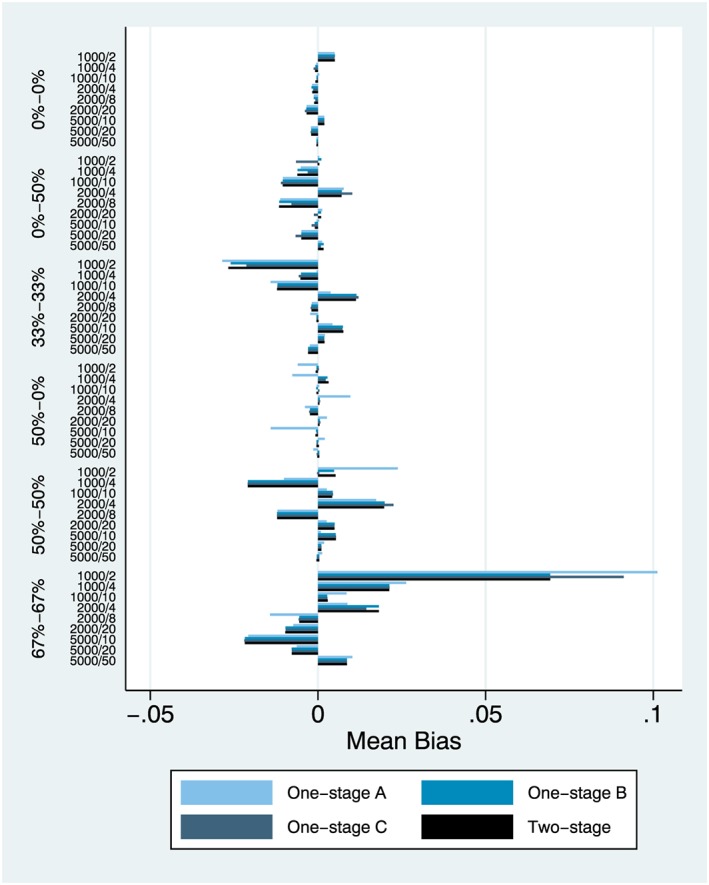
Mean bias, simulation setting 1 (main effect) [Colour figure can be viewed at http://wileyonlinelibrary.com]

**Figure 2:**
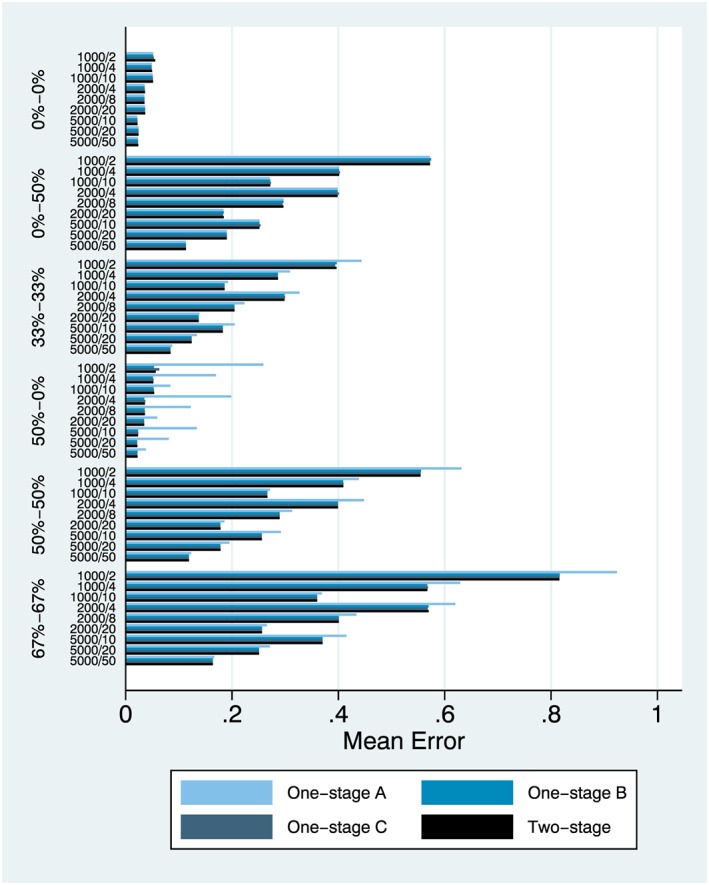
Mean error, simulation setting 1 (main effect) [Colour figure can be viewed at http://wileyonlinelibrary.com]

**Figure 3:**
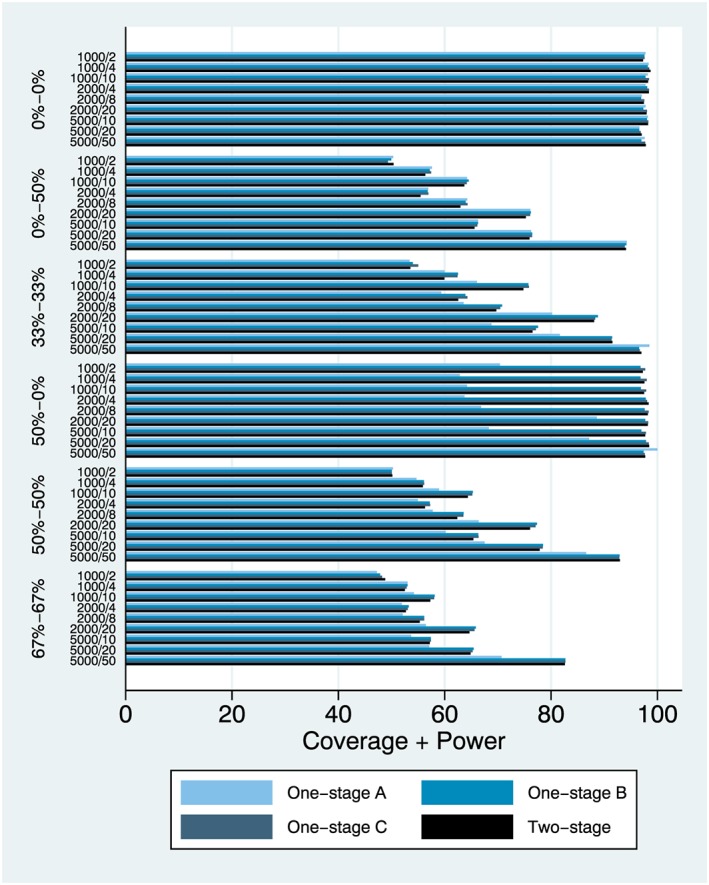
Coverage and power (%), plotted together [(coverage + power)/2], simulation setting 1 (main effect) [Colour figure can be viewed at http://wileyonlinelibrary.com]

**Figure 4:**
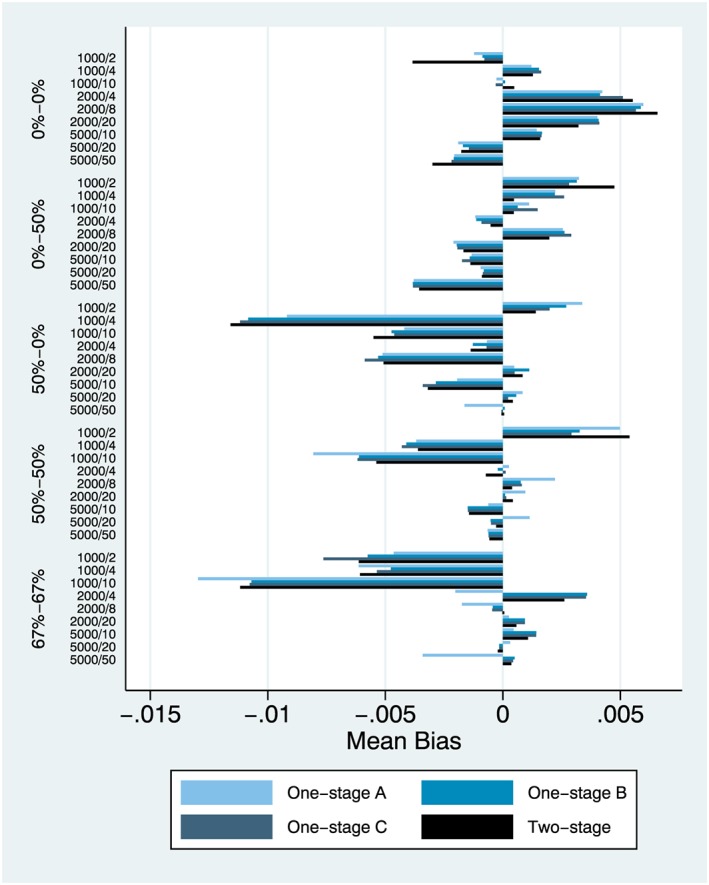
Mean bias, simulation setting 6 (interaction effect) [Colour figure can be viewed at http://wileyonlinelibrary.com]

**Figure 5:**
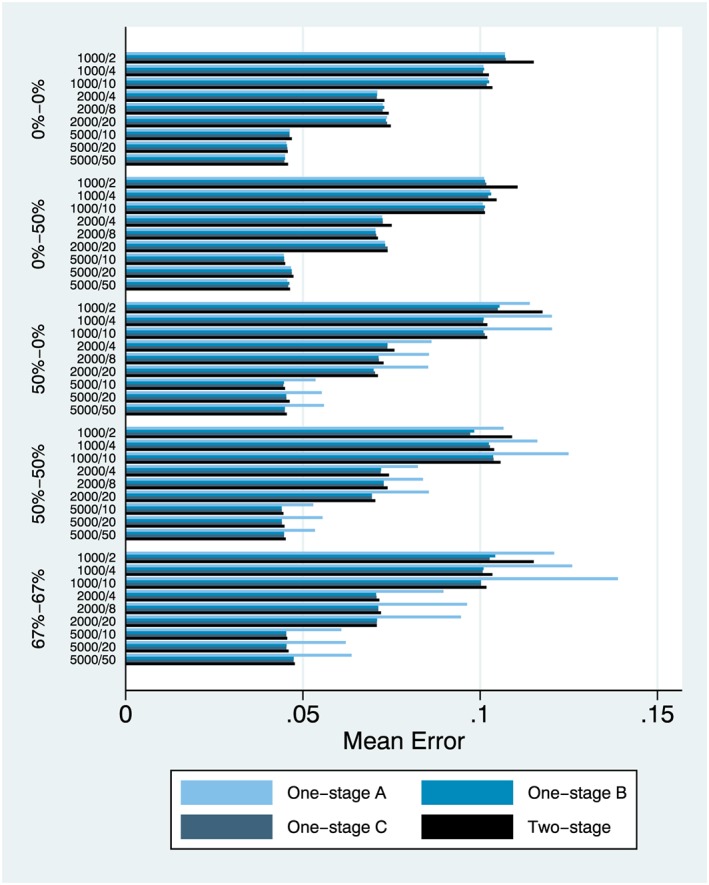
Mean error, simulation setting 6 (interaction effect) [Colour figure can be viewed at http://wileyonlinelibrary.com]

**Figure 6:**
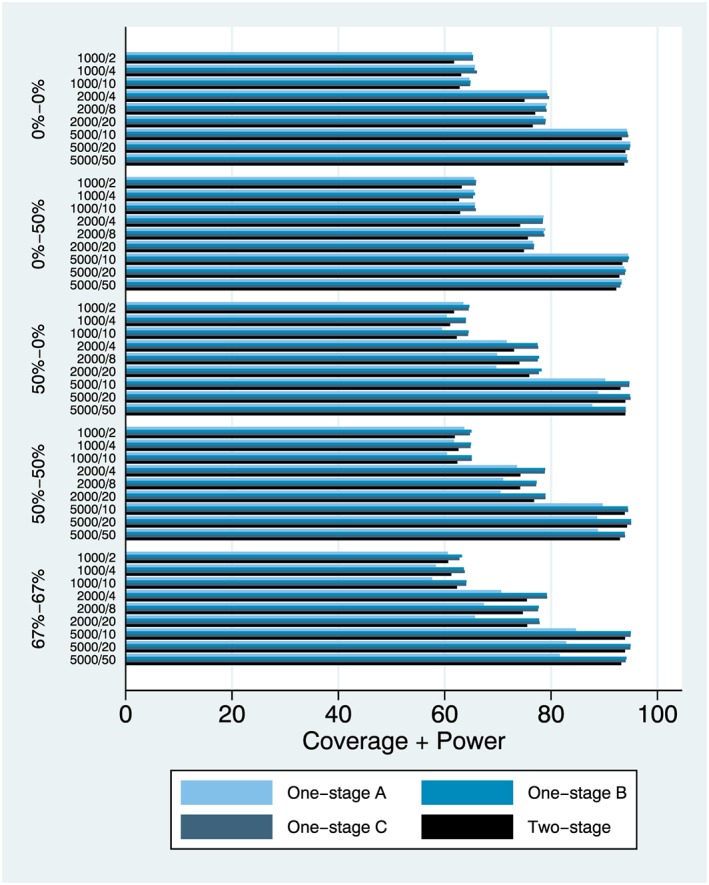
Coverage and power (%), plotted together [(coverage + power)/2], simulation setting 6 (interaction effect) [Colour figure can be viewed at http://wileyonlinelibrary.com]

We focus on results from simulation setting 1 and the main effect (Figures 1, 2, 3) and also simulation setting 6 and the interaction effect (Figures 4, 5, 6). Complete results from all 8 simulation settings are presented in Appendix S1, including information on model convergence. Information on interpreting the figures is provided in Table 2.

**Table 2:** Interpreting the *Y* axis labels on all figures

Level	What Is It	Additional Information
One	The total number of patients and studies	1000/2, implies a total of 1000 patients over 2 studies
Two	*I* ^*2*^ levels for the intercept and exposure	0%‐50%, implies no heterogeneity for the intercept and *I* ^*2*^ = 50% for the exposure
Legend	The 4 models used (three 1‐stage, one 2‐stage)	One‐stage A: fixed common intercept, random exposure effect, and fixed effect for the covariate One‐stage B: fixed study‐specific intercepts, random exposure effect, and fixed study‐specific effects for the covariate One‐stage C: random study intercept, random exposure effect, and fixed study‐specific effects for the covariate. Two‐stage: called through *ipdmetan*, pooling study results using a restricted maximum likelihood random‐effects meta‐analysis model

### 3.1 Mean bias

Patterns of mean bias were very low and comparable across all 4 models, with the exception of high heterogeneity at both levels and very small IPD, of 1000 patients over two studies (Figure 1). In that particular scenario, the two‐stage model (d) and 1‐step model (b) were the best performers. In general, there was very little to separate the models, with only one‐stage model (a) lagging somewhat in performance.

### 3.2 Mean error

Mean error was almost identical across all 4 models for no heterogeneity and exposure only heterogeneity (Figure 2). However, as intercept heterogeneity increases, the performance of model (a) deteriorated, in relation to that of models (b), (c), and (d), especially for smaller IPD. Performance of one‐stage models (b) and (c) and the two‐stage model (d) was almost indistinguishable.

### 3.3 Coverage and power

Coverage and power are interlinked, and it makes sense to assess simultaneously. Model performance is almost identical when heterogeneity is absent, both in terms of coverage and power (Figures S3 and S4). However, model performance diverges when heterogeneity is introduced with model (a) generally having higher coverage and lower power than models (b) to (d), often above the nominal 95%, especially for large samples and when heterogeneity for the intercept is introduced. To more reliably assess performance across both metrics, we plotted coverage and power together (Figure 3) where we observe that models (b) to (d) outperform model (a) in the presence of intercept heterogeneity (which is not surprising since the latter does not account for study‐varying intercepts). Comparing the 1‐stage models (b) and (c) with the two‐stage model (d), there are small differences but one‐stage model (c) seems to be performing slightly better, overall. In most cases, however, model (b) and model (c) performance was identical.

### 3.4 Convergence

The two‐stage model (c) was the only model that converged in all cases (Figure S6). One‐stage models (a) and (b) always converged except when heterogeneity was not modelled, and then only a handful of cases did not converge. As expected, the fully specified model (c) was the most susceptible to non‐convergence but only in one case did the convergence rate fall below 80% (very small IPD and high heterogeneity on both levels). In general, the larger the IPD, the higher the convergence rate for model (c), while interestingly non‐convergence issues appeared to be more resistant to sample size increases when only one level of heterogeneity was modelled (intercept or exposure but not both or neither).

### 3.5 Sensitivity analyses for the main effect

All results are presented in detail in Appendix S1. Model convergence was almost identical unless otherwise stated.

#### 3.5.1 Simulation setting 2: publication bias

Mean bias was much higher than in simulation setting 1, as expected, while levels of mean error were only slightly higher. Coverage and power levels were similar to what was seen in the main results. Relative model performance was very similar across models (b) to (d), with the fully specified one‐stage model (c) performing very similarly to one‐stage model (b), and only slightly outperforming the two‐stage model (d) in terms of cumulative coverage and power (Figures S7‐S12).

#### 3.5.2 Simulation setting 3: varying within‐study variance levels across studies

There were practically no differences to the main results in all 4 metrics, and relative model performance was the same (Figures S13‐S18).

#### 3.5.3 Simulation setting 4: skew‐normal distribution for the random effects

Results were almost identical to the main results (Figures S19‐S24).

### 3.6 Interaction effect

In simulation settings 5 to 8, we focused on the interaction term, rather than the main effect, and we investigated a binary by continuous (setting 5) and a binary by binary interaction term (settings 6‐8). Results were broadly similar to what we observed for the main effect, with some differences that are discussed below, for each simulation setting.

#### 3.6.1 Simulation setting 5: binary (exposure) by continuous (covariate) interaction

Mean bias levels were almost zero, as in the analyses for the main effect, but consistently so even for very small IPD of high heterogeneity. Mean error was also very low but higher in the two‐stage model (d) for small IPD of exposure only heterogeneity and, especially, when no heterogeneity was modelled. When intercept heterogeneity was modelled, models (a) and (b) were the worst performers on mean error, as expected. Coverage was almost at nominal levels for all models in all simulation scenarios, but there was great variation in power with one‐stage models (b) and (c) being the best performers overall and two‐stage model (d) the worst performer in most no intercept heterogeneity scenarios and consistently outperformed by (b) and (c) (Figures S25‐S30).

#### 3.6.2 Simulation setting 6: binary (exposure) by binary (covariate) interaction

Mean bias results were similar to what was observed in simulation setting 5, but mean error results were higher (Figures 4 and 5). Nevertheless, relative performance was the same across both metrics with the only difference being the slightly better performance of one‐stage models (b) and (c) compared to two‐stage model (d) in all settings, especially in small IPD. Coverage and power results were very close to what was observed in simulation setting 5, with one‐stage models (b) and (c) being the best performers overall (Figure 6). Complete results for this scenario are also presented in Figures S31 to S36.

#### 3.6.3 Simulation setting 7: binary (exposure) by binary (covariate) interaction with varying covariate levels across studies

Model differences in mean bias were generally very small, although they spiked for very small meta‐analyses of high heterogeneity at both levels for model (a). One‐stage models (b) and (c) were the best performers in terms of bias (which was considerable in this scenario) and better than two‐stage model (d) in all scenarios except very small IPD of high or very high heterogeneity at both levels. In terms of cumulative power and coverage, one‐stage models (b) and (c) were again the best performers in the vast majority of scenarios, greatly outperforming two‐stage model (d) in no heterogeneity, exposure only heterogeneity, and intercept only heterogeneity scenarios. Convergence was also affected in this setting, especially for model (d) that fell to around 90% for many small IPD scenarios (Figures S37‐S42).

#### 3.6.4 Simulation setting 8: binary (exposure) by binary (covariate) interaction, including heterogeneity for the covariate and the interaction term

In this scenario, one‐stage models (a) to (c) that did not account for the heterogeneity in the interaction term greatly underperformed compared with the two‐stage model on all metrics. However, when a random‐effects term for the interaction was included in one‐stage models (e) to (f), performance became similar across the three one‐stage models, and it was frequently marginally higher than in the two‐stage model—except for the smallest datasets we investigated (1000 cases and up to 4 studies). Convergence did not appear to be an issue with the more complex one‐stage models (Figures S43‐S54).

## 4 DISCUSSION

Our results showed that a fully specified one‐stage model that accounts for study‐varying intercepts (either as fixed or random effects) and a two‐stage approach are very close in terms of performance, irrespective of heterogeneity levels and IPD sizes. However, some small yet consistent differences were observed. In terms of evaluating the main effect, although there was practically no difference in mean bias or mean error, the fully specified one‐stage models performed slightly better overall in the cumulative of coverage and power. This difference in the coverage‐power cumulative (mainly driven by differences in power) was evident and more pronounced when investigating interaction terms, arguably the focus of IPD meta‐analysis. In addition, when evaluating interaction terms, mean error rates were generally lower for the fully specified one‐stage approaches, especially for small IPD and for a binary by binary interaction with varying covariate levels across studies (which is a rather common scenario). In the presence of high heterogeneity for the interaction term, which does not seem like a common scenario, the fully specified one‐stage approaches that included a random effect for the interaction slightly outperformed the two‐stage approach.

### 4.1 Strengths and limitations

We generated realistic data across numerous assumptions and scenarios to extensively compare one‐stage and two‐stage approaches in IPD meta‐analysis. Nevertheless, some limitations exist. First, although realistic and numerous, our simulated scenarios are not exhaustive and results may vary in alternative scenarios. However, the patterns of results are consistent across all simulation settings, and we would expect the methods to perform similarly in other scenarios, at least relatively to each other, and our conclusions not to be affected. Second, the precision obtained with simulations of 1000 iterations is not ideal but the models we executed are complex and require considerable computational time—especially due to the more complex one‐stage models. Although one‐stage models are computationally more expensive, this is irrelevant in the context of a single analysis with a modern computer, and each model converges in seconds (for example, it may take up to 20 s for a one‐stage model when a two‐stage model may converge in under a second). Third, we focused on a continuous outcome when dichotomous or count outcomes are common. This is because computational time for logistic or Poisson regressions is higher (much higher for the latter) and non‐convergence is much more prevalent, an issue that necessarily limits comparisons to larger IPD. It is likely that the relative performance of the methods will be similar, although absolute performance levels could be quite different. Nevertheless, more research is needed to validate this. Finally, on the surface, our analysis may appear to be an unfair comparison of numerous one‐stage models vs one two‐stage model. However, one‐stage models are much more complex, with many more parameters to consider and model. In the two‐stage model, the main issue is the way to model heterogeneity and it has been shown that all random‐effects approaches are broadly similar and share the same limitations.^6^, ^27^

### 4.2 Practical recommendations

When investigating main effects, and in the absence of heterogeneity for the intercept, there is practically nothing to separate the 4 models we investigated. Heterogeneity is almost always an unknown, however, despite the fact that levels of heterogeneity (or unexplained between‐study variance) tend to be lower in IPD compared with traditional meta‐analyses because of more focused research questions and covariates that are included in the models (eg, age and sex). Therefore, we recommend the use of a fully specified one‐stage model (Equations (5) and (6)), and if convergence issues are encountered, the two‐stage model should be used instead.

When investigating interaction effects of any type, we also recommend the use of a fully specified one‐stage model, which outperformed the other models in the most challenging settings of small IPD with intercept heterogeneity. Non‐convergence in one‐stage models is possible, especially for the fully specified model with random intercepts. In the rare situation when neither of the fully specified one‐stage models converges, the alternative choice should depend on intercept heterogeneity expectations. If considerable intercept heterogeneity is expected, then the two‐stage model should be used, but if that is not the case, then a simpler common intercept assuming one‐stage model should be preferred. Finally, in the presence of considerable heterogeneity for the interaction term, which does not seem like a common scenario, fully specified one‐stage models should be preferred, provided that a random effect for the interaction is included.

Of particular interest in investigating interactions was simulation setting 7, with a binary covariate of varying levels across studies and of considerable heterogeneity, when consequently within‐study investigations are not possible in all studies (for example, if the covariate of interest is sex yet studies that enrol a single sex are common). This aimed to evaluate claims that in such scenarios, the focus should be on within‐study interactions alone, and all studies in which such evaluations are not possible should be excluded, pointing towards the use of a two‐stage model.^24^ Yet our findings show that even in that challenging context, a fully specified one‐stage model is still the best choice (and even more so compared to other simulation settings). In addition, as evidenced in simulation setting 8 where numerous heterogeneity components were modelled, a fully specified one‐stage model can provide a reliably estimate of the interaction term and can practically distinguish between various within‐ and between‐study components. Returning to simulation setting 7, completely excluding studies greatly affects power and if we recommend the use of a fully specified 1‐stage model and the inclusion of all studies, unless underlying bias (confounding) is suspected in the association between the distribution/level of the covariate and the effect size. As discussed in Section 1, one‐stage approaches, if not appropriately modelled, can be susceptible to confounding and analysts need to be careful when implementing them in this context.^7^

Besides performance, other practical implications need to be considered when deciding between a one‐stage and a two‐stage IPD model. One‐stage models can directly use multiple imputation techniques since they are standard mixed‐effects models. Although heterogeneity makes the imputation challenging and different approaches have been proposed,^20^, ^21^, ^28^, ^29^, ^30^ missing data at the patient level are common in such investigations and the flexibility of the one‐stage model is invaluable in this context. In addition, evaluating study‐level covariates and their interactions with exposure, although challenging, is only possible through one‐stage models.

Whether using one‐stage or two‐stage models, IPD meta‐analyses will not manage to collect patient data from all identified studies, but a self‐selected sample of usually newer and location‐clustered studies (due to the fact IPD collaborations are partly driven by personal relationships). Therefore, a comparison between the exposure estimate in the IPD sample and in all studies (usually reported in an aggregate data meta‐analysis) is always recommended. However, two‐stage models can use reported study‐level estimates from studies for which patient data are not available, and seamlessly incorporate them in the analyses. This approach can be very helpful when investigating main effects but less so when the focus is on interactions, since interactions are much less likely to be reported. Nevertheless, authors who were unwilling to share their data may be willing to run such an analysis and share the results.

### 4.3 Conclusions

Fully specified one‐stage models that account for possible exposure and intercept heterogeneity (random study intercept or fixed study‐specific intercept; random exposure effect; and fixed study‐specific effects for a covariate—and additionally, random interaction effect in the presence of interaction heterogeneity), and which have been practically described elsewhere,^10^ were the best performers across the simulation scenarios we generated. This was true when estimating main effects or, especially, interaction effects, but on the assumption there exists no underlying confounding in the association between the confounder and the effect size. Although non‐convergence is not uncommon for the random study intercept one‐stage model, it is unlikely for the fixed study‐specific intercept (at least for a continuous outcome), and analysts should prefer one of these two models.

## FUNDING

MRC Health eResearch Centre grant MR/K006665/1 supported the time and facilities of EK.

## CONFLICT OF INTEREST

E.K. declares no conflict of interests.

## Supporting information

Data S1. Figure A1: Mean Bias
Figure A2: Mean Error
Figure A3: Coverage probability (%), against 95% nominal line
Figure A4: Power probability (%)
Figure A5: Coverage and Power (%), plotted together [(coverage+power)/2]
Figure A6: Model convergence (%)
Figure A7: Mean Bias
Figure A8: Mean Error
Figure A9: Coverage probability (%), against 95% nominal line
Figure A10: Power probability (%)
Figure A11: Coverage and Power (%), plotted together [(coverage+power)/2]
Figure A12: Model convergence (%)
Figure A13: Mean Bias
Figure A14: Mean Error
Figure A15: Coverage probability (%), against 95% nominal line
Figure A16: Power probability (%)
Figure A17: Coverage and Power (%), plotted together [(coverage+power)/2]
Figure A18: Model convergence (%)
Figure A19: Mean Bias
Figure A20: Mean Error
Figure A21: Coverage probability (%), against 95% nominal line
Figure A22: Power probability (%)
Figure A23: Coverage and Power (%), plotted together [(coverage+power)/2]
Figure A24: Model convergence (%)
Figure A25: Mean Bias
Figure A26: Mean Error
Figure A27: Coverage probability (%), against 95% nominal line
Figure A28: Power probability (%)
Figure A29: Coverage and Power (%), plotted together [(coverage+power)/2]
Figure A30: Model convergence (%)
Figure A31: Mean Bias
Figure A32: Mean Error
Figure A33: Coverage probability (%), against 95% nominal line
Figure A34: Power probability (%)
Figure A35: Coverage and Power (%), plotted together [(coverage+power)/2]
Figure A36: Model convergence (%)
Figure A37: Mean Bias
Figure A38: Mean Error
Figure A39: Coverage probability (%), against 95% nominal lin
Figure A40: Power probability (%)
Figure A41: Coverage and Power (%), plotted together [(coverage+power)/2]
Figure A42: Model convergence (%)
Figure A43: Mean Bias, models A‐D
Figure A44: Mean Bias, models D & E‐G
Figure A45: Mean Error, models A‐D
Figure A46: Mean Error, models D & E‐G
Figure A47: Coverage probability (%), against 95% nominal line, models A‐D
Figure A48: Coverage probability (%), against 95% nominal line, models D & E‐G
Figure A49: Power probability (%), models A‐D
Figure A50: Power probability (%), models D & E‐G
Figure A51: Coverage and Power (%), plotted together [(coverage+power)/2], models A‐D
Figure A52: Coverage and Power (%), plotted together [(coverage+power)/2], models D & E‐G
Figure A53: Model convergence (%), models A‐D
Figure A54: Model convergence (%), models D & E‐G

Data S2. Supporting Information
