# Supplementary material for: A comparison of one‐stage vs two‐stage individual patient data meta‐analysis methods: A simulation study
Source: Res Synth Methods. 2018 Jun 21;9(3):417–30. doi: 10.1002/jrsm.1303 (PMC6175226; doi:10.1002/jrsm.1303)

Supplementary file for "A comparison of one-stage vs two-stage individual patient data meta-analysis methods: a simulation study"

This supplementary file includes results for all additional simulation settings, which were not fully presented in the main paper and were only discussed in comparison to the main results (simulation setting 1). Some aspects of this, around simulation setting 1 and simulation setting 6, were presented in the main paper

Simulation setting 1

Continuous outcome Y, binary exposure $group$, continuous covariate x and interaction term between the exposure and the covariate. Balanced with a 0.5 exposure probability, while the distribution of the continuous covariate did not vary across studies. Fixed within-study variance, and normally distributed random effects. Results presented in main paper.

Simulation setting 2

Introducing a modest level of publication bias. Studies ranked on size and effect size, with 20% of the studies ranked the lowest on both parameters being dropped.

Simulation setting 3

Varying within-study variance across studies.

Simulation setting 4

Skew-normal distribution for the random effects (skew=1 and kurtosis=4).

Simulation setting 5

Including an interaction term between the binary exposure and continuous covariate, which becomes the focus.

Simulation setting 6

Including a binary rather than a continuous covariate, with a balanced 0.5 probability.

Simulation setting 7

As in simulation setting 6, but also allowing the levels of the binary covariate to vary greatly across studies from 0 to 100%.

Simulation setting 8

As in simulation setting 6, but also including heterogeneity (I^2=50%) for both the covariate and the interaction term.

Table 1: Interpreting the Y axis labels on all figures

Level	What is it	Additional information
One	The total number of patients and studies	1000/2, implies a total of 1000 patients over 2 studies
Two	I^2 levels for the intercept and exposure	0%-50%, implies no heterogeneity for the intercept and I^2=50% for the exposure
Legend	The 4 models used (3 one-stage, 1 two-stage)	One-stage A: fixed common intercept, random exposure effect, and fixed effect for the covariate<br><br>One-stage B: fixed study-specific intercepts, random exposure effect, and fixed study-specific effects for the covariate<br><br>One-stage C: random study intercept, random exposure effect, and fixed study-specific effects for the covariate.<br><br>Two-stage: called through *ipdmetan*, pooling study results using a restricted maximum likelihood random-effects meta-analysis model

Simulation setting 1

Figure A1: Mean Bias 5
Figure A2: Mean Error 6
Figure A3: Coverage probability (%), against 95% nominal line 7
Figure A4: Power probability (%) 8
Figure A5: Coverage and Power (%), plotted together [(coverage+power)/2] 9
Figure A6: Model convergence (%) 10

Simulation setting 2

Figure A7: Mean Bias 11
Figure A8: Mean Error 12
Figure A9: Coverage probability (%), against 95% nominal line 13
Figure A10: Power probability (%) 14
Figure A11: Coverage and Power (%), plotted together [(coverage+power)/2] 15
Figure A12: Model convergence (%) 16

Simulation setting 3

Figure A13: Mean Bias 17
Figure A14: Mean Error 18
Figure A15: Coverage probability (%), against 95% nominal line 19
Figure A16: Power probability (%) 20
Figure A17: Coverage and Power (%), plotted together [(coverage+power)/2] 21
Figure A18: Model convergence (%) 22

Simulation setting 4

Figure A19: Mean Bias 23
Figure A20: Mean Error 24
Figure A21: Coverage probability (%), against 95% nominal line 25
Figure A22: Power probability (%) 26
Figure A23: Coverage and Power (%), plotted together [(coverage+power)/2] 27
Figure A24: Model convergence (%) 28

Simulation setting 5

Figure A25: Mean Bias 29
Figure A26: Mean Error 30
Figure A27: Coverage probability (%), against 95% nominal line 31
Figure A28: Power probability (%) 32
Figure A29: Coverage and Power (%), plotted together [(coverage+power)/2] 33
Figure A30: Model convergence (%) 34

Simulation setting 6

Figure A31: Mean Bias 35
Figure A32: Mean Error 36
Figure A33: Coverage probability (%), against 95% nominal line 37
Figure A34: Power probability (%) 38
Figure A35: Coverage and Power (%), plotted together [(coverage+power)/2] 39
Figure A36: Model convergence (%) 40

Simulation setting 7

Figure A37: Mean Bias 41
Figure A38: Mean Error 42
Figure A39: Coverage probability (%), against 95% nominal line 43

Figure A40: Power probability (%) 44
Figure A41: Coverage and Power (%), plotted together [(coverage+power)/2] 45
Figure A42: Model convergence (%) 46

Simulation setting 8

Figure A43: Mean Bias, models A-D 47
Figure A44: Mean Bias, models D & E-G 48
Figure A45: Mean Error, models A-D 49
Figure A46: Mean Error, models D & E-G 50
Figure A47: Coverage probability (%), against 95% nominal line, models A-D 51
Figure A48: Coverage probability (%), against 95% nominal line, models D & E-G 52
Figure A49: Power probability (%), models A-D 53
Figure A50: Power probability (%), models D & E-G 54
Figure A51: Coverage and Power (%), plotted together [(coverage+power)/2], models A-D 55
Figure A52: Coverage and Power (%), plotted together [(coverage+power)/2], models D & E-G 56
Figure A53: Model convergence (%), models A-D 57
Figure A54: Model convergence (%), models D & E-G 58

Simulation setting 1

Figure A1: Mean Bias

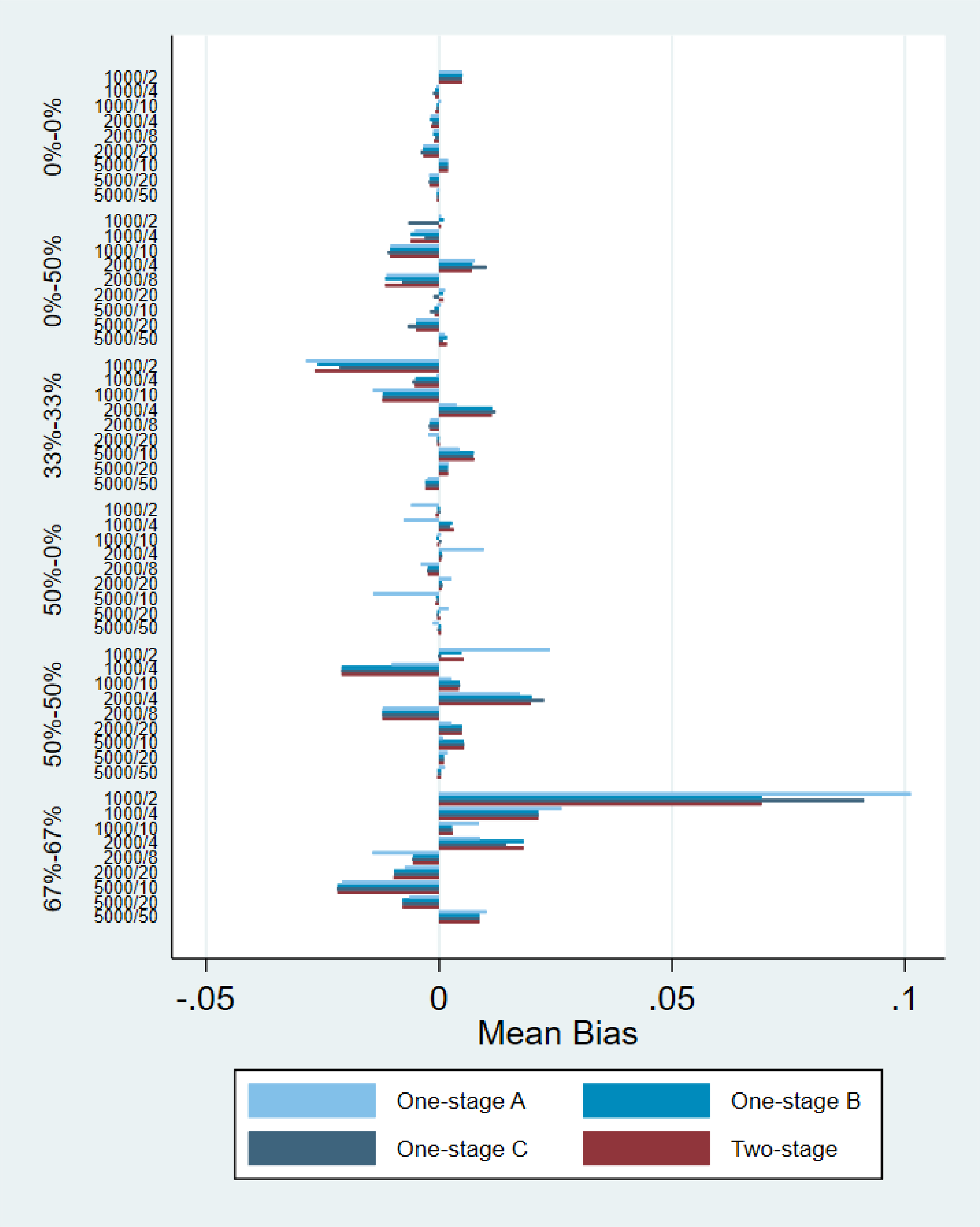

Simulation setting 1

Figure A2: Mean Error

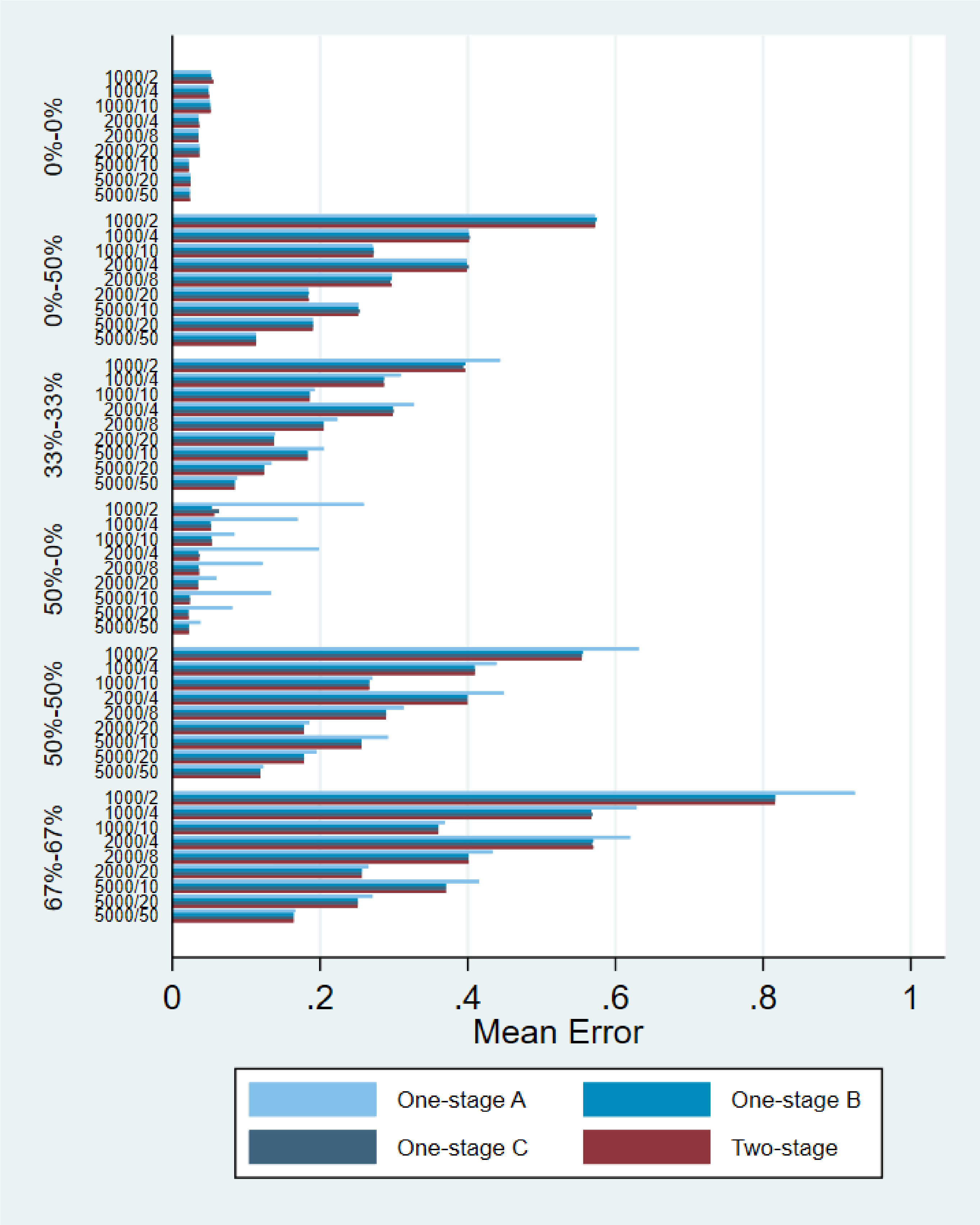

Simulation setting 1

Figure A3: Coverage probability (%), against 95% nominal line

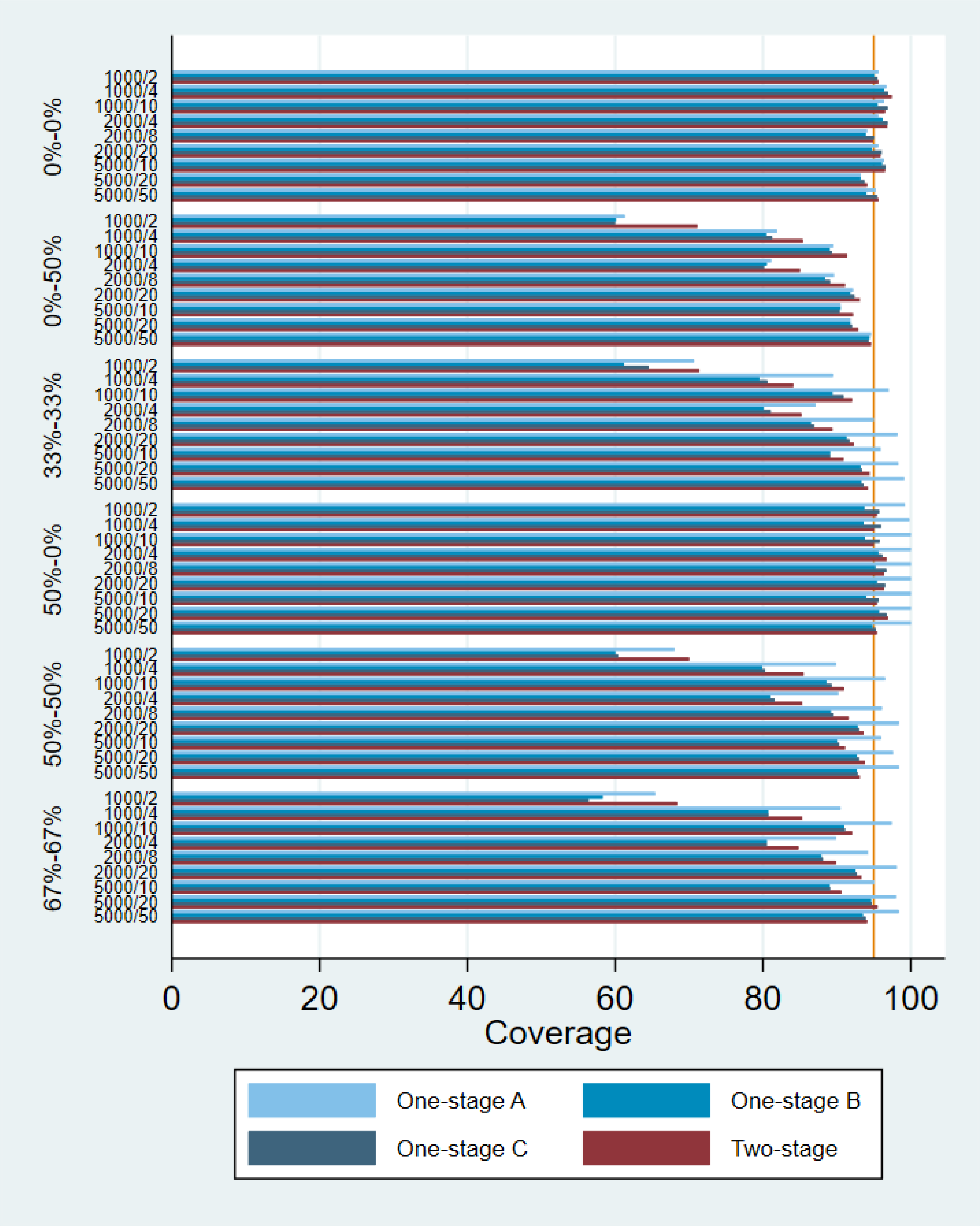

Simulation setting 1

Figure A4: Power probability (%)

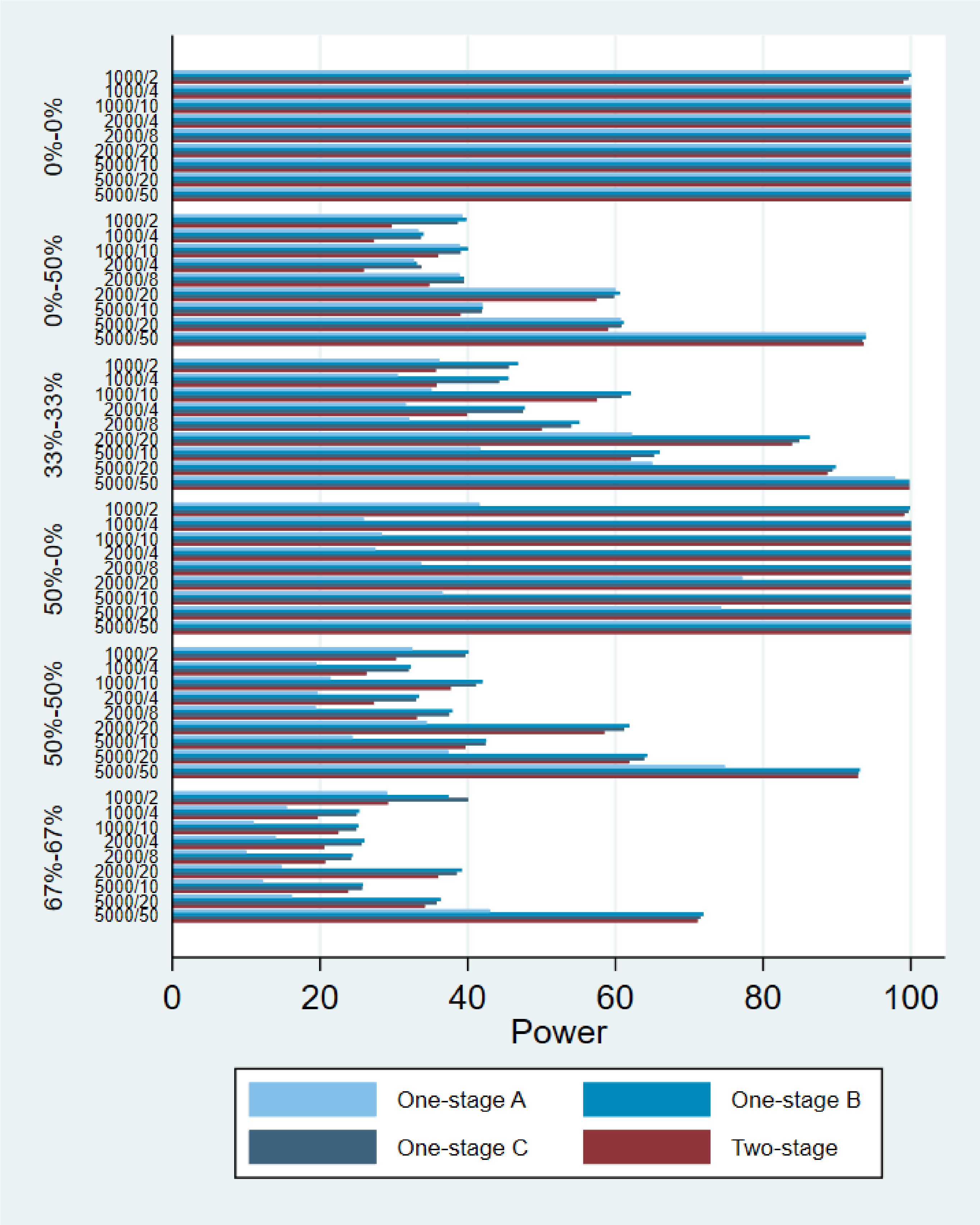

Simulation setting 1

Figure A5: Coverage and Power (%), plotted together [(coverage+power)/2]

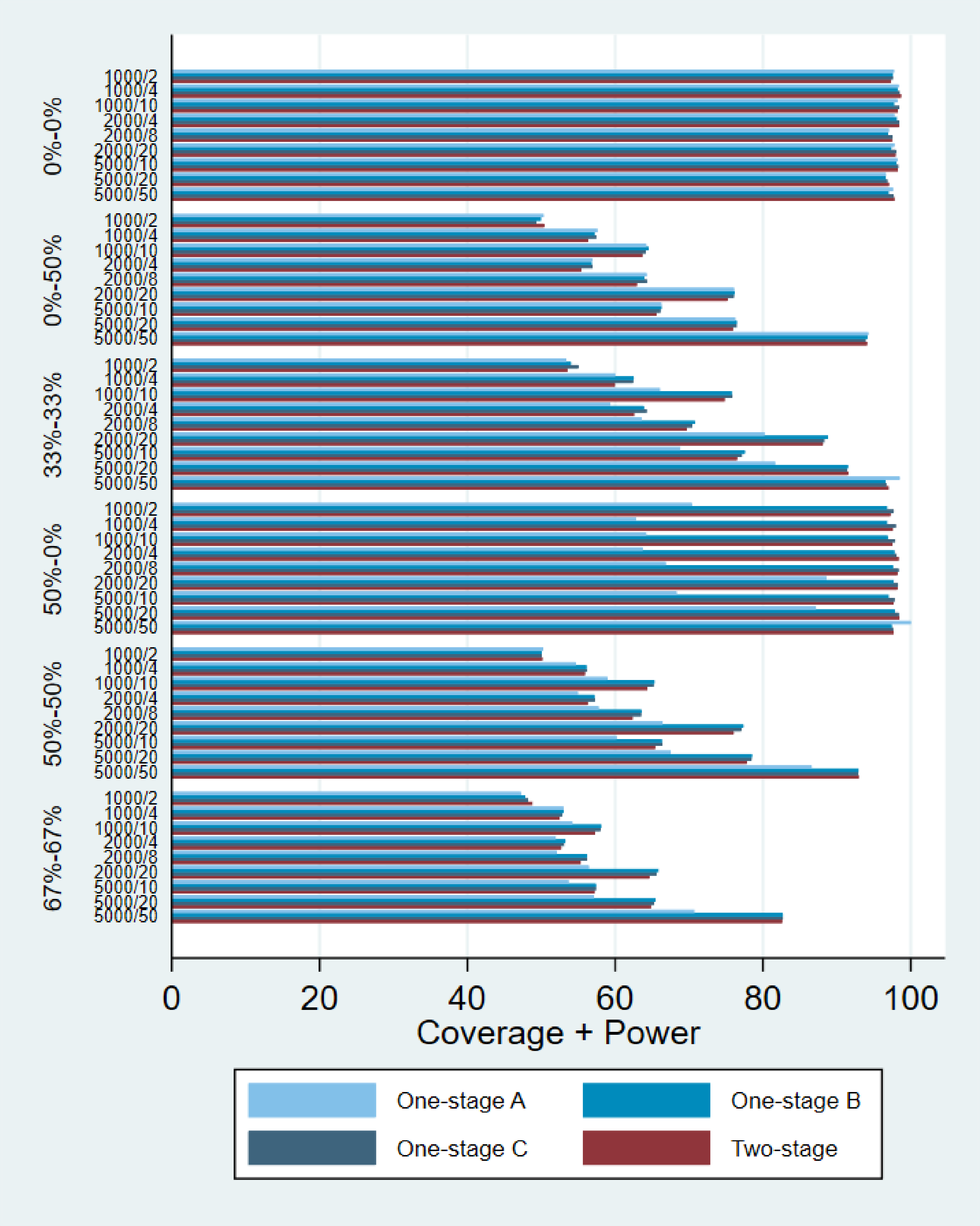

Simulation setting 1

Figure A6: Model convergence (%)

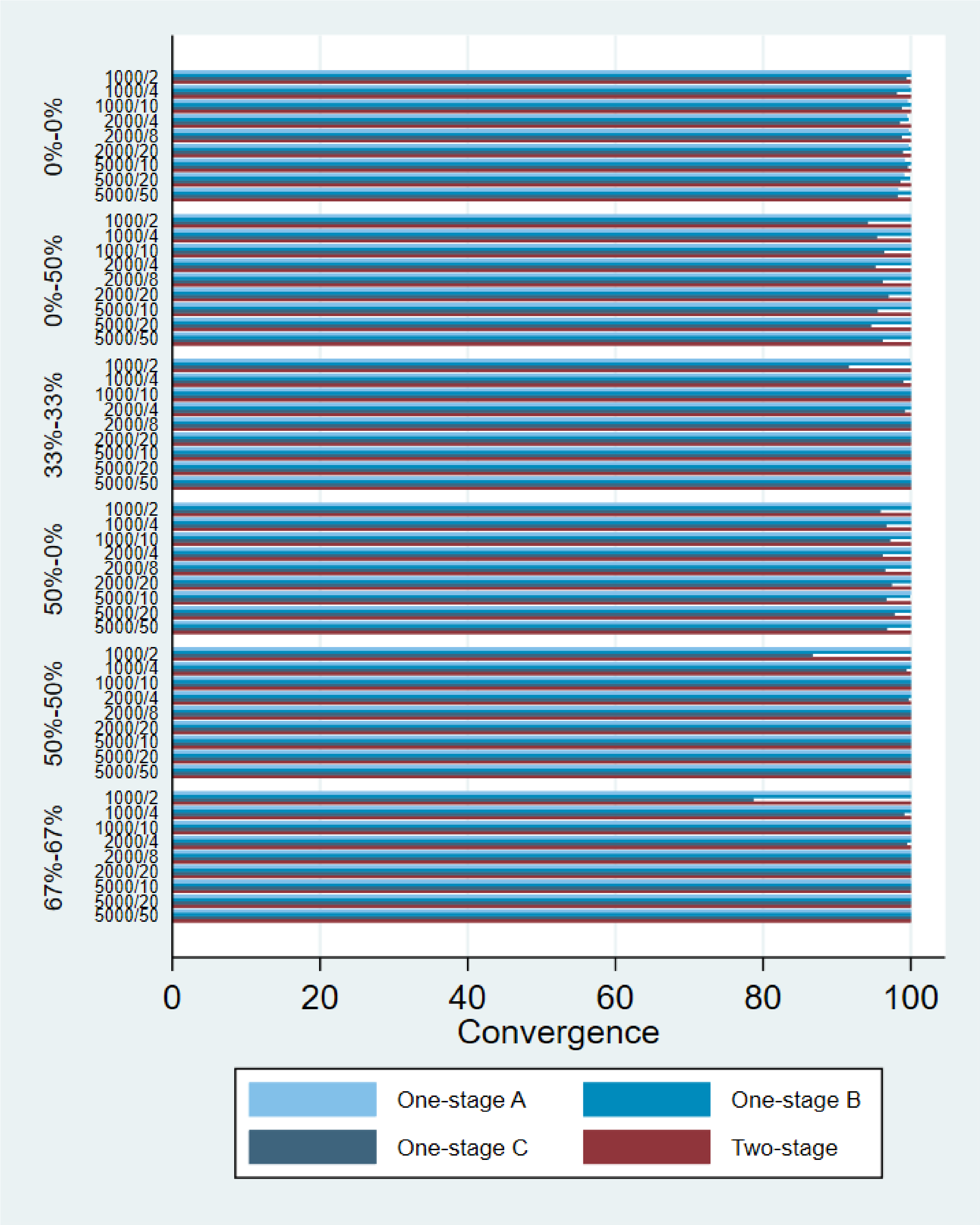

Simulation setting 2

Figure A7: Mean Bias

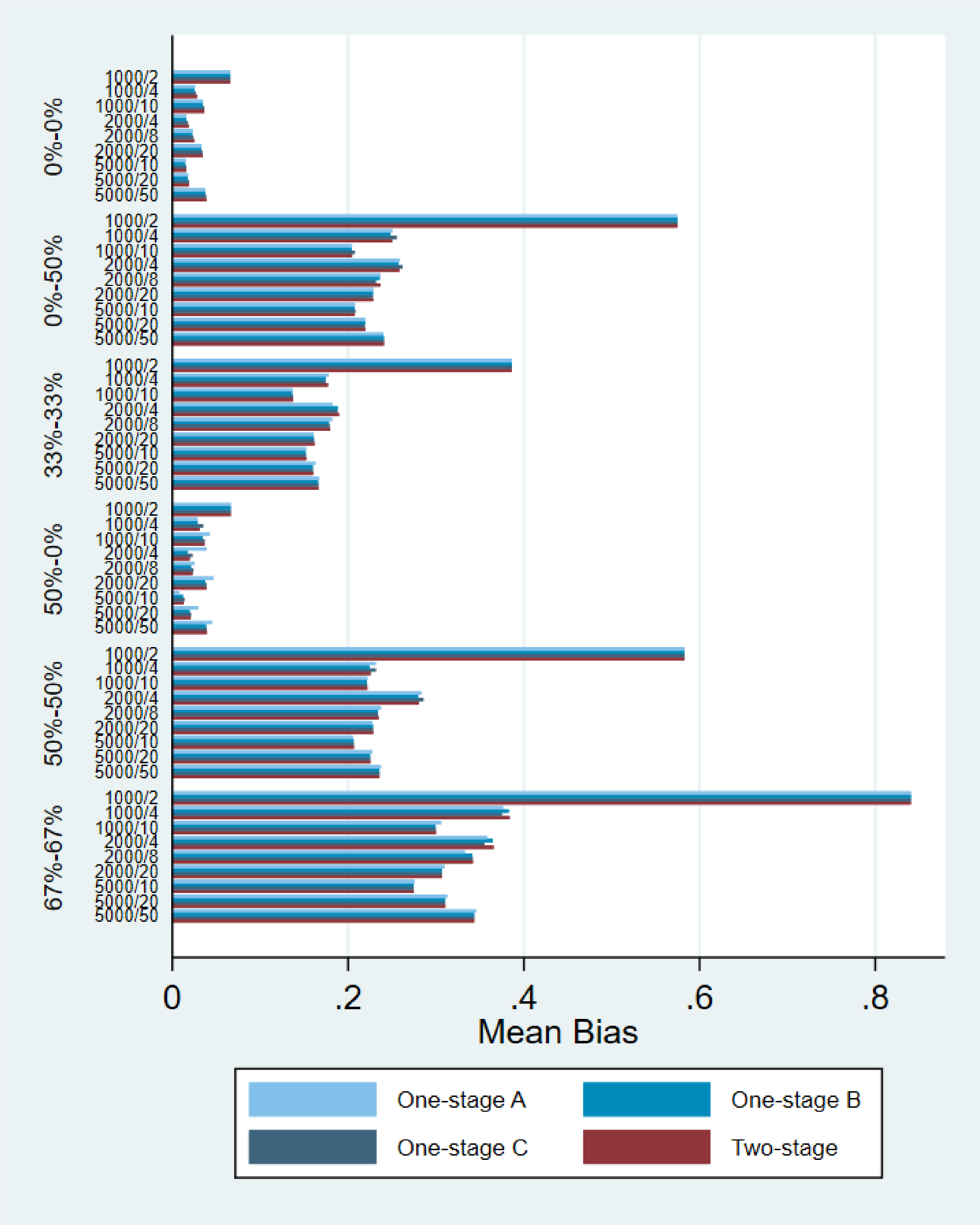

Simulation setting 2

Figure A8: Mean Error

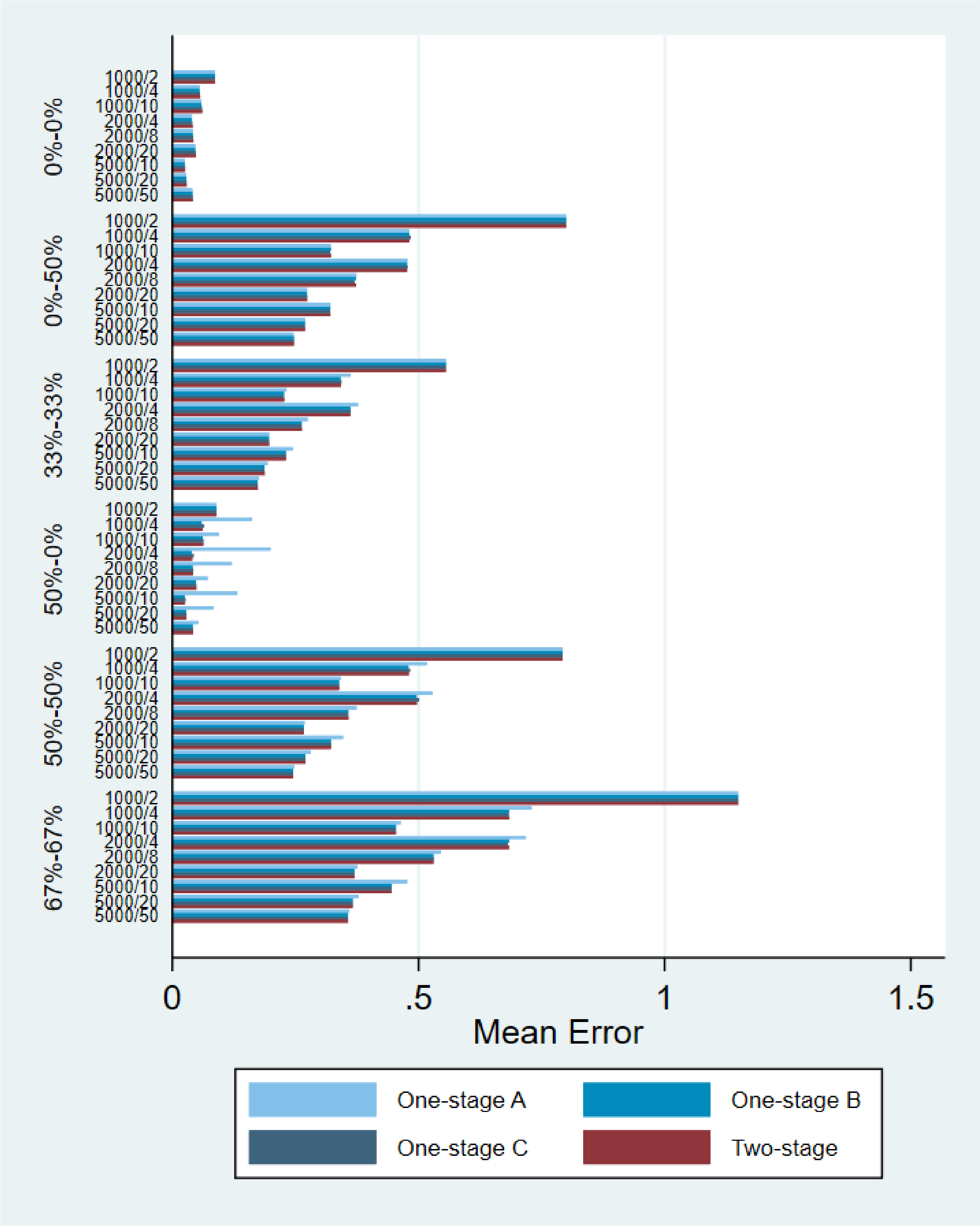

Simulation setting 2

Figure A9: Coverage probability (%), against 95% nominal line

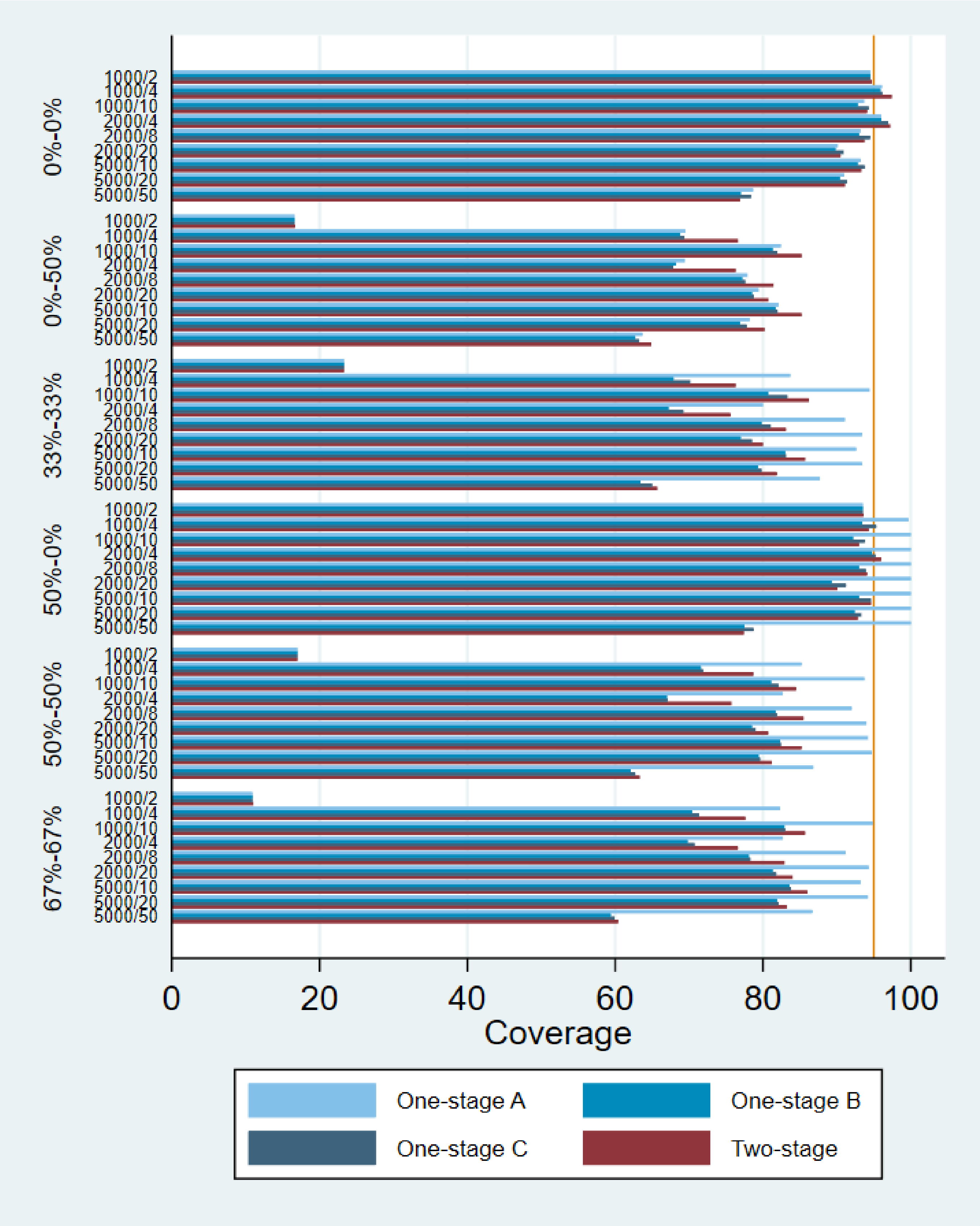

Simulation setting 2

Figure A10: Power probability (%)

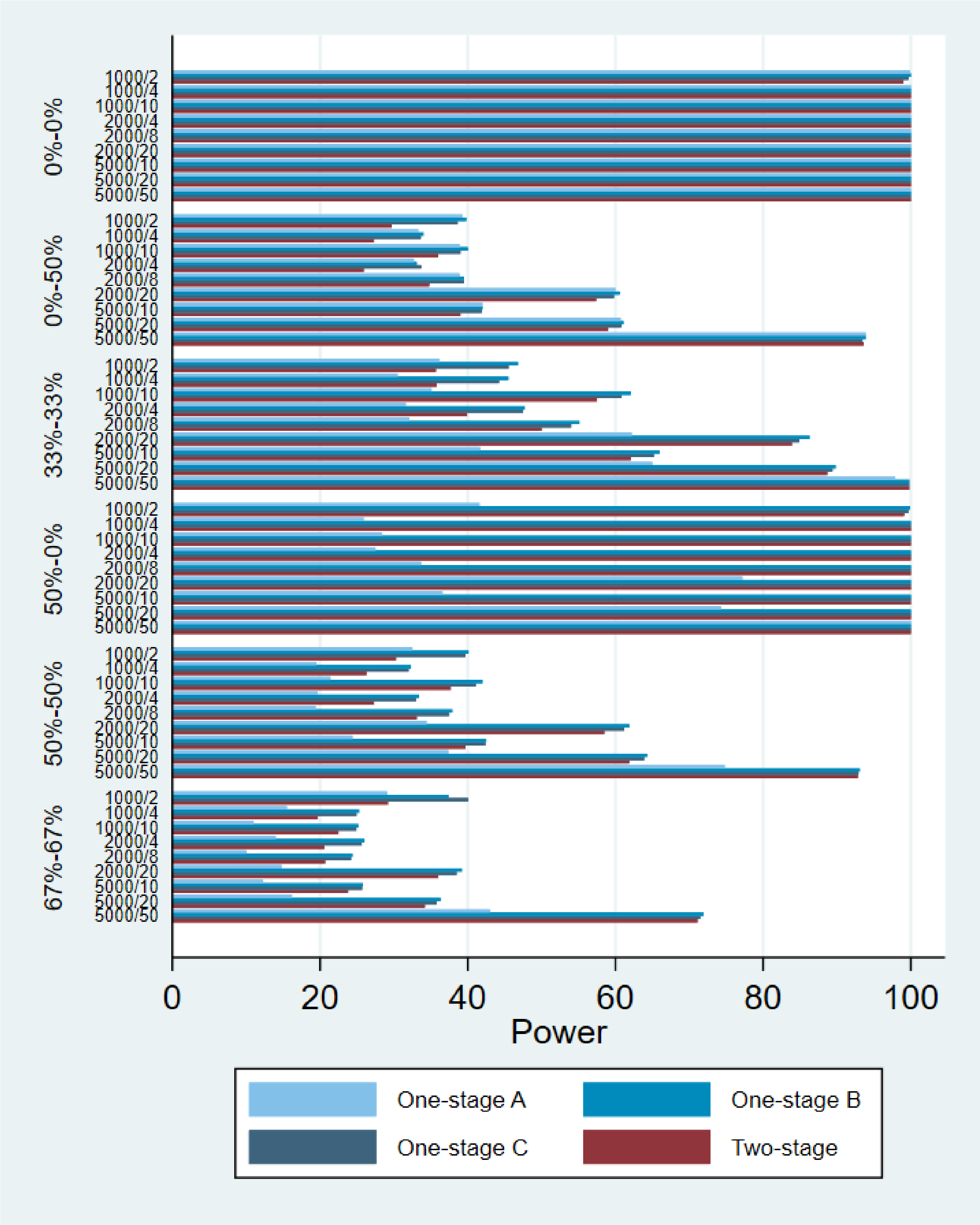

Simulation setting 2

Figure A11: Coverage and Power (%), plotted together [(coverage+power)/2]

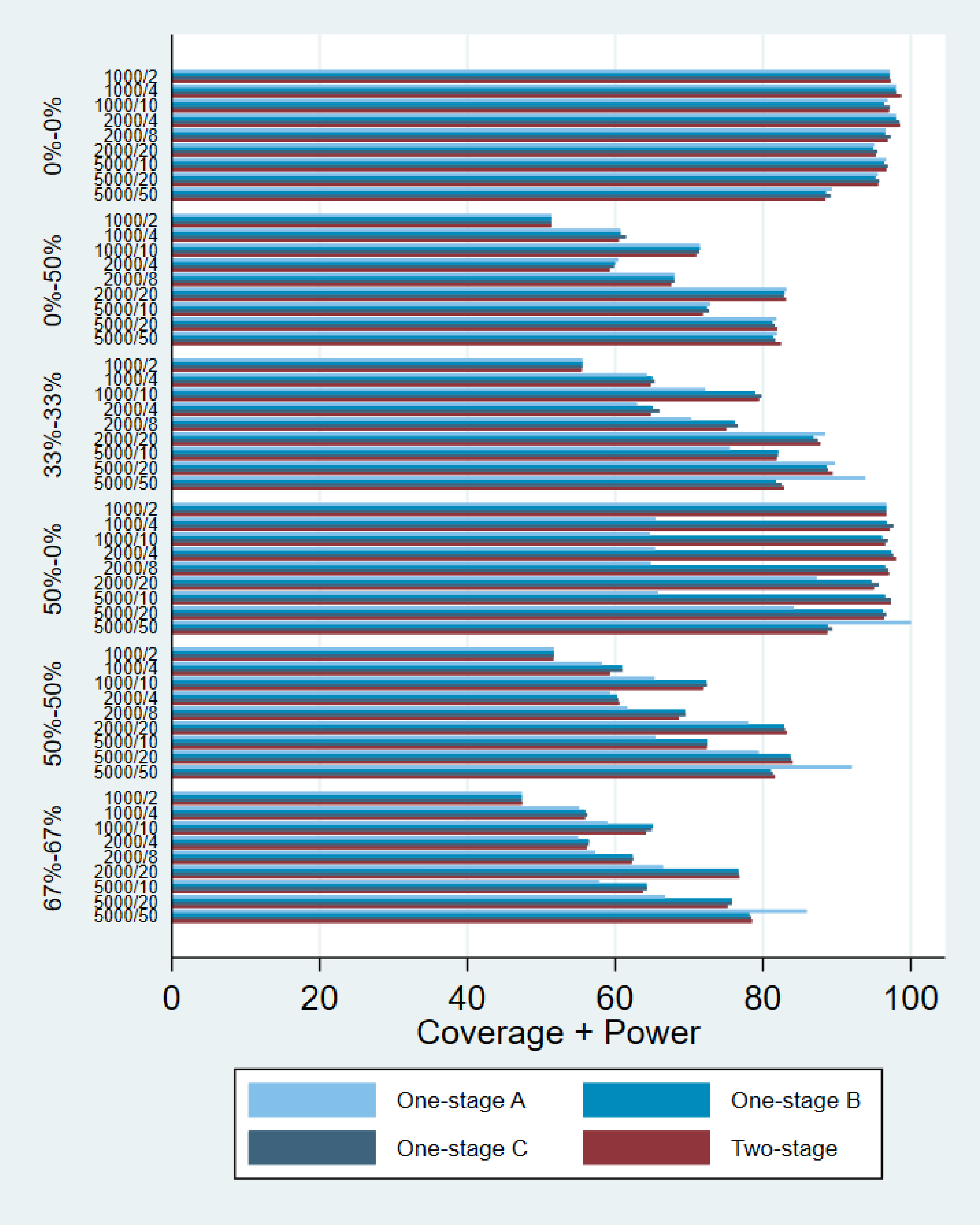

Simulation setting 2

Figure A12: Model convergence (%)

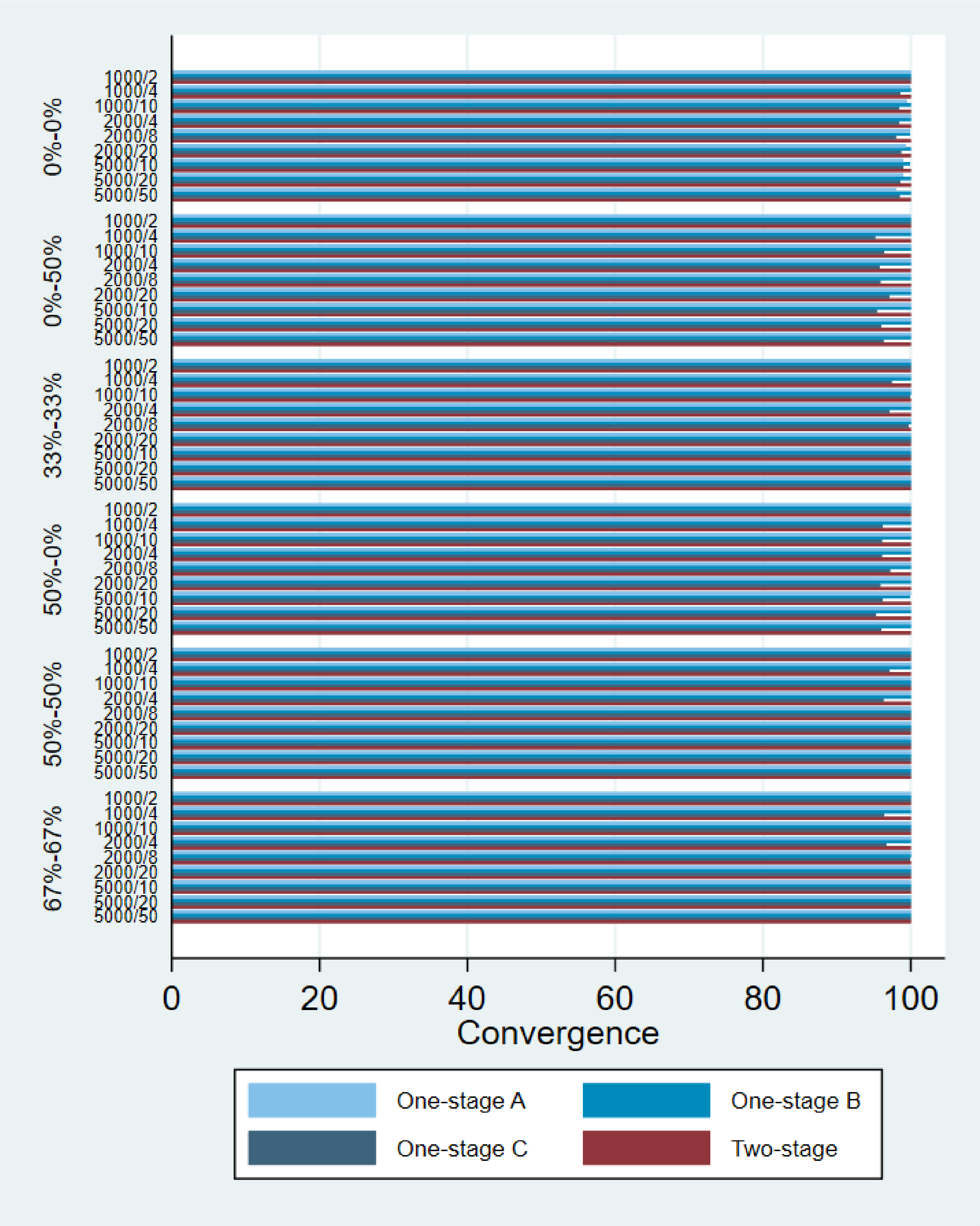

Simulation setting 3

Figure A13: Mean Bias

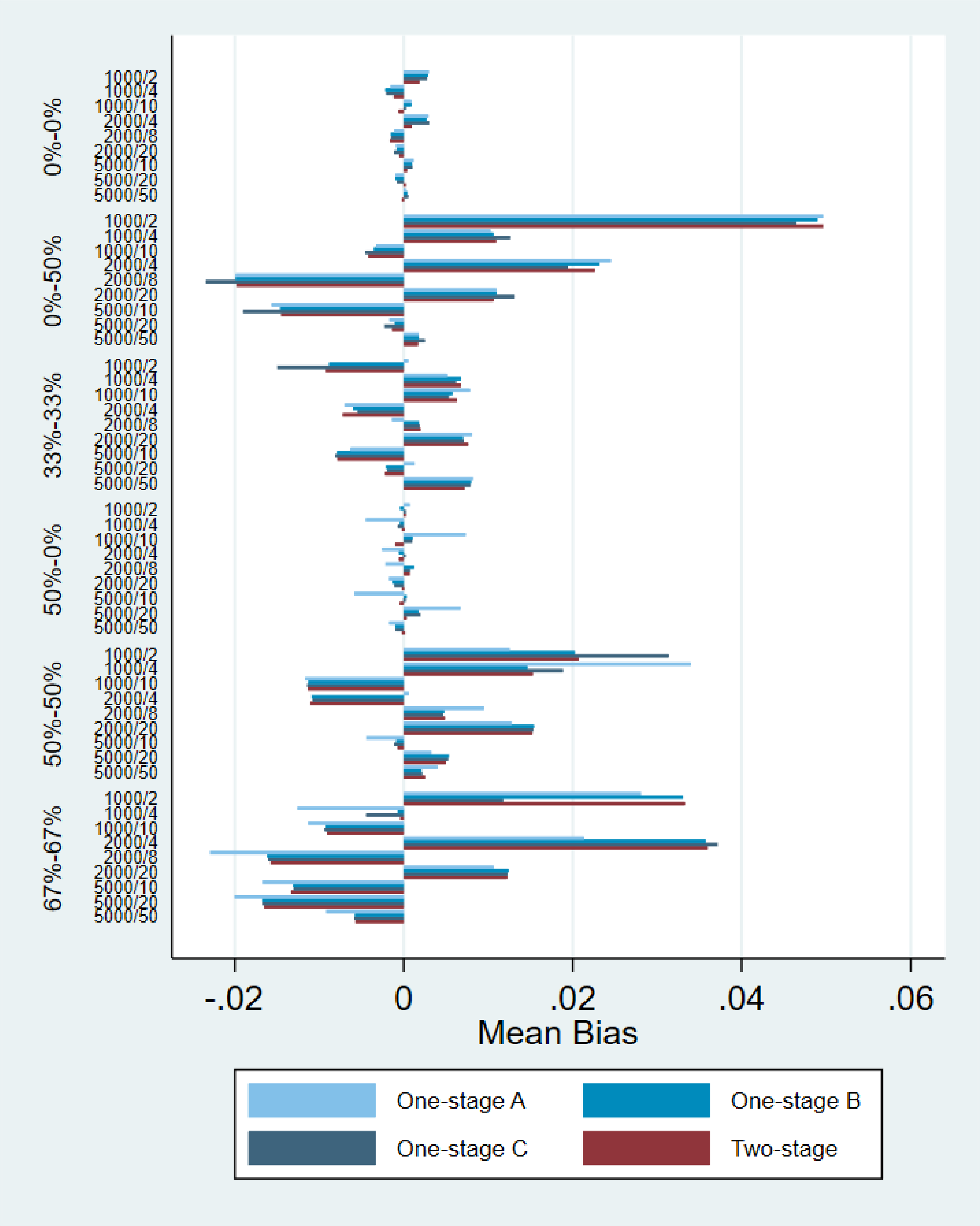

Simulation setting 3

Figure A14: Mean Error

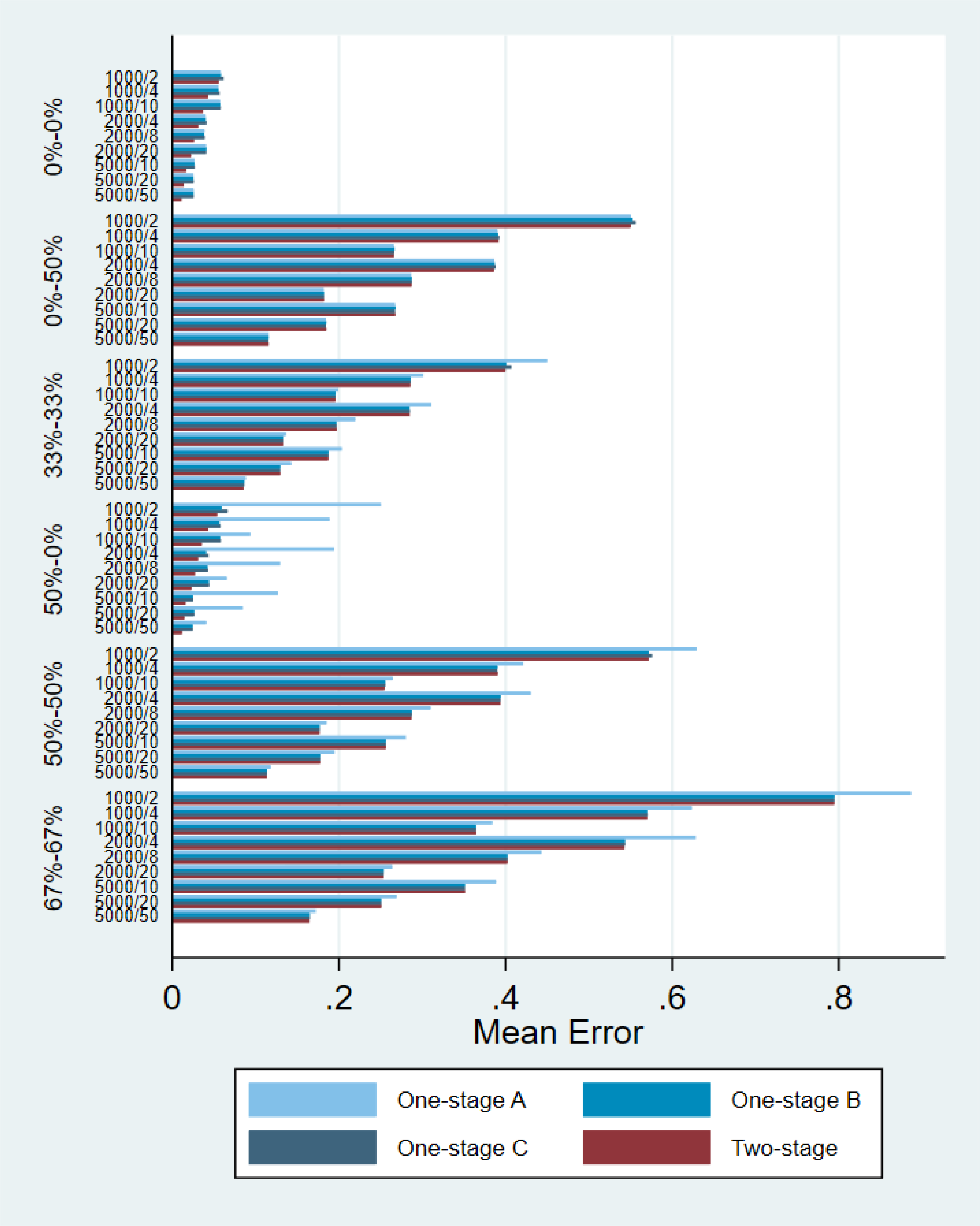

Simulation setting 3

Figure A15: Coverage probability (%), against 95% nominal line

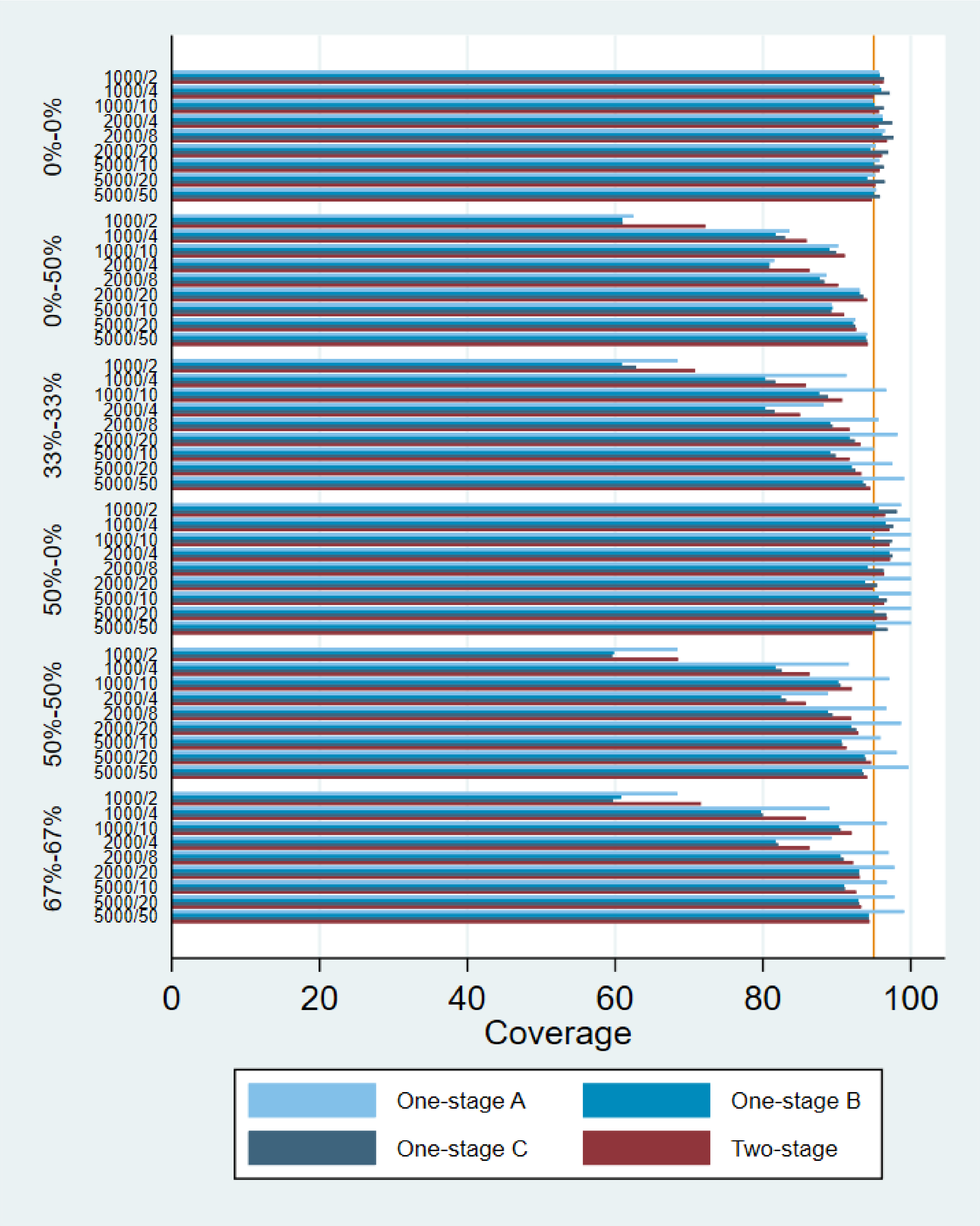

Simulation setting 3

Figure A16: Power probability (%)

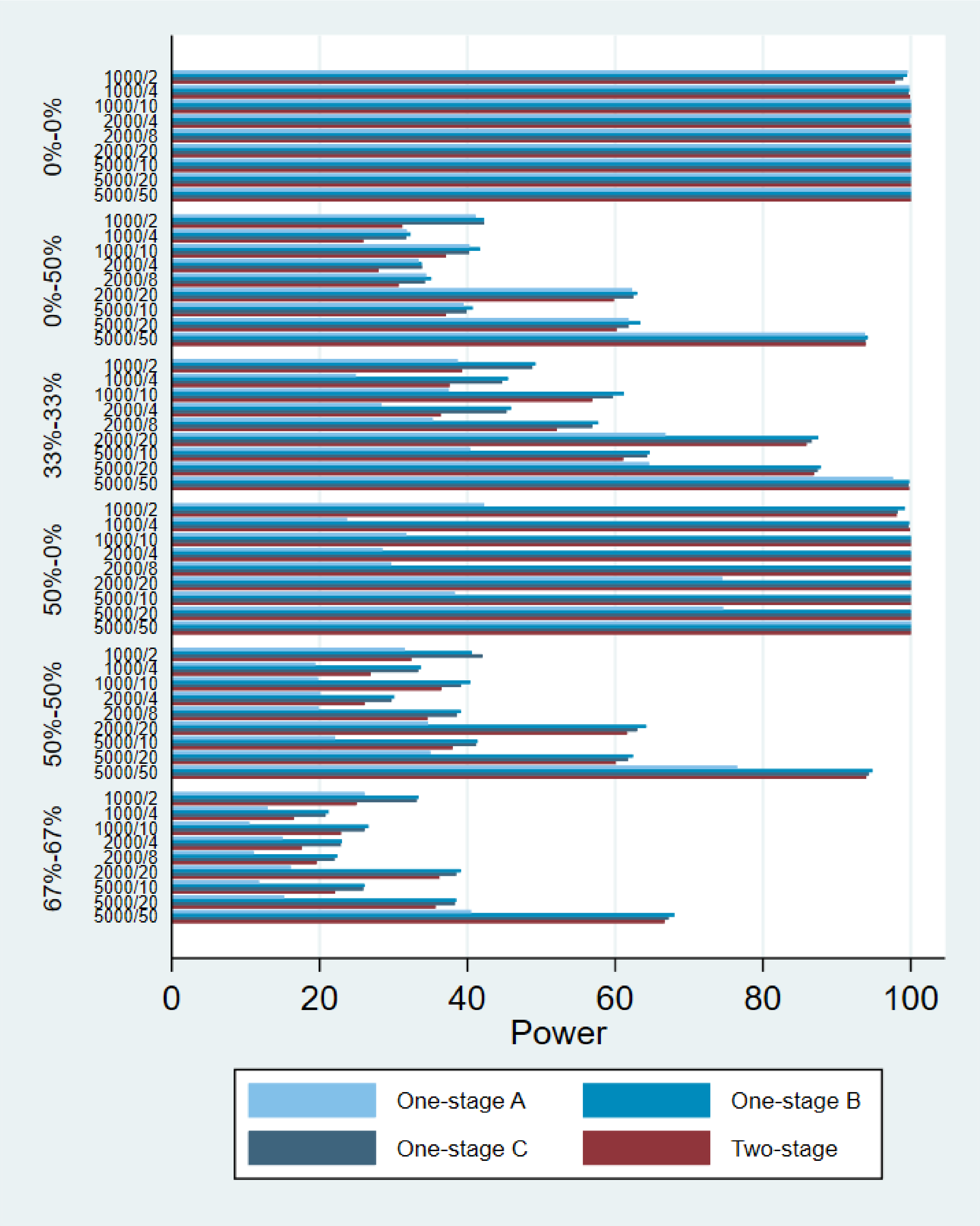

Simulation setting 3

Figure A17: Coverage and Power (%), plotted together [(coverage+power)/2]

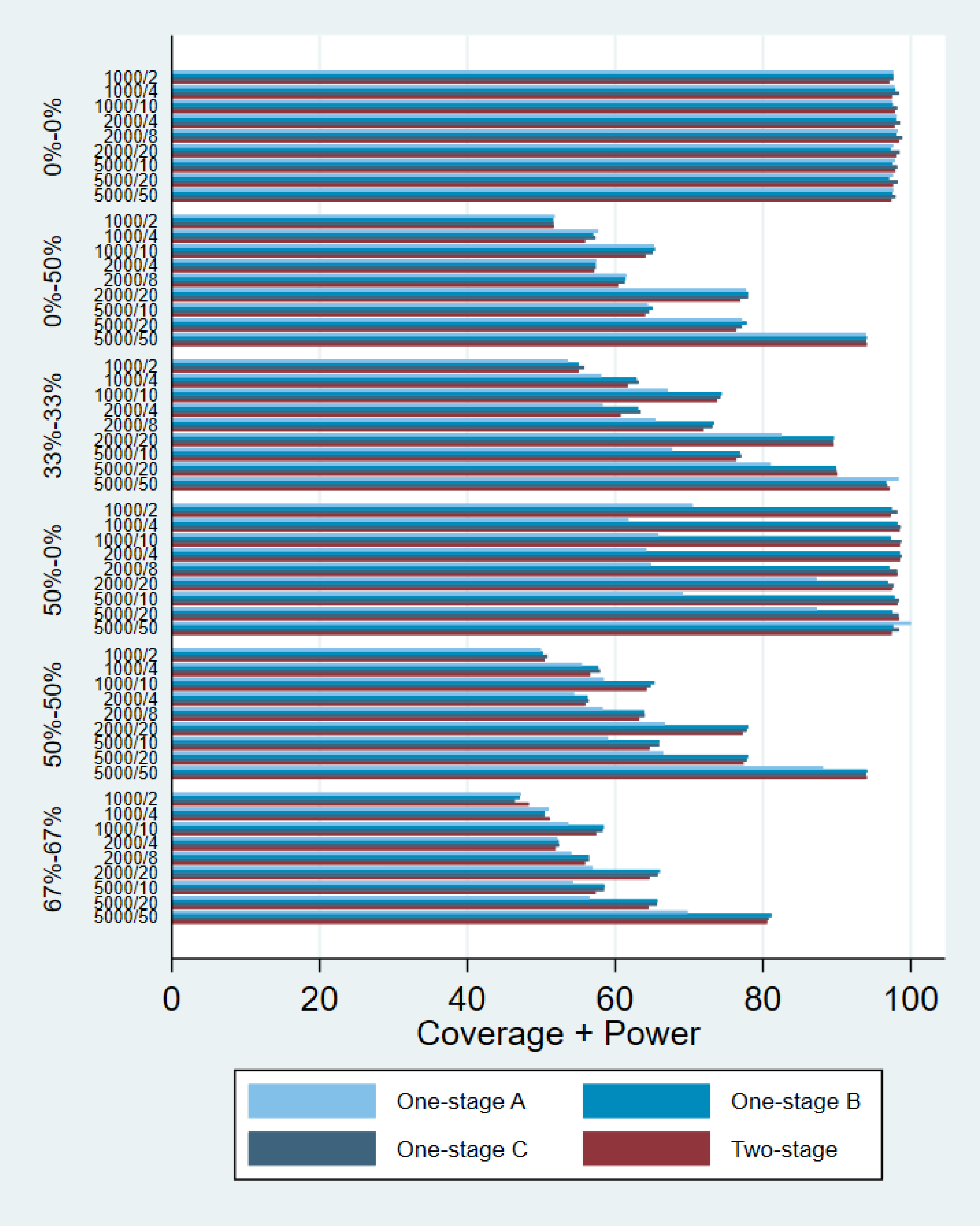

Simulation setting 3

Figure A18: Model convergence (%)

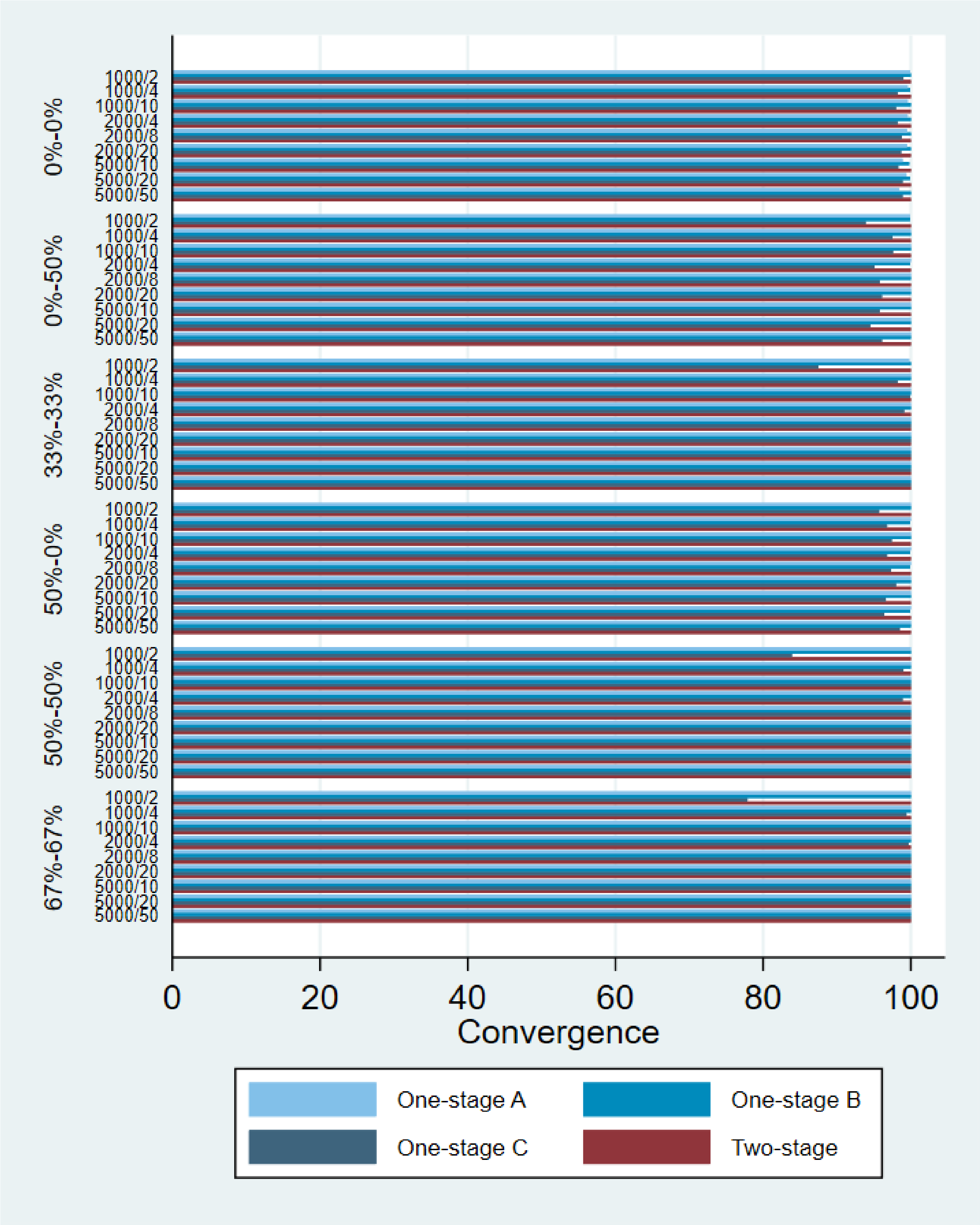

Simulation setting 4

Figure A19: Mean Bias

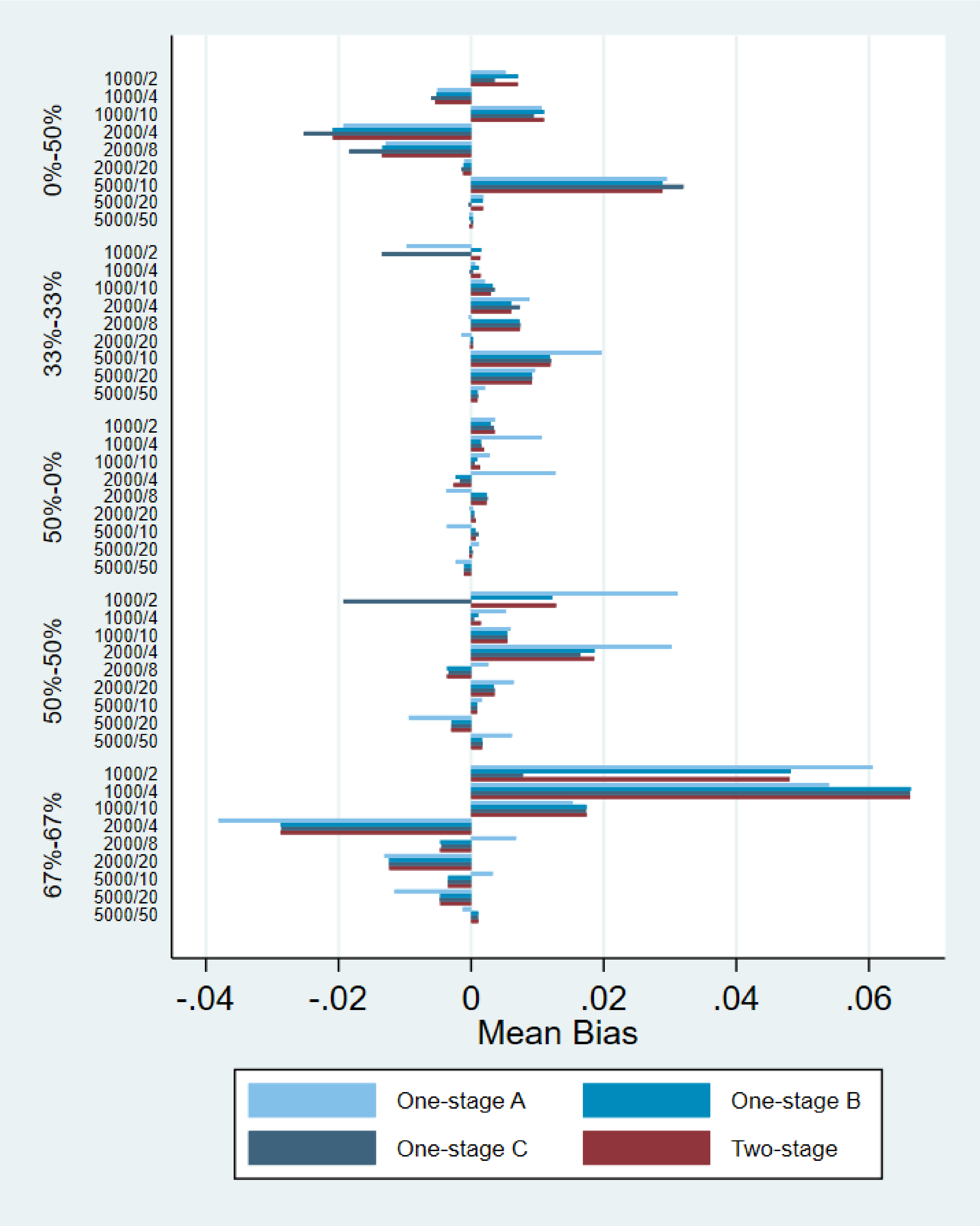

Simulation setting 4

Figure A20: Mean Error

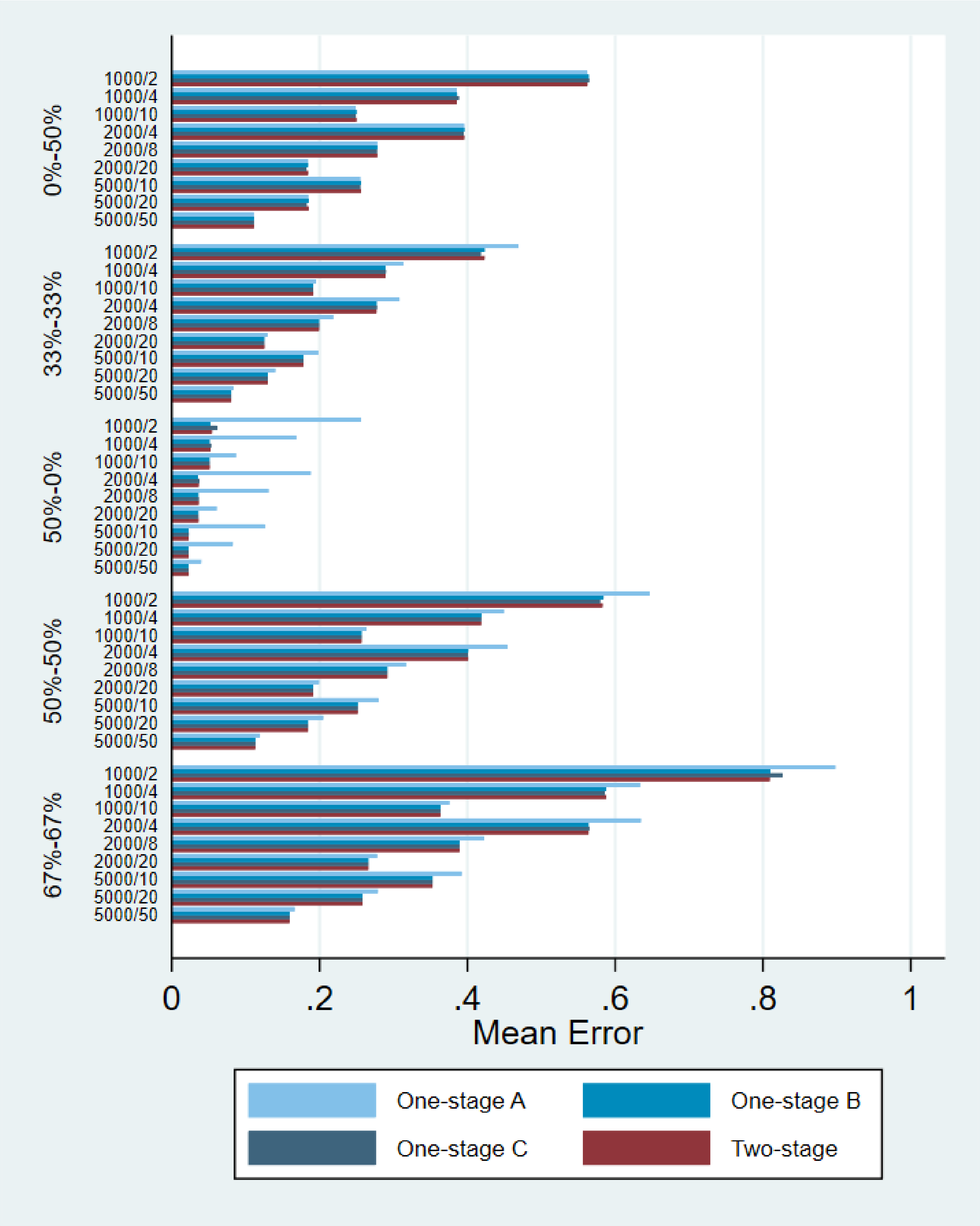

Simulation setting 4

Figure A21: Coverage probability (%), against 95% nominal line

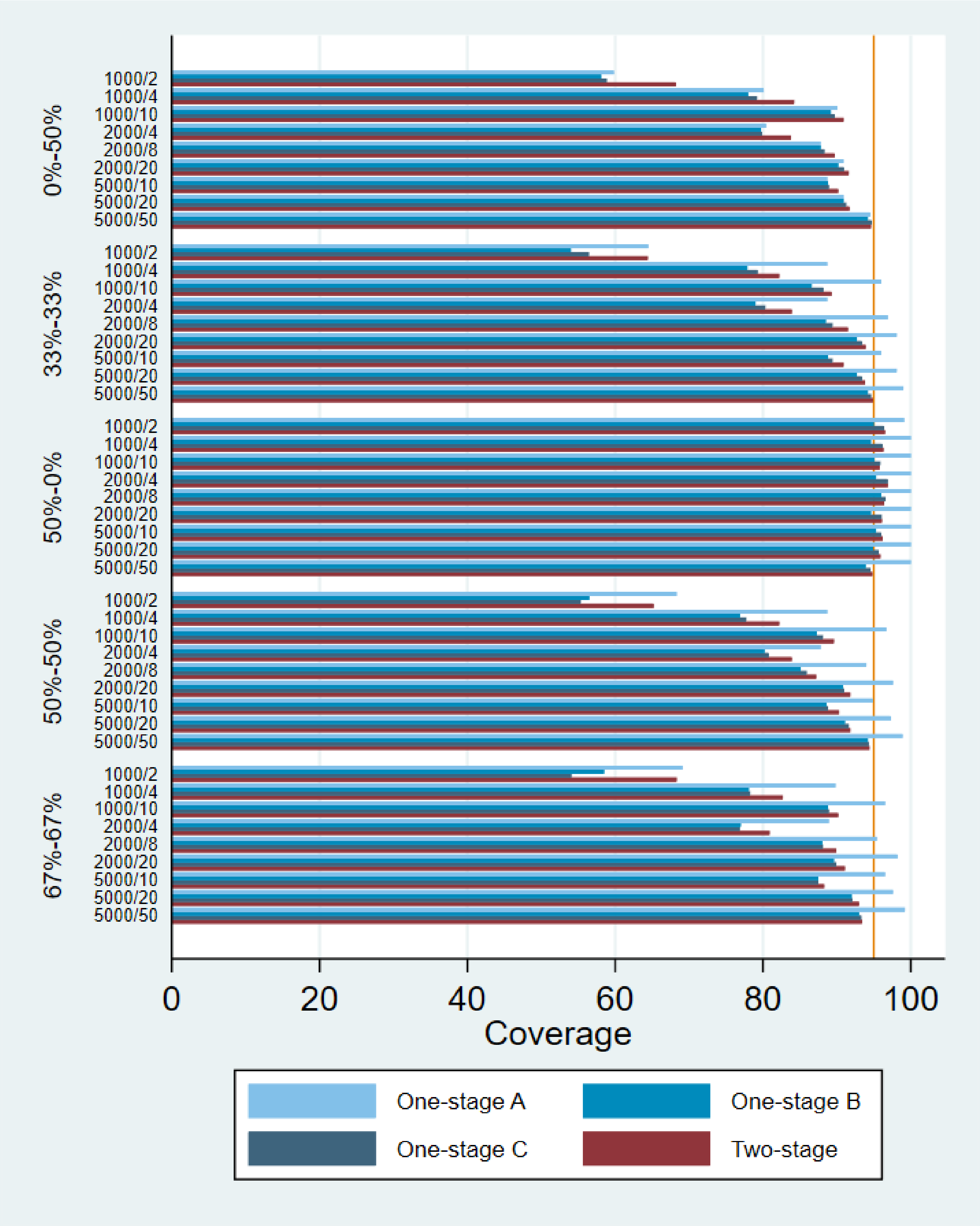

Simulation setting 4

Figure A22: Power probability (%)

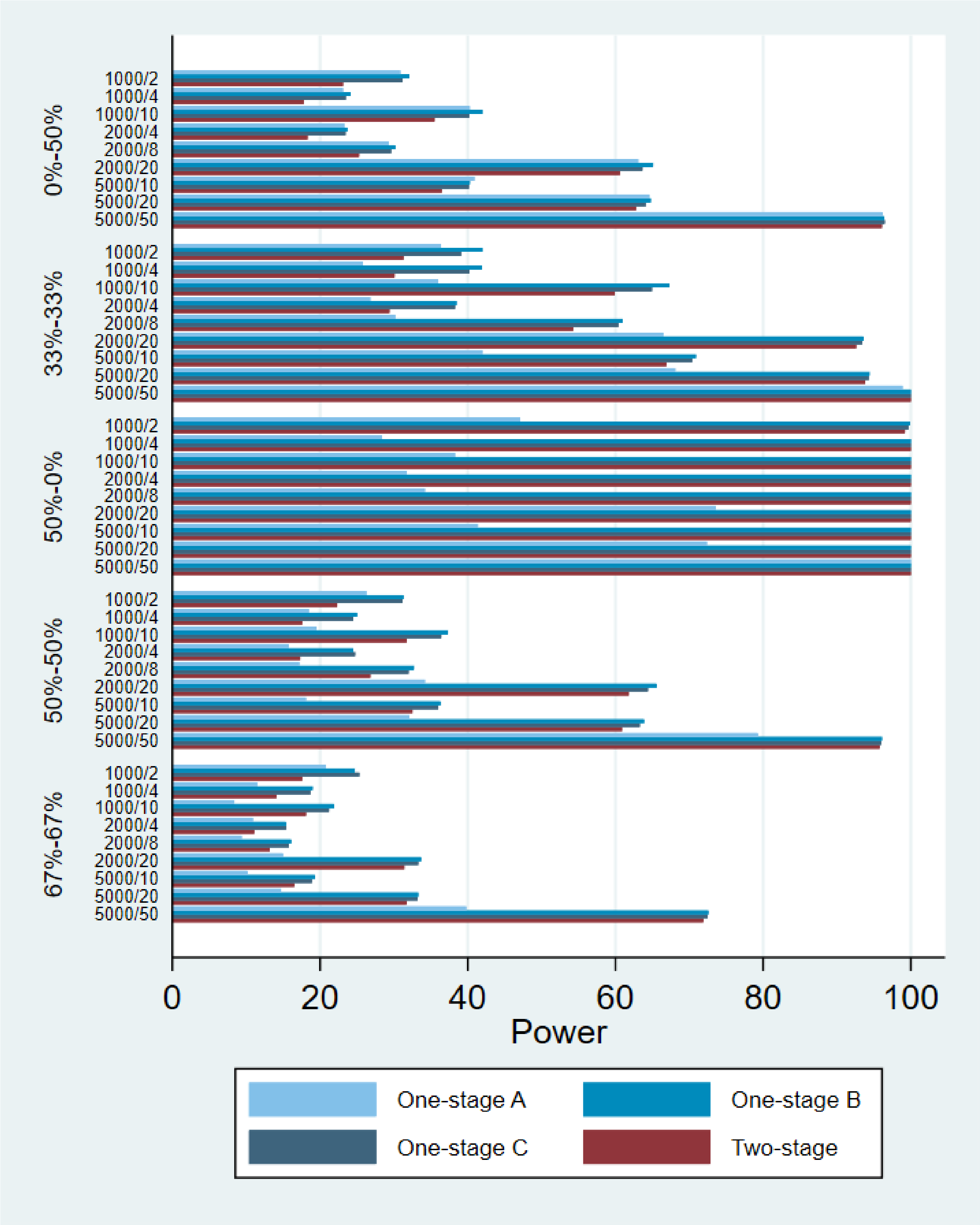

Simulation setting 4

Figure A23: Coverage and Power (%), plotted together [(coverage+power)/2]

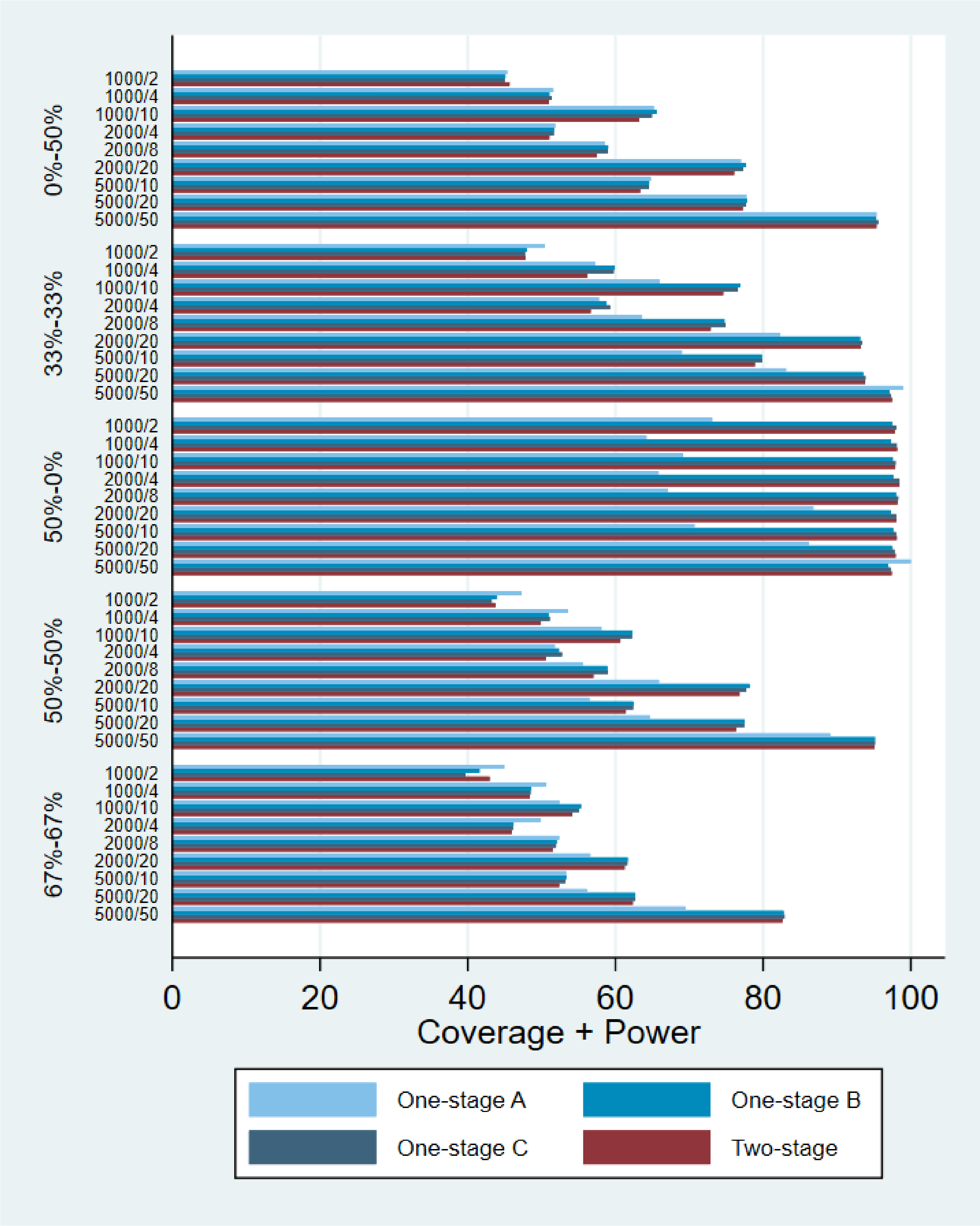

Simulation setting 4

Figure A24: Model convergence (%)

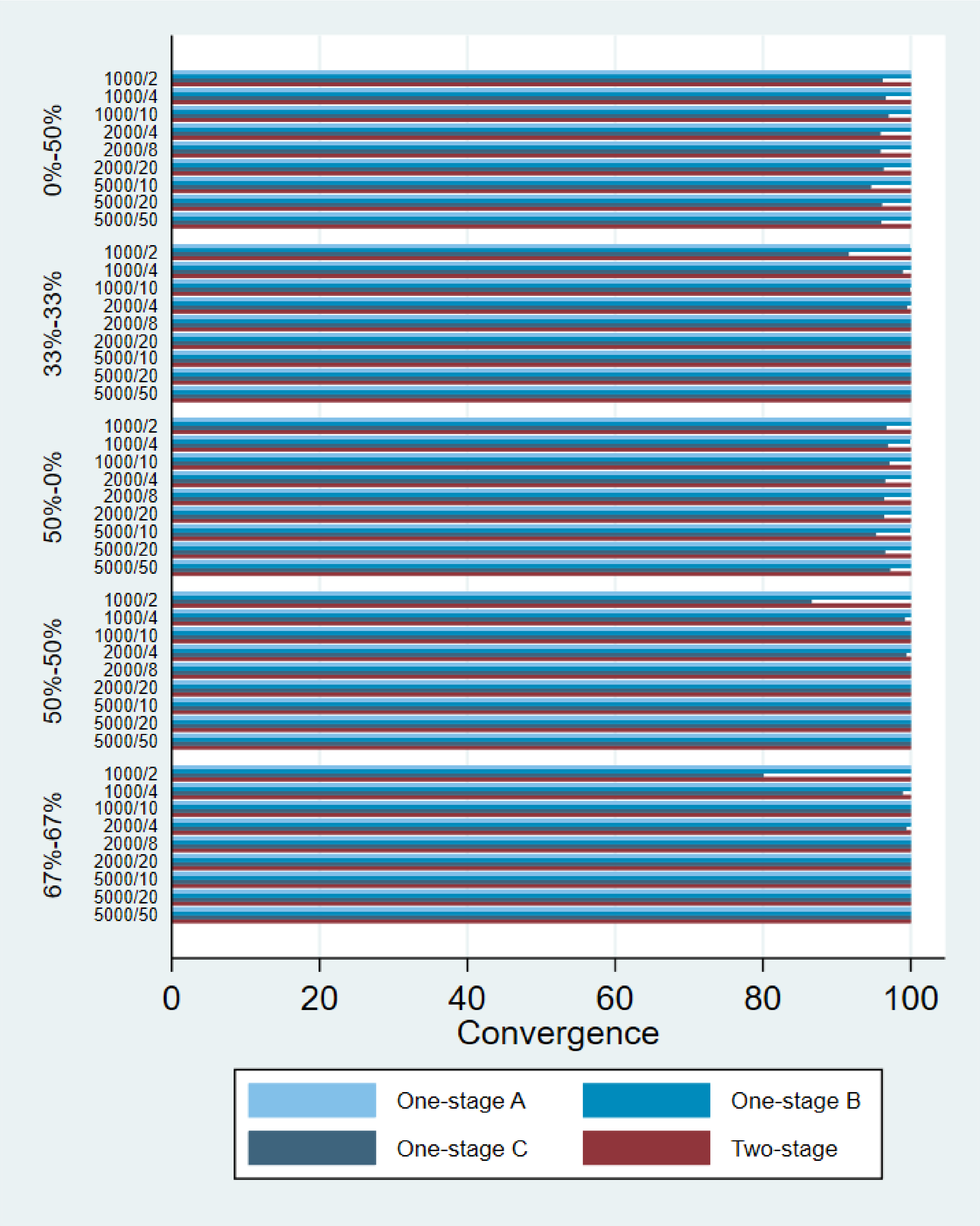

Simulation setting 5

Figure A25: Mean Bias

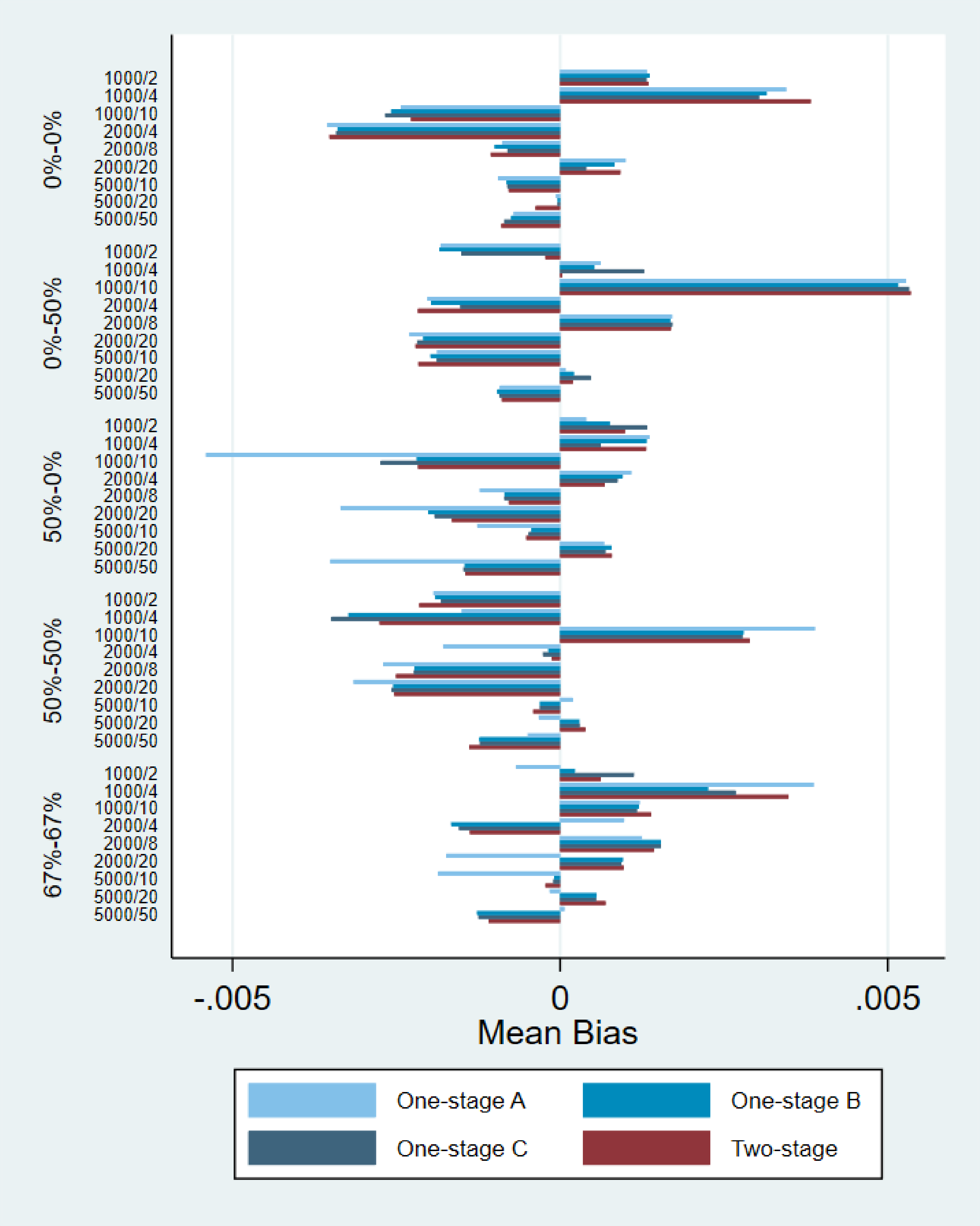

Simulation setting 5

Figure A26: Mean Error

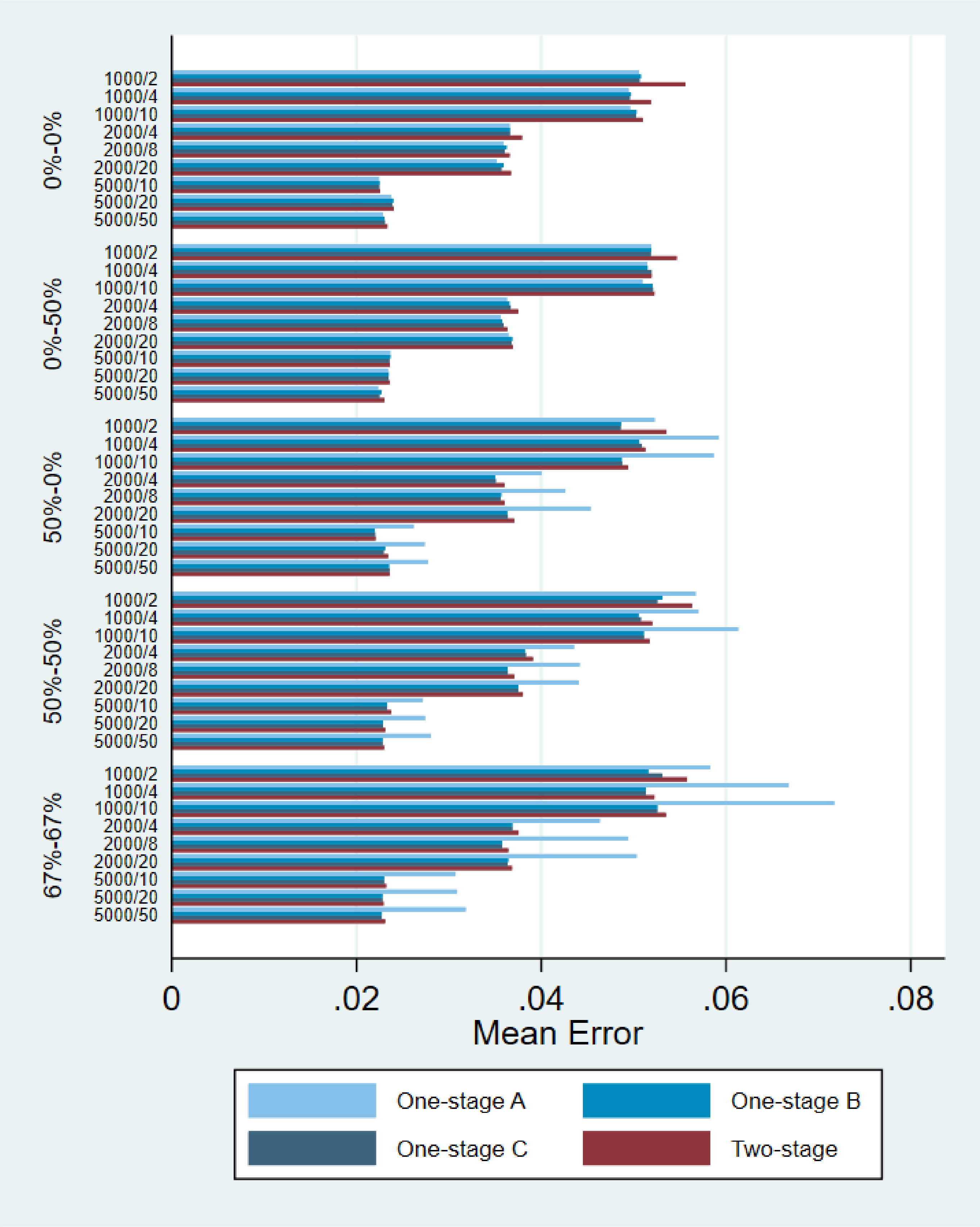

Simulation setting 5

Figure A27: Coverage probability (%), against 95% nominal line

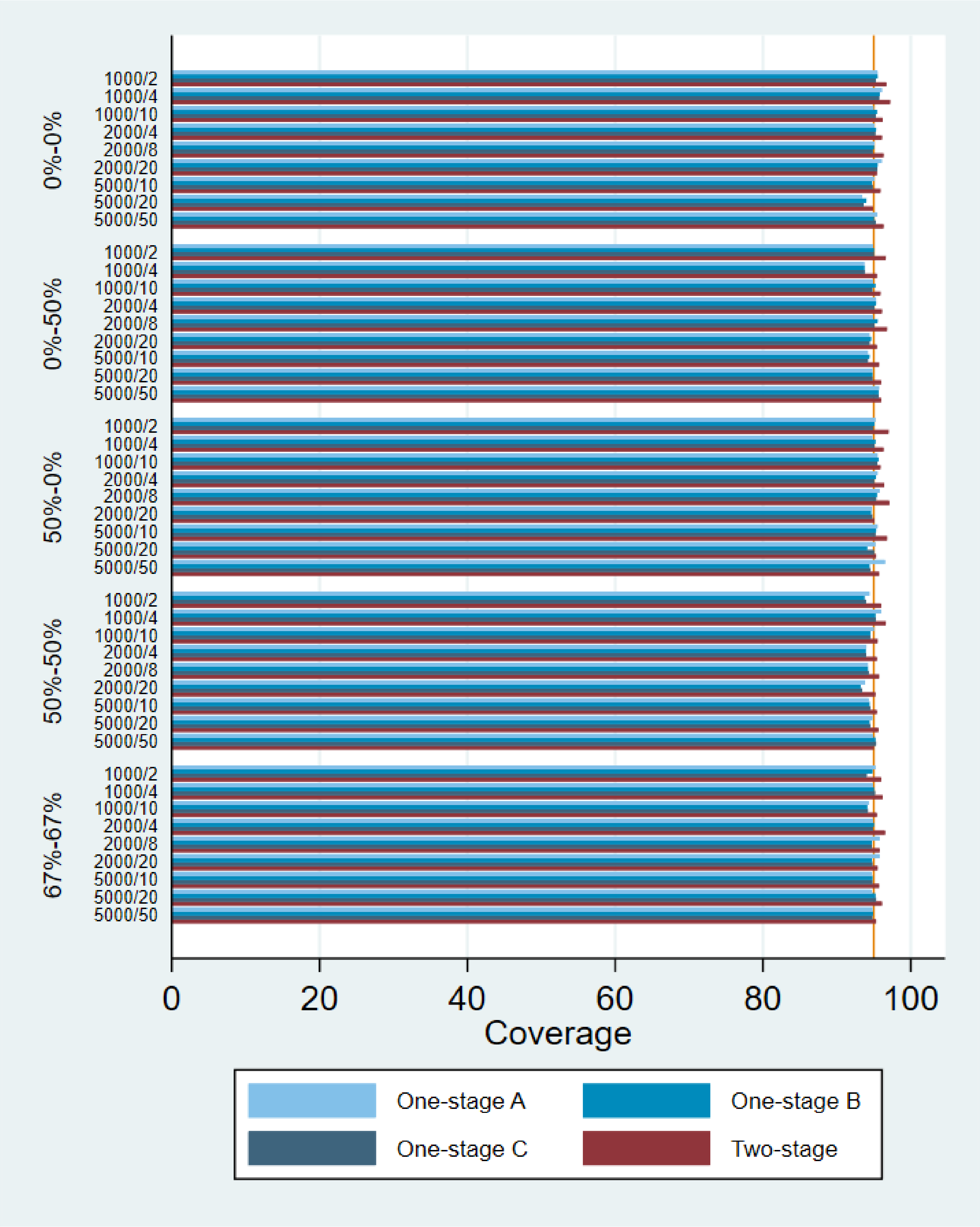

Simulation setting 5

Figure A28: Power probability (%)

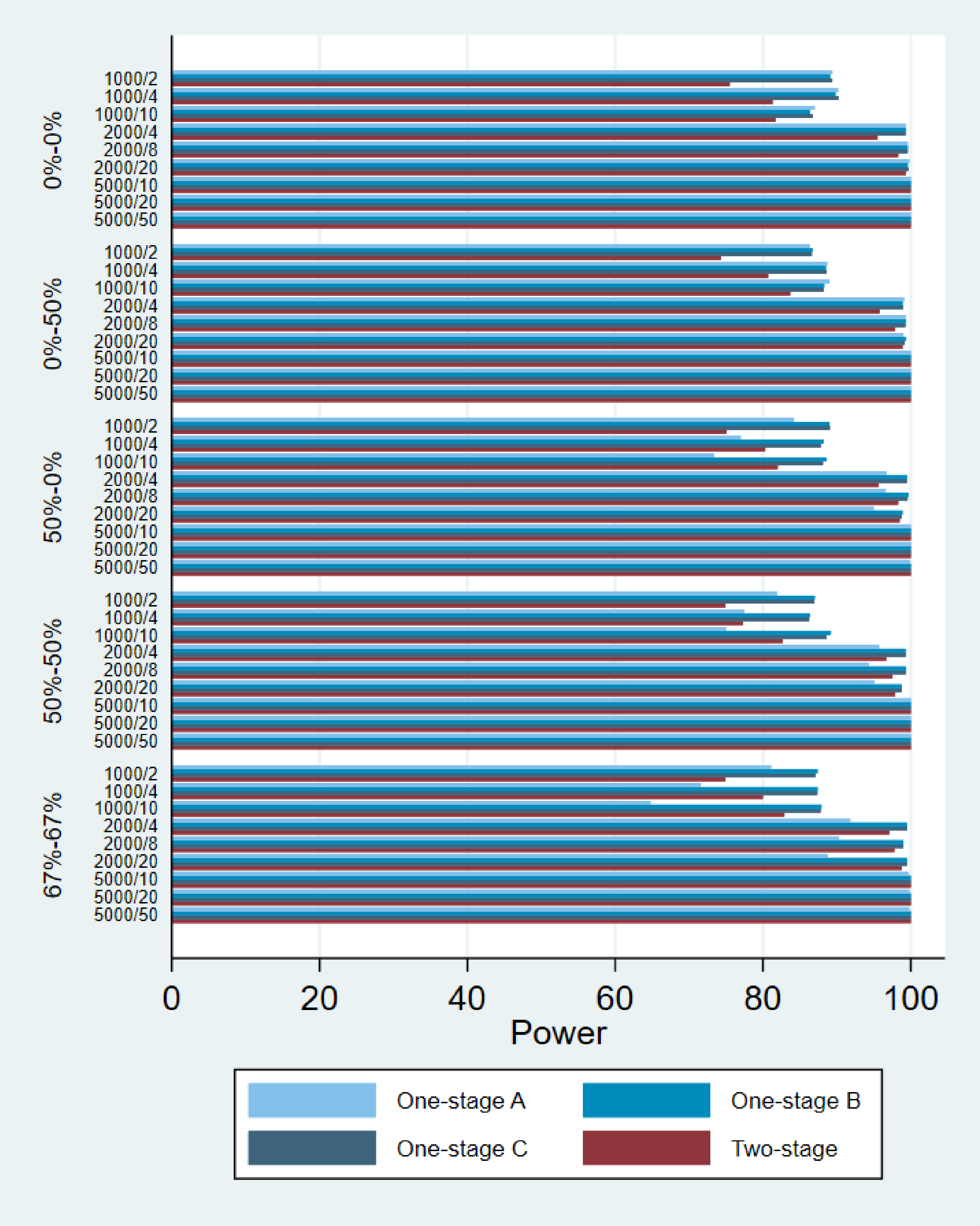

Simulation setting 5

Figure A29: Coverage and Power (%), plotted together [(coverage+power)/2]

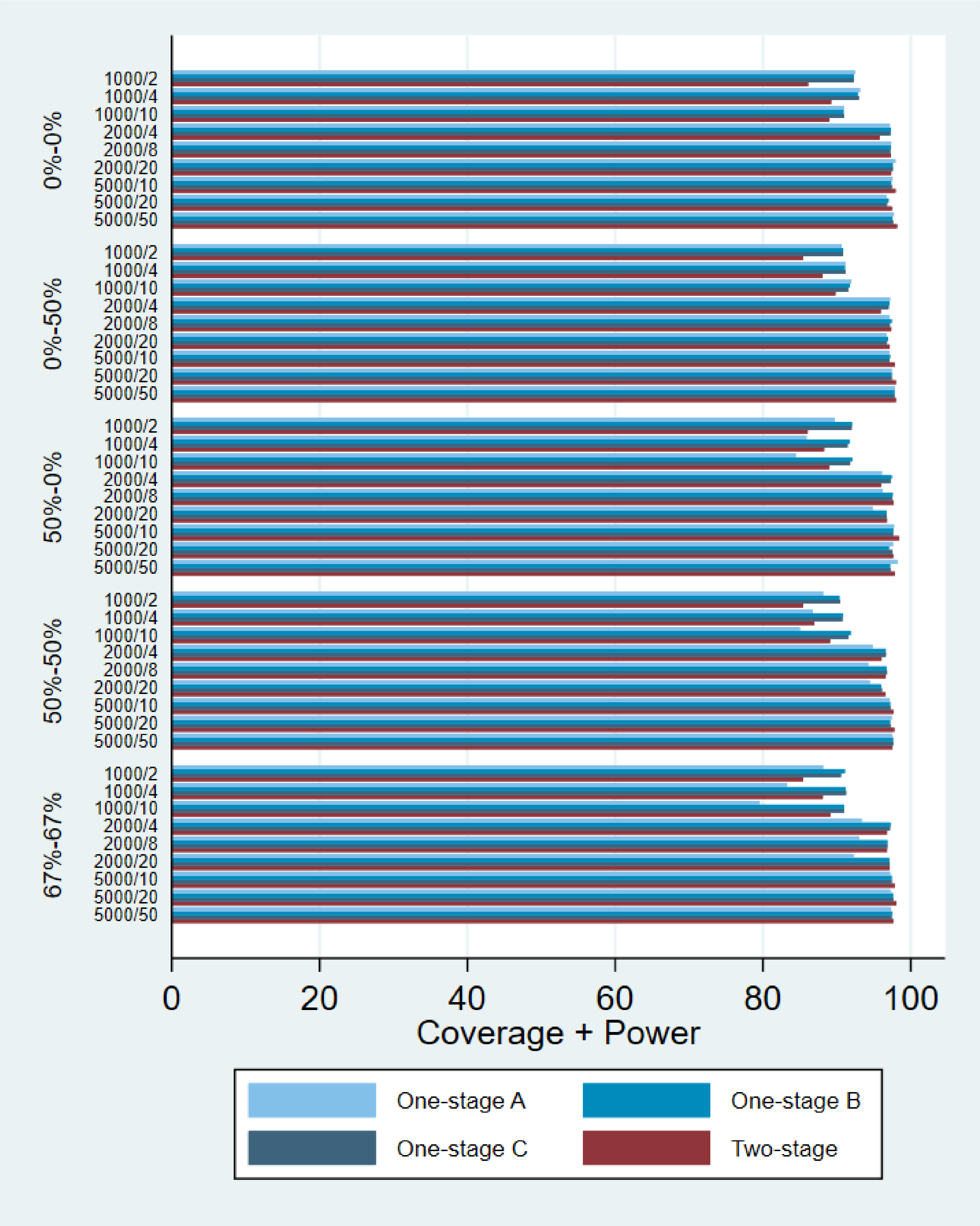

Simulation setting 5

Figure A30: Model convergence (%)

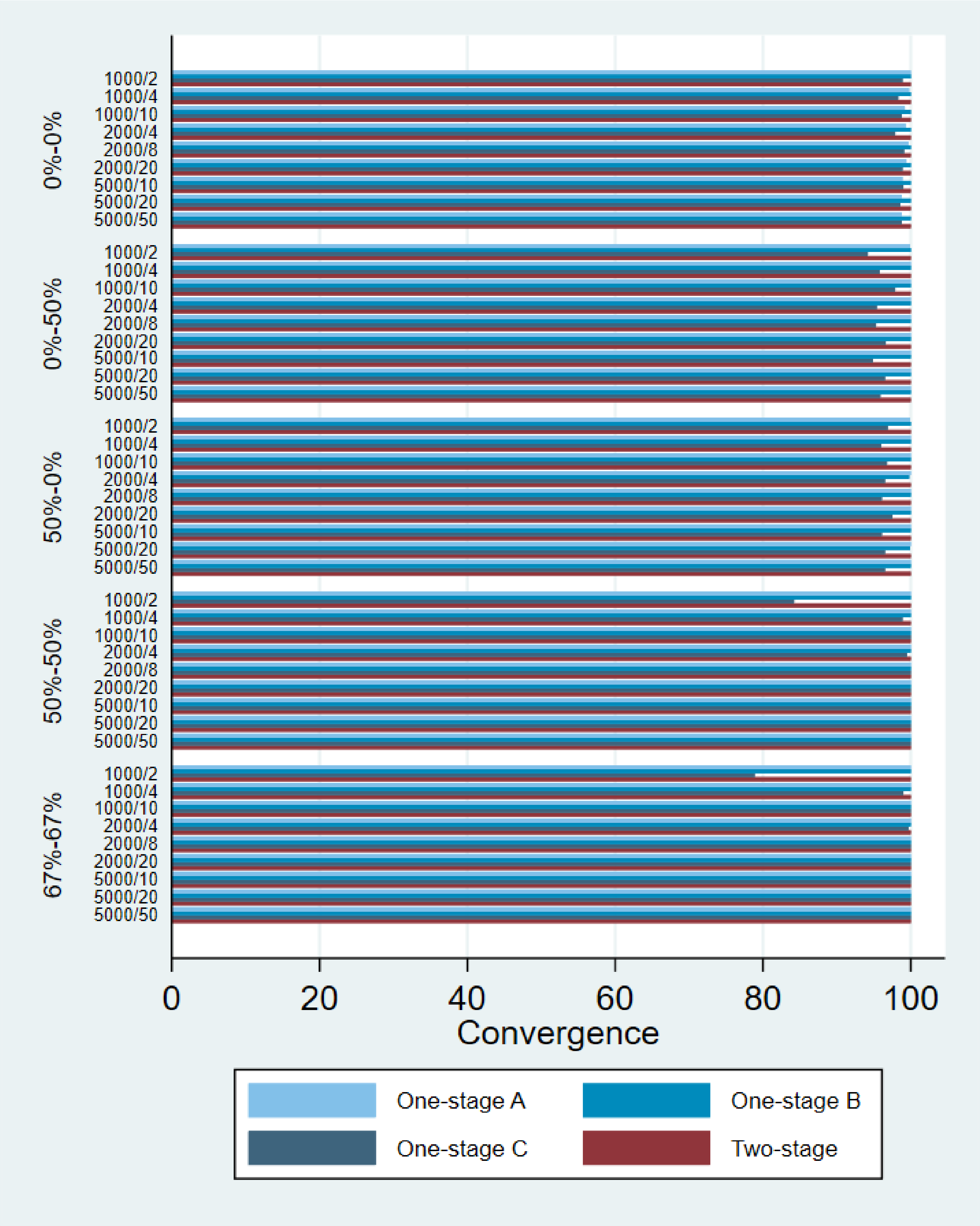

Simulation setting 6

Figure A31: Mean Bias

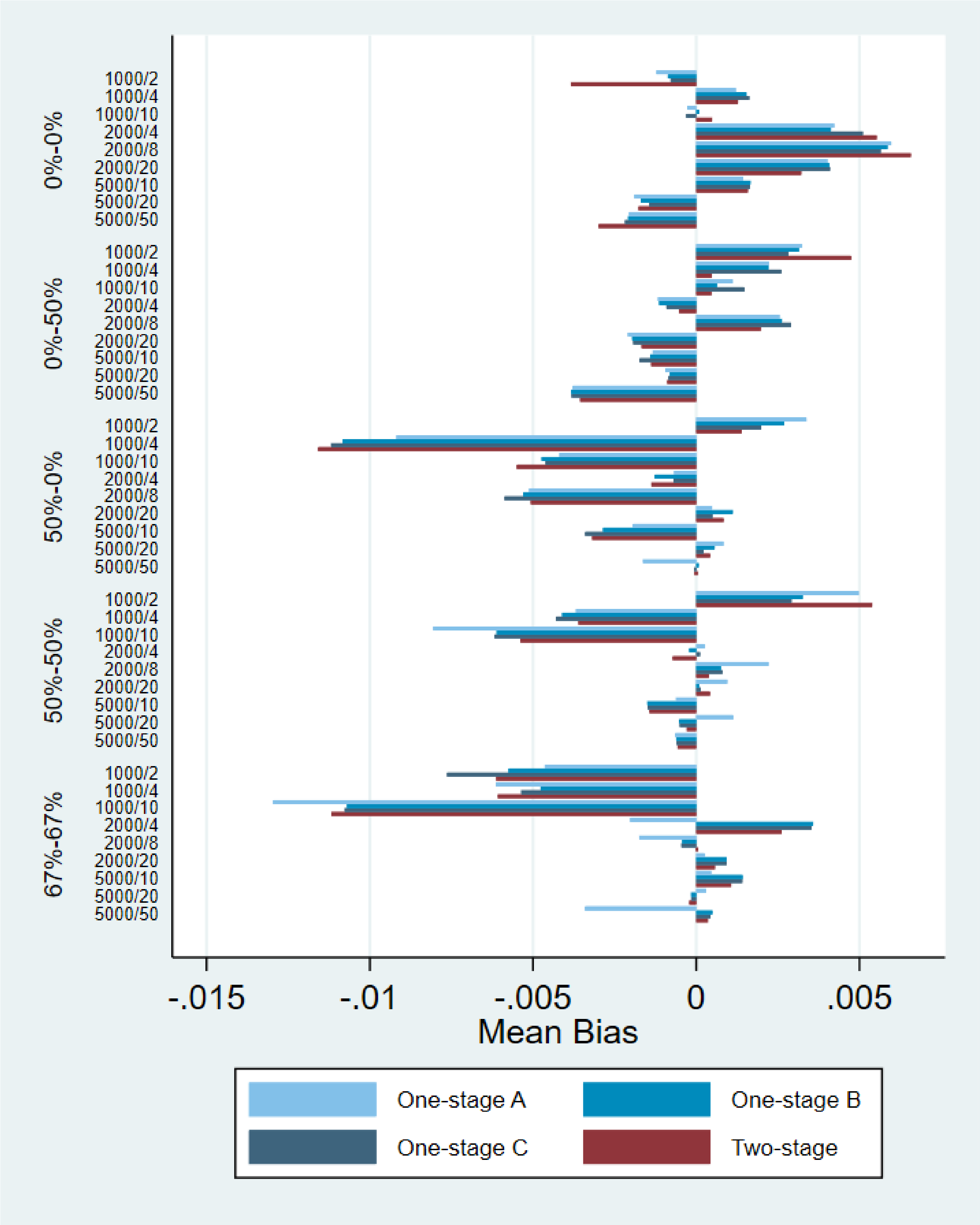

Simulation setting 6

Figure A32: Mean Error

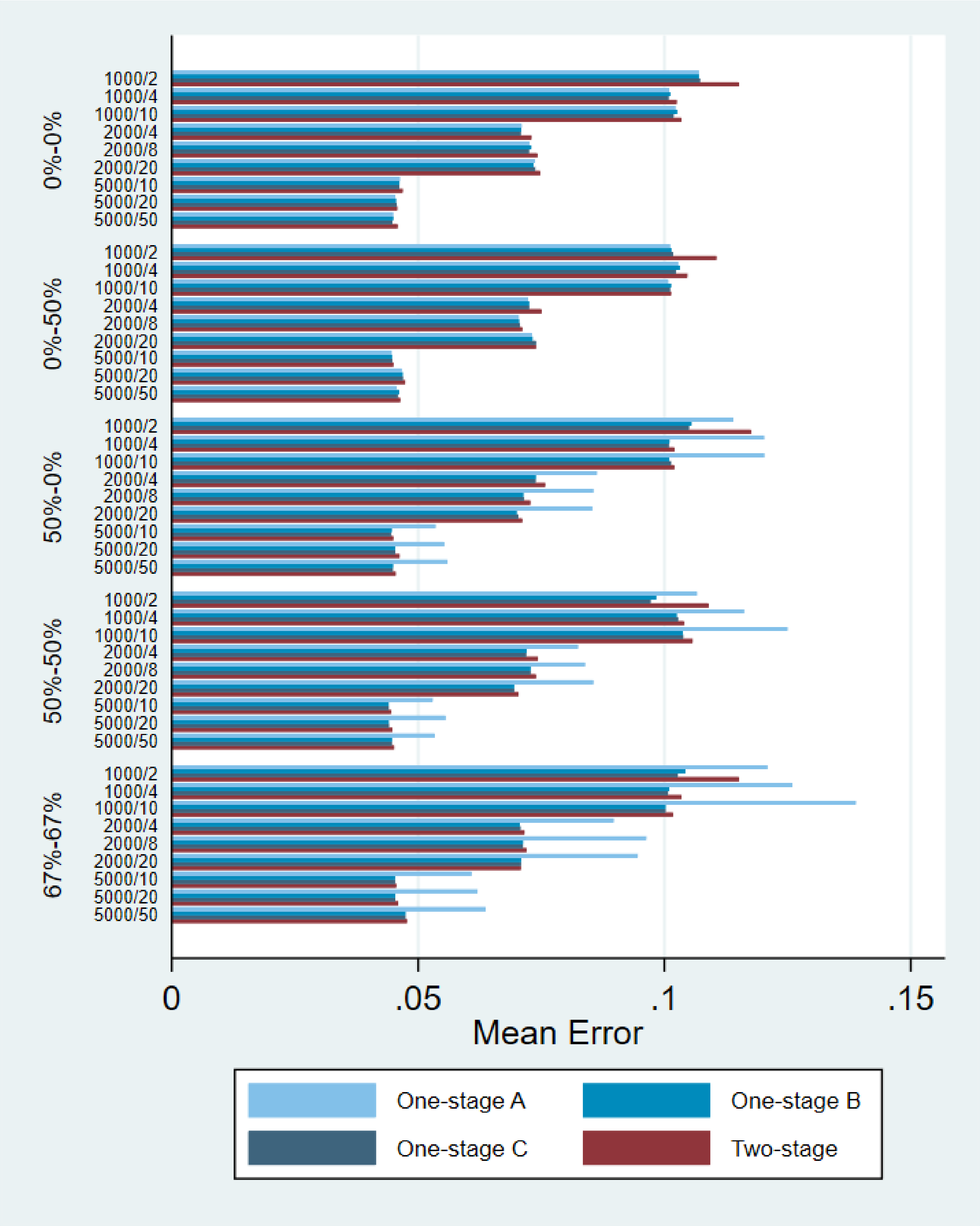

Simulation setting 6

Figure A33: Coverage probability (%), against 95% nominal line

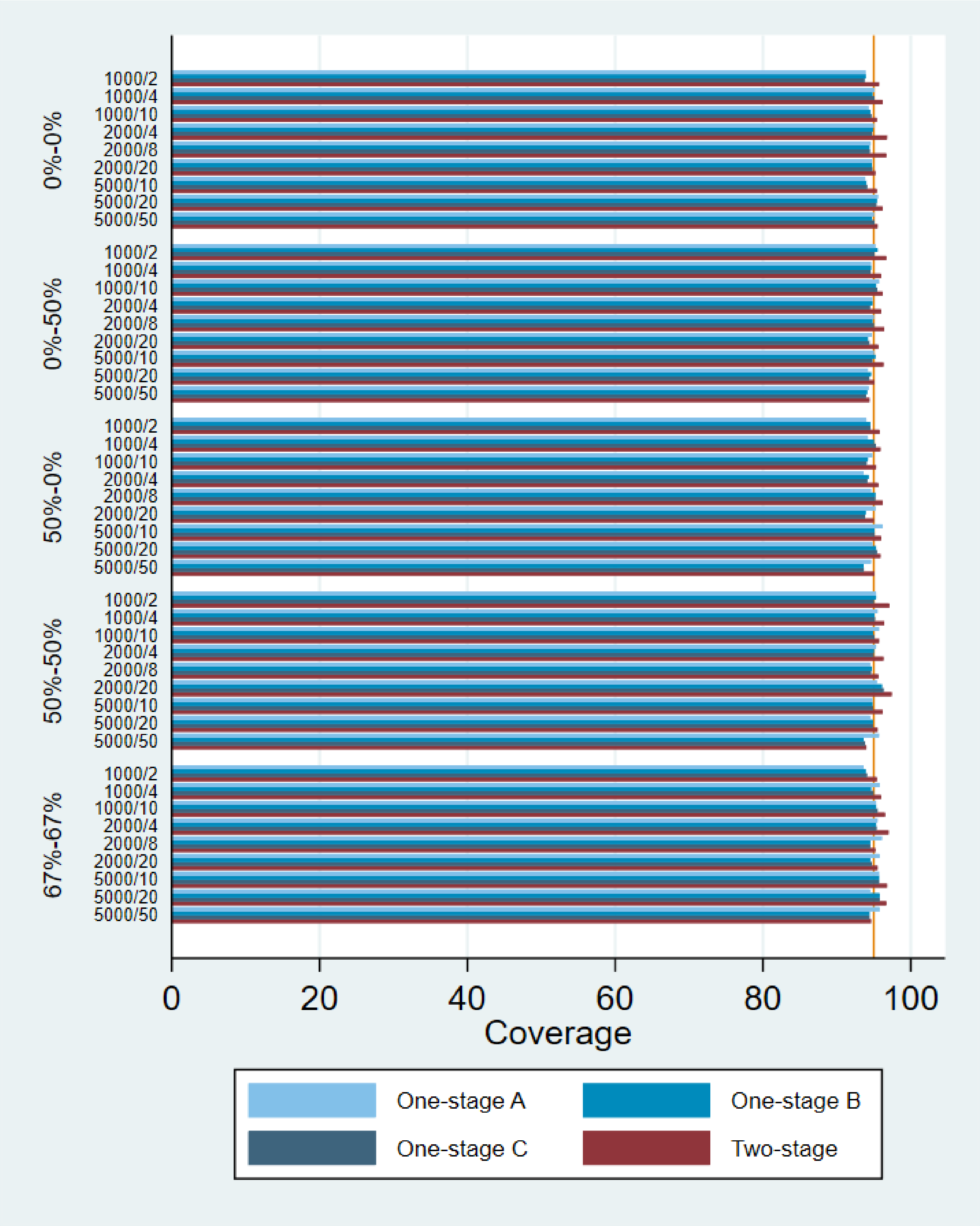

Simulation setting 6

Figure A34: Power probability (%)

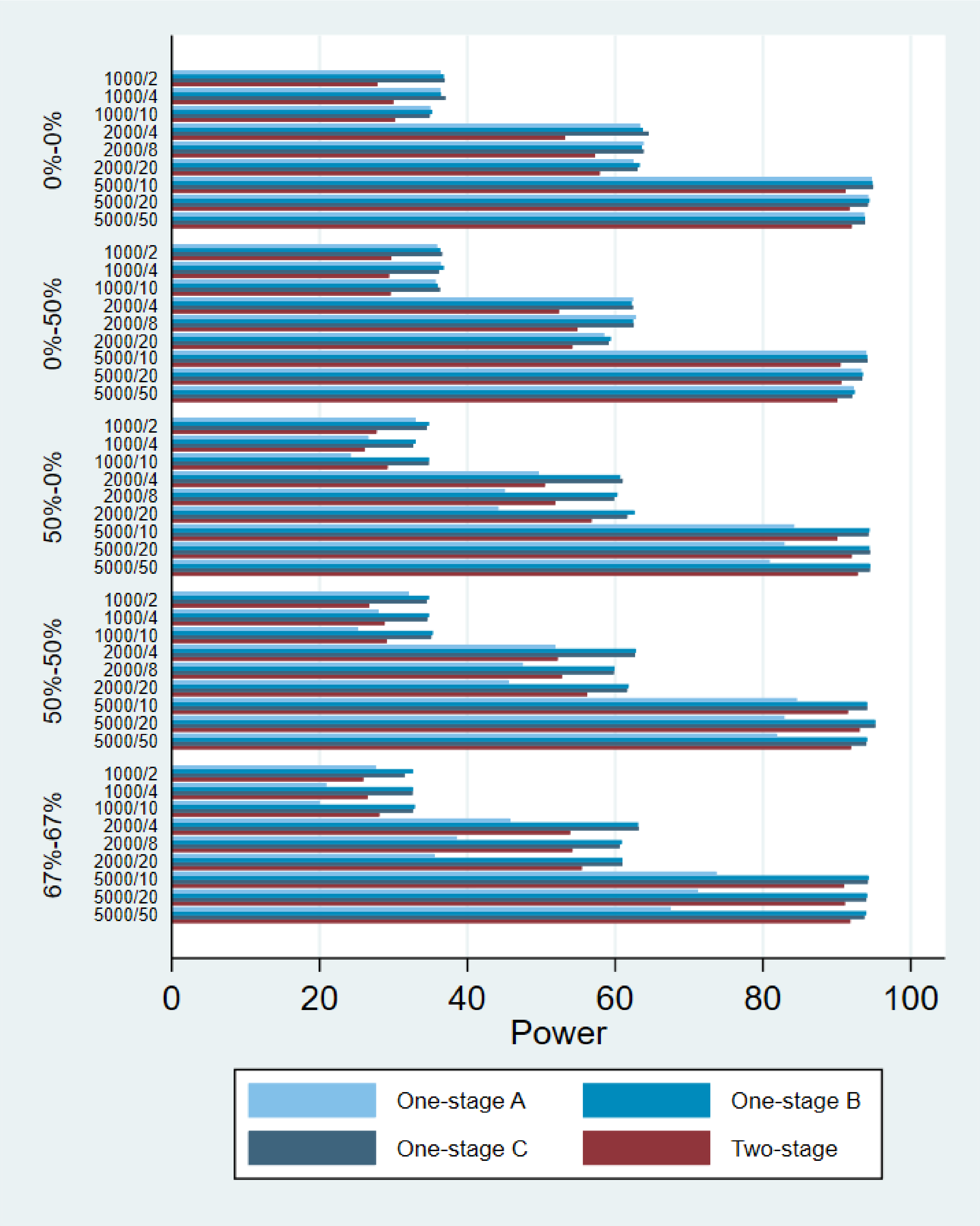

Simulation setting 6

Figure A35: Coverage and Power (%), plotted together [(coverage+power)/2]

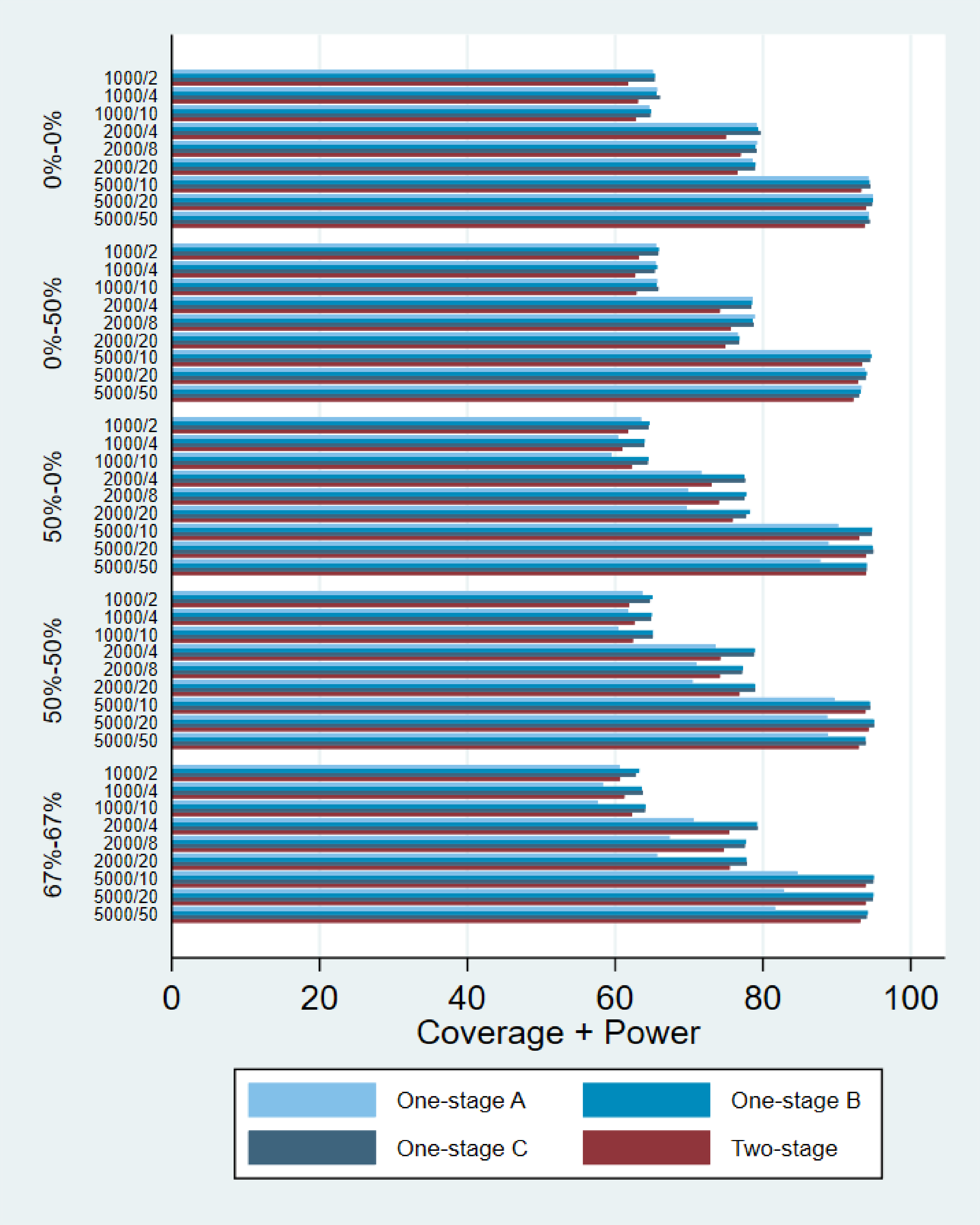

Simulation setting 6

Figure A36: Model convergence (%)

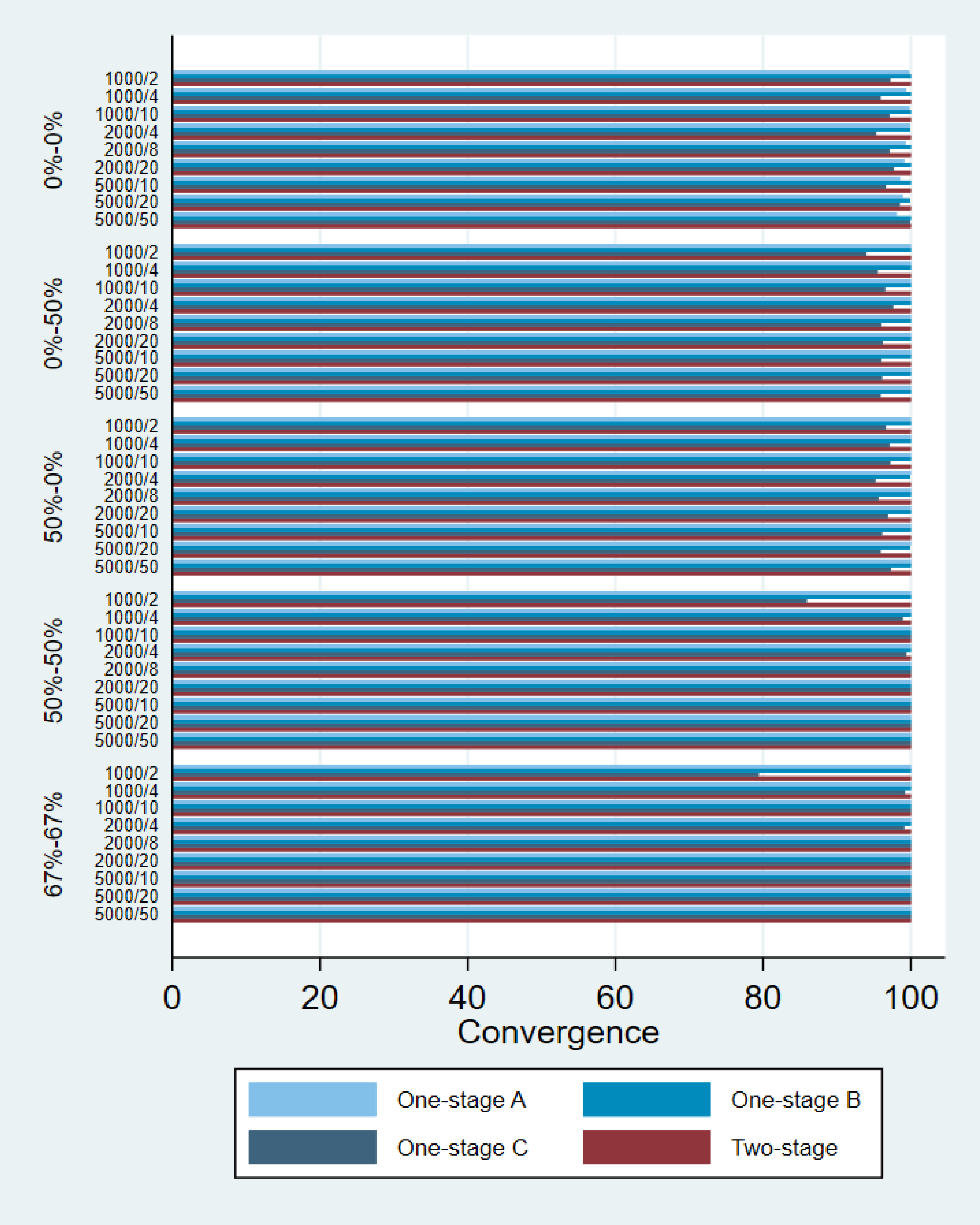

Simulation setting 7

Figure A37: Mean Bias

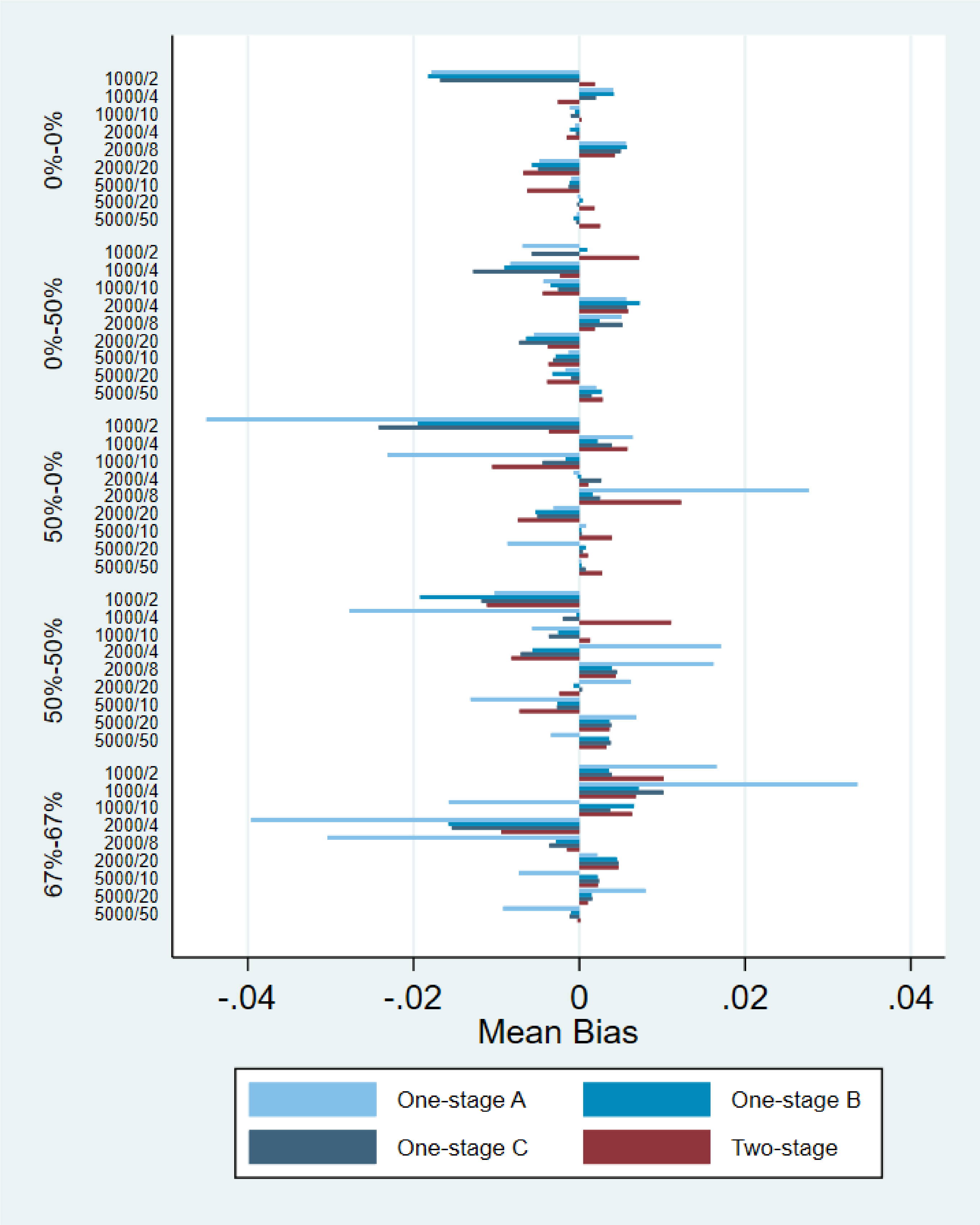

Simulation setting 7

Figure A38: Mean Error

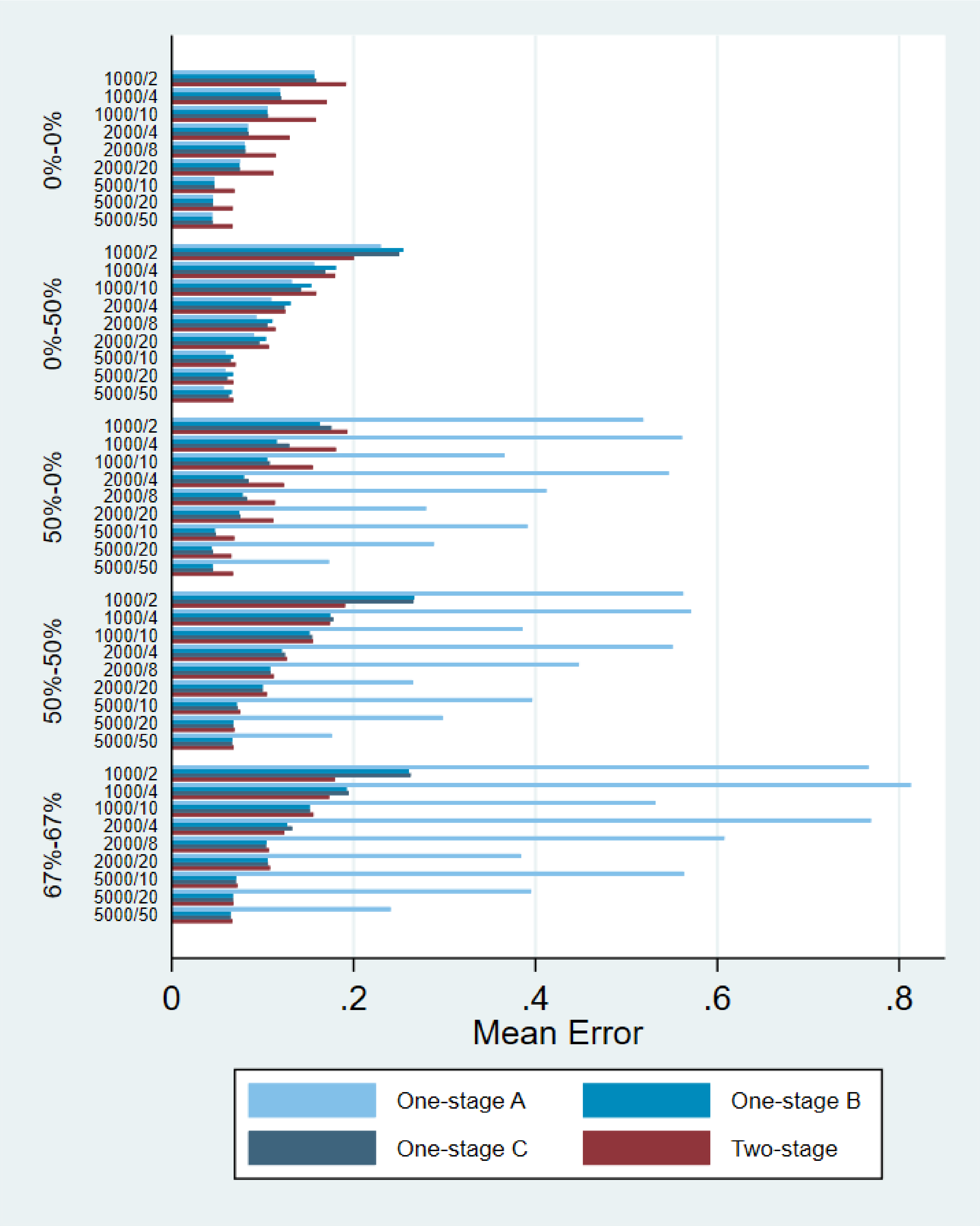

Simulation setting 7

Figure A39: Coverage probability (%), against 95% nominal line

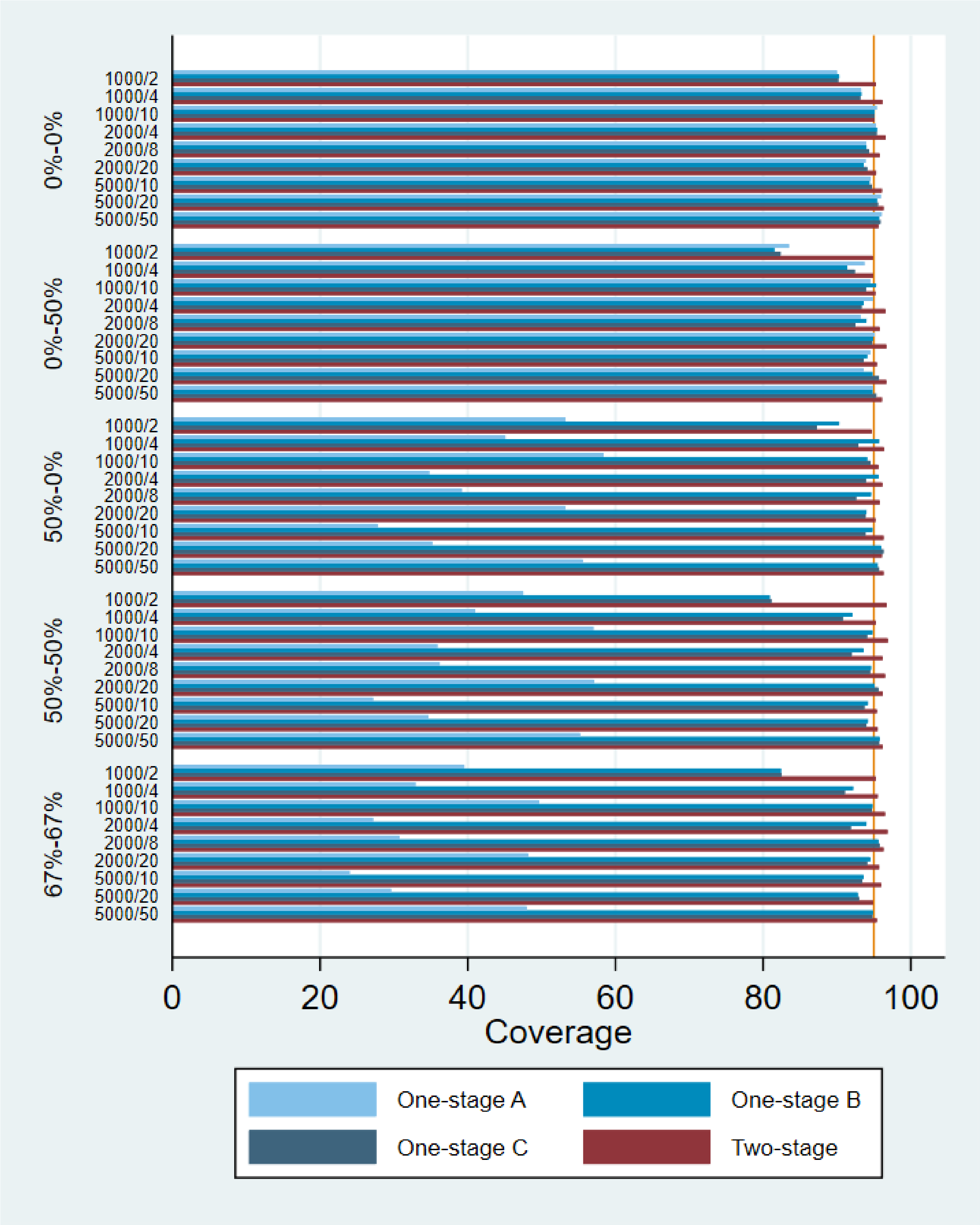

Simulation setting 7

Figure A40: Power probability (%)

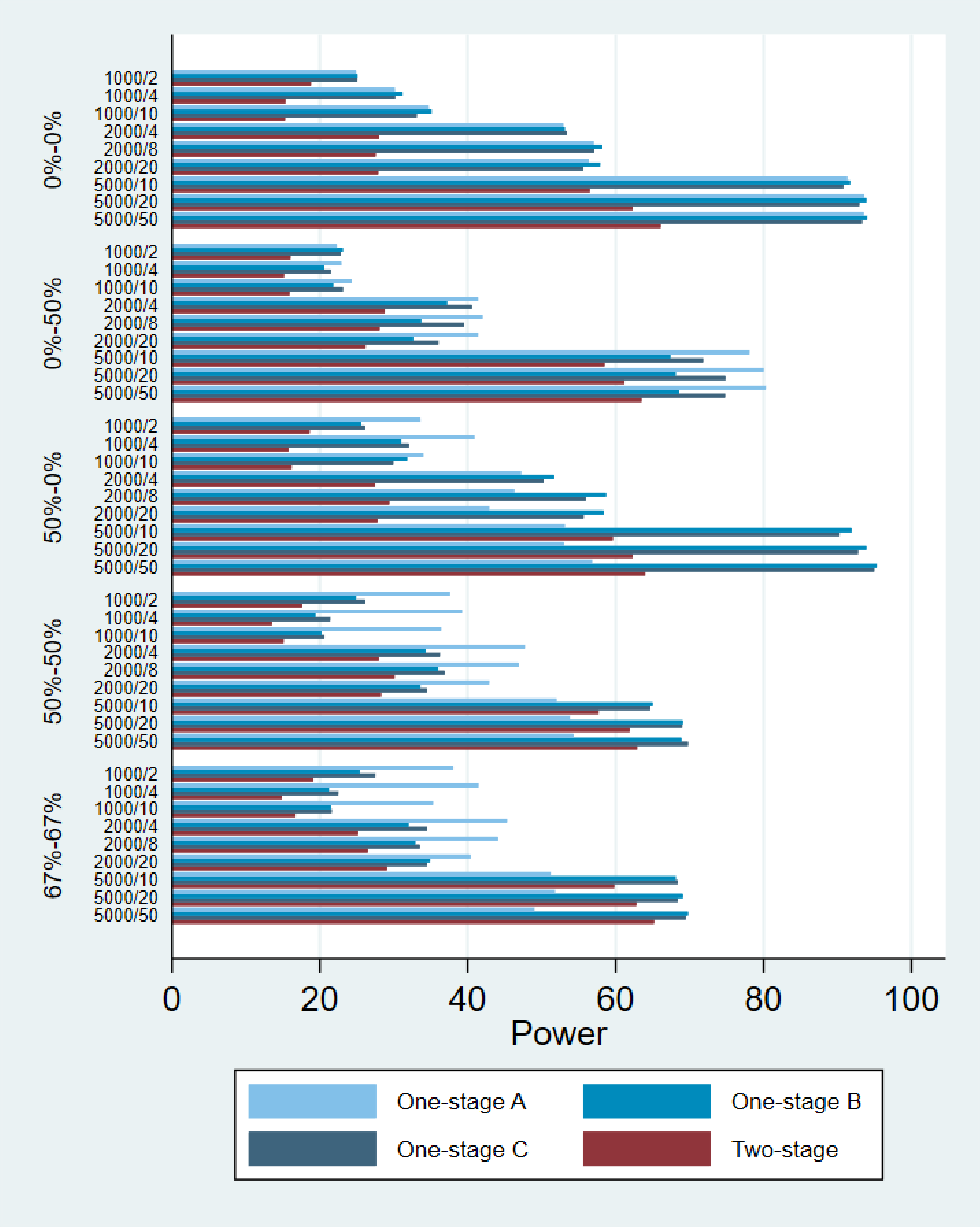

Simulation setting 7

Figure A41: Coverage and Power (%), plotted together [(coverage+power)/2]

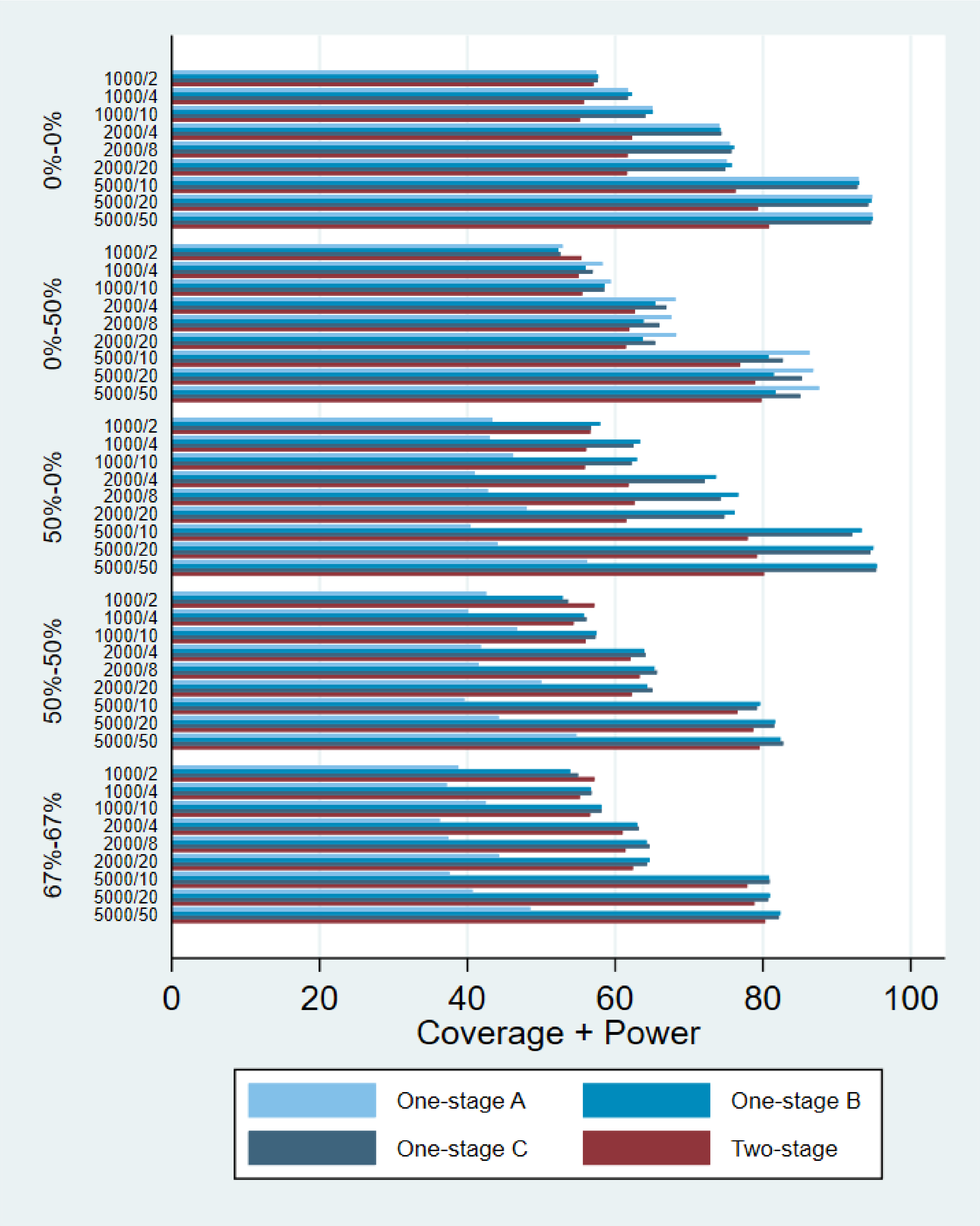

Simulation setting 7

Figure A42: Model convergence (%)

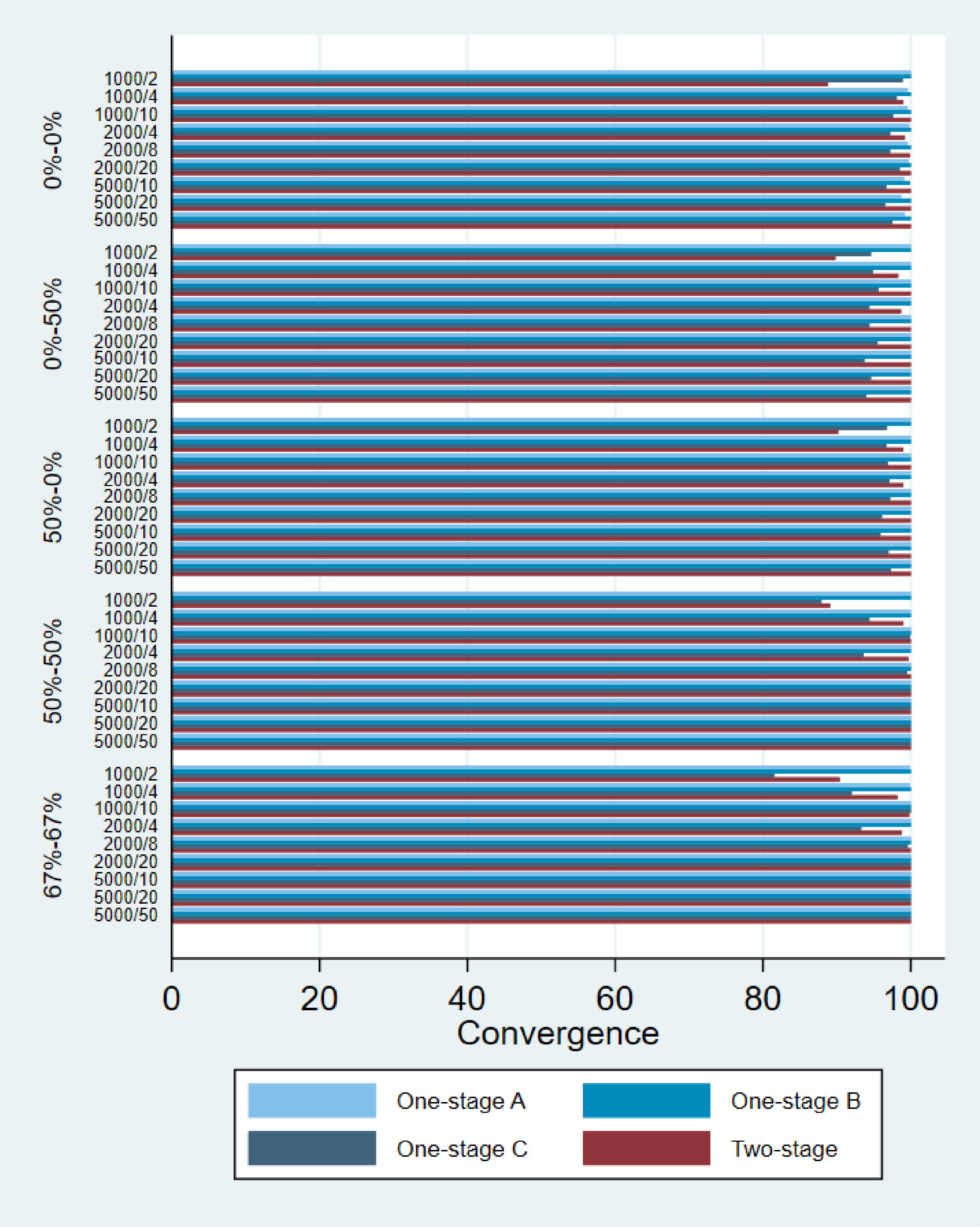

Simulation setting 8

Figure A43: Mean Bias, models A-D

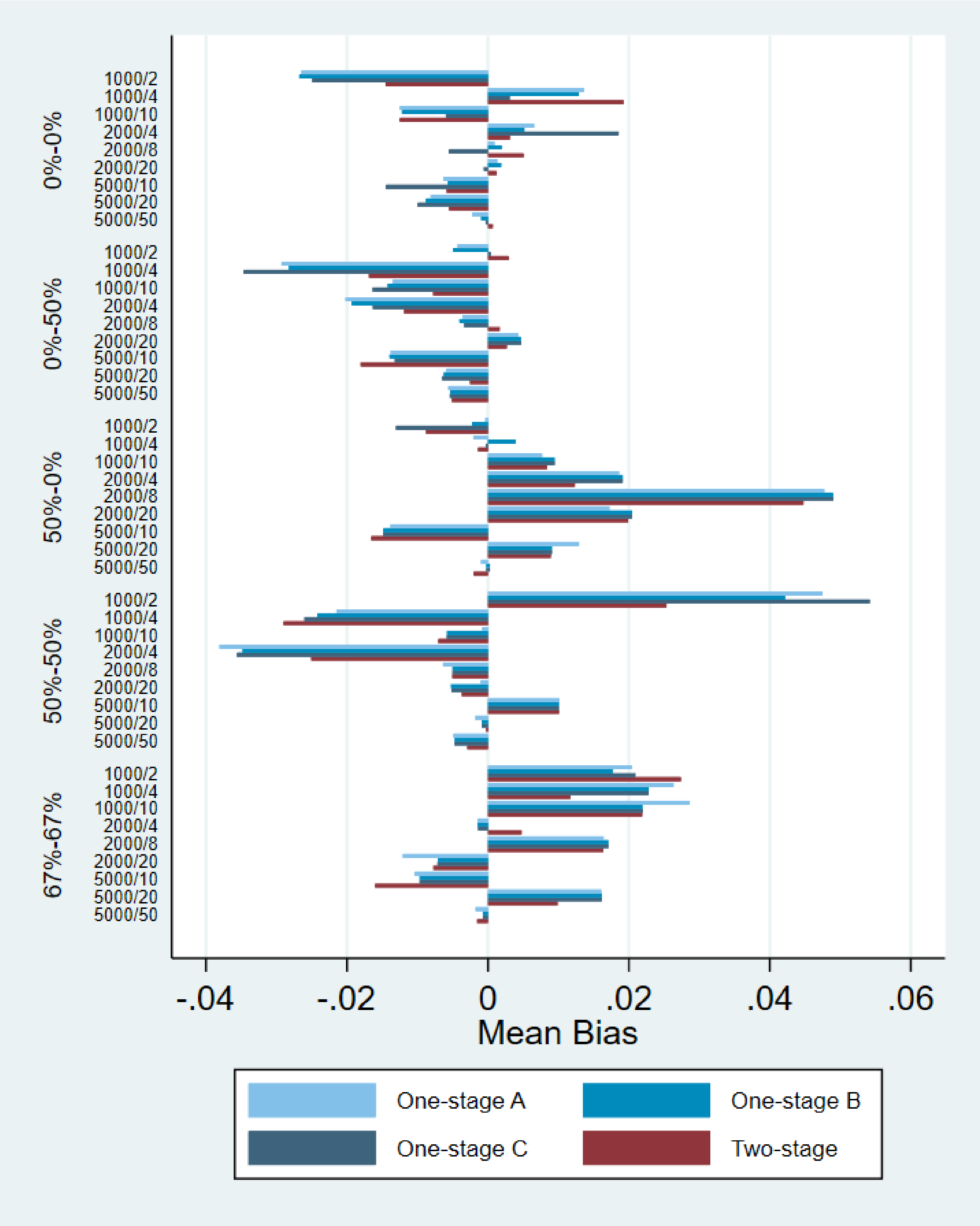

Simulation setting 8

Figure A44: Mean Bias, models D & E-G

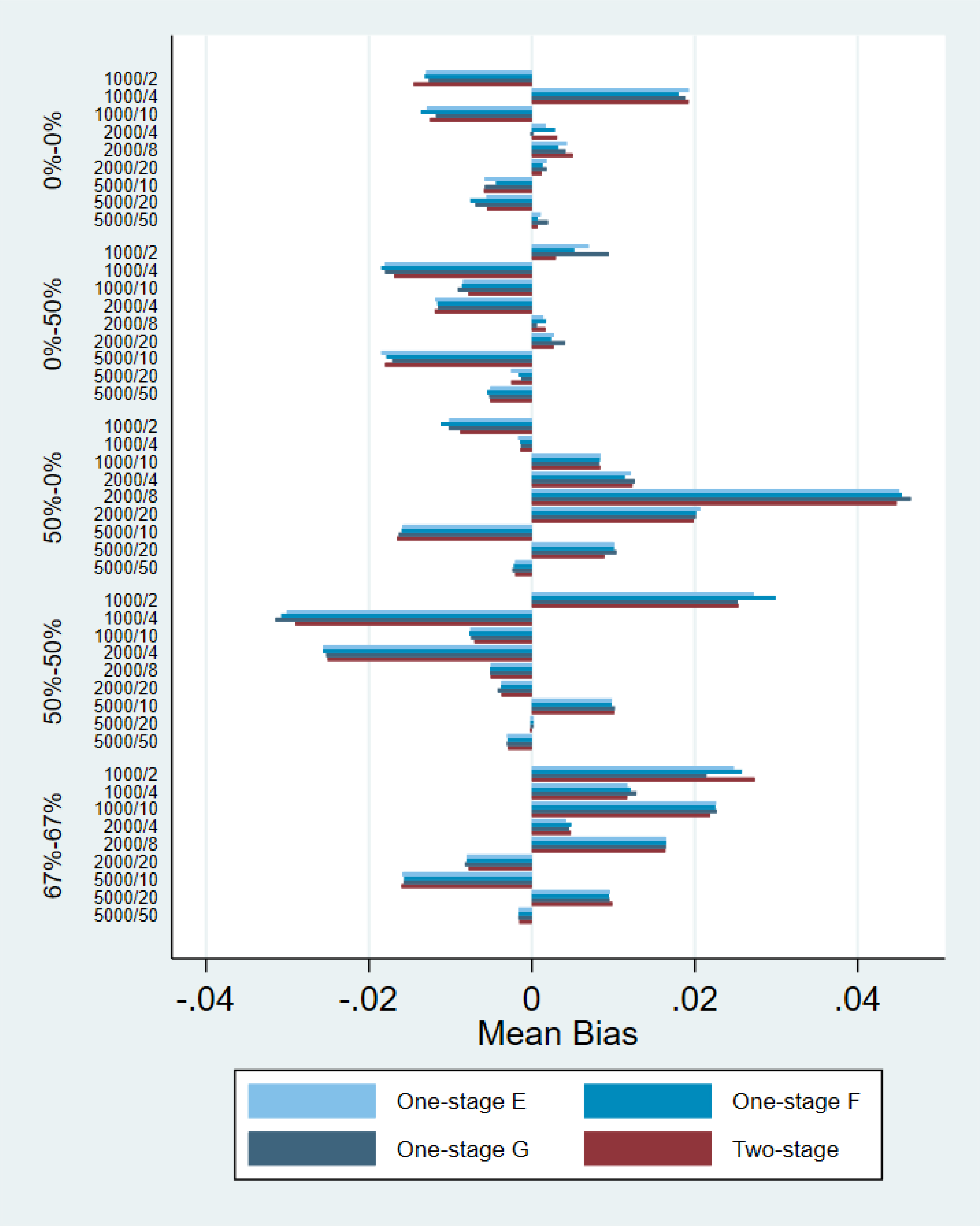

Simulation setting 8

Figure A45: Mean Error, models A-D

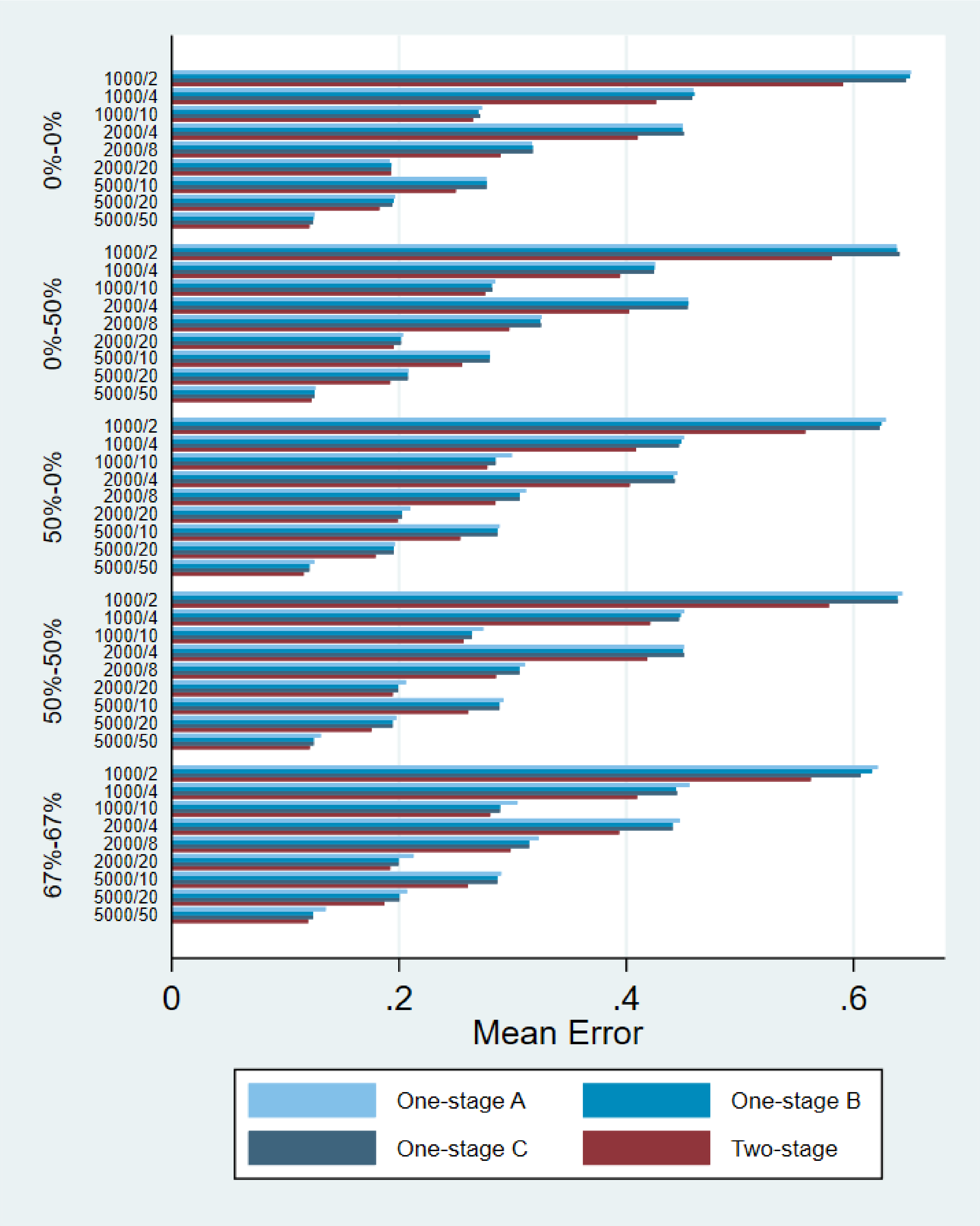

Simulation setting 8

Figure A46: Mean Error, models D & E-G

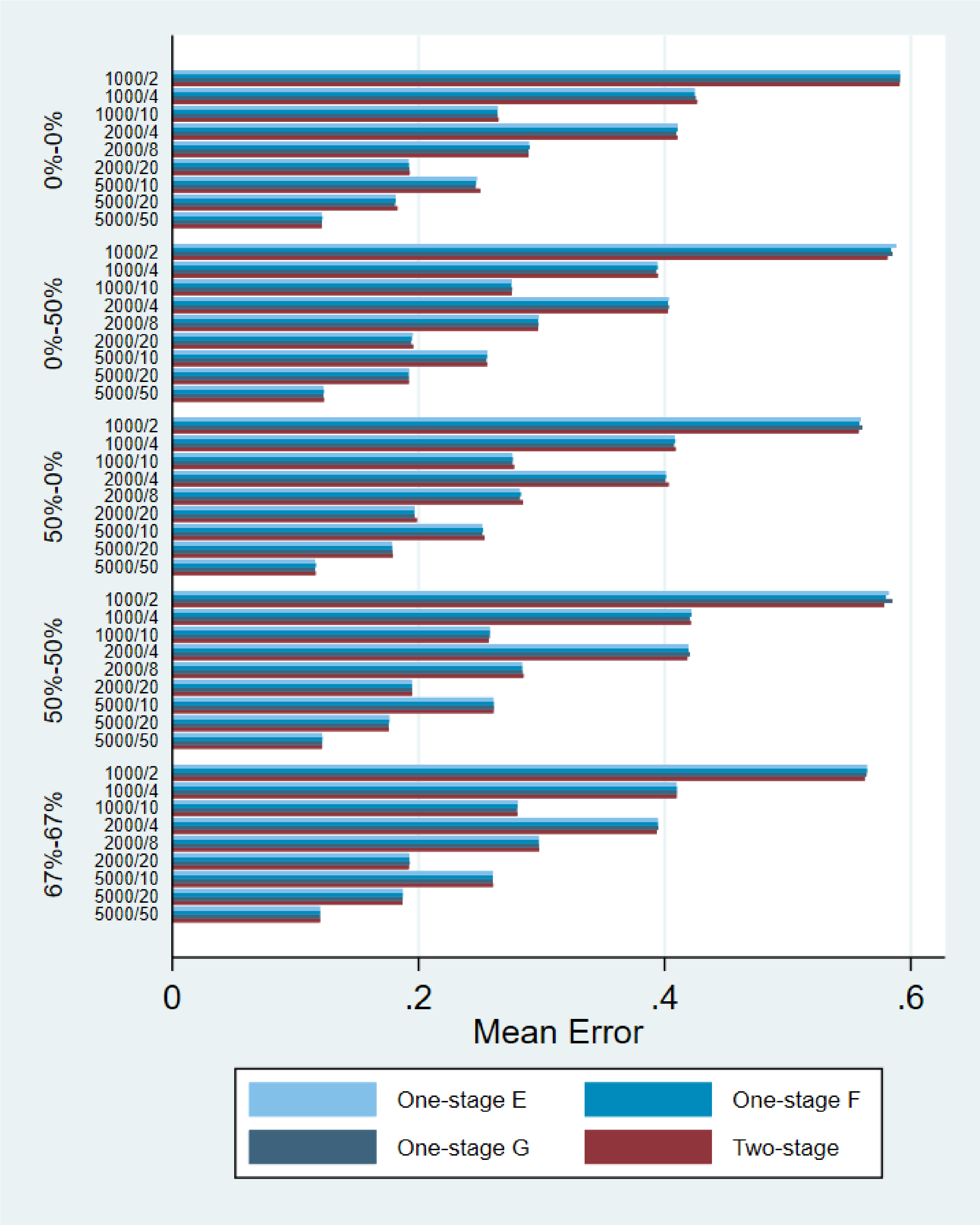

Simulation setting 8

Figure A47: Coverage probability (%), against 95% nominal line, models A-D

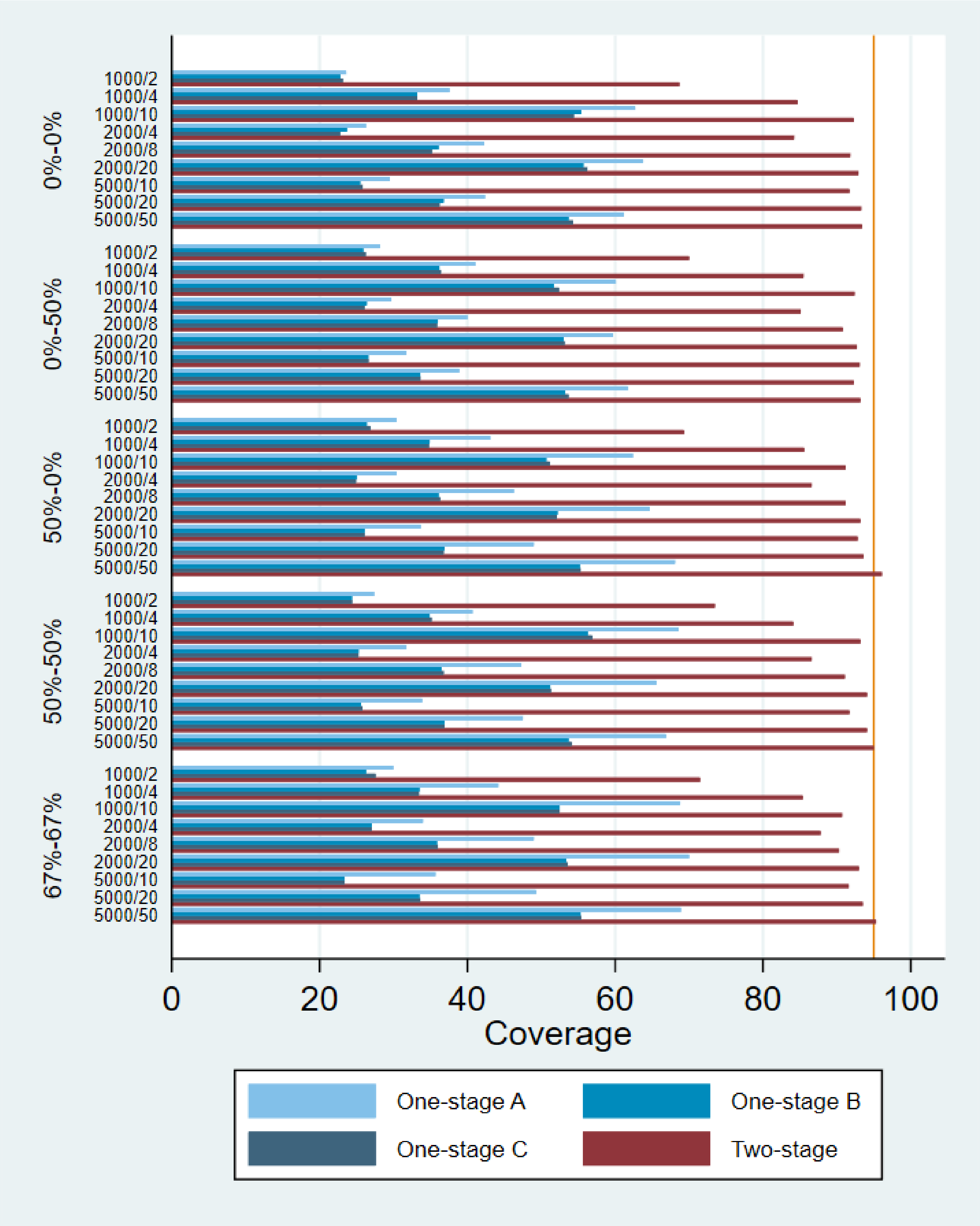

Simulation setting 8

Figure A48: Coverage probability (%), against 95% nominal line, models D & E-G

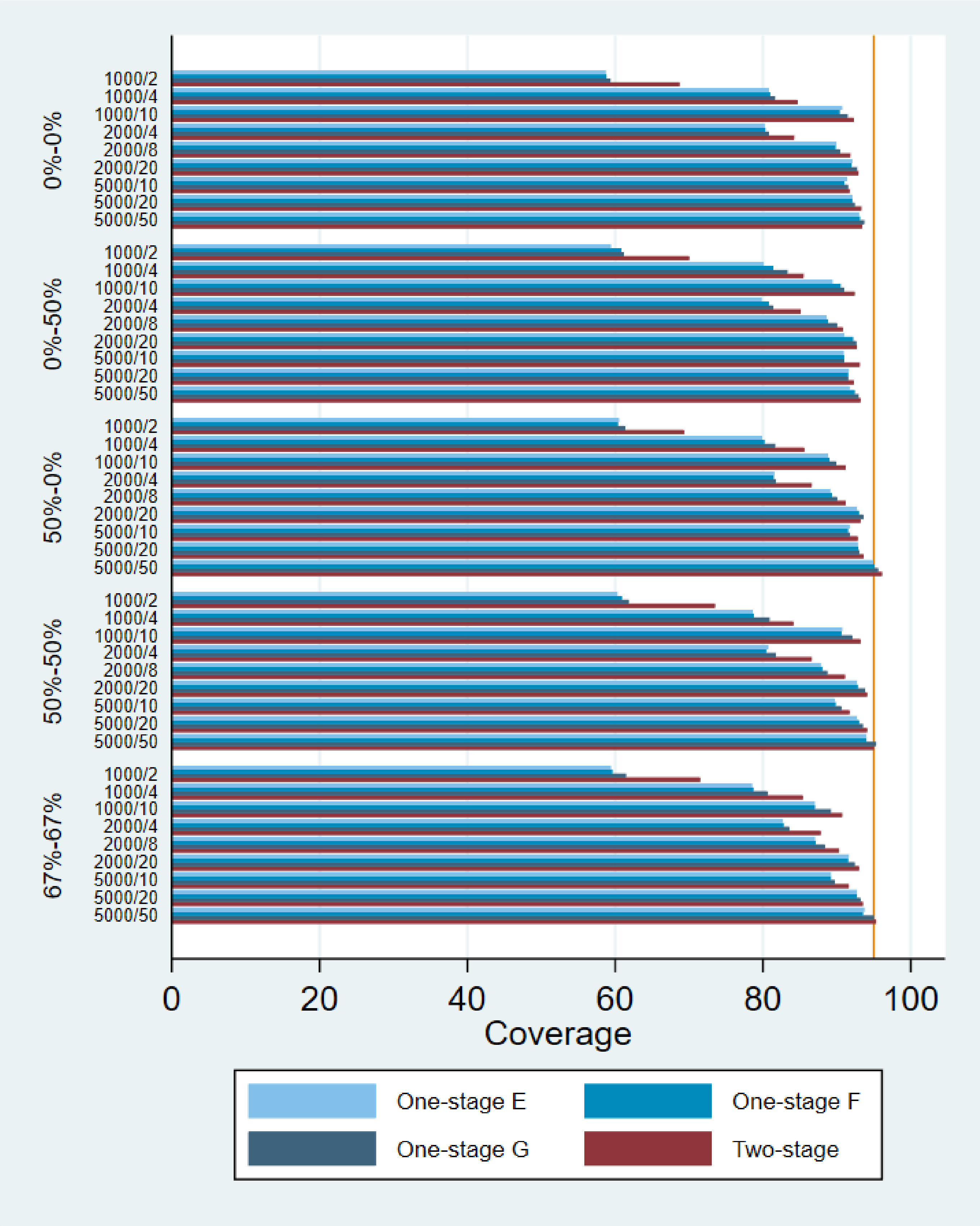

Simulation setting 8

Figure A49: Power probability (%), models A-D

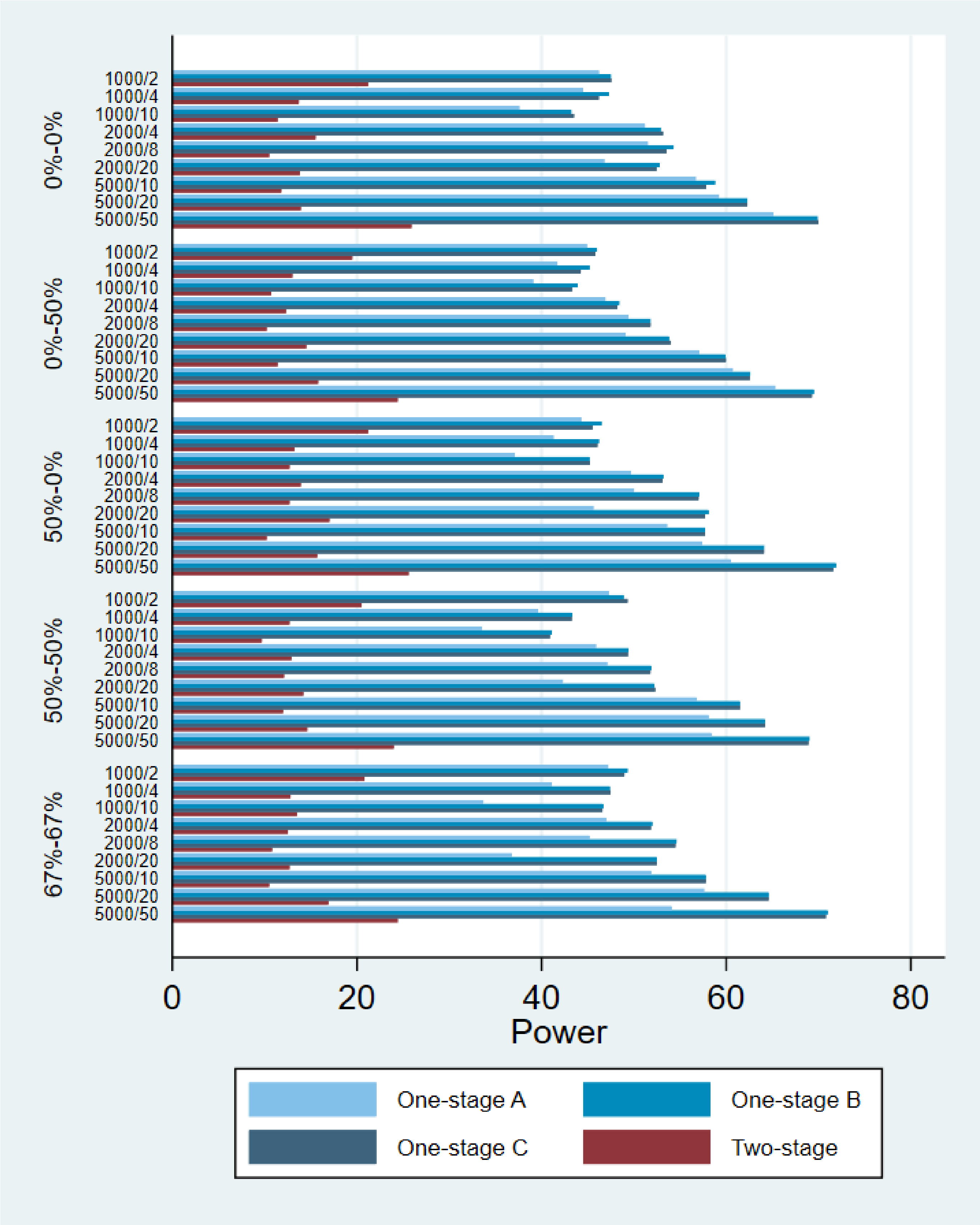

Simulation setting 8

Figure A50: Power probability (%), models D & E-G

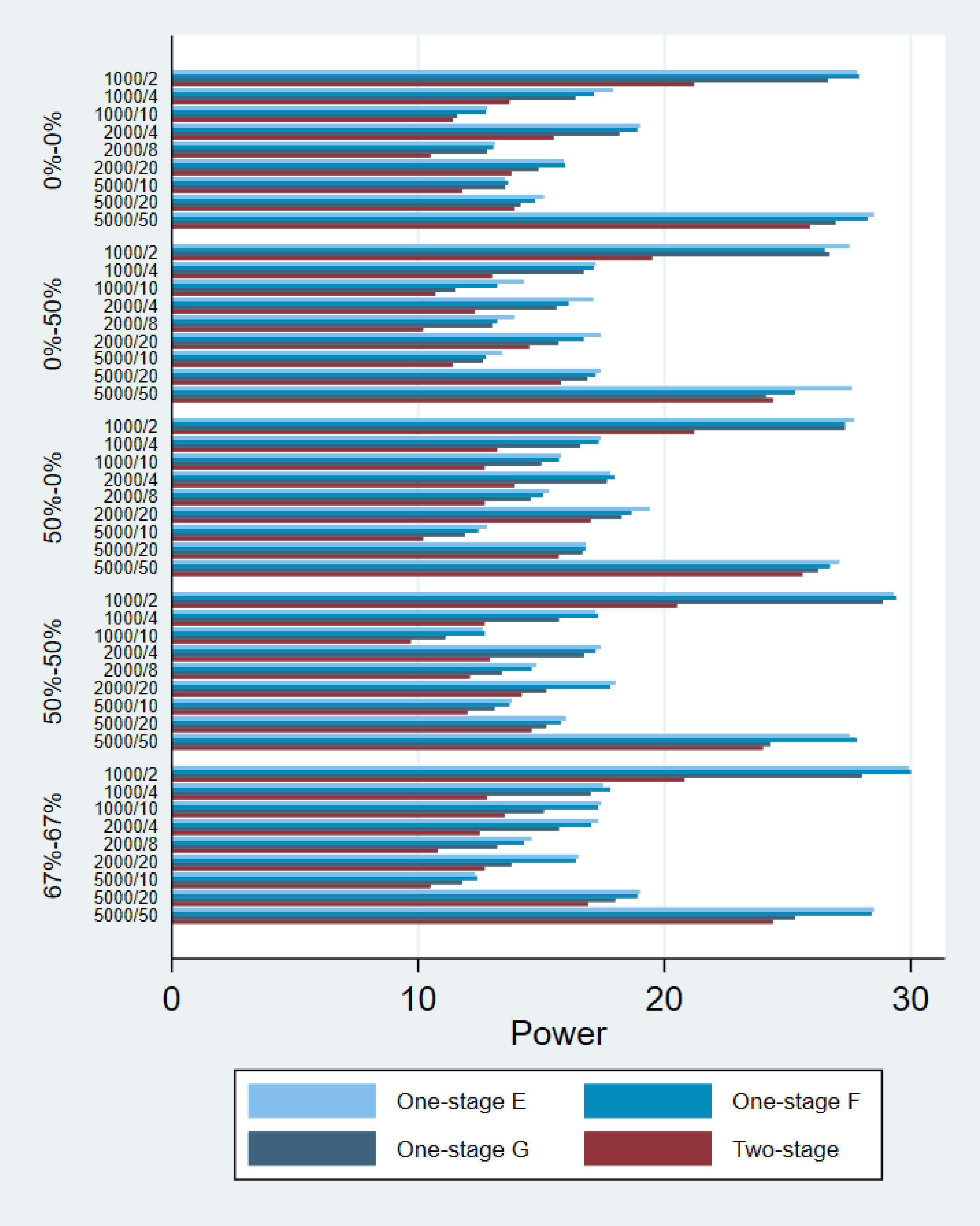

Simulation setting 8

Figure A51: Coverage and Power (%), plotted together [(coverage+power)/2], models A-D

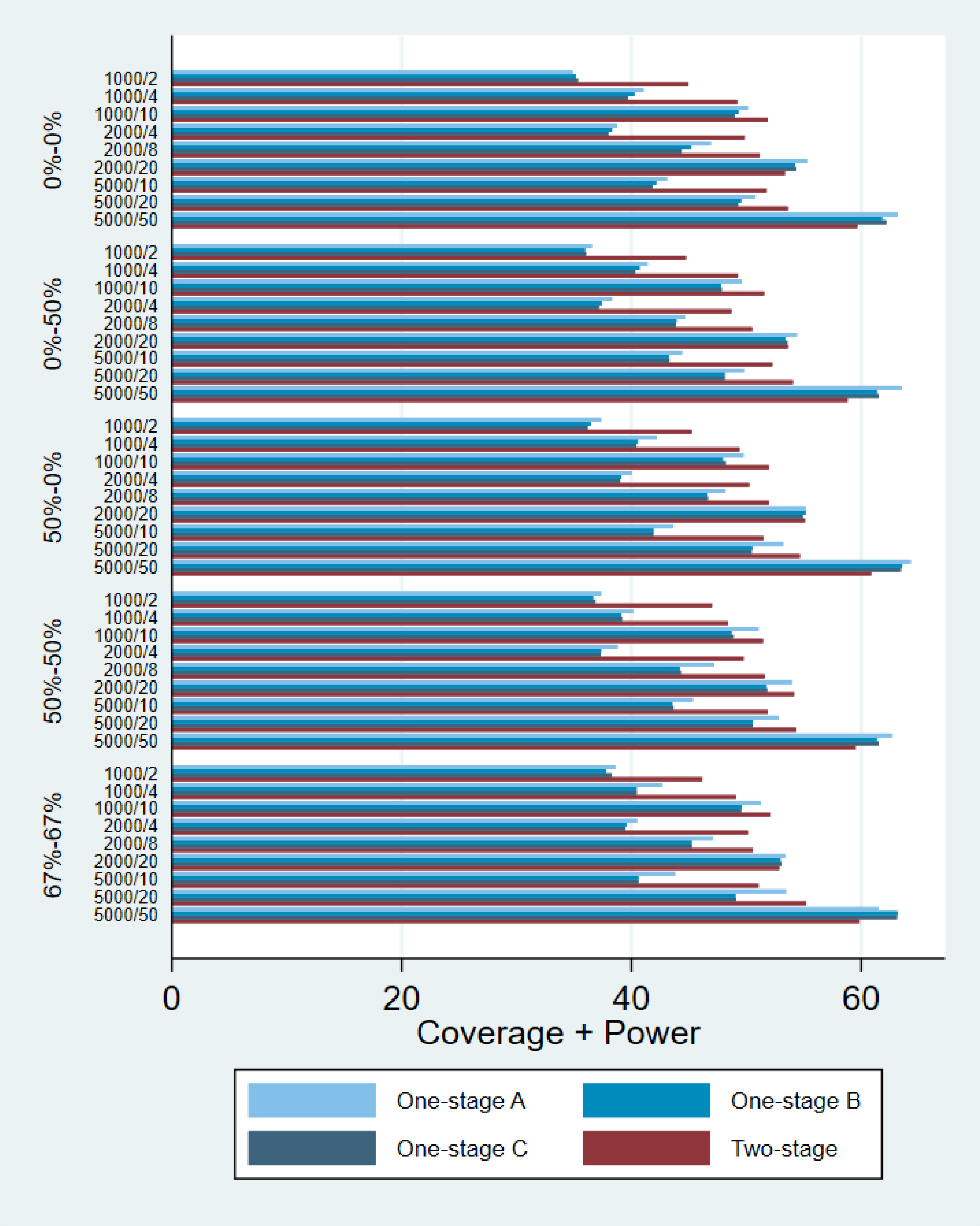

Simulation setting 8

Figure A52: Coverage and Power (%), plotted together [(coverage+power)/2], models D & E-G

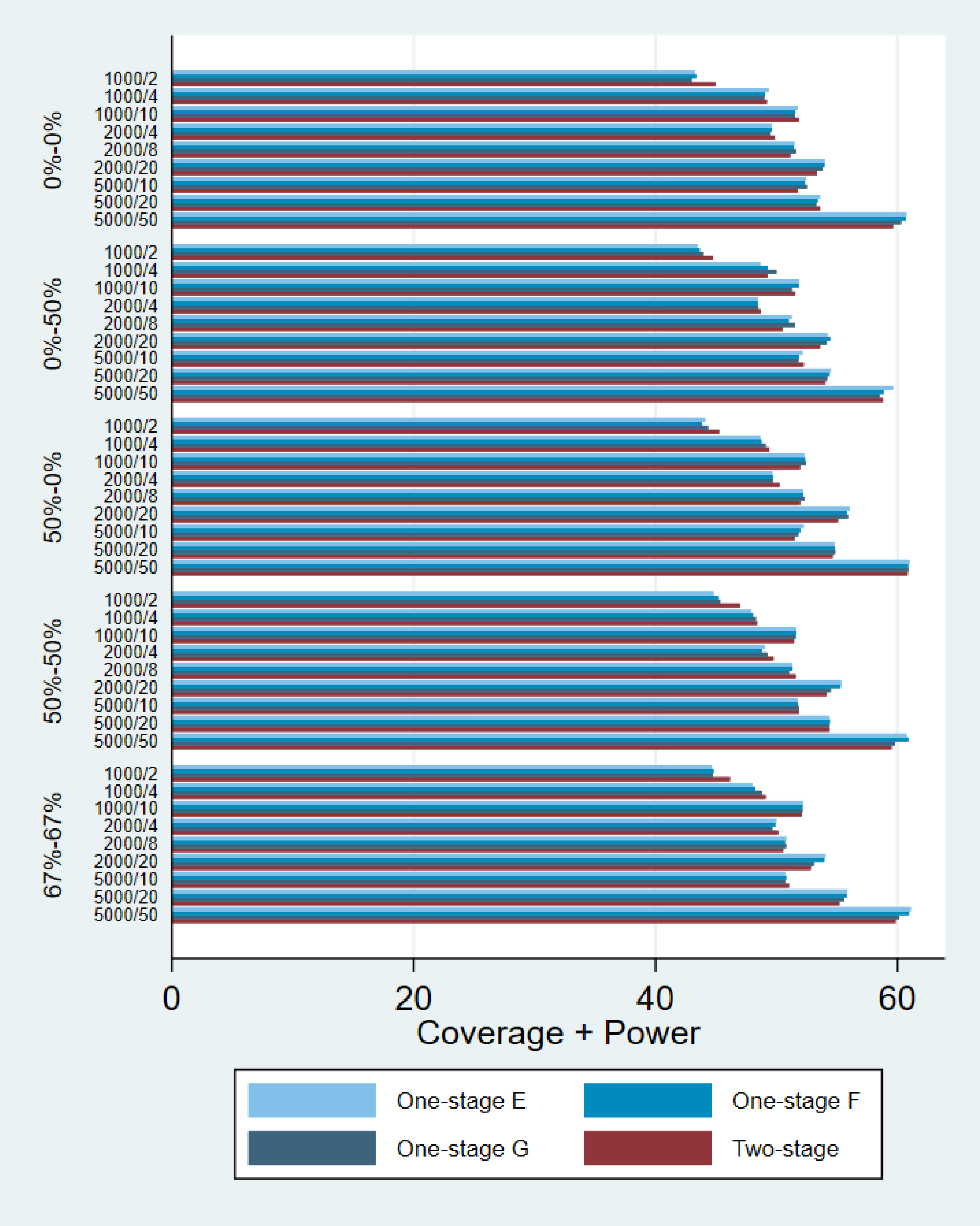

Simulation setting 8

Figure A53: Model convergence (%), models A-D

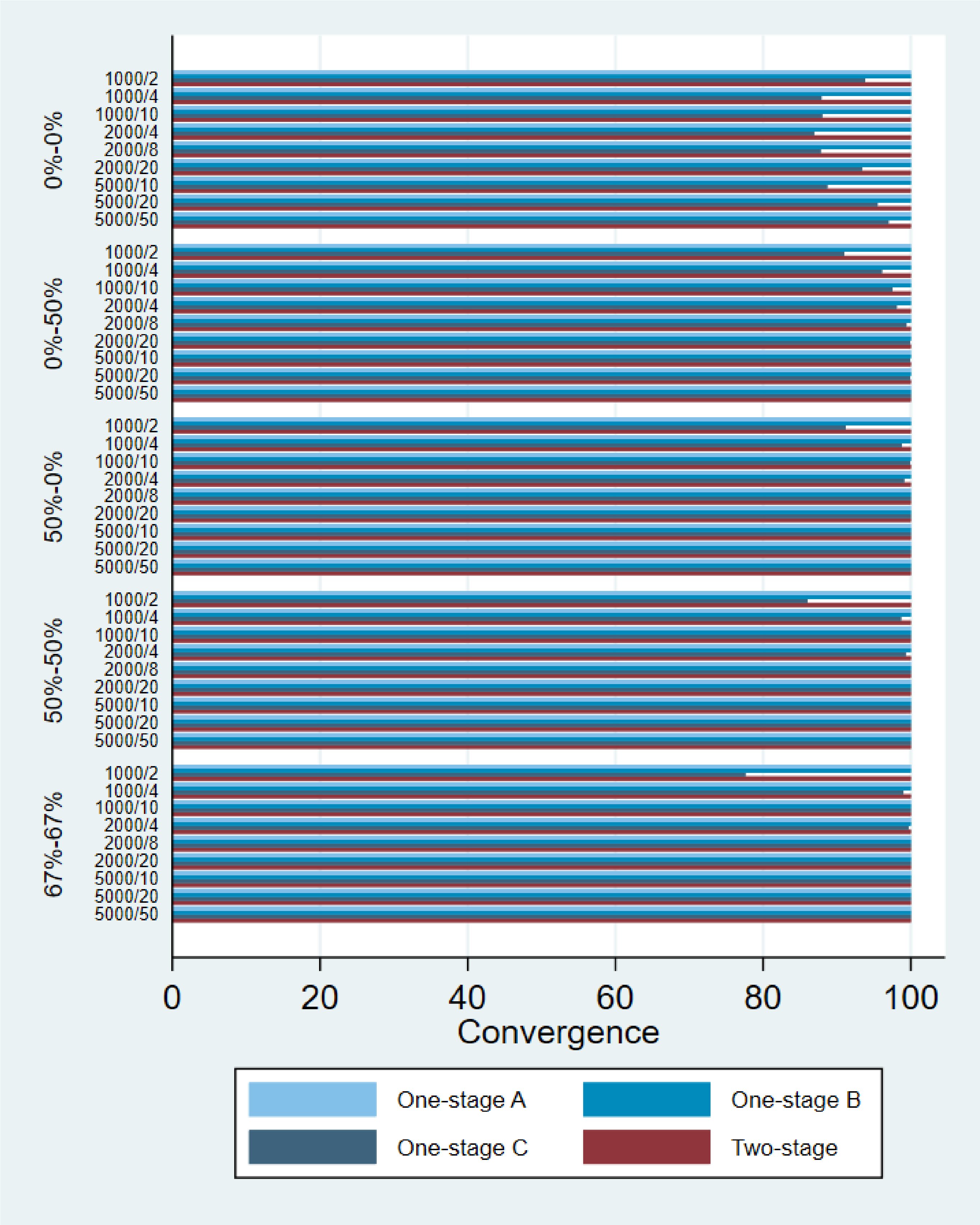

Simulation setting 8

Figure A54: Model convergence (%), models D & E-G

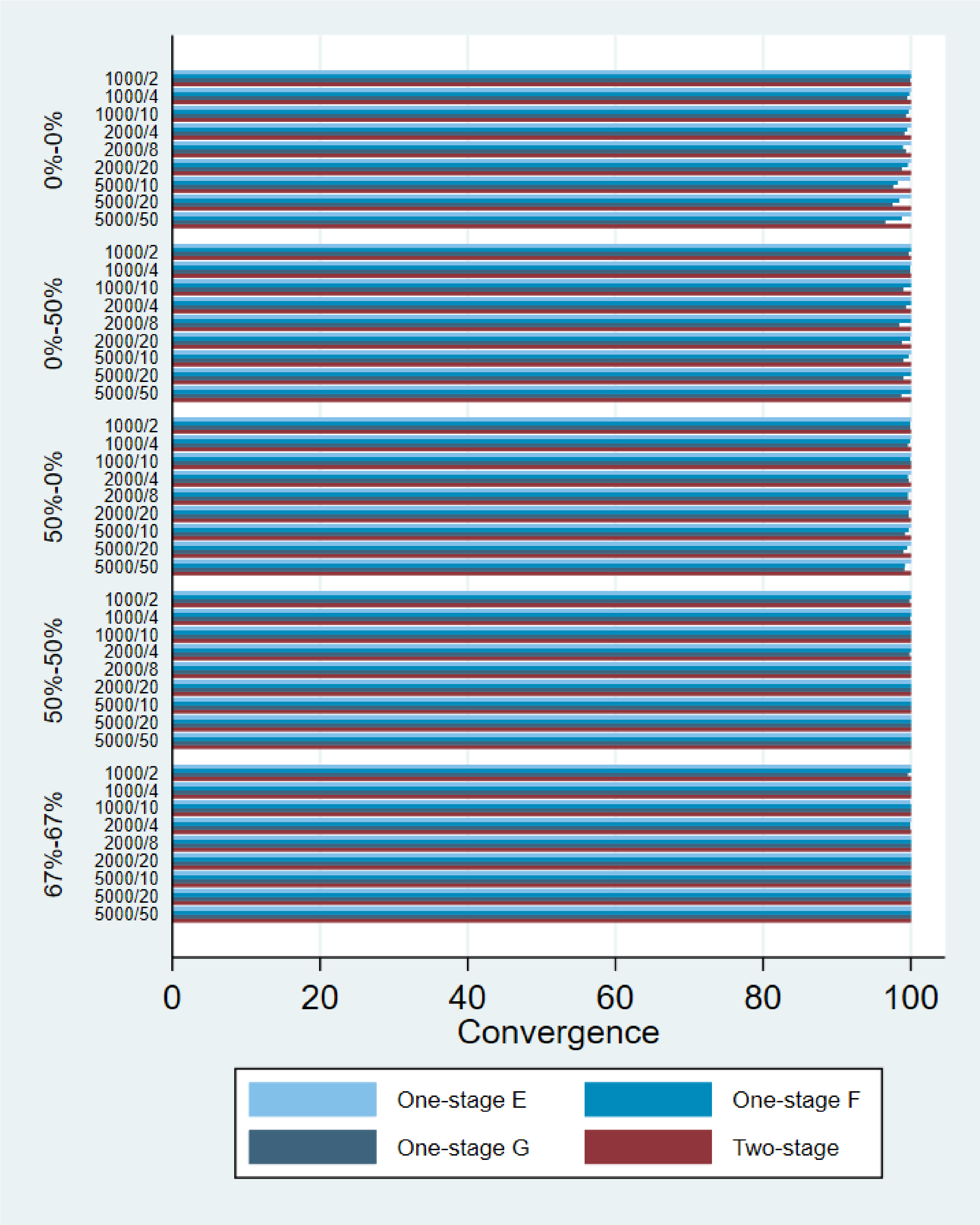

Supplement: Supplementary file 1 — Data S1. Figure A1: Mean Bias Figure A2: Mean Error Figure A3: Coverage probability (%), against 95% nominal line Figure A4: Power probability (%) Figure A5: Coverage and Power (%), plotted together [(coverage+power)/2] Figure A6: Model convergence (%) Figure A7: Mean Bias Figure A8: Mean Error Figure A9: Coverage probability (%), against 95% nominal line Figure A10: Power probability (%) Figure A11: Coverage and Power (%), plotted together [(coverage+power)/2] Figure A12: Model convergence (%) Figure A13: Mean Bias Figure A14: Mean Error Figure A15: Coverage probability (%), against 95% nominal line Figure A16: Power probability (%) Figure A17: Coverage and Power (%), plotted together [(coverage+power)/2] Figure A18: Model convergence (%) Figure A19: Mean Bias Figure A20: Mean Error Figure A21: Coverage probability (%), against 95% nominal line Figure A22: Power probability (%) Figure A23: Coverage and Power (%), plotted together [(coverage+power)/2] Figure A24: Model convergence (%) Figure A25: Mean Bias Figure A26: Mean Error Figure A27: Coverage probability (%), against 95% nominal line Figure A28: Power probability (%) Figure A29: Coverage and Power (%), plotted together [(coverage+power)/2] Figure A30: Model convergence (%) Figure A31: Mean Bias Figure A32: Mean Error Figure A33: Coverage probability (%), against 95% nominal line Figure A34: Power probability (%) Figure A35: Coverage and Power (%), plotted together [(coverage+power)/2] Figure A36: Model convergence (%) Figure A37: Mean Bias Figure A38: Mean Error Figure A39: Coverage probability (%), against 95% nominal lin Figure A40: Power probability (%) Figure A41: Coverage and Power (%), plotted together [(coverage+power)/2] Figure A42: Model convergence (%) Figure A43: Mean Bias, models A‐D Figure A44: Mean Bias, models D & E‐G Figure A45: Mean Error, models A‐D Figure A46: Mean Error, models D & E‐G Figure A47: Coverage probability (%), against 95% nominal line, models A‐D Figure A48: Coverage probabili [file JRSM-9-417-s001.pdf]
