# Supplementary material for: A comparison of one‐stage vs two‐stage individual patient data meta‐analysis methods: A simulation study
Source: Res Synth Methods. 2018 Jun 21;9(3):417–30. doi: 10.1002/jrsm.1303 (PMC6175226; doi:10.1002/jrsm.1303)
Supplement: Supplementary file 2 — Data S2. Supporting Information [file JRSM-9-417-s002.pdf]

Main results, main effect

Convergence

ssl	ssh	tsq0	tsq1	outc	beta	model1	model2	model3	model4
5000	10	0	0	0	.5	99.2	100	99.6	100
2000	4	0	0	0	.5	99.5	99.7	98.5	100
1000	2	0	0	0	.5	100	100	99.4	100
5000	20	0	0	0	.5	99.1	99.9	98.6	100
2000	8	0	0	0	.5	99.7	100	98.8	100
1000	4	0	0	0	.5	99.8	100	98.1	100
5000	50	0	0	0	.5	98.3	100	98.2	100
2000	20	0	0	0	.5	99.7	100	98.9	100
1000	10	0	0	0	.5	99.6	100	98.8	100
5000	10	1	0	0	.5	100	99.9	96.7	100
2000	4	1	0	0	.5	100	100	96.2	100
1000	2	1	0	0	.5	100	100	95.9	100
5000	20	1	0	0	.5	100	100	97.8	100
2000	8	1	0	0	.5	100	100	96.5	100
1000	4	1	0	0	.5	100	100	96.7	100
5000	50	1	0	0	.5	100	100	96.8	100
2000	20	1	0	0	.5	100	100	97.4	100
1000	10	1	0	0	.5	100	100	97.2	100
5000	10	0	1	0	.5	100	100	95.5	100
2000	4	0	1	0	.5	100	100	95.2	100
1000	2	0	1	0	.5	100	100	94.2	100
5000	20	0	1	0	.5	100	100	94.6	100
2000	8	0	1	0	.5	100	100	96.2	100
1000	4	0	1	0	.5	100	100	95.4	100
5000	50	0	1	0	.5	100	100	96.2	100
2000	20	0	1	0	.5	100	100	97	100
1000	10	0	1	0	.5	100	100	96.4	100
5000	10	1	1	0	.5	100	100	100	100
2000	4	1	1	0	.5	100	100	99.7	100
1000	2	1	1	0	.5	100	100	86.7	100
5000	20	1	1	0	.5	100	100	100	100
2000	8	1	1	0	.5	100	100	100	100
1000	4	1	1	0	.5	100	100	99.4	100
5000	50	1	1	0	.5	100	100	100	100
2000	20	1	1	0	.5	100	100	100	100
1000	10	1	1	0	.5	100	100	100	100
5000	10	.5	.5	0	.5	100	100	100	100
2000	4	.5	.5	0	.5	100	100	99.2	100
1000	2	.5	.5	0	.5	99.9	100	91.6	100
5000	20	.5	.5	0	.5	100	100	100	100
2000	8	.5	.5	0	.5	100	100	100	100
1000	4	.5	.5	0	.5	100	100	99	100
5000	50	.5	.5	0	.5	100	100	100	100
2000	20	.5	.5	0	.5	100	100	100	100
1000	10	.5	.5	0	.5	100	100	100	100
5000	10	2	2	0	.5	100	100	100	100
2000	4	2	2	0	.5	100	100	99.5	100
1000	2	2	2	0	.5	100	100	78.7	100
5000	20	2	2	0	.5	100	100	100	100
2000	8	2	2	0	.5	100	100	100	100
1000	4	2	2	0	.5	100	100	99.1	100
5000	50	2	2	0	.5	100	100	100	100
2000	20	2	2	0	.5	100	100	100	100
1000	10	2	2	0	.5	100	100	100	100

SE of convergence

ssl	ssh	tsq0	tsq1	outc	beta	model1	model2	model3	model4
5000	10	0	0	0	.5	.2817091	0	.1995996	0
2000	4	0	0	0	.5	.2230471	.1729451	.3843826	0
1000	2	0	0	0	.5	0	0	.244213	0
5000	20	0	0	0	.5	.2986469	.09995	.3715373	0
2000	8	0	0	0	.5	.1729451	0	.3443254	0
1000	4	0	0	0	.5	.1412799	0	.4317291	0
5000	50	0	0	0	.5	.4087909	0	.4204284	0
2000	20	0	0	0	.5	.1729451	0	.3298333	0
1000	10	0	0	0	.5	.1995996	0	.3443254	0
5000	10	1	0	0	.5	0	.09995	.5648982	0
2000	4	1	0	0	.5	0	0	.6046156	0
1000	2	1	0	0	.5	0	0	.6270486	0
5000	20	1	0	0	.5	0	0	.4638534	0
2000	8	1	0	0	.5	0	0	.5811626	0
1000	4	1	0	0	.5	0	0	.5648982	0
5000	50	1	0	0	.5	0	0	.5565609	0
2000	20	1	0	0	.5	0	0	.5032296	0
1000	10	1	0	0	.5	0	0	.5216896	0
5000	10	0	1	0	.5	0	0	.6555532	0
2000	4	0	1	0	.5	0	0	.6759882	0
1000	2	0	1	0	.5	0	0	.7391617	0
5000	20	0	1	0	.5	0	0	.7147307	0

2000	8	0	1	0	.5	0	0	.6046156	0
1000	4	0	1	0	.5	0	0	.66245	0
5000	50	0	1	0	.5	0	0	.6046156	0
2000	20	0	1	0	.5	0	0	.5394442	0
1000	10	0	1	0	.5	0	0	.589101	0
5000	10	1	1	0	.5	0	0	0	0
2000	4	1	1	0	.5	0	0	.1729451	0
1000	2	1	1	0	.5	0	0	1.07383	0
5000	20	1	1	0	.5	0	0	0	0
2000	8	1	1	0	.5	0	0	0	0
1000	4	1	1	0	.5	0	0	.244213	0
5000	50	1	1	0	.5	0	0	0	0
2000	20	1	1	0	.5	0	0	0	0
1000	10	1	1	0	.5	0	0	0	0
5000	10	.5	.5	0	.5	0	0	0	0
2000	4	.5	.5	0	.5	0	0	.2817091	0
1000	2	.5	.5	0	.5	.09995	0	.8771773	0
5000	20	.5	.5	0	.5	0	0	0	0
2000	8	.5	.5	0	.5	0	0	0	0
1000	4	.5	.5	0	.5	0	0	.3146427	0
5000	50	.5	.5	0	.5	0	0	0	0
2000	20	.5	.5	0	.5	0	0	0	0
1000	10	.5	.5	0	.5	0	0	0	0
5000	10	2	2	0	.5	0	0	0	0
2000	4	2	2	0	.5	0	0	.2230471	0
1000	2	2	2	0	.5	0	0	1.294724	0
5000	20	2	2	0	.5	0	0	0	0
2000	8	2	2	0	.5	0	0	0	0
1000	4	2	2	0	.5	0	0	.2986469	0
5000	50	2	2	0	.5	0	0	0	0
2000	20	2	2	0	.5	0	0	0	0
1000	10	2	2	0	.5	0	0	0	0

Coverage

ssl	ssh	tsq0	tsq1	outc	beta	model1	model2	model3	model4
5000	10	0	0	0	.5	96.37097	96.1	96.58635	96.5
2000	4	0	0	0	.5	95.57789	96.18857	96.85279	96.8
1000	2	0	0	0	.5	95.6	95.1	95.47284	95.6
5000	20	0	0	0	.5	93.23915	93.19319	93.71197	94.1
2000	8	0	0	0	.5	94.08225	93.9	95.04049	94.9
1000	4	0	0	0	.5	96.69339	96.4	96.9419	97.4
5000	50	0	0	0	.5	95.21872	94	95.41752	95.6
2000	20	0	0	0	.5	95.58676	94.7	96.05662	95.9
1000	10	0	0	0	.5	96.38554	95.5	96.86235	96.5
5000	10	1	0	0	.5	100	93.99399	95.65667	95.4
2000	4	1	0	0	.5	100	95.6	96.15385	96.7
1000	2	1	0	0	.5	99.2	93.7	95.72471	95.4
5000	20	1	0	0	.5	100	95.7	96.72802	96.9
2000	8	1	0	0	.5	100	95.2	96.68394	96.4
1000	4	1	0	0	.5	99.8	93.6	95.96691	95.1
5000	50	1	0	0	.5	100	94.8	95.24793	95.4
2000	20	1	0	0	.5	100	95.4	96.50924	96.4
1000	10	1	0	0	.5	100	93.8	95.78189	95
5000	10	0	1	0	.5	90.5	90.5	90.36649	92.2
2000	4	0	1	0	.5	81.1	80.5	80.14706	85
1000	2	0	1	0	.5	61.3	60.1	59.97877	71.1
5000	20	0	1	0	.5	91.8	91.8	92.07188	92.9
2000	8	0	1	0	.5	89.6	88.4	89.08524	91.1
1000	4	0	1	0	.5	81.9	80.4	81.2369	85.4
5000	50	0	1	0	.5	94.6	94.4	94.28274	94.6
2000	20	0	1	0	.5	92.2	91.8	92.37113	93.1
1000	10	0	1	0	.5	89.5	89	89.31535	91.4
5000	10	1	1	0	.5	96	90.1	90.3	91.1
2000	4	1	1	0	.5	90.2	81	81.54463	85.3
1000	2	1	1	0	.5	68	60	60.43829	70
5000	20	1	1	0	.5	97.6	92.7	93	93.8
2000	8	1	1	0	.5	96.1	89.2	89.5	91.6
1000	4	1	1	0	.5	89.9	79.9	80.28169	85.5
5000	50	1	1	0	.5	98.4	92.7	92.8	93.1
2000	20	1	1	0	.5	98.4	92.8	93	93.6
1000	10	1	1	0	.5	96.5	88.6	89.3	91
5000	10	.5	.5	0	.5	95.9	89.1	89.1	90.9
2000	4	.5	.5	0	.5	87.1	80.1	81.04839	85.2
1000	2	.5	.5	0	.5	70.67067	61.2	64.51965	71.4
5000	20	.5	.5	0	.5	98.3	93.2	93.4	94.4
2000	8	.5	.5	0	.5	95	86.5	86.9	89.4
1000	4	.5	.5	0	.5	89.5	79.5	80.60606	84.1
5000	50	.5	.5	0	.5	99.1	93.3	93.6	94.2
2000	20	.5	.5	0	.5	98.2	91.3	91.7	92.3
1000	10	.5	.5	0	.5	97	89.4	90.9	92.1
5000	10	2	2	0	.5	95.1	89	89.1	90.6
2000	4	2	2	0	.5	89.9	80.5	80.50251	84.8
1000	2	2	2	0	.5	65.4	58.3	56.41677	68.4
5000	20	2	2	0	.5	98	94.6	94.7	95.5
2000	8	2	2	0	.5	94.2	87.9	88.1	89.9
1000	4	2	2	0	.5	90.5	80.7	80.72654	85.3
5000	50	2	2	0	.5	98.4	93.5	93.9	94.1
2000	20	2	2	0	.5	98.1	92.5	92.7	93.3
1000	10	2	2	0	.5	97.4	91	91.1	92.1

SE of coverage

ssl	ssh	tsq0	tsq1	outc	beta	model1	model2	model3	model4
5000	10	0	0	0	.5	.5937624	.6122009	.5753578	.5811626
2000	4	0	0	0	.5	.6517514	.6063988	.5562893	.5565609
1000	2	0	0	0	.5	.6485677	.6826346	.6594165	.6485677
5000	20	0	0	0	.5	.7975592	.7968582	.7730658	.7451107
2000	8	0	0	0	.5	.7472823	.7568289	.6907095	.6956939
1000	4	0	0	0	.5	.5660109	.589101	.5497274	.5032296
5000	50	0	0	0	.5	.6805446	.7509993	.6672811	.6485677
2000	20	0	0	0	.5	.6504742	.7084561	.6188703	.6270486
1000	10	0	0	0	.5	.5914225	.6555532	.5546275	.5811626
5000	10	1	0	0	.5	0	.7517271	.6554745	.66245
2000	4	1	0	0	.5	0	.6485677	.6200249	.5648982
1000	2	1	0	0	.5	.2817091	.7683163	.6532589	.66245
5000	20	1	0	0	.5	0	.6414905	.568869	.5480785
2000	8	1	0	0	.5	0	.6759882	.5764012	.589101
1000	4	1	0	0	.5	.1412799	.7739767	.6326545	.6826346
5000	50	1	0	0	.5	0	.7021111	.6838035	.66245
2000	20	1	0	0	.5	0	.66245	.5881186	.589101
1000	10	1	0	0	.5	0	.7626008	.6447144	.6892024
5000	10	0	1	0	.5	.927227	.927227	.9547601	.848033
2000	4	0	1	0	.5	1.238059	1.252897	1.292819	1.129159
1000	2	0	1	0	.5	1.540231	1.548544	1.596315	1.433454
5000	20	0	1	0	.5	.8676174	.8676174	.8784216	.8121515
2000	8	0	1	0	.5	.9653186	1.01264	1.005362	.9004388
1000	4	0	1	0	.5	1.217534	1.255325	1.264023	1.11662
5000	50	0	1	0	.5	.7147307	.7270763	.7485527	.7147307
2000	20	0	1	0	.5	.848033	.8676174	.8523388	.8014924
1000	10	0	1	0	.5	.969407	.9894443	.9949577	.886589
5000	10	1	1	0	.5	.6196773	.9444522	.9359006	.9004388
2000	4	1	1	0	.5	.9401915	1.240564	1.228603	1.119781
1000	2	1	1	0	.5	1.475127	1.549193	1.660673	1.449138
5000	20	1	1	0	.5	.4839835	.8226239	.8068457	.7626008
2000	8	1	1	0	.5	.6122009	.981509	.969407	.8771773
1000	4	1	1	0	.5	.9528851	1.267277	1.261973	1.113441
5000	50	1	1	0	.5	.3967871	.8226239	.8174105	.8014924
2000	20	1	1	0	.5	.3967871	.8174105	.8068457	.7739767
1000	10	1	1	0	.5	.5811626	1.005007	.9775019	.9049862
5000	10	.5	.5	0	.5	.6270486	.9854897	.9854897	.9094999
2000	4	.5	.5	0	.5	1.059995	1.262533	1.244341	1.122925
1000	2	.5	.5	0	.5	1.440416	1.540961	1.580856	1.429
5000	20	.5	.5	0	.5	.4087909	.7960904	.7851369	.7270763
2000	8	.5	.5	0	.5	.6892024	1.080625	1.066954	.973468
1000	4	.5	.5	0	.5	.969407	1.276617	1.256606	1.156369
5000	50	.5	.5	0	.5	.2986469	.790639	.7739767	.7391617
2000	20	.5	.5	0	.5	.4204284	.8912407	.8724162	.8430362
1000	10	.5	.5	0	.5	.5394442	.973468	.9094999	.8529889
5000	10	2	2	0	.5	.6826346	.9894443	.9854897	.9228434
2000	4	2	2	0	.5	.9528851	1.252897	1.255979	1.135324
1000	2	2	2	0	.5	1.504274	1.559202	1.767569	1.470184
5000	20	2	2	0	.5	.4427189	.7147307	.7084561	.6555532
2000	8	2	2	0	.5	.7391617	1.031305	1.023909	.9528851
1000	4	2	2	0	.5	.927227	1.248002	1.253	1.119781
5000	50	2	2	0	.5	.3967871	.7795832	.7568289	.7451107
2000	20	2	2	0	.5	.4317291	.8329166	.8226239	.790639
1000	10	2	2	0	.5	.5032296	.9049862	.9004388	.8529889

Mean error

ssl	ssh	tsq0	tsq1	outc	beta	model1	model2	model3	model4
5000	10	0	0	0	.5	.0219807	.0220177	.0221478	.0222511
2000	4	0	0	0	.5	.0358282	.0357723	.0361255	.0365792
1000	2	0	0	0	.5	.0514914	.0518484	.0531961	.0555726
5000	20	0	0	0	.5	.0241545	.024225	.0243421	.0243476
2000	8	0	0	0	.5	.035249	.0353822	.0354154	.0354527
1000	4	0	0	0	.5	.0491438	.0487426	.0490311	.0497346
5000	50	0	0	0	.5	.0232904	.0233347	.0232104	.0237866
2000	20	0	0	0	.5	.0363445	.036703	.0365459	.036737
1000	10	0	0	0	.5	.0507713	.0509529	.051353	.0516423
5000	10	1	0	0	.5	.1335261	.0231971	.0236354	.0234034
2000	4	1	0	0	.5	.1984541	.0351672	.0369165	.0362666
1000	2	1	0	0	.5	.2589226	.05323	.0633993	.0566575
5000	20	1	0	0	.5	.0809831	.0216745	.0215615	.0218802
2000	8	1	0	0	.5	.1223403	.0357265	.0361898	.0363242
1000	4	1	0	0	.5	.169815	.0513406	.052487	.0519516
5000	50	1	0	0	.5	.0380419	.022265	.0220756	.0222836
2000	20	1	0	0	.5	.0595662	.0347931	.0349366	.0350851
1000	10	1	0	0	.5	.084195	.0526695	.0534297	.0535016
5000	10	0	1	0	.5	.2518915	.2515215	.2535663	.2515408
2000	4	0	1	0	.5	.3985945	.3985519	.4012092	.3986602
1000	2	0	1	0	.5	.572172	.5744419	.5724817	.5723676
5000	20	0	1	0	.5	.1900953	.1902284	.1903925	.1901802
2000	8	0	1	0	.5	.2972017	.2968567	.2952905	.2967862
1000	4	0	1	0	.5	.4010492	.4012855	.403086	.4014271
5000	50	0	1	0	.5	.1133125	.1134415	.1136008	.1134435
2000	20	0	1	0	.5	.1841191	.1846291	.1833956	.1846368
1000	10	0	1	0	.5	.2713402	.2720434	.2729604	.2720521
5000	10	1	1	0	.5	.2919717	.2560385	.2559221	.2560633
2000	4	1	1	0	.5	.4483684	.399397	.3992881	.3993784
1000	2	1	1	0	.5	.6315677	.5556429	.5547501	.5545532
5000	20	1	1	0	.5	.1952573	.1782931	.1784659	.1783025
2000	8	1	1	0	.5	.3130098	.289581	.2896604	.2896019

1000	4	1	1	0	.5	.4386861	.4089874	.4095718	.4092903
5000	50	1	1	0	.5	.1231847	.1190292	.1190187	.1190056
2000	20	1	1	0	.5	.1857524	.1782588	.1782665	.1783025
1000	10	1	1	0	.5	.2711181	.2665456	.2663227	.2665499
5000	10	.5	.5	0	.5	.2048701	.1828186	.1828753	.1828902
2000	4	.5	.5	0	.5	.327006	.2989787	.2996574	.29878
1000	2	.5	.5	0	.5	.4437755	.3969602	.3939692	.3964864
5000	20	.5	.5	0	.5	.134179	.1241517	.1241008	.124179
2000	8	.5	.5	0	.5	.2233257	.2046052	.2046034	.2046101
1000	4	.5	.5	0	.5	.3094094	.2865404	.2860921	.2865023
5000	50	.5	.5	0	.5	.0877795	.0844342	.0844508	.0844311
2000	20	.5	.5	0	.5	.1387586	.1373541	.1373329	.1374817
1000	10	.5	.5	0	.5	.1925168	.1862023	.186163	.1860605
5000	10	2	2	0	.5	.4152352	.370261	.3702541	.3702544
2000	4	2	2	0	.5	.6199469	.5697192	.5681377	.5697692
1000	2	2	2	0	.5	.9241954	.8162147	.8161388	.8158788
5000	20	2	2	0	.5	.2712468	.2508325	.250901	.2508371
2000	8	2	2	0	.5	.4339258	.4009658	.4008483	.4009742
1000	4	2	2	0	.5	.6288604	.5672184	.5684958	.5673478
5000	50	2	2	0	.5	.1664259	.1639844	.1639524	.1639744
2000	20	2	2	0	.5	.2656808	.2564948	.2564602	.256423
1000	10	2	2	0	.5	.3689002	.3601702	.3601048	.3601266

SE of mean error

ssl	ssh	tsq0	tsq1	outc	beta	model1	model2	model3	model4
5000	10	0	0	0	.5	.0000168	.0000167	.0000168	.0000168
2000	4	0	0	0	.5	.0000269	.0000267	.0000272	.0000275
1000	2	0	0	0	.5	.0000375	.0000379	.0000401	.0000424
5000	20	0	0	0	.5	.0000184	.0000181	.0000184	.0000182
2000	8	0	0	0	.5	.0000278	.0000276	.0000281	.0000281
1000	4	0	0	0	.5	.0000358	.0000358	.0000372	.0000371
5000	50	0	0	0	.5	.0000173	.0000171	.0000173	.0000171
2000	20	0	0	0	.5	.0000271	.0000271	.0000273	.0000275
1000	10	0	0	0	.5	.0000379	.000038	.0000387	.0000388
5000	10	1	0	0	.5	.000099	.0000175	.0000185	.0000178
2000	4	1	0	0	.5	.0001685	.0000274	.0000295	.0000281
1000	2	1	0	0	.5	.0002697	.0000415	.0000526	.0000448
5000	20	1	0	0	.5	.0000627	.0000167	.0000171	.0000169
2000	8	1	0	0	.5	.0000963	.0000277	.0000288	.000028
1000	4	1	0	0	.5	.0001462	.0000401	.0000423	.0000407
5000	50	1	0	0	.5	.0000278	.0000174	.0000179	.0000174
2000	20	1	0	0	.5	.000047	.0000257	.0000268	.0000262
1000	10	1	0	0	.5	.0000671	.0000396	.0000409	.00004
5000	10	0	1	0	.5	.0001952	.0001954	.000206	.0001954
2000	4	0	1	0	.5	.0002925	.0002919	.0003079	.0002917
1000	2	0	1	0	.5	.0004346	.0004349	.0004622	.0004355
5000	20	0	1	0	.5	.0001419	.0001422	.0001506	.0001422
2000	8	0	1	0	.5	.0002105	.0002107	.0002175	.0002107
1000	4	0	1	0	.5	.0003082	.0003087	.0003235	.0003085
5000	50	0	1	0	.5	.0000845	.0000847	.000088	.0000847
2000	20	0	1	0	.5	.0001422	.0001423	.0001463	.0001423
1000	10	0	1	0	.5	.0001998	.0001998	.0002078	.0001999
5000	10	1	1	0	.5	.0002099	.0001903	.0001903	.0001903
2000	4	1	1	0	.5	.0003342	.0003041	.0003053	.0003042
1000	2	1	1	0	.5	.0004921	.0004166	.0004856	.0004186
5000	20	1	1	0	.5	.0001476	.0001356	.0001355	.0001356
2000	8	1	1	0	.5	.0002332	.0002135	.0002134	.0002134
1000	4	1	1	0	.5	.0003303	.0003044	.0003066	.0003045
5000	50	1	1	0	.5	.0000941	.0000912	.0000912	.0000912
2000	20	1	1	0	.5	.0001377	.0001322	.0001322	.0001322
1000	10	1	1	0	.5	.0002079	.000202	.0002021	.0002021
5000	10	.5	.5	0	.5	.000154	.0001415	.0001415	.0001415
2000	4	.5	.5	0	.5	.0002506	.0002281	.00023	.0002282
1000	2	.5	.5	0	.5	.0003489	.000314	.0003359	.0003133
5000	20	.5	.5	0	.5	.0001021	.0000953	.0000951	.0000953
2000	8	.5	.5	0	.5	.0001628	.0001486	.0001486	.0001486
1000	4	.5	.5	0	.5	.0002419	.0002229	.000225	.000223
5000	50	.5	.5	0	.5	.000064	.0000626	.0000626	.0000627
2000	20	.5	.5	0	.5	.0001042	.0001026	.0001026	.0001025
1000	10	.5	.5	0	.5	.0001439	.0001418	.0001415	.0001417
5000	10	2	2	0	.5	.0003109	.0002763	.0002763	.0002763
2000	4	2	2	0	.5	.0004839	.0004378	.0004391	.0004377
1000	2	2	2	0	.5	.0007106	.0006324	.0007978	.0006321
5000	20	2	2	0	.5	.0002014	.0001848	.0001847	.0001849
2000	8	2	2	0	.5	.0003355	.0003058	.0003059	.0003058
1000	4	2	2	0	.5	.0004452	.0004092	.0004128	.000409
5000	50	2	2	0	.5	.0001263	.0001237	.0001236	.0001237
2000	20	2	2	0	.5	.0002051	.0001954	.0001954	.0001954
1000	10	2	2	0	.5	.0002763	.0002669	.0002669	.0002669

Mean bias

ssl	ssh	tsq0	tsq1	outc	beta	model1	model2	model3	model4
5000	10	0	0	0	.5	.0019006	.0019125	.0019178	.001894
2000	4	0	0	0	.5	-.0017911	-.0019196	-.0014505	-.0017131
1000	2	0	0	0	.5	.0049718	.005052	.0049601	.0050338
5000	20	0	0	0	.5	-.0020873	-.0020089	-.0021604	-.0020041
2000	8	0	0	0	.5	-.0012374	-.0012534	-.0007916	-.0011138

1000	4	0	0	0	.5	-.0005356	-.0007942	-.0012714	-.0008379
5000	50	0	0	0	.5	-.0003492	-.0004164	-.0003975	-.0004069
2000	20	0	0	0	.5	-.0033945	-.0034641	-.0038581	-.0033744
1000	10	0	0	0	.5	.0001268	-.0004152	-.0004509	-.0007456
5000	10	1	0	0	.5	-.0140962	-.000607	-.0004227	-.0007541
2000	4	1	0	0	.5	.0096583	.000522	.0006606	.000436
1000	2	1	0	0	.5	-.0060185	-.0002192	-.0001393	-.0006912
5000	20	1	0	0	.5	.0020442	-.0002566	-.0004332	-.0001443
2000	8	1	0	0	.5	-.0038721	-.0023933	-.0026207	-.002371
1000	4	1	0	0	.5	-.0076191	.0028313	.0023273	.0031717
5000	50	1	0	0	.5	-.0013898	.0002761	.0000291	.0002324
2000	20	1	0	0	.5	.0026534	.0005808	.0007479	.0004886
1000	10	1	0	0	.5	.000019	-.0005082	.000454	-.0002976
5000	10	0	1	0	.5	-.0000457	-.0009705	-.0018932	-.0009333
2000	4	0	1	0	.5	.0076776	.0071411	.0102339	.0070543
1000	2	0	1	0	.5	.0004008	.0010035	-.0065321	.0003071
5000	20	0	1	0	.5	-.0049257	-.0049655	-.0066737	-.0049763
2000	8	0	1	0	.5	-.0112364	-.0115502	-.0078941	-.0115926
1000	4	0	1	0	.5	-.0051353	-.0061056	-.0030758	-.0061417
5000	50	0	1	0	.5	.0012237	.0016884	.0008635	.0016544
2000	20	0	1	0	.5	.0012886	.0009226	-.0012186	.0009416
1000	10	0	1	0	.5	-.0104149	-.0104954	-.0110071	-.010515
5000	10	1	1	0	.5	.0008553	.0053099	.0053933	.0053446
2000	4	1	1	0	.5	.0173382	.0198719	.0225026	.0197153
1000	2	1	1	0	.5	.0237935	.0048332	.0001525	.0052676
5000	20	1	1	0	.5	.0018085	.001028	.0010625	.0010312
2000	8	1	1	0	.5	-.0120813	-.0121955	-.0122072	-.0121391
1000	4	1	1	0	.5	-.0101309	-.0209085	-.0209704	-.0209125
5000	50	1	1	0	.5	.0012603	.0000513	.0000729	-.0000296
2000	20	1	1	0	.5	.0026065	.0049831	.0049359	.0049278
1000	10	1	1	0	.5	.0026431	.0044002	.0044744	.0042488
5000	10	.5	.5	0	.5	.0043469	.007458	.0073205	.0075613
2000	4	.5	.5	0	.5	.0037599	.0114761	.0121029	.0113185
1000	2	.5	.5	0	.5	-.0285369	-.0260154	-.021331	-.0267252
5000	20	.5	.5	0	.5	.0020666	.0018896	.0018884	.0019318
2000	8	.5	.5	0	.5	-.0018474	-.0019708	-.0021615	-.0019118
1000	4	.5	.5	0	.5	-.0004367	-.0050606	-.0057397	-.0052107
5000	50	.5	.5	0	.5	-.0024144	-.0029627	-.0029402	-.0029509
2000	20	.5	.5	0	.5	-.0022883	-.0003105	-.0003579	-.0002674
1000	10	.5	.5	0	.5	-.0141502	-.0120669	-.0121145	-.0121809
5000	10	2	2	0	.5	-.0207867	-.0218243	-.0219996	-.0218062
2000	4	2	2	0	.5	.0087713	.0181708	.0144414	.0181395
1000	2	2	2	0	.5	.1012426	.0692948	.0911812	.0692546
5000	20	2	2	0	.5	-.0063301	-.0078261	-.0078049	-.0078096
2000	8	2	2	0	.5	-.0142844	-.0055891	-.0057656	-.0055896
1000	4	2	2	0	.5	.0263092	.021291	.0213528	.0212767
5000	50	2	2	0	.5	.010255	.0086596	.0086557	.008654
2000	20	2	2	0	.5	-.0073487	-.009681	-.0096483	-.0097176
1000	10	2	2	0	.5	.0085371	.0027615	.00283	.002913

SE of mean bias

ssl	ssh	tsq0	tsq1	outc	beta	model1	model2	model3	model4
5000	10	0	0	0	.5	.0000278	.0000276	.0000278	.0000278
2000	4	0	0	0	.5	.000045	.0000447	.0000456	.0000458
1000	2	0	0	0	.5	.0000635	.000064	.0000667	.0000698
5000	20	0	0	0	.5	.0000305	.0000302	.0000307	.0000303
2000	8	0	0	0	.5	.000045	.0000449	.0000455	.0000452
1000	4	0	0	0	.5	.0000609	.0000605	.0000623	.0000621
5000	50	0	0	0	.5	.0000294	.0000289	.0000293	.0000293
2000	20	0	0	0	.5	.0000453	.0000455	.0000458	.0000458
1000	10	0	0	0	.5	.0000635	.0000636	.0000648	.0000646
5000	10	1	0	0	.5	.0001657	.0000291	.0000307	.0000294
2000	4	1	0	0	.5	.0002602	.0000446	.0000484	.0000459
1000	2	1	0	0	.5	.0003739	.0000675	.0000845	.0000723
5000	20	1	0	0	.5	.0001024	.0000274	.0000279	.0000276
2000	8	1	0	0	.5	.0001557	.0000452	.0000472	.0000458
1000	4	1	0	0	.5	.000224	.0000651	.0000688	.000066
5000	50	1	0	0	.5	.0000471	.0000283	.000029	.0000283
2000	20	1	0	0	.5	.0000759	.0000433	.0000448	.0000438
1000	10	1	0	0	.5	.0001077	.0000659	.0000685	.0000668
5000	10	0	1	0	.5	.0003188	.0003186	.0003362	.0003186
2000	4	0	1	0	.5	.0004945	.0004941	.000522	.0004941
1000	2	0	1	0	.5	.0007187	.0007207	.0007637	.0007194
5000	20	0	1	0	.5	.0002372	.0002375	.0002514	.0002375
2000	8	0	1	0	.5	.0003641	.000364	.0003762	.0003639
1000	4	0	1	0	.5	.000506	.0005064	.0005323	.0005064
5000	50	0	1	0	.5	.0001414	.0001416	.0001473	.0001416
2000	20	0	1	0	.5	.0002327	.0002332	.0002391	.0002332
1000	10	0	1	0	.5	.0003369	.0003375	.0003512	.0003375
5000	10	1	1	0	.5	.0003597	.000319	.000319	.0003191
2000	4	1	1	0	.5	.0005591	.0005018	.0005032	.0005018
1000	2	1	1	0	.5	.0008005	.0006947	.0008036	.000695
5000	20	1	1	0	.5	.0002449	.0002241	.0002242	.0002241
2000	8	1	1	0	.5	.0003903	.0003597	.0003597	.0003596
1000	4	1	1	0	.5	.0005492	.0005096	.0005134	.0005099
5000	50	1	1	0	.5	.0001551	.00015	.00015	.00015
2000	20	1	1	0	.5	.0002313	.0002219	.0002219	.000222
1000	10	1	1	0	.5	.0003418	.0003345	.0003344	.0003346
5000	10	.5	.5	0	.5	.0002564	.0002312	.0002312	.0002312
2000	4	.5	.5	0	.5	.0004121	.000376	.0003796	.0003759
1000	2	.5	.5	0	.5	.0005643	.0005056	.0005454	.0005048
5000	20	.5	.5	0	.5	.0001686	.0001565	.0001564	.0001566
2000	8	.5	.5	0	.5	.0002764	.000253	.000253	.000253
1000	4	.5	.5	0	.5	.0003928	.0003631	.0003663	.0003632

5000	50	.5	.5	0	.5	.0001086	.0001051	.0001051	.0001051
2000	20	.5	.5	0	.5	.0001736	.0001715	.0001715	.0001716
1000	10	.5	.5	0	.5	.00024	.0002338	.0002336	.0002337
5000	10	2	2	0	.5	.0005185	.0004616	.0004616	.0004616
2000	4	2	2	0	.5	.0007867	.0007185	.0007204	.0007185
1000	2	2	2	0	.5	.0011618	.0010305	.0013038	.0010301
5000	20	2	2	0	.5	.0003379	.0003116	.0003116	.0003116
2000	8	2	2	0	.5	.0005485	.0005044	.0005044	.0005044
1000	4	2	2	0	.5	.0007703	.0006993	.0007066	.0006993
5000	50	2	2	0	.5	.0002088	.0002053	.0002052	.0002053
2000	20	2	2	0	.5	.0003356	.0003224	.0003224	.0003223
1000	10	2	2	0	.5	.000461	.0004484	.0004483	.0004484

Power

ssl	ssh	tsq0	tsq1	outc	beta	model1	model2	model3	model4
5000	10	0	0	0	.5	100	100	100	100
2000	4	0	0	0	.5	100	100	100	100
1000	2	0	0	0	.5	99.9	100	99.69819	99
5000	20	0	0	0	.5	100	100	100	100
2000	8	0	0	0	.5	100	100	100	100
1000	4	0	0	0	.5	100	100	100	100
5000	50	0	0	0	.5	100	100	100	100
2000	20	0	0	0	.5	100	100	100	100
1000	10	0	0	0	.5	100	100	100	100
5000	10	1	0	0	.5	36.6	100	100	100
2000	4	1	0	0	.5	27.5	100	100	100
1000	2	1	0	0	.5	41.6	99.9	99.68717	99.1
5000	20	1	0	0	.5	74.3	100	100	100
2000	8	1	0	0	.5	33.7	100	100	100
1000	4	1	0	0	.5	25.9	100	100	100
5000	50	1	0	0	.5	100	100	100	100
2000	20	1	0	0	.5	77.2	100	100	100
1000	10	1	0	0	.5	28.4	100	100	100
5000	10	0	1	0	.5	42	42	41.88482	39
2000	4	0	1	0	.5	32.7	33.1	33.71849	25.9
1000	2	0	1	0	.5	39.3	39.8	38.64119	29.7
5000	20	0	1	0	.5	60.7	61.1	60.78224	59
2000	8	0	1	0	.5	38.9	39.5	39.50104	34.8
1000	4	0	1	0	.5	33.3	34	33.6478	27.3
5000	50	0	1	0	.5	93.9	93.9	93.45114	93.6
2000	20	0	1	0	.5	60	60.6	59.79381	57.4
1000	10	0	1	0	.5	38.9	40	39.00415	36
5000	10	1	1	0	.5	24.4	42.5	42.4	39.7
2000	4	1	1	0	.5	19.7	33.4	32.999	27.3
1000	2	1	1	0	.5	32.5	40.1	39.67705	30.3
5000	20	1	1	0	.5	37.4	64.3	63.9	61.9
2000	8	1	1	0	.5	19.4	37.9	37.5	33.1
1000	4	1	1	0	.5	19.5	32.3	31.99195	26.3
5000	50	1	1	0	.5	74.8	93.1	92.9	92.8
2000	20	1	1	0	.5	34.4	61.9	61.2	58.5
1000	10	1	1	0	.5	21.4	42	41.1	37.7
5000	10	.5	.5	0	.5	41.7	66	65.2	62.1
2000	4	.5	.5	0	.5	31.6	47.7	47.47984	39.9
1000	2	.5	.5	0	.5	36.13614	46.8	45.52402	35.7
5000	20	.5	.5	0	.5	65	89.8	89.4	88.7
2000	8	.5	.5	0	.5	32.1	55.1	54	50
1000	4	.5	.5	0	.5	30.5	45.5	44.24242	35.8
5000	50	.5	.5	0	.5	97.9	99.8	99.8	99.8
2000	20	.5	.5	0	.5	62.2	86.3	84.9	83.9
1000	10	.5	.5	0	.5	35.1	62.1	60.8	57.5
5000	10	2	2	0	.5	12.3	25.8	25.7	23.8
2000	4	2	2	0	.5	14	26	25.62814	20.6
1000	2	2	2	0	.5	29.1	37.4	40.02541	29.2
5000	20	2	2	0	.5	16.2	36.3	35.8	34.2
2000	8	2	2	0	.5	10	24.4	24.2	20.7
1000	4	2	2	0	.5	15.5	25.3	24.92432	19.7
5000	50	2	2	0	.5	43	71.9	71.5	71.1
2000	20	2	2	0	.5	14.8	39.2	38.5	36
1000	10	2	2	0	.5	11	25.2	24.9	22.5

SE of power

ssl	ssh	tsq0	tsq1	outc	beta	model1	model2	model3	model4
5000	10	0	0	0	.5	0	0	0	0
2000	4	0	0	0	.5	0	0	0	0
1000	2	0	0	0	.5	.09995	0	.1739874	.3146427
5000	20	0	0	0	.5	0	0	0	0
2000	8	0	0	0	.5	0	0	0	0
1000	4	0	0	0	.5	0	0	0	0
5000	50	0	0	0	.5	0	0	0	0
2000	20	0	0	0	.5	0	0	0	0
1000	10	0	0	0	.5	0	0	0	0
5000	10	1	0	0	.5	1.523299	0	0	0
2000	4	1	0	0	.5	1.412002	0	0	0
1000	2	1	0	0	.5	1.558666	.09995	.1803274	.2986469
5000	20	1	0	0	.5	1.38185	0	0	0
2000	8	1	0	0	.5	1.494761	0	0	0
1000	4	1	0	0	.5	1.385348	0	0	0

5000	50	1	0	0	.5	0	0	0	0
2000	20	1	0	0	.5	1.32671	0	0	0
1000	10	1	0	0	.5	1.425987	0	0	0
5000	10	0	1	0	.5	1.560769	1.560769	1.596509	1.542401
2000	4	0	1	0	.5	1.483479	1.488083	1.532187	1.385348
1000	2	0	1	0	.5	1.54451	1.547889	1.586493	1.44496
5000	20	0	1	0	.5	1.54451	1.541684	1.587392	1.555313
2000	8	0	1	0	.5	1.541684	1.545882	1.576125	1.506307
1000	4	0	1	0	.5	1.490339	1.497999	1.52979	1.408797
5000	50	0	1	0	.5	.7568289	.7568289	.7976043	.7739767
2000	20	0	1	0	.5	1.549193	1.545199	1.574304	1.563726
1000	10	0	1	0	.5	1.541684	1.549193	1.570967	1.517893
5000	10	1	1	0	.5	1.358175	1.56325	1.562767	1.547227
2000	4	1	1	0	.5	1.25774	1.491456	1.489167	1.408797
1000	2	1	1	0	.5	1.481131	1.549835	1.661504	1.453241
5000	20	1	1	0	.5	1.530111	1.515094	1.518812	1.535705
2000	8	1	1	0	.5	1.250456	1.534141	1.530931	1.488083
1000	4	1	1	0	.5	1.252897	1.478753	1.479474	1.392232
5000	50	1	1	0	.5	1.372938	.8014924	.8121515	.8174105
2000	20	1	1	0	.5	1.502212	1.535705	1.540961	1.558124
1000	10	1	1	0	.5	1.296935	1.560769	1.555889	1.53255
5000	10	.5	.5	0	.5	1.559202	1.497999	1.506307	1.534141
2000	4	.5	.5	0	.5	1.470184	1.579465	1.585484	1.548544
1000	2	.5	.5	0	.5	1.519902	1.577897	1.645414	1.515094
5000	20	.5	.5	0	.5	1.50831	.957058	.973468	1.001154
2000	8	.5	.5	0	.5	1.476343	1.572892	1.576071	1.581139
1000	4	.5	.5	0	.5	1.455936	1.574722	1.578533	1.516034
5000	50	.5	.5	0	.5	.4534203	.1412799	.1412799	.1412799
2000	20	.5	.5	0	.5	1.533349	1.087341	1.13225	1.162235
1000	10	.5	.5	0	.5	1.509301	1.534141	1.543813	1.56325
5000	10	2	2	0	.5	1.03861	1.383604	1.38185	1.346685
2000	4	2	2	0	.5	1.097269	1.387083	1.384049	1.278921
1000	2	2	2	0	.5	1.436381	1.530111	1.746482	1.437832
5000	20	2	2	0	.5	1.165144	1.520628	1.516034	1.50012
2000	8	2	2	0	.5	.9486833	1.358175	1.354385	1.281214
1000	4	2	2	0	.5	1.144443	1.37474	1.374119	1.25774
5000	50	2	2	0	.5	1.565567	1.421404	1.427498	1.433454
2000	20	2	2	0	.5	1.122925	1.543813	1.538749	1.517893
1000	10	2	2	0	.5	.9894443	1.372938	1.367476	1.320511

Model 1: Fixed common intercept; random treatment effect; Fixed effect for baseline
Model 2: Fixed study-specific intercepts; random treatment effect; Fixed study-specific effects for baseline
Model 3: Random study intercept; random treatment effect; fixed study-specific effects for baseline
Model 4: two-stage IPD with ipdmetan

ss1: size for lower level unit (patients)
ssh: size for higher level unit (studies)
tsq0: between study variance for the intercept
tsq1: between study variance for the exposure
outc: outcome type (0=continuous)
beta: true effect size

Publication bias=20%, main effect

Convergence

	ssl	ssh	tsq0	tsq1	outc	beta	model1	model2	model3	model4
	5000	10	0	0	0	.5	99	99.9	99	100
	2000	4	0	0	0	.5	100	100	98.4	100
	1000	2	0	0	0	.5	100	100	100	100
	5000	20	0	0	0	.5	99	100	98.6	100
	2000	8	0	0	0	.5	99.9	99.9	98	100
	1000	4	0	0	0	.5	99.9	100	98.6	100
	5000	50	0	0	0	.5	98	100	98.5	100
	2000	20	0	0	0	.5	99.3	100	98.7	100
	1000	10	0	0	0	.5	99.5	100	98.4	100
	5000	10	1	0	0	.5	100	99.9	96.2	100
	2000	4	1	0	0	.5	100	100	96.1	100
	1000	2	1	0	0	.5	100	100	100	100
	5000	20	1	0	0	.5	100	100	95.3	100
	2000	8	1	0	0	.5	100	100	97.2	100
	1000	4	1	0	0	.5	100	100	96.2	100
	5000	50	1	0	0	.5	100	100	96	100
	2000	20	1	0	0	.5	100	100	95.9	100
	1000	10	1	0	0	.5	100	100	96.1	100
	5000	10	0	1	0	.5	100	100	95.4	100
	2000	4	0	1	0	.5	100	100	95.8	100
	1000	2	0	1	0	.5	100	100	100	100
	5000	20	0	1	0	.5	100	100	96	100
	2000	8	0	1	0	.5	100	100	95.9	100
	1000	4	0	1	0	.5	100	100	95.2	100
	5000	50	0	1	0	.5	100	100	96.3	100
	2000	20	0	1	0	.5	100	100	97.1	100
	1000	10	0	1	0	.5	100	100	96.4	100
	5000	10	1	1	0	.5	100	100	100	100
	2000	4	1	1	0	.5	100	100	96.3	100
	1000	2	1	1	0	.5	100	100	100	100
	5000	20	1	1	0	.5	100	100	100	100
	2000	8	1	1	0	.5	100	100	100	100
	1000	4	1	1	0	.5	100	100	97.1	100
	5000	50	1	1	0	.5	100	100	100	100
	2000	20	1	1	0	.5	100	100	100	100
	1000	10	1	1	0	.5	100	100	100	100
	5000	10	.5	.5	0	.5	100	100	100	100
	2000	4	.5	.5	0	.5	100	100	97.1	100
	1000	2	.5	.5	0	.5	100	100	100	100
	5000	20	.5	.5	0	.5	100	100	100	100
	2000	8	.5	.5	0	.5	100	100	99.7	100
	1000	4	.5	.5	0	.5	100	100	97.4	100
	5000	50	.5	.5	0	.5	100	100	100	100
	2000	20	.5	.5	0	.5	100	100	100	100
	1000	10	.5	.5	0	.5	100	100	99.9	100
	5000	10	2	2	0	.5	100	100	100	100
	2000	4	2	2	0	.5	100	100	96.7	100
	1000	2	2	2	0	.5	100	100	100	100
	5000	20	2	2	0	.5	100	100	100	100
	2000	8	2	2	0	.5	100	100	99.9	100
	1000	4	2	2	0	.5	100	100	96.4	100
	5000	50	2	2	0	.5	100	100	100	100
	2000	20	2	2	0	.5	100	100	100	100
	1000	10	2	2	0	.5	100	100	100	100

SE of convergence

	ssl	ssh	tsq0	tsq1	outc	beta	model1	model2	model3	model4
	5000	10	0	0	0	.5	.3146427	.09995	.3146427	0
	2000	4	0	0	0	.5	0	0	.3967871	0
	1000	2	0	0	0	.5	0	0	0	0
	5000	20	0	0	0	.5	.3146427	0	.3715373	0
	2000	8	0	0	0	.5	.09995	.09995	.4427189	0
	1000	4	0	0	0	.5	.09995	0	.3715373	0
	5000	50	0	0	0	.5	.4427189	0	.3843826	0
	2000	20	0	0	0	.5	.2636475	0	.3582039	0
	1000	10	0	0	0	.5	.2230471	0	.3967871	0
	5000	10	1	0	0	.5	0	.09995	.6046156	0
	2000	4	1	0	0	.5	0	0	.6122009	0
	1000	2	1	0	0	.5	0	0	0	0
	5000	20	1	0	0	.5	0	0	.6692608	0
	2000	8	1	0	0	.5	0	0	.5216896	0
	1000	4	1	0	0	.5	0	0	.6046156	0
	5000	50	1	0	0	.5	0	0	.6196773	0
	2000	20	1	0	0	.5	0	0	.6270486	0
	1000	10	1	0	0	.5	0	0	.6122009	0
	5000	10	0	1	0	.5	0	0	.66245	0
	2000	4	0	1	0	.5	0	0	.6343185	0
	1000	2	0	1	0	.5	0	0	0	0
	5000	20	0	1	0	.5	0	0	.6196773	0

	ssl	ssh	tsq0	tsq1	outc	beta	model1	model2	model3	model4
	2000	8	0	1	0	.5	0	0	.6270486	0
	1000	4	0	1	0	.5	0	0	.6759882	0
	5000	50	0	1	0	.5	0	0	.5969171	0
	2000	20	0	1	0	.5	0	0	.5306505	0
	1000	10	0	1	0	.5	0	0	.589101	0
	5000	10	1	1	0	.5	0	0	0	0
	2000	4	1	1	0	.5	0	0	.5969171	0
	1000	2	1	1	0	.5	0	0	0	0
	5000	20	1	1	0	.5	0	0	0	0
	2000	8	1	1	0	.5	0	0	0	0
	1000	4	1	1	0	.5	0	0	.5306505	0
	5000	50	1	1	0	.5	0	0	0	0
	2000	20	1	1	0	.5	0	0	0	0
	1000	10	1	1	0	.5	0	0	0	0
	5000	10	.5	.5	0	.5	0	0	0	0
	2000	4	.5	.5	0	.5	0	0	.5306505	0
	1000	2	.5	.5	0	.5	0	0	0	0
	5000	20	.5	.5	0	.5	0	0	0	0
	2000	8	.5	.5	0	.5	0	0	.1729451	0
	1000	4	.5	.5	0	.5	0	0	.5032296	0
	5000	50	.5	.5	0	.5	0	0	0	0
	2000	20	.5	.5	0	.5	0	0	0	0
	1000	10	.5	.5	0	.5	0	0	.09995	0
	5000	10	2	2	0	.5	0	0	0	0
	2000	4	2	2	0	.5	0	0	.5648982	0
	1000	2	2	2	0	.5	0	0	0	0
	5000	20	2	2	0	.5	0	0	0	0
	2000	8	2	2	0	.5	0	0	.09995	0
	1000	4	2	2	0	.5	0	0	.589101	0
	5000	50	2	2	0	.5	0	0	0	0
	2000	20	2	2	0	.5	0	0	0	0
	1000	10	2	2	0	.5	0	0	0	0

Coverage

	ssl	ssh	tsq0	tsq1	outc	beta	model1	model2	model3	model4
	5000	10	0	0	0	.5	93.23232	92.79279	93.73737	93.3
	2000	4	0	0	0	.5	96	96	96.95122	97.2
	1000	2	0	0	0	.5	94.5	94.5	94.5	94.7
	5000	20	0	0	0	.5	91.0101	90.4	91.37931	91.1
	2000	8	0	0	0	.5	93.19319	92.99299	94.4898	93.7
	1000	4	0	0	0	.5	96.0961	95.9	96.14604	97.4
	5000	50	0	0	0	.5	78.67347	77	78.37563	76.9
	2000	20	0	0	0	.5	90.13092	89.8	90.88146	90.5
	1000	10	0	0	0	.5	93.66834	92.8	94.30894	94.1
	5000	10	1	0	0	.5	100	92.99299	94.59459	94.6
	2000	4	1	0	0	.5	100	94.7	95.21332	96
	1000	2	1	0	0	.5	93.5	93.5	93.5	93.6
	5000	20	1	0	0	.5	100	92.4	93.28437	92.8
	2000	8	1	0	0	.5	100	93	93.93004	94.1
	1000	4	1	0	0	.5	99.7	93.4	95.32225	94.3
	5000	50	1	0	0	.5	100	77.5	78.75	77.4
	2000	20	1	0	0	.5	100	89.3	91.24088	90.1
	1000	10	1	0	0	.5	100	92.2	93.7565	93
	5000	10	0	1	0	.5	82.1	81.7	81.97065	85.2
	2000	4	0	1	0	.5	69.4	68.2	67.84969	76.3
	1000	2	0	1	0	.5	16.6	16.6	16.6	16.7
	5000	20	0	1	0	.5	78.2	76.9	77.8125	80.2
	2000	8	0	1	0	.5	77.9	77.2	77.58081	81.4
	1000	4	0	1	0	.5	69.5	68.8	69.32773	76.6
	5000	50	0	1	0	.5	63.7	62.7	63.23988	64.9
	2000	20	0	1	0	.5	79.4	78.5	78.78476	80.7
	1000	10	0	1	0	.5	82.4	81.3	81.95021	85.2
	5000	10	1	1	0	.5	94.2	82.3	82.4	85.2
	2000	4	1	1	0	.5	82.7	67	67.08204	75.7
	1000	2	1	1	0	.5	17	17	17	17
	5000	20	1	1	0	.5	94.7	79.4	79.6	81.2
	2000	8	1	1	0	.5	92	81.7	81.9	85.5
	1000	4	1	1	0	.5	85.2	71.6	71.88465	78.7
	5000	50	1	1	0	.5	86.8	62.1	62.7	63.3
	2000	20	1	1	0	.5	94	78.5	79	80.7
	1000	10	1	1	0	.5	93.7	81.1	82.1	84.5
	5000	10	.5	.5	0	.5	92.6	83	83.1	85.7
	2000	4	.5	.5	0	.5	80	67.2	69.207	75.6
	1000	2	.5	.5	0	.5	23.3	23.3	23.3	23.3
	5000	20	.5	.5	0	.5	93.4	79.3	79.8	81.9
	2000	8	.5	.5	0	.5	91.1	79.8	81.04313	83.1
	1000	4	.5	.5	0	.5	83.7	67.9	70.1232	76.3
	5000	50	.5	.5	0	.5	87.7	63.4	65	65.7
	2000	20	.5	.5	0	.5	93.4	77	78.5	80
	1000	10	.5	.5	0	.5	94.4	80.7	83.28328	86.2
	5000	10	2	2	0	.5	93.2	83.6	83.8	86
	2000	4	2	2	0	.5	82.7	69.8	70.73423	76.6
	1000	2	2	2	0	.5	10.9	10.9	10.9	11
	5000	20	2	2	0	.5	94.2	81.9	82.1	83.2
	2000	8	2	2	0	.5	91.2	78.1	78.27828	82.9
	1000	4	2	2	0	.5	82.3	70.4	71.36929	77.6
	5000	50	2	2	0	.5	86.7	59.4	59.9	60.4
	2000	20	2	2	0	.5	94.3	81.3	81.8	84
	1000	10	2	2	0	.5	94.9	82.9	83	85.7

SE of coverage

ssl	ssh	tsq0	tsq1	outc	beta	model1	model2	model3	model4
5000	10	0	0	0	.5	.7983355	.818197	.7700467	.790639
2000	4	0	0	0	.5	.6196773	.6196773	.5480777	.5216896
1000	2	0	0	0	.5	.7209369	.7209369	.7209369	.7084561
5000	20	0	0	0	.5	.9090852	.9315793	.8938332	.9004388
2000	8	0	0	0	.5	.7968582	.8076229	.7288921	.7683163
1000	4	0	0	0	.5	.6128013	.6270486	.6130284	.5032296
5000	50	0	0	0	.5	1.308462	1.330789	1.311729	1.332813
2000	20	0	0	0	.5	.9464566	.957058	.9163086	.927227
1000	10	0	0	0	.5	.7720468	.8174105	.7385423	.7451107
5000	10	1	0	0	.5	0	.8076229	.7290541	.7147307
2000	4	1	0	0	.5	0	.7084561	.6886592	.6196773
1000	2	1	0	0	.5	.7795832	.7795832	.7795832	.7739767
5000	20	1	0	0	.5	0	.8379976	.8107772	.8174105
2000	8	1	0	0	.5	0	.8068457	.7658822	.7451107
1000	4	1	0	0	.5	.1729451	.7851369	.6808137	.7331507
5000	50	1	0	0	.5	0	1.320511	1.320289	1.322588
2000	20	1	0	0	.5	0	.9775019	.9128843	.9444522
1000	10	1	0	0	.5	0	.848033	.7804641	.8068457
5000	10	0	1	0	.5	1.212266	1.222747	1.244644	1.122925
2000	4	0	1	0	.5	1.457271	1.472671	1.508981	1.344734
1000	2	0	1	0	.5	1.176622	1.176622	1.176622	1.179453
5000	20	0	1	0	.5	1.305665	1.332813	1.341045	1.260143
2000	8	0	1	0	.5	1.312094	1.32671	1.346721	1.230463
1000	4	0	1	0	.5	1.455936	1.465114	1.494541	1.33882
5000	50	0	1	0	.5	1.520628	1.529284	1.553713	1.509301
2000	20	0	1	0	.5	1.278921	1.299134	1.312005	1.248002
1000	10	0	1	0	.5	1.204259	1.233009	1.238718	1.122925
5000	10	1	1	0	.5	.7391617	1.206942	1.204259	1.122925
2000	4	1	1	0	.5	1.196123	1.486943	1.514281	1.356285
1000	2	1	1	0	.5	1.187855	1.187855	1.187855	1.187855
5000	20	1	1	0	.5	.7084561	1.278921	1.2743	1.23554
2000	8	1	1	0	.5	.8579044	1.222747	1.217534	1.113441
1000	4	1	1	0	.5	1.122925	1.425987	1.442714	1.294724
5000	50	1	1	0	.5	1.070402	1.534141	1.529284	1.524175
2000	20	1	1	0	.5	.7509993	1.299134	1.288022	1.248002
1000	10	1	1	0	.5	.7683163	1.238059	1.212266	1.144443
5000	10	.5	.5	0	.5	.8277922	1.187855	1.18507	1.107028
2000	4	.5	.5	0	.5	1.264911	1.484641	1.481465	1.358175
1000	2	.5	.5	0	.5	1.336828	1.336828	1.336828	1.336828
5000	20	.5	.5	0	.5	.7851369	1.281214	1.26963	1.217534
2000	8	.5	.5	0	.5	.9004388	1.26963	1.241349	1.18507
1000	4	.5	.5	0	.5	1.168037	1.476343	1.466623	1.344734
5000	50	.5	.5	0	.5	1.03861	1.523299	1.50831	1.50117
2000	20	.5	.5	0	.5	.7851369	1.330789	1.299134	1.264911
1000	10	.5	.5	0	.5	.7270763	1.248002	1.180515	1.09067
5000	10	2	2	0	.5	.7960904	1.170914	1.165144	1.097269
2000	4	2	2	0	.5	1.196123	1.451882	1.463125	1.33882
1000	2	2	2	0	.5	.9854897	.9854897	.9854897	.9894443
5000	20	2	2	0	.5	.7391617	1.217534	1.212266	1.182269
2000	8	2	2	0	.5	.8958571	1.307819	1.304623	1.190626
1000	4	2	2	0	.5	1.206942	1.443551	1.455906	1.318423
5000	50	2	2	0	.5	1.07383	1.552946	1.549835	1.546557
2000	20	2	2	0	.5	.7331507	1.233009	1.220148	1.15931
1000	10	2	2	0	.5	.6956939	1.190626	1.187855	1.107028

Mean error

ssl	ssh	tsq0	tsq1	outc	beta	model1	model2	model3	model4
5000	10	0	0	0	.5	.0255371	.0253929	.0260404	.0260599
2000	4	0	0	0	.5	.0399606	.0398309	.0404562	.0411814
1000	2	0	0	0	.5	.0863533	.0863533	.0863533	.0863533
5000	20	0	0	0	.5	.0284429	.028319	.0288721	.0290502
2000	8	0	0	0	.5	.0415784	.0413724	.0422077	.0428173
1000	4	0	0	0	.5	.0556471	.0551203	.055911	.0566454
5000	50	0	0	0	.5	.0407616	.0406704	.0413084	.0415721
2000	20	0	0	0	.5	.0463022	.046245	.0468331	.0475417
1000	10	0	0	0	.5	.0590861	.0591454	.0599064	.0604801
5000	10	1	0	0	.5	.1319996	.0256407	.0266534	.0260468
2000	4	1	0	0	.5	.1992593	.0396111	.0431627	.0406726
1000	2	1	0	0	.5	.0891516	.0891516	.0891516	.0891516
5000	20	1	0	0	.5	.0838765	.0278376	.0287349	.0286932
2000	8	1	0	0	.5	.1205872	.0411472	.0426142	.0420146
1000	4	1	0	0	.5	.1616919	.0593977	.0637389	.0610635
5000	50	1	0	0	.5	.0534337	.0412441	.0418555	.042252
2000	20	1	0	0	.5	.0718626	.0477364	.048428	.0485561
1000	10	1	0	0	.5	.0947192	.0615908	.0627938	.0628859
5000	10	0	1	0	.5	.32091	.320594	.3200914	.3207843
2000	4	0	1	0	.5	.4771539	.4765647	.4775047	.4766317
1000	2	0	1	0	.5	.7997716	.7997716	.7997716	.7997716
5000	20	0	1	0	.5	.269383	.2692601	.2690519	.26943
2000	8	0	1	0	.5	.3735859	.3727994	.3697718	.3731388
1000	4	0	1	0	.5	.4811564	.4808015	.484347	.4813514
5000	50	0	1	0	.5	.2459849	.2464272	.2465158	.246577
2000	20	0	1	0	.5	.2735747	.2732874	.2737949	.2735021
1000	10	0	1	0	.5	.3215798	.3219871	.3199889	.3222098
5000	10	1	1	0	.5	.3464324	.3220457	.3222085	.3222055
2000	4	1	1	0	.5	.5285225	.4960435	.5010576	.4964726
1000	2	1	1	0	.5	.792684	.792684	.792684	.792684
5000	20	1	1	0	.5	.2814536	.2702902	.2704132	.2704616
2000	8	1	1	0	.5	.3742302	.357187	.3570509	.3575566

1000	4	1	1	0	.5	.5168818	.4794611	.4826622	.4802719
5000	50	1	1	0	.5	.2472166	.2451791	.2452371	.2452399
2000	20	1	1	0	.5	.2686216	.2671359	.2671217	.2674234
1000	10	1	1	0	.5	.342122	.3385743	.3385774	.338903
5000	10	.5	.5	0	.5	.2447904	.2303323	.2304778	.2307868
2000	4	.5	.5	0	.5	.3773159	.3609764	.3620319	.3610908
1000	2	.5	.5	0	.5	.5553067	.5553067	.5553067	.5553067
5000	20	.5	.5	0	.5	.1934796	.1870934	.187139	.1874201
2000	8	.5	.5	0	.5	.2746812	.2623981	.2624713	.2630436
1000	4	.5	.5	0	.5	.3619865	.341562	.3431216	.342649
5000	50	.5	.5	0	.5	.1753712	.173163	.1733742	.1734884
2000	20	.5	.5	0	.5	.1968781	.1961537	.1963147	.1966632
1000	10	.5	.5	0	.5	.2317147	.226787	.2266682	.2272582
5000	10	2	2	0	.5	.4770222	.4448799	.444808	.4449585
2000	4	2	2	0	.5	.7182094	.6834493	.6812387	.6836311
1000	2	2	2	0	.5	1.14911	1.14911	1.14911	1.14911
5000	20	2	2	0	.5	.3787812	.3662055	.3661875	.366313
2000	8	2	2	0	.5	.5452915	.5299332	.5304506	.530105
1000	4	2	2	0	.5	.729628	.6833724	.6829831	.6838096
5000	50	2	2	0	.5	.3593336	.3566494	.3566529	.3567091
2000	20	2	2	0	.5	.3759067	.3697332	.3697422	.3698665
1000	10	2	2	0	.5	.4638547	.4541443	.4541029	.4542912

SE of mean error

ssl	ssh	tsq0	tsq1	outc	beta	model1	model2	model3	model4
5000	10	0	0	0	.5	.0000195	.0000194	.0000199	.0000198
2000	4	0	0	0	.5	.0000305	.0000301	.0000319	.0000329
1000	2	0	0	0	.5	.0000787	.0000787	.0000787	.0000787
5000	20	0	0	0	.5	.0000219	.0000215	.0000223	.0000221
2000	8	0	0	0	.5	.0000322	.0000319	.0000329	.0000324
1000	4	0	0	0	.5	.0000413	.0000413	.0000429	.0000434
5000	50	0	0	0	.5	.0000261	.0000255	.0000262	.000026
2000	20	0	0	0	.5	.0000347	.0000347	.0000355	.0000352
1000	10	0	0	0	.5	.0000452	.0000451	.0000464	.0000459
5000	10	1	0	0	.5	.0001008	.000019	.0000204	.0000195
2000	4	1	0	0	.5	.0001925	.0000319	.0000368	.0000331
1000	2	1	0	0	.5	.0000807	.0000807	.0000807	.0000807
5000	20	1	0	0	.5	.0000637	.00002	.0000213	.0000205
2000	8	1	0	0	.5	.0001	.0000314	.0000331	.0000319
1000	4	1	0	0	.5	.0001495	.0000449	.0000498	.0000465
5000	50	1	0	0	.5	.0000374	.0000254	.0000264	.0000255
2000	20	1	0	0	.5	.0000546	.0000336	.0000356	.0000343
1000	10	1	0	0	.5	.0000691	.0000454	.0000475	.0000468
5000	10	0	1	0	.5	.0002336	.0002336	.0002469	.0002337
2000	4	0	1	0	.5	.0003545	.0003534	.0003707	.0003536
1000	2	0	1	0	.5	.0006012	.0006012	.0006012	.0006012
5000	20	0	1	0	.5	.0001916	.0001922	.0002008	.0001923
2000	8	0	1	0	.5	.0002674	.000267	.0002759	.0002671
1000	4	0	1	0	.5	.0003619	.0003619	.0003826	.0003619
5000	50	0	1	0	.5	.000139	.0001391	.000145	.0001392
2000	20	0	1	0	.5	.0001937	.000194	.0001998	.000194
1000	10	0	1	0	.5	.000255	.0002551	.0002625	.0002552
5000	10	1	1	0	.5	.0002439	.0002294	.0002292	.0002295
2000	4	1	1	0	.5	.0003884	.0003534	.0003681	.000353
1000	2	1	1	0	.5	.0006051	.0006051	.0006051	.0006051
5000	20	1	1	0	.5	.0001931	.0001813	.0001813	.0001814
2000	8	1	1	0	.5	.0002846	.0002683	.0002685	.0002685
1000	4	1	1	0	.5	.0003821	.0003598	.0003685	.0003597
5000	50	1	1	0	.5	.000146	.0001429	.0001429	.0001429
2000	20	1	1	0	.5	.0001892	.0001847	.0001848	.0001848
1000	10	1	1	0	.5	.0002448	.0002371	.0002374	.0002374
5000	10	.5	.5	0	.5	.000177	.0001656	.0001656	.0001658
2000	4	.5	.5	0	.5	.0002925	.0002727	.0002808	.000273
1000	2	.5	.5	0	.5	.0004339	.0004339	.0004339	.0004339
5000	20	.5	.5	0	.5	.000141	.0001338	.0001338	.000134
2000	8	.5	.5	0	.5	.0002038	.0001935	.0001945	.0001939
1000	4	.5	.5	0	.5	.0002752	.0002577	.0002666	.000258
5000	50	.5	.5	0	.5	.0001029	.0001016	.0001016	.0001017
2000	20	.5	.5	0	.5	.0001404	.0001395	.0001396	.0001396
1000	10	.5	.5	0	.5	.0001727	.0001679	.000168	.000168
5000	10	2	2	0	.5	.0003514	.0003307	.0003305	.0003307
2000	4	2	2	0	.5	.0005536	.00051	.000529	.0005101
1000	2	2	2	0	.5	.00087	.00087	.00087	.00087
5000	20	2	2	0	.5	.0002785	.0002686	.0002686	.0002686
2000	8	2	2	0	.5	.0003966	.0003851	.0003853	.0003852
1000	4	2	2	0	.5	.0005337	.0005093	.0005248	.0005094
5000	50	2	2	0	.5	.0001986	.0001955	.0001955	.0001955
2000	20	2	2	0	.5	.0002681	.0002605	.0002605	.0002605
1000	10	2	2	0	.5	.0003426	.0003315	.0003316	.0003317

Mean bias

ssl	ssh	tsq0	tsq1	outc	beta	model1	model2	model3	model4
5000	10	0	0	0	.5	.0151354	.0147451	.0159598	.0158647
2000	4	0	0	0	.5	.0160924	.0158624	.0176329	.0185007
1000	2	0	0	0	.5	.0659198	.0659198	.0659198	.0659198
5000	20	0	0	0	.5	.0178536	.0174994	.0186564	.018754
2000	8	0	0	0	.5	.0231305	.0226387	.0239781	.0248656

1000	4	0	0	0	.5	.0256465	.0250041	.0266332	.0280039
5000	50	0	0	0	.5	.0378954	.0376521	.0384182	.0387315
2000	20	0	0	0	.5	.0332817	.0329369	.0339844	.0345819
1000	10	0	0	0	.5	.0347807	.0343989	.0359438	.0362605
5000	10	1	0	0	.5	.0077747	.011869	.0136481	.0129307
2000	4	1	0	0	.5	.0390828	.0175609	.0228661	.0196896
1000	2	1	0	0	.5	.0663129	.0663129	.0663129	.0663129
5000	20	1	0	0	.5	.0300217	.0195072	.0211459	.0208602
2000	8	1	0	0	.5	.0251953	.0211334	.023944	.0231526
1000	4	1	0	0	.5	.028528	.0286556	.0350451	.0312828
5000	50	1	0	0	.5	.045681	.0384258	.0391398	.0395245
2000	20	1	0	0	.5	.0467576	.0374048	.0387707	.038864
1000	10	1	0	0	.5	.0426383	.0343459	.036719	.0366465
5000	10	0	1	0	.5	.2078371	.2072536	.208068	.2076182
2000	4	0	1	0	.5	.2590446	.25745	.2620712	.2585047
1000	2	0	1	0	.5	.5745761	.5745761	.5745761	.5745761
5000	20	0	1	0	.5	.2195136	.2195417	.2189142	.2198192
2000	8	0	1	0	.5	.2368638	.2361676	.2314987	.2369342
1000	4	0	1	0	.5	.2504485	.2481699	.2551511	.250175
5000	50	0	1	0	.5	.2402214	.2405987	.2407237	.2407607
2000	20	0	1	0	.5	.2287871	.228439	.228183	.2287741
1000	10	0	1	0	.5	.2046117	.2041518	.2077339	.2047354
5000	10	1	1	0	.5	.2054418	.2062821	.2063892	.2066362
2000	4	1	1	0	.5	.2834189	.27959	.2858098	.2803813
1000	2	1	1	0	.5	.5824614	.5824614	.5824614	.5824614
5000	20	1	1	0	.5	.2276944	.2249615	.2250769	.2252501
2000	8	1	1	0	.5	.2374933	.2338931	.2339809	.2347866
1000	4	1	1	0	.5	.2314648	.2241632	.2315022	.2255146
5000	50	1	1	0	.5	.2375234	.2357522	.2358468	.2358266
2000	20	1	1	0	.5	.2276804	.2283578	.2284255	.2286882
1000	10	1	1	0	.5	.2221637	.2214073	.2217858	.2219319
5000	10	.5	.5	0	.5	.1522563	.1517521	.1518783	.1524579
2000	4	.5	.5	0	.5	.1826102	.1881547	.187836	.1896779
1000	2	.5	.5	0	.5	.3863793	.3863793	.3863793	.3863793
5000	20	.5	.5	0	.5	.1627723	.1597461	.1599109	.1602043
2000	8	.5	.5	0	.5	.18182	.1781666	.1786161	.1794881
1000	4	.5	.5	0	.5	.1776569	.174805	.1741782	.1770901
5000	50	.5	.5	0	.5	.1673608	.1659333	.1661799	.1663121
2000	20	.5	.5	0	.5	.1607499	.1609041	.1611869	.1616749
1000	10	.5	.5	0	.5	.1368687	.1362906	.1372672	.1374261
5000	10	2	2	0	.5	.2760141	.2744511	.2742518	.2747148
2000	4	2	2	0	.5	.3581582	.3646763	.35527	.3656153
1000	2	2	2	0	.5	.8404618	.8404618	.8404618	.8404618
5000	20	2	2	0	.5	.3126476	.3104755	.3105073	.310646
2000	8	2	2	0	.5	.3327775	.3415161	.3417911	.3420111
1000	4	2	2	0	.5	.3763245	.3828384	.3748832	.3838199
5000	50	2	2	0	.5	.3461361	.3433929	.3434044	.3434578
2000	20	2	2	0	.5	.3096985	.3066377	.3066608	.3068124
1000	10	2	2	0	.5	.3059975	.2996125	.2997855	.3000209

SE of mean bias

ssl	ssh	tsq0	tsq1	outc	beta	model1	model2	model3	model4
5000	10	0	0	0	.5	.0000285	.0000284	.0000288	.0000286
2000	4	0	0	0	.5	.0000476	.0000474	.0000489	.0000494
1000	2	0	0	0	.5	.0000965	.0000965	.0000965	.0000965
5000	20	0	0	0	.5	.0000313	.000031	.0000316	.0000313
2000	8	0	0	0	.5	.0000473	.0000471	.0000484	.0000476
1000	4	0	0	0	.5	.0000645	.0000642	.0000658	.0000657
5000	50	0	0	0	.5	.0000303	.0000298	.0000304	.0000301
2000	20	0	0	0	.5	.0000475	.0000475	.0000482	.000048
1000	10	0	0	0	.5	.000066	.000066	.0000673	.0000667
5000	10	1	0	0	.5	.0001659	.0000296	.0000314	.0000299
2000	4	1	0	0	.5	.0002744	.0000477	.000053	.0000486
1000	2	1	0	0	.5	.0001003	.0001003	.0001003	.0001003
5000	20	1	0	0	.5	.000101	.0000282	.0000295	.0000284
2000	8	1	0	0	.5	.0001546	.0000472	.0000491	.0000474
1000	4	1	0	0	.5	.0002184	.0000687	.0000745	.0000701
5000	50	1	0	0	.5	.0000466	.0000295	.0000306	.0000296
2000	20	1	0	0	.5	.0000772	.0000448	.0000467	.000045
1000	10	1	0	0	.5	.0001092	.0000684	.0000712	.0000693
5000	10	0	1	0	.5	.0003383	.0003383	.000355	.0003383
2000	4	0	1	0	.5	.0005351	.0005347	.0005579	.0005344
1000	2	0	1	0	.5	.0008193	.0008193	.0008193	.0008193
5000	20	0	1	0	.5	.0002472	.0002476	.0002586	.0002475
2000	8	0	1	0	.5	.0003938	.0003932	.0004082	.0003931
1000	4	0	1	0	.5	.0005477	.0005484	.0005776	.000548
5000	50	0	1	0	.5	.0001487	.000149	.0001552	.000149
2000	20	0	1	0	.5	.0002451	.0002453	.0002534	.0002452
1000	10	0	1	0	.5	.0003559	.0003565	.0003643	.0003565
5000	10	1	1	0	.5	.0003706	.0003374	.0003374	.0003374
2000	4	1	1	0	.5	.0005916	.0005412	.0005642	.000541
1000	2	1	1	0	.5	.0008097	.0008097	.0008097	.0008097
5000	20	1	1	0	.5	.0002543	.0002352	.0002353	.0002353
2000	8	1	1	0	.5	.0004059	.0003807	.0003807	.0003806
1000	4	1	1	0	.5	.0005998	.0005561	.0005712	.0005562
5000	50	1	1	0	.5	.0001613	.000158	.000158	.000158
2000	20	1	1	0	.5	.0002369	.000231	.0002309	.000231
1000	10	1	1	0	.5	.0003573	.0003491	.0003491	.0003493
5000	10	.5	.5	0	.5	.000261	.0002398	.0002398	.0002399
2000	4	.5	.5	0	.5	.0004412	.0004116	.0004249	.0004111
1000	2	.5	.5	0	.5	.0005895	.0005895	.0005895	.0005895
5000	20	.5	.5	0	.5	.0001756	.0001655	.0001654	.0001656
2000	8	.5	.5	0	.5	.0002898	.0002731	.000274	.0002731
1000	4	.5	.5	0	.5	.0004187	.0003907	.0004041	.0003908

5000	50	.5	.5	0	.5	.0001155	.0001131	.000113	.000113
2000	20	.5	.5	0	.5	.0001807	.000179	.0001791	.000179
1000	10	.5	.5	0	.5	.0002546	.0002472	.0002467	.000247
5000	10	2	2	0	.5	.0005244	.0004817	.0004816	.0004817
2000	4	2	2	0	.5	.0008333	.0007711	.0008009	.0007709
1000	2	2	2	0	.5	.0011712	.0011712	.0011712	.0011712
5000	20	2	2	0	.5	.0003512	.0003315	.0003315	.0003315
2000	8	2	2	0	.5	.0005866	.0005592	.0005599	.0005591
1000	4	2	2	0	.5	.0008222	.0007617	.0007915	.0007617
5000	50	2	2	0	.5	.0002208	.000218	.0002179	.0002179
2000	20	2	2	0	.5	.0003425	.0003326	.0003325	.0003325
1000	10	2	2	0	.5	.0004889	.0004759	.0004759	.0004759

Power

ssl	ssh	tsq0	tsq1	outc	beta	model1	model2	model3	model4
5000	10	0	0	0	.5	100	100	100	100
2000	4	0	0	0	.5	100	100	100	100
1000	2	0	0	0	.5	99.8	99.8	99.8	99.8
5000	20	0	0	0	.5	100	100	100	100
2000	8	0	0	0	.5	100	100	100	100
1000	4	0	0	0	.5	100	100	100	100
5000	50	0	0	0	.5	100	100	100	100
2000	20	0	0	0	.5	100	100	100	100
1000	10	0	0	0	.5	100	100	100	100
5000	10	1	0	0	.5	31.6	100	100	100
2000	4	1	0	0	.5	30.7	100	100	100
1000	2	1	0	0	.5	99.8	99.8	99.8	99.7
5000	20	1	0	0	.5	68.3	100	100	100
2000	8	1	0	0	.5	29.6	100	100	100
1000	4	1	0	0	.5	31.2	100	100	100
5000	50	1	0	0	.5	100	100	100	100
2000	20	1	0	0	.5	74.5	100	100	100
1000	10	1	0	0	.5	29.3	100	100	100
5000	10	0	1	0	.5	63.6	63.1	63.41719	58.5
2000	4	0	1	0	.5	51.4	51.7	51.77453	42.2
1000	2	0	1	0	.5	86.1	86.1	86.1	86
5000	20	0	1	0	.5	85.4	85.6	85.3125	83.7
2000	8	0	1	0	.5	58.1	58.7	58.39416	53.7
1000	4	0	1	0	.5	51.9	52.6	53.57143	44.4
5000	50	0	1	0	.5	100	100	100	99.9
2000	20	0	1	0	.5	86.9	87.3	86.81771	85.5
1000	10	0	1	0	.5	60.5	61.6	60.78838	56.7
5000	10	1	1	0	.5	36.7	62.7	62.6	59.6
2000	4	1	1	0	.5	36	53.5	53.79024	45.4
1000	2	1	1	0	.5	86.4	86.4	86.4	86.3
5000	20	1	1	0	.5	64.1	88	88	86.8
2000	8	1	1	0	.5	31.2	57.3	57.2	51.7
1000	4	1	1	0	.5	31.2	50.3	50.15448	39.9
5000	50	1	1	0	.5	97.2	100	99.9	99.9
2000	20	1	1	0	.5	62	87.2	86.9	85.7
1000	10	1	1	0	.5	36.9	63.6	62.7	59.3
5000	10	.5	.5	0	.5	58.4	81.1	80.9	78
2000	4	.5	.5	0	.5	45.9	62.9	62.82183	54
1000	2	.5	.5	0	.5	87.8	87.8	87.8	87.7
5000	20	.5	.5	0	.5	86	97.9	97.7	96.9
2000	8	.5	.5	0	.5	49.5	72.5	72.11635	67.1
1000	4	.5	.5	0	.5	44.8	62.1	60.47228	53.3
5000	50	.5	.5	0	.5	100	100	100	100
2000	20	.5	.5	0	.5	83.4	96.6	96.3	95.5
1000	10	.5	.5	0	.5	49.9	77.2	76.27628	72.7
5000	10	2	2	0	.5	22.4	44.9	44.8	41.5
2000	4	2	2	0	.5	27.2	43.1	42.08893	35.7
1000	2	2	2	0	.5	83.8	83.8	83.8	83.8
5000	20	2	2	0	.5	39.3	69.7	69.5	67.2
2000	8	2	2	0	.5	23.4	46.6	46.54655	41.6
1000	4	2	2	0	.5	27.9	41.5	41.07884	34.2
5000	50	2	2	0	.5	85.1	96.9	96.9	96.6
2000	20	2	2	0	.5	38.7	72	71.6	69.6
1000	10	2	2	0	.5	22.9	47.3	46.9	42.6

SE of power

ssl	ssh	tsq0	tsq1	outc	beta	model1	model2	model3	model4
5000	10	0	0	0	.5	0	0	0	0
2000	4	0	0	0	.5	0	0	0	0
1000	2	0	0	0	.5	.1412799	.1412799	.1412799	.1412799
5000	20	0	0	0	.5	0	0	0	0
2000	8	0	0	0	.5	0	0	0	0
1000	4	0	0	0	.5	0	0	0	0
5000	50	0	0	0	.5	0	0	0	0
2000	20	0	0	0	.5	0	0	0	0
1000	10	0	0	0	.5	0	0	0	0
5000	10	1	0	0	.5	1.470184	0	0	0
2000	4	1	0	0	.5	1.458599	0	0	0
1000	2	1	0	0	.5	.1412799	.1412799	.1412799	.1729451
5000	20	1	0	0	.5	1.471431	0	0	0
2000	8	1	0	0	.5	1.443551	0	0	0
1000	4	1	0	0	.5	1.465114	0	0	0

5000	50	1	0	0	.5	0	0	0	0
2000	20	1	0	0	.5	1.378314	0	0	0
1000	10	1	0	0	.5	1.439274	0	0	0
5000	10	0	1	0	.5	1.521526	1.525906	1.559437	1.558124
2000	4	0	1	0	.5	1.580519	1.580225	1.614409	1.561781
1000	2	0	1	0	.5	1.093979	1.093979	1.093979	1.097269
5000	20	0	1	0	.5	1.11662	1.110243	1.14247	1.168037
2000	8	0	1	0	.5	1.560253	1.55702	1.591668	1.576804
1000	4	0	1	0	.5	1.579997	1.579	1.61637	1.571191
5000	50	0	1	0	.5	0	0	0	.09995
2000	20	0	1	0	.5	1.066954	1.052953	1.08565	1.113441
1000	10	0	1	0	.5	1.545882	1.537999	1.572458	1.566879
5000	10	1	1	0	.5	1.524175	1.529284	1.530111	1.551722
2000	4	1	1	0	.5	1.517893	1.57726	1.606591	1.574433
1000	2	1	1	0	.5	1.083993	1.083993	1.083993	1.087341
5000	20	1	1	0	.5	1.516967	1.027619	1.027619	1.070402
2000	8	1	1	0	.5	1.465114	1.564196	1.56466	1.580225
1000	4	1	1	0	.5	1.465114	1.58111	1.604569	1.548544
5000	50	1	1	0	.5	.5216896	0	.09995	.09995
2000	20	1	1	0	.5	1.534927	1.056485	1.066954	1.107028
1000	10	1	1	0	.5	1.525906	1.521526	1.529284	1.553548
5000	10	.5	.5	0	.5	1.558666	1.238059	1.243057	1.309962
2000	4	.5	.5	0	.5	1.575814	1.527609	1.550921	1.576071
1000	2	.5	.5	0	.5	1.034969	1.034969	1.034969	1.03861
5000	20	.5	.5	0	.5	1.097269	.4534203	.4740359	.5480785
2000	8	.5	.5	0	.5	1.58106	1.412002	1.420182	1.485796
1000	4	.5	.5	0	.5	1.572565	1.534141	1.566569	1.577691
5000	50	.5	.5	0	.5	0	0	0	0
2000	20	.5	.5	0	.5	1.176622	.5730969	.5969171	.6555532
1000	10	.5	.5	0	.5	1.581136	1.32671	1.345871	1.408797
5000	10	2	2	0	.5	1.318423	1.572892	1.572565	1.558124
2000	4	2	2	0	.5	1.407182	1.566011	1.587638	1.515094
1000	2	2	2	0	.5	1.165144	1.165144	1.165144	1.165144
5000	20	2	2	0	.5	1.54451	1.453241	1.455936	1.484641
2000	8	2	2	0	.5	1.33882	1.577479	1.578152	1.558666
1000	4	2	2	0	.5	1.418305	1.558124	1.584551	1.50012
5000	50	2	2	0	.5	1.126051	.5480785	.5480785	.5730969
2000	20	2	2	0	.5	1.540231	1.419859	1.425987	1.454593
1000	10	2	2	0	.5	1.328755	1.578832	1.578097	1.563726

Model 1: Fixed common intercept; random treatment effect; Fixed effect for baseline
Model 2: Fixed study-specific intercepts; random treatment effect; Fixed study-specific effects for baseline
Model 3: Random study intercept; random treatment effect; fixed study-specific effects for baseline
Model 4: two-stage IPD with ipdmetan

ss1: size for lower level unit (patients)
ssh: size for higher level unit (studies)
tsq0: between study variance for the intercept
tsq1: between study variance for the exposure
outc: outcome type (0=continuous)
beta: true effect size

Varying residual errors, main effect

Convergence

ssl	ssh	tsq0	tsq1	outc	beta	model1	model2	model3	model4
5000	10	0	0	0	.5	98.9	99.8	98.3	100
2000	4	0	0	0	.5	99.6	100	98.2	100
1000	2	0	0	0	.5	99.9	100	99	100
5000	20	0	0	0	.5	99.4	99.9	98.9	100
2000	8	0	0	0	.5	99.5	100	98.8	100
1000	4	0	0	0	.5	99.6	99.9	98.2	100
5000	50	0	0	0	.5	98.4	100	98.9	100
2000	20	0	0	0	.5	99.5	100	98.7	100
1000	10	0	0	0	.5	99.6	100	98	100
5000	10	1	0	0	.5	100	100	96.6	100
2000	4	1	0	0	.5	100	99.9	96.8	100
1000	2	1	0	0	.5	100	100	95.7	100
5000	20	1	0	0	.5	100	99.9	96.4	100
2000	8	1	0	0	.5	100	99.9	97.3	100
1000	4	1	0	0	.5	100	99.9	96.8	100
5000	50	1	0	0	.5	100	100	98.5	100
2000	20	1	0	0	.5	100	100	98	100
1000	10	1	0	0	.5	100	100	97.4	100
5000	10	0	1	0	.5	100	100	95.8	100
2000	4	0	1	0	.5	100	99.9	95.1	100
1000	2	0	1	0	.5	99.9	99.9	93.9	100
5000	20	0	1	0	.5	100	100	94.5	100
2000	8	0	1	0	.5	100	100	95.8	100
1000	4	0	1	0	.5	100	100	97.5	100
5000	50	0	1	0	.5	100	100	96.1	100
2000	20	0	1	0	.5	100	100	96.1	100
1000	10	0	1	0	.5	100	100	97.6	100
5000	10	1	1	0	.5	100	100	100	100
2000	4	1	1	0	.5	100	100	98.9	100
1000	2	1	1	0	.5	100	100	83.9	100
5000	20	1	1	0	.5	100	100	100	100
2000	8	1	1	0	.5	100	100	100	100
1000	4	1	1	0	.5	100	100	99	100
5000	50	1	1	0	.5	100	100	100	100
2000	20	1	1	0	.5	100	100	100	100
1000	10	1	1	0	.5	100	100	100	100
5000	10	.5	.5	0	.5	100	100	100	100
2000	4	.5	.5	0	.5	100	100	99.1	100
1000	2	.5	.5	0	.5	99.8	100	87.5	100
5000	20	.5	.5	0	.5	100	100	100	100
2000	8	.5	.5	0	.5	100	100	100	100
1000	4	.5	.5	0	.5	100	100	98.2	100
5000	50	.5	.5	0	.5	100	100	100	100
2000	20	.5	.5	0	.5	100	100	100	100
1000	10	.5	.5	0	.5	100	100	99.9	100
5000	10	2	2	0	.5	100	100	100	100
2000	4	2	2	0	.5	100	100	99.7	100
1000	2	2	2	0	.5	100	100	77.9	100
5000	20	2	2	0	.5	100	100	100	100
2000	8	2	2	0	.5	100	100	100	100
1000	4	2	2	0	.5	100	100	99.4	100
5000	50	2	2	0	.5	100	100	100	100
2000	20	2	2	0	.5	100	100	100	100
1000	10	2	2	0	.5	100	100	100	100

SE of convergence

ssl	ssh	tsq0	tsq1	outc	beta	model1	model2	model3	model4
5000	10	0	0	0	.5	.3298333	.1412799	.4087909	0
2000	4	0	0	0	.5	.1995996	0	.4204284	0
1000	2	0	0	0	.5	.09995	0	.3146427	0
5000	20	0	0	0	.5	.244213	.09995	.3298333	0
2000	8	0	0	0	.5	.2230471	0	.3443254	0
1000	4	0	0	0	.5	.1995996	.09995	.4204284	0
5000	50	0	0	0	.5	.3967871	0	.3298333	0
2000	20	0	0	0	.5	.2230471	0	.3582039	0
1000	10	0	0	0	.5	.1995996	0	.4427189	0
5000	10	1	0	0	.5	0	0	.5730969	0
2000	4	1	0	0	.5	0	.09995	.5565609	0
1000	2	1	0	0	.5	0	0	.6414905	0
5000	20	1	0	0	.5	0	.09995	.589101	0
2000	8	1	0	0	.5	0	.09995	.5125524	0
1000	4	1	0	0	.5	0	.09995	.5565609	0
5000	50	1	0	0	.5	0	0	.3843826	0
2000	20	1	0	0	.5	0	0	.4427189	0
1000	10	1	0	0	.5	0	0	.5032296	0
5000	10	0	1	0	.5	0	0	.6343185	0
2000	4	0	1	0	.5	0	.09995	.6826346	0
1000	2	0	1	0	.5	.09995	.09995	.7568289	0
5000	20	0	1	0	.5	0	0	.7209369	0

2000	8	0	1	0	.5	0	0	.6343185	0
1000	4	0	1	0	.5	0	0	.4937104	0
5000	50	0	1	0	.5	0	0	.6122009	0
2000	20	0	1	0	.5	0	0	.6122009	0
1000	10	0	1	0	.5	0	0	.4839835	0
5000	10	1	1	0	.5	0	0	0	0
2000	4	1	1	0	.5	0	0	.3298333	0
1000	2	1	1	0	.5	0	0	1.162235	0
5000	20	1	1	0	.5	0	0	0	0
2000	8	1	1	0	.5	0	0	0	0
1000	4	1	1	0	.5	0	0	.3146427	0
5000	50	1	1	0	.5	0	0	0	0
2000	20	1	1	0	.5	0	0	0	0
1000	10	1	1	0	.5	0	0	0	0
5000	10	.5	.5	0	.5	0	0	0	0
2000	4	.5	.5	0	.5	0	0	.2986469	0
1000	2	.5	.5	0	.5	.1412799	0	1.045825	0
5000	20	.5	.5	0	.5	0	0	0	0
2000	8	.5	.5	0	.5	0	0	0	0
1000	4	.5	.5	0	.5	0	0	.4204284	0
5000	50	.5	.5	0	.5	0	0	0	0
2000	20	.5	.5	0	.5	0	0	0	0
1000	10	.5	.5	0	.5	0	0	.09995	0
5000	10	2	2	0	.5	0	0	0	0
2000	4	2	2	0	.5	0	0	.1729451	0
1000	2	2	2	0	.5	0	0	1.312094	0
5000	20	2	2	0	.5	0	0	0	0
2000	8	2	2	0	.5	0	0	0	0
1000	4	2	2	0	.5	0	0	.244213	0
5000	50	2	2	0	.5	0	0	0	0
2000	20	2	2	0	.5	0	0	0	0
1000	10	2	2	0	.5	0	0	0	0

Coverage

ssl	ssh	tsq0	tsq1	outc	beta	model1	model2	model3	model4
5000	10	0	0	0	.5	95.75329	94.98998	96.33774	95.8
2000	4	0	0	0	.5	96.18474	96.2	97.45418	95.6
1000	2	0	0	0	.5	95.6957	95.8	96.36364	96.3
5000	20	0	0	0	.5	95.17103	94.09409	96.46107	95.2
2000	8	0	0	0	.5	96.48241	96.1	97.67206	96.8
1000	4	0	0	0	.5	95.78313	95.996	97.14868	95
5000	50	0	0	0	.5	95.3252	95	95.8544	94.7
2000	20	0	0	0	.5	95.27638	94.5	96.96049	96.1
1000	10	0	0	0	.5	94.87952	95.1	96.32653	95.7
5000	10	1	0	0	.5	100	95.6	96.79089	96.4
2000	4	1	0	0	.5	99.9	97.0971	97.52066	97.2
1000	2	1	0	0	.5	98.7	95.6	98.11912	96.5
5000	20	1	0	0	.5	100	94.99499	96.6805	96.8
2000	8	1	0	0	.5	100	94.19419	96.3001	96.4
1000	4	1	0	0	.5	99.9	96.5966	97.62397	97.1
5000	50	1	0	0	.5	100	95.3	96.85279	94.8
2000	20	1	0	0	.5	100	93.8	95.40816	94.9
1000	10	1	0	0	.5	100	94.6	97.43326	97.1
5000	10	0	1	0	.5	89.3	89.4	89.24843	91
2000	4	0	1	0	.5	81.5	80.88088	80.86225	86.3
1000	2	0	1	0	.5	62.46246	60.96096	61.02236	72.2
5000	20	0	1	0	.5	92.5	92.2	92.48677	92.6
2000	8	0	1	0	.5	88.6	87.7	88.30898	90.2
1000	4	0	1	0	.5	83.6	81.7	82.97436	85.9
5000	50	0	1	0	.5	94.1	93.9	94.06868	94.2
2000	20	0	1	0	.5	93.1	93.1	93.54839	94.1
1000	10	0	1	0	.5	90.2	89	89.85656	91.1
5000	10	1	1	0	.5	95.9	90.6	90.7	91.3
2000	4	1	1	0	.5	88.8	82.4	83.11426	85.8
1000	2	1	1	0	.5	68.4	59.8	59.59476	68.5
5000	20	1	1	0	.5	98.1	93.7	93.9	94.6
2000	8	1	1	0	.5	96.7	88.8	89.4	91.9
1000	4	1	1	0	.5	91.6	81.7	82.52525	86.3
5000	50	1	1	0	.5	99.7	93.4	93.6	94.1
2000	20	1	1	0	.5	98.7	91.9	92.6	92.9
1000	10	1	1	0	.5	97.1	90.2	90.5	92
5000	10	.5	.5	0	.5	95	89.1	89.8	91.7
2000	4	.5	.5	0	.5	88.2	80.3	81.5338	85
1000	2	.5	.5	0	.5	68.43687	60.9	62.85714	70.8
5000	20	.5	.5	0	.5	97.5	92	92.5	93.3
2000	8	.5	.5	0	.5	95.6	89.1	89.4	91.7
1000	4	.5	.5	0	.5	91.3	80.3	81.67006	85.8
5000	50	.5	.5	0	.5	99.1	93.5	93.9	94.5
2000	20	.5	.5	0	.5	98.2	91.7	92.4	93.2
1000	10	.5	.5	0	.5	96.7	87.6	88.78879	90.7
5000	10	2	2	0	.5	96.8	91	91.1	92.6
2000	4	2	2	0	.5	89.3	81.7	82.04614	86.3
1000	2	2	2	0	.5	68.4	60.8	59.69191	71.6
5000	20	2	2	0	.5	97.8	92.9	93	93.3
2000	8	2	2	0	.5	97	90.5	90.9	92.2
1000	4	2	2	0	.5	89	79.7	79.97988	85.8
5000	50	2	2	0	.5	99.1	94.3	94.3	94.4
2000	20	2	2	0	.5	97.8	93	93	93.1
1000	10	2	2	0	.5	96.8	90.3	90.5	92

SE of coverage

ssl	ssh	tsq0	tsq1	outc	beta	model1	model2	model3	model4
5000	10	0	0	0	.5	.6412173	.6905472	.5990954	.6343185
2000	4	0	0	0	.5	.6069956	.6046156	.502642	.6485677
1000	2	0	0	0	.5	.6421181	.6343185	.5949393	.5969171
5000	20	0	0	0	.5	.6799651	.7458332	.587508	.6759882
2000	8	0	0	0	.5	.5840298	.6122009	.4797248	.5565609
1000	4	0	0	0	.5	.6368099	.6202847	.5311118	.6892024
5000	50	0	0	0	.5	.6729576	.6892024	.6338721	.7084561
2000	20	0	0	0	.5	.6725406	.7209369	.546438	.6122009
1000	10	0	0	0	.5	.6984125	.6826346	.6008944	.6414905
5000	10	1	0	0	.5	0	.6485677	.5670495	.589101
2000	4	1	0	0	.5	.09995	.5311738	.4997797	.5216896
1000	2	1	0	0	.5	.3582039	.6485677	.4391381	.5811626
5000	20	1	0	0	.5	0	.6898742	.5769888	.5565609
2000	8	1	0	0	.5	0	.7398788	.6051343	.589101
1000	4	1	0	0	.5	.09995	.5736604	.4895159	.5306505
5000	50	1	0	0	.5	0	.6692608	.5562893	.7021111
2000	20	1	0	0	.5	0	.7626008	.6686101	.6956939
1000	10	1	0	0	.5	0	.7147307	.5067161	.5306505
5000	10	0	1	0	.5	.9775019	.973468	1.000814	.9049862
2000	4	0	1	0	.5	1.227905	1.244154	1.27564	1.087341
1000	2	0	1	0	.5	1.532003	1.543451	1.591547	1.416743
5000	20	0	1	0	.5	.8329166	.848033	.857506	.8277922
2000	8	0	1	0	.5	1.005007	1.03861	1.038116	.9401915
1000	4	0	1	0	.5	1.170914	1.222747	1.203709	1.100541
5000	50	0	1	0	.5	.7451107	.7568289	.7619677	.7391617
2000	20	0	1	0	.5	.8014924	.8014924	.7924842	.7451107
1000	10	0	1	0	.5	.9401915	.9894443	.9663683	.9004388
5000	10	1	1	0	.5	.6270486	.9228434	.918428	.8912407
2000	4	1	1	0	.5	.9972763	1.204259	1.191241	1.103793
1000	2	1	1	0	.5	1.470184	1.550471	1.694111	1.468928
5000	20	1	1	0	.5	.4317291	.7683163	.7568289	.7147307
2000	8	1	1	0	.5	.5648982	.9972763	.973468	.8627804
1000	4	1	1	0	.5	.8771773	1.222747	1.206928	1.087341
5000	50	1	1	0	.5	.1729451	.7851369	.7739767	.7451107
2000	20	1	1	0	.5	.3582039	.8627804	.8277922	.8121515
1000	10	1	1	0	.5	.5306505	.9401915	.927227	.8579044
5000	10	.5	.5	0	.5	.6892024	.9854897	.957058	.8724162
2000	4	.5	.5	0	.5	1.020176	1.25774	1.232596	1.129159
1000	2	.5	.5	0	.5	1.471194	1.54311	1.633469	1.437832
5000	20	.5	.5	0	.5	.4937104	.8579044	.8329166	.790639
2000	8	.5	.5	0	.5	.6485677	.9854897	.973468	.8724162
1000	4	.5	.5	0	.5	.8912407	1.25774	1.234685	1.103793
5000	50	.5	.5	0	.5	.2986469	.7795832	.7568289	.7209369
2000	20	.5	.5	0	.5	.4204284	.8724162	.8379976	.7960904
1000	10	.5	.5	0	.5	.5648982	1.042228	.9982115	.918428
5000	10	2	2	0	.5	.5565609	.9049862	.9004388	.8277922
2000	4	2	2	0	.5	.9775019	1.222747	1.215515	1.087341
1000	2	2	2	0	.5	1.470184	1.543813	1.757459	1.425987
5000	20	2	2	0	.5	.4638534	.8121515	.8068457	.790639
2000	8	2	2	0	.5	.5394442	.927227	.9094999	.848033
1000	4	2	2	0	.5	.9894443	1.271971	1.269201	1.103793
5000	50	2	2	0	.5	.2986469	.7331507	.7331507	.7270763
2000	20	2	2	0	.5	.4638534	.8068457	.8068457	.8014924
1000	10	2	2	0	.5	.5565609	.9359006	.927227	.8579044

Mean error

ssl	ssh	tsq0	tsq1	outc	beta	model1	model2	model3	model4
5000	10	0	0	0	.5	.0266785	.026622	.0268088	.0167089
2000	4	0	0	0	.5	.0399682	.0400663	.0408984	.0311978
1000	2	0	0	0	.5	.058111	.0583257	.0613117	.0556491
5000	20	0	0	0	.5	.0252179	.0252405	.0254926	.0136016
2000	8	0	0	0	.5	.0385117	.0381876	.0386837	.0259
1000	4	0	0	0	.5	.0551067	.0552021	.0566042	.0428605
5000	50	0	0	0	.5	.025394	.0253718	.0254319	.0107467
2000	20	0	0	0	.5	.0404036	.040765	.0405774	.0221858
1000	10	0	0	0	.5	.0575851	.0578183	.058127	.0366717
5000	10	1	0	0	.5	.1266388	.0246298	.0250059	.0157081
2000	4	1	0	0	.5	.1942599	.0404964	.0433556	.0308769
1000	2	1	0	0	.5	.2502959	.0590065	.0663626	.053679
5000	20	1	0	0	.5	.0844537	.0261168	.0263127	.0143181
2000	8	1	0	0	.5	.1290469	.042111	.0426953	.027116
1000	4	1	0	0	.5	.1885028	.0565638	.0580067	.043138
5000	50	1	0	0	.5	.0407104	.0242965	.024361	.0114157
2000	20	1	0	0	.5	.0652527	.0437197	.0439901	.0227399
1000	10	1	0	0	.5	.093492	.0576351	.0581738	.0352087
5000	10	0	1	0	.5	.2671997	.2677806	.2670757	.2674304
2000	4	0	1	0	.5	.3864025	.3867695	.3879638	.3859248
1000	2	0	1	0	.5	.5503045	.5520791	.5560681	.5502025
5000	20	0	1	0	.5	.1842355	.1845973	.1843303	.184637
2000	8	0	1	0	.5	.2865105	.2871904	.2872868	.2869938
1000	4	0	1	0	.5	.390057	.3908735	.3921982	.3915064
5000	50	0	1	0	.5	.1155935	.1156199	.1151272	.1155797
2000	20	0	1	0	.5	.1814592	.1820318	.1821038	.1821622
1000	10	0	1	0	.5	.2656759	.2666606	.2662701	.2659066
5000	10	1	1	0	.5	.279882	.2562077	.2560638	.2561944
2000	4	1	1	0	.5	.4298792	.3938629	.3936592	.3934488
1000	2	1	1	0	.5	.6291368	.5717297	.5757304	.5715795
5000	20	1	1	0	.5	.1944749	.1776687	.1775766	.1775938
2000	8	1	1	0	.5	.3098164	.287318	.2873423	.2869044

1000	4	1	1	0	.5	.4209433	.3902172	.3898628	.3904644
5000	50	1	1	0	.5	.1179544	.1135518	.1135338	.1132854
2000	20	1	1	0	.5	.1848507	.1766861	.1766806	.1765983
1000	10	1	1	0	.5	.2645857	.2553333	.2554402	.2546023
5000	10	.5	.5	0	.5	.2031192	.1872597	.1871886	.1870441
2000	4	.5	.5	0	.5	.3107869	.284639	.284966	.2847513
1000	2	.5	.5	0	.5	.4499642	.4010238	.4066023	.3997498
5000	20	.5	.5	0	.5	.1425952	.1293291	.1292622	.1293204
2000	8	.5	.5	0	.5	.2194604	.1966816	.1968024	.1971197
1000	4	.5	.5	0	.5	.3010827	.2861803	.2859091	.2855824
5000	50	.5	.5	0	.5	.0880183	.0862389	.0862006	.0854139
2000	20	.5	.5	0	.5	.1362788	.1332767	.1332079	.1331489
1000	10	.5	.5	0	.5	.1991788	.1959161	.1957827	.195671
5000	10	2	2	0	.5	.3883719	.3511723	.3510548	.3513047
2000	4	2	2	0	.5	.6280173	.5431522	.5433134	.5425594
1000	2	2	2	0	.5	.8863608	.7946334	.7945263	.7944489
5000	20	2	2	0	.5	.2689951	.2506115	.2505981	.2507026
2000	8	2	2	0	.5	.4427845	.4021445	.4022134	.4021469
1000	4	2	2	0	.5	.623359	.5698902	.5704347	.5699022
5000	50	2	2	0	.5	.171622	.1650375	.1650456	.1647734
2000	20	2	2	0	.5	.2637518	.2534469	.2534089	.2532989
1000	10	2	2	0	.5	.3839839	.3644895	.3646078	.3647184

SE of mean error

ssl	ssh	tsq0	tsq1	outc	beta	model1	model2	model3	model4
5000	10	0	0	0	.5	.0000208	.0000207	.0000211	.0000147
2000	4	0	0	0	.5	.0000317	.0000324	.0000337	.0000277
1000	2	0	0	0	.5	.0000515	.0000537	.0000566	.0000545
5000	20	0	0	0	.5	.0000193	.0000192	.0000196	.0000129
2000	8	0	0	0	.5	.0000307	.0000302	.0000311	.0000227
1000	4	0	0	0	.5	.0000448	.0000456	.000047	.0000373
5000	50	0	0	0	.5	.0000197	.0000194	.0000197	.0000105
2000	20	0	0	0	.5	.0000316	.0000316	.0000321	.0000198
1000	10	0	0	0	.5	.0000445	.0000444	.0000456	.0000323
5000	10	1	0	0	.5	.0000996	.0000197	.000021	.0000141
2000	4	1	0	0	.5	.000166	.0000322	.0000367	.0000268
1000	2	1	0	0	.5	.0002482	.0000521	.0000627	.0000503
5000	20	1	0	0	.5	.0000629	.0000199	.0000208	.0000134
2000	8	1	0	0	.5	.0001001	.0000336	.0000353	.0000253
1000	4	1	0	0	.5	.0001565	.0000437	.0000462	.0000363
5000	50	1	0	0	.5	.0000307	.0000183	.0000186	.0000106
2000	20	1	0	0	.5	.000048	.0000318	.0000326	.0000212
1000	10	1	0	0	.5	.0000696	.0000434	.000045	.0000304
5000	10	0	1	0	.5	.0002003	.0002001	.0002072	.0002
2000	4	0	1	0	.5	.0002979	.0002991	.0003132	.0002978
1000	2	0	1	0	.5	.0004076	.0004101	.0004361	.0004074
5000	20	0	1	0	.5	.0001397	.0001397	.0001487	.0001398
2000	8	0	1	0	.5	.0002194	.0002211	.0002301	.0002213
1000	4	0	1	0	.5	.0002984	.0002991	.0003074	.0003005
5000	50	0	1	0	.5	.0000872	.0000874	.0000911	.0000874
2000	20	0	1	0	.5	.0001402	.0001397	.000146	.0001397
1000	10	0	1	0	.5	.0001954	.000196	.0002011	.0001948
5000	10	1	1	0	.5	.0002083	.0001876	.0001875	.0001875
2000	4	1	1	0	.5	.0003374	.0002922	.0002953	.0002918
1000	2	1	1	0	.5	.0004818	.0004268	.0005155	.0004263
5000	20	1	1	0	.5	.0001493	.0001275	.0001277	.0001275
2000	8	1	1	0	.5	.0002367	.0002103	.0002106	.0002106
1000	4	1	1	0	.5	.0003232	.0003016	.000305	.0003016
5000	50	1	1	0	.5	.0000875	.000086	.000086	.0000861
2000	20	1	1	0	.5	.0001407	.0001337	.0001337	.0001337
1000	10	1	1	0	.5	.0001982	.000196	.000196	.0001954
5000	10	.5	.5	0	.5	.0001544	.0001406	.0001409	.00014
2000	4	.5	.5	0	.5	.0002434	.0002183	.0002219	.0002181
1000	2	.5	.5	0	.5	.0003469	.0003087	.0003529	.000306
5000	20	.5	.5	0	.5	.0001104	.0001004	.0001005	.0001007
2000	8	.5	.5	0	.5	.0001677	.0001513	.0001515	.0001507
1000	4	.5	.5	0	.5	.0002337	.0002237	.0002286	.0002235
5000	50	.5	.5	0	.5	.0000655	.0000643	.0000642	.0000641
2000	20	.5	.5	0	.5	.0001029	.0001014	.0001012	.0001006
1000	10	.5	.5	0	.5	.0001482	.0001425	.0001427	.0001409
5000	10	2	2	0	.5	.000278	.000256	.0002559	.0002562
2000	4	2	2	0	.5	.0004567	.0004169	.0004189	.0004173
1000	2	2	2	0	.5	.0006812	.0005953	.0007604	.0005956
5000	20	2	2	0	.5	.0002069	.0001951	.0001951	.0001951
2000	8	2	2	0	.5	.0003282	.0002989	.0002988	.0002985
1000	4	2	2	0	.5	.0004587	.0004318	.000434	.0004301
5000	50	2	2	0	.5	.0001257	.0001205	.0001205	.0001204
2000	20	2	2	0	.5	.000208	.0001985	.0001986	.0001983
1000	10	2	2	0	.5	.0002815	.000269	.000269	.0002688

Mean bias

ssl	ssh	tsq0	tsq1	outc	beta	model1	model2	model3	model4
5000	10	0	0	0	.5	.0011637	.0009644	.0010002	.000431
2000	4	0	0	0	.5	.002926	.0026794	.0030442	.0009456
1000	2	0	0	0	.5	.0029818	.0028683	.0027385	.0018475
5000	20	0	0	0	.5	-.0009809	-.0009799	-.0008139	.0001836
2000	8	0	0	0	.5	-.001124	-.0015214	-.0015028	-.0016529

1000	4	0	0	0	.5	-.0015655	-.002182	-.0020579	-.0011787
5000	50	0	0	0	.5	.0001861	.0004634	.0005302	-.0001699
2000	20	0	0	0	.5	-.0009096	-.0008484	-.0011506	-.0005261
1000	10	0	0	0	.5	.0008884	.0008917	.0002299	-.0006154
5000	10	1	0	0	.5	-.0058373	.0003306	.0001591	-.0004609
2000	4	1	0	0	.5	-.0025923	-.0005698	.000134	-.0005679
1000	2	1	0	0	.5	.0006975	-.0004175	.000187	.0001975
5000	20	1	0	0	.5	.0066985	.0017184	.0019575	.0003301
2000	8	1	0	0	.5	-.0021543	.0012342	.0007159	.0006838
1000	4	1	0	0	.5	-.0044899	-.0004373	-.0007141	-.0001096
5000	50	1	0	0	.5	-.0017452	-.0010031	-.0009658	-.0000747
2000	20	1	0	0	.5	-.0017564	-.001307	-.0011007	-.0001662
1000	10	1	0	0	.5	.0073253	.0010407	.0009785	-.0010078
5000	10	0	1	0	.5	-.0156521	-.0146442	-.0190135	-.0145048
2000	4	0	1	0	.5	.0245027	.0231416	.0194089	.0226109
1000	2	0	1	0	.5	.049609	.0489579	.0464763	.0495996
5000	20	0	1	0	.5	-.0016906	-.0010472	-.0023107	-.0013664
2000	8	0	1	0	.5	-.0199119	-.0198899	-.0233989	-.0197912
1000	4	0	1	0	.5	.0102553	.0106127	.0125662	.0109112
5000	50	0	1	0	.5	.0017579	.0017692	.0024866	.0016793
2000	20	0	1	0	.5	.0109493	.010943	.0131048	.0106416
1000	10	0	1	0	.5	-.0032671	-.0035289	-.0045605	-.004191
5000	10	1	1	0	.5	-.0044096	-.0008868	-.0011272	-.0007319
2000	4	1	1	0	.5	.0005949	-.0108996	-.0107818	-.011047
1000	2	1	1	0	.5	.0125134	.0202556	.0313678	.0206807
5000	20	1	1	0	.5	.0032527	.0053435	.0052426	.004991
2000	8	1	1	0	.5	.0094998	.0047762	.0046786	.0048658
1000	4	1	1	0	.5	.0339953	.014633	.0188277	.0152828
5000	50	1	1	0	.5	.0039948	.002102	.0021645	.0025222
2000	20	1	1	0	.5	.0127793	.0154201	.0153684	.0152278
1000	10	1	1	0	.5	-.011687	-.0112983	-.0114251	-.0113763
5000	10	.5	.5	0	.5	-.0063115	-.0079136	-.0080842	-.0078597
2000	4	.5	.5	0	.5	-.0069698	-.0060266	-.0054412	-.0072343
1000	2	.5	.5	0	.5	.0005388	-.0088414	-.0149569	-.0092326
5000	20	.5	.5	0	.5	.0012938	-.0020872	-.0019507	-.0022735
2000	8	.5	.5	0	.5	-.0013931	.0017793	.0018617	.0019829
1000	4	.5	.5	0	.5	.0051364	.0067868	.0061902	.006775
5000	50	.5	.5	0	.5	.0081818	.0079673	.0079207	.0071796
2000	20	.5	.5	0	.5	.0080825	.0070139	.007084	.0076344
1000	10	.5	.5	0	.5	.0078705	.0057522	.0053369	.0062861
5000	10	2	2	0	.5	-.0166825	-.0131058	-.0130376	-.0133307
2000	4	2	2	0	.5	.0213441	.0357306	.0371128	.035963
1000	2	2	2	0	.5	.0280498	.033042	.0117391	.0333086
5000	20	2	2	0	.5	-.0200101	-.0167224	-.016691	-.0165284
2000	8	2	2	0	.5	-.022919	-.0161819	-.0160719	-.0157616
1000	4	2	2	0	.5	-.0125908	-.0007058	-.0044407	-.0003999
5000	50	2	2	0	.5	-.0091679	-.0057755	-.0058386	-.0057278
2000	20	2	2	0	.5	.0106392	.0123884	.0122825	.0122593
1000	10	2	2	0	.5	-.0113231	-.0092629	-.0093674	-.0090841

SE of mean bias

ssl	ssh	tsq0	tsq1	outc	beta	model1	model2	model3	model4
5000	10	0	0	0	.5	.0000341	.0000338	.0000345	.0000223
2000	4	0	0	0	.5	.0000511	.0000515	.0000535	.0000417
1000	2	0	0	0	.5	.0000776	.0000792	.0000839	.0000779
5000	20	0	0	0	.5	.0000319	.0000317	.0000324	.0000188
2000	8	0	0	0	.5	.0000494	.0000487	.00005	.0000344
1000	4	0	0	0	.5	.0000712	.0000717	.0000744	.0000568
5000	50	0	0	0	.5	.0000325	.000032	.0000324	.000015
2000	20	0	0	0	.5	.0000515	.0000516	.0000522	.0000298
1000	10	0	0	0	.5	.000073	.0000729	.0000749	.0000489
5000	10	1	0	0	.5	.0001611	.0000316	.0000334	.0000211
2000	4	1	0	0	.5	.0002556	.0000518	.0000579	.0000409
1000	2	1	0	0	.5	.0003526	.0000787	.0000935	.0000736
5000	20	1	0	0	.5	.0001051	.0000328	.0000343	.0000196
2000	8	1	0	0	.5	.0001633	.0000539	.0000563	.0000371
1000	4	1	0	0	.5	.000245	.0000716	.0000757	.0000564
5000	50	1	0	0	.5	.000051	.0000304	.0000309	.0000156
2000	20	1	0	0	.5	.000081	.0000541	.0000555	.0000311
1000	10	1	0	0	.5	.0001164	.0000722	.0000748	.0000465
5000	10	0	1	0	.5	.0003337	.0003341	.0003469	.0003337
2000	4	0	1	0	.5	.0004874	.0004888	.0005141	.0004871
1000	2	0	1	0	.5	.0006837	.0006866	.0007341	.0006831
5000	20	0	1	0	.5	.0002313	.0002315	.0002453	.0002317
2000	8	0	1	0	.5	.0003604	.000362	.0003773	.000362
1000	4	0	1	0	.5	.0004911	.0004922	.0005063	.0004936
5000	50	0	1	0	.5	.0001448	.000145	.0001505	.0001449
2000	20	0	1	0	.5	.0002291	.0002293	.0002389	.0002294
1000	10	0	1	0	.5	.0003299	.000331	.000339	.0003297
5000	10	1	1	0	.5	.0003489	.0003176	.0003175	.0003176
2000	4	1	1	0	.5	.0005466	.0004905	.0004957	.0004899
1000	2	1	1	0	.5	.0007926	.0007134	.0008578	.000713
5000	20	1	1	0	.5	.0002453	.0002187	.0002187	.0002186
2000	8	1	1	0	.5	.0003899	.0003561	.0003564	.000356
1000	4	1	1	0	.5	.0005298	.0004931	.0004979	.0004933
5000	50	1	1	0	.5	.0001468	.0001425	.0001424	.0001423
2000	20	1	1	0	.5	.000232	.0002211	.0002211	.000221
1000	10	1	1	0	.5	.0003305	.0003218	.0003218	.0003208
5000	10	.5	.5	0	.5	.0002552	.0002341	.0002342	.0002336
2000	4	.5	.5	0	.5	.0003948	.0003588	.0003633	.0003587
1000	2	.5	.5	0	.5	.000569	.0005061	.0005834	.0005035
5000	20	.5	.5	0	.5	.0001804	.0001638	.0001638	.0001639
2000	8	.5	.5	0	.5	.0002763	.0002482	.0002484	.0002482
1000	4	.5	.5	0	.5	.0003812	.0003633	.0003702	.0003627

5000	50	.5	.5	0	.5	.0001094	.0001073	.0001072	.0001066
2000	20	.5	.5	0	.5	.0001706	.0001673	.0001672	.0001668
1000	10	.5	.5	0	.5	.0002482	.0002423	.0002425	.0002411
5000	10	2	2	0	.5	.0004775	.0004345	.0004344	.0004347
2000	4	2	2	0	.5	.0007765	.000684	.0006865	.0006838
1000	2	2	2	0	.5	.0011179	.0009926	.0012727	.0009927
5000	20	2	2	0	.5	.0003389	.0003173	.0003172	.0003173
2000	8	2	2	0	.5	.0005508	.0005009	.000501	.0005007
1000	4	2	2	0	.5	.0007741	.0007152	.0007197	.0007142
5000	50	2	2	0	.5	.0002126	.0002043	.0002043	.0002041
2000	20	2	2	0	.5	.0003359	.0003218	.0003218	.0003216
1000	10	2	2	0	.5	.0004761	.000453	.0004531	.0004531

Power

ssl	ssh	tsq0	tsq1	outc	beta	model1	model2	model3	model4
5000	10	0	0	0	.5	100	100	100	100
2000	4	0	0	0	.5	100	99.8	99.79633	100
1000	2	0	0	0	.5	99.5996	99.5	98.9899	97.9
5000	20	0	0	0	.5	100	100	100	100
2000	8	0	0	0	.5	100	100	100	100
1000	4	0	0	0	.5	99.7992	99.7998	99.6945	99.9
5000	50	0	0	0	.5	100	100	100	100
2000	20	0	0	0	.5	100	100	100	100
1000	10	0	0	0	.5	100	100	100	100
5000	10	1	0	0	.5	38.3	100	100	100
2000	4	1	0	0	.5	28.5	100	100	100
1000	2	1	0	0	.5	42.3	99.2	98.22362	98.1
5000	20	1	0	0	.5	74.6	100	100	100
2000	8	1	0	0	.5	29.7	100	100	100
1000	4	1	0	0	.5	23.7	99.7998	99.69008	99.9
5000	50	1	0	0	.5	100	100	100	100
2000	20	1	0	0	.5	74.5	100	100	100
1000	10	1	0	0	.5	31.7	100	100	100
5000	10	0	1	0	.5	39.5	40.7	39.87474	37.1
2000	4	0	1	0	.5	33.4	33.83383	33.8591	28
1000	2	0	1	0	.5	41.14114	42.24224	42.27902	31.2
5000	20	0	1	0	.5	61.8	63.4	61.79894	60.2
2000	8	0	1	0	.5	34.4	35.1	34.238	30.7
1000	4	0	1	0	.5	31.8	32.3	31.69231	25.9
5000	50	0	1	0	.5	93.8	94.2	93.86056	93.9
2000	20	0	1	0	.5	62.3	63	62.43496	59.8
1000	10	0	1	0	.5	40.3	41.7	40.26639	37.1
5000	10	1	1	0	.5	22.1	41.4	41.2	38
2000	4	1	1	0	.5	20.1	30.1	29.727	26.1
1000	2	1	1	0	.5	31.5	40.6	42.0739	32.4
5000	20	1	1	0	.5	35	62.4	61.7	60.1
2000	8	1	1	0	.5	19.9	39.1	38.6	34.6
1000	4	1	1	0	.5	19.4	33.7	33.33333	26.9
5000	50	1	1	0	.5	76.5	94.8	94.3	94
2000	20	1	1	0	.5	34.7	64.2	63	61.6
1000	10	1	1	0	.5	19.8	40.4	39.1	36.5
5000	10	.5	.5	0	.5	40.4	64.7	64.3	61.1
2000	4	.5	.5	0	.5	28.4	45.9	45.30777	36.4
1000	2	.5	.5	0	.5	38.67735	49.2	48.8	39.3
5000	20	.5	.5	0	.5	64.6	87.8	87.4	86.9
2000	8	.5	.5	0	.5	35.3	57.7	56.9	52.1
1000	4	.5	.5	0	.5	24.9	45.5	44.70468	37.6
5000	50	.5	.5	0	.5	97.6	99.8	99.7	99.8
2000	20	.5	.5	0	.5	66.8	87.5	86.6	85.9
1000	10	.5	.5	0	.5	37.5	61.2	59.65966	56.9
5000	10	2	2	0	.5	11.8	26.1	25.9	22.1
2000	4	2	2	0	.5	15	23	22.86861	17.6
1000	2	2	2	0	.5	26.1	33.4	33.11938	25
5000	20	2	2	0	.5	15.2	38.5	38.3	35.7
2000	8	2	2	0	.5	11.1	22.4	22	19.6
1000	4	2	2	0	.5	13	21.2	20.82495	16.5
5000	50	2	2	0	.5	40.5	68	67.2	66.7
2000	20	2	2	0	.5	16.1	39.1	38.5	36.2
1000	10	2	2	0	.5	10.5	26.6	26.1	22.9

SE of power

ssl	ssh	tsq0	tsq1	outc	beta	model1	model2	model3	model4
5000	10	0	0	0	.5	0	0	0	0
2000	4	0	0	0	.5	0	.1412799	.1438669	0
1000	2	0	0	0	.5	.199799	.2230471	.3178046	.4534203
5000	20	0	0	0	.5	0	0	0	0
2000	8	0	0	0	.5	0	0	0	0
1000	4	0	0	0	.5	.1418467	.1414211	.1761103	.09995
5000	50	0	0	0	.5	0	0	0	0
2000	20	0	0	0	.5	0	0	0	0
1000	10	0	0	0	.5	0	0	0	0
5000	10	1	0	0	.5	1.537241	0	0	0
2000	4	1	0	0	.5	1.427498	0	0	0
1000	2	1	0	0	.5	1.562277	.2817091	.4269927	.4317291
5000	20	1	0	0	.5	1.376532	0	0	0
2000	8	1	0	0	.5	1.44496	0	0	0
1000	4	1	0	0	.5	1.344734	.1414211	.1786534	.09995

5000	50	1	0	0	.5	0	0	0	0
2000	20	1	0	0	.5	1.378314	0	0	0
1000	10	1	0	0	.5	1.471431	0	0	0
5000	10	0	1	0	.5	1.545882	1.553548	1.581957	1.527609
2000	4	0	1	0	.5	1.491456	1.496962	1.534555	1.419859
1000	2	0	1	0	.5	1.556902	1.562773	1.612117	1.465114
5000	20	0	1	0	.5	1.536476	1.523299	1.580565	1.547889
2000	8	0	1	0	.5	1.502212	1.509301	1.533059	1.458599
1000	4	0	1	0	.5	1.472671	1.478753	1.490079	1.385348
5000	50	0	1	0	.5	.7626008	.7391617	.7743623	.7568289
2000	20	0	1	0	.5	1.53255	1.526761	1.562227	1.550471
1000	10	0	1	0	.5	1.5511	1.559202	1.569842	1.527609
5000	10	1	1	0	.5	1.312094	1.557575	1.556458	1.534927
2000	4	1	1	0	.5	1.267277	1.450514	1.453355	1.388809
1000	2	1	1	0	.5	1.468928	1.552946	1.704365	1.479946
5000	20	1	1	0	.5	1.50831	1.531744	1.537241	1.548544
2000	8	1	1	0	.5	1.262533	1.54311	1.539493	1.504274
1000	4	1	1	0	.5	1.250456	1.494761	1.498222	1.40228
5000	50	1	1	0	.5	1.340802	.7021111	.7331507	.7509993
2000	20	1	1	0	.5	1.505294	1.516034	1.526761	1.537999
1000	10	1	1	0	.5	1.260143	1.551722	1.54311	1.522416
5000	10	.5	.5	0	.5	1.551722	1.511261	1.515094	1.541684
2000	4	.5	.5	0	.5	1.425987	1.575814	1.581293	1.521526
1000	2	.5	.5	0	.5	1.541607	1.580936	1.689822	1.54451
5000	20	.5	.5	0	.5	1.51223	1.034969	1.0494	1.066954
2000	8	.5	.5	0	.5	1.511261	1.562277	1.566011	1.579744
1000	4	.5	.5	0	.5	1.367476	1.574722	1.586591	1.531744
5000	50	.5	.5	0	.5	.4839835	.1412799	.1729451	.1412799
2000	20	.5	.5	0	.5	1.489215	1.045825	1.077237	1.100541
1000	10	.5	.5	0	.5	1.530931	1.540961	1.552128	1.566011
5000	10	2	2	0	.5	1.020176	1.388809	1.385348	1.312094
2000	4	2	2	0	.5	1.129159	1.330789	1.330111	1.204259
1000	2	2	2	0	.5	1.388809	1.491456	1.686252	1.369306
5000	20	2	2	0	.5	1.135324	1.538749	1.537241	1.515094
2000	8	2	2	0	.5	.993373	1.318423	1.309962	1.255325
1000	4	2	2	0	.5	1.063485	1.292501	1.287932	1.173776
5000	50	2	2	0	.5	1.552337	1.475127	1.484641	1.490339
2000	20	2	2	0	.5	1.162235	1.54311	1.538749	1.519724
1000	10	2	2	0	.5	.969407	1.397297	1.388809	1.328755

Model 1: Fixed common intercept; random treatment effect; Fixed effect for baseline
Model 2: Fixed study-specific intercepts; random treatment effect; Fixed study-specific effects for baseline
Model 3: Random study intercept; random treatment effect; fixed study-specific effects for baseline
Model 4: two-stage IPD with ipdmetan

ss1: size for lower level unit (patients)
ssh: size for higher level unit (studies)
tsq0: between study variance for the intercept
tsq1: between study variance for the exposure
outc: outcome type (0=continuous)
beta: true effect size

Varying residual errors, main effect

Convergence

	ssl	ssh	tsq0	tsq1	outc	beta	model1	model2	model3	model4
	5000	10	1	0	0	.5	100	99.9	95.3	100
	2000	4	1	0	0	.5	100	100	96.5	100
	1000	2	1	0	0	.5	100	100	96.7	100
	5000	20	1	0	0	.5	100	100	96.5	100
	2000	8	1	0	0	.5	100	100	96.4	100
	1000	4	1	0	0	.5	100	99.9	96.9	100
	5000	50	1	0	0	.5	100	100	97.2	100
	2000	20	1	0	0	.5	100	100	96.4	100
	1000	10	1	0	0	.5	100	100	97.1	100
	5000	10	0	1	0	.5	100	100	94.6	100
	2000	4	0	1	0	.5	100	100	95.9	100
	1000	2	0	1	0	.5	100	100	96.2	100
	5000	20	0	1	0	.5	100	100	96.1	100
	2000	8	0	1	0	.5	100	100	95.9	100
	1000	4	0	1	0	.5	100	100	96.6	100
	5000	50	0	1	0	.5	100	100	96	100
	2000	20	0	1	0	.5	100	100	96.3	100
	1000	10	0	1	0	.5	100	100	97	100
	5000	10	1	1	0	.5	100	100	100	100
	2000	4	1	1	0	.5	100	100	99.4	100
	1000	2	1	1	0	.5	100	100	86.6	100
	5000	20	1	1	0	.5	100	100	100	100
	2000	8	1	1	0	.5	100	100	100	100
	1000	4	1	1	0	.5	100	100	99.2	100
	5000	50	1	1	0	.5	100	100	100	100
	2000	20	1	1	0	.5	100	100	100	100
	1000	10	1	1	0	.5	100	100	100	100
	5000	10	.5	.5	0	.5	100	100	100	100
	2000	4	.5	.5	0	.5	100	100	99.5	100
	1000	2	.5	.5	0	.5	99.9	100	91.6	100
	5000	20	.5	.5	0	.5	100	100	100	100
	2000	8	.5	.5	0	.5	100	100	100	100
	1000	4	.5	.5	0	.5	100	100	98.9	100
	5000	50	.5	.5	0	.5	100	100	100	100
	2000	20	.5	.5	0	.5	100	100	100	100
	1000	10	.5	.5	0	.5	100	100	99.9	100
	5000	10	2	2	0	.5	100	100	100	100
	2000	4	2	2	0	.5	100	100	99.4	100
	1000	2	2	2	0	.5	100	100	80.1	100
	5000	20	2	2	0	.5	100	100	100	100
	2000	8	2	2	0	.5	100	100	100	100
	1000	4	2	2	0	.5	100	100	98.9	100
	5000	50	2	2	0	.5	100	100	100	100
	2000	20	2	2	0	.5	100	100	100	100
	1000	10	2	2	0	.5	100	100	100	100

SE of convergence

	ssl	ssh	tsq0	tsq1	outc	beta	model1	model2	model3	model4
	5000	10	1	0	0	.5	0	.09995	.6692608	0
	2000	4	1	0	0	.5	0	0	.5811626	0
	1000	2	1	0	0	.5	0	0	.5648982	0
	5000	20	1	0	0	.5	0	0	.5811626	0
	2000	8	1	0	0	.5	0	0	.589101	0
	1000	4	1	0	0	.5	0	.09995	.5480785	0
	5000	50	1	0	0	.5	0	0	.5216896	0
	2000	20	1	0	0	.5	0	0	.589101	0
	1000	10	1	0	0	.5	0	0	.5306505	0
	5000	10	0	1	0	.5	0	0	.7147307	0
	2000	4	0	1	0	.5	0	0	.6270486	0
	1000	2	0	1	0	.5	0	0	.6046156	0
	5000	20	0	1	0	.5	0	0	.6122009	0
	2000	8	0	1	0	.5	0	0	.6270486	0
	1000	4	0	1	0	.5	0	0	.5730969	0
	5000	50	0	1	0	.5	0	0	.6196773	0
	2000	20	0	1	0	.5	0	0	.5969171	0
	1000	10	0	1	0	.5	0	0	.5394442	0
	5000	10	1	1	0	.5	0	0	0	0
	2000	4	1	1	0	.5	0	0	.244213	0
	1000	2	1	1	0	.5	0	0	1.077237	0
	5000	20	1	1	0	.5	0	0	0	0
	2000	8	1	1	0	.5	0	0	0	0
	1000	4	1	1	0	.5	0	0	.2817091	0
	5000	50	1	1	0	.5	0	0	0	0
	2000	20	1	1	0	.5	0	0	0	0
	1000	10	1	1	0	.5	0	0	0	0
	5000	10	.5	.5	0	.5	0	0	0	0
	2000	4	.5	.5	0	.5	0	0	.2230471	0
	1000	2	.5	.5	0	.5	.09995	0	.8771773	0
	5000	20	.5	.5	0	.5	0	0	0	0

2000	8	.5	.5	0	.5	0	0	0	0
1000	4	.5	.5	0	.5	0	0	.3298333	0
5000	50	.5	.5	0	.5	0	0	0	0
2000	20	.5	.5	0	.5	0	0	0	0
1000	10	.5	.5	0	.5	0	0	.09995	0
5000	10	2	2	0	.5	0	0	0	0
2000	4	2	2	0	.5	0	0	.244213	0
1000	2	2	2	0	.5	0	0	1.262533	0
5000	20	2	2	0	.5	0	0	0	0
2000	8	2	2	0	.5	0	0	0	0
1000	4	2	2	0	.5	0	0	.3298333	0
5000	50	2	2	0	.5	0	0	0	0
2000	20	2	2	0	.5	0	0	0	0
1000	10	2	2	0	.5	0	0	0	0

Coverage

ssl	ssh	tsq0	tsq1	outc	beta	model1	model2	model3	model4
5000	10	1	0	0	.5	100	95.2953	96.01259	96.2
2000	4	1	0	0	.5	100	95.3	96.89119	96.9
1000	2	1	0	0	.5	99.1	95	96.38056	96.5
5000	20	1	0	0	.5	100	94.9	95.64767	95.9
2000	8	1	0	0	.5	100	96	96.57676	96.4
1000	4	1	0	0	.5	100	94.59459	96.18163	96.3
5000	50	1	0	0	.5	100	93.9	94.54733	94.8
2000	20	1	0	0	.5	100	94.6	96.05809	96.1
1000	10	1	0	0	.5	100	95.1	95.88054	95.8
5000	10	0	1	0	.5	88.7	88.8	88.90063	90.2
2000	4	0	1	0	.5	80.4	79.7	79.87487	83.8
1000	2	0	1	0	.5	59.8	58.1	58.83576	68.2
5000	20	0	1	0	.5	90.9	90.9	91.25911	91.7
2000	8	0	1	0	.5	87.8	87.8	88.32117	89.7
1000	4	0	1	0	.5	80.1	78	79.19255	84.2
5000	50	0	1	0	.5	94.5	94.2	94.6875	94.6
2000	20	0	1	0	.5	90.9	90.2	90.96573	91.6
1000	10	0	1	0	.5	90.1	89.2	89.69072	90.9
5000	10	1	1	0	.5	94.9	88.6	88.8	90.3
2000	4	1	1	0	.5	87.8	80.2	80.78471	83.9
1000	2	1	1	0	.5	68.3	56.5	55.31178	65.2
5000	20	1	1	0	.5	97.3	91.1	91.6	91.8
2000	8	1	1	0	.5	94	85.1	85.9	87.2
1000	4	1	1	0	.5	88.7	76.9	77.72177	82.2
5000	50	1	1	0	.5	98.9	94.2	94.3	94.4
2000	20	1	1	0	.5	97.6	90.8	91	91.8
1000	10	1	1	0	.5	96.7	87.3	88.1	89.6
5000	10	.5	.5	0	.5	96	88.8	89.4	90.9
2000	4	.5	.5	0	.5	88.7	79	80.30151	83.9
1000	2	.5	.5	0	.5	64.46446	54	56.44105	64.4
5000	20	.5	.5	0	.5	98.1	92.7	93.4	93.8
2000	8	.5	.5	0	.5	96.9	88.5	89.4	91.5
1000	4	.5	.5	0	.5	88.7	77.9	79.27199	82.2
5000	50	.5	.5	0	.5	99	94.2	94.6	94.9
2000	20	.5	.5	0	.5	98.1	92.7	93.4	93.9
1000	10	.5	.5	0	.5	96	86.6	88.18819	89.3
5000	10	2	2	0	.5	96.5	87.5	87.5	88.3
2000	4	2	2	0	.5	88.9	77	76.86117	80.9
1000	2	2	2	0	.5	69.1	58.5	54.05743	68.3
5000	20	2	2	0	.5	97.6	92	92.1	93
2000	8	2	2	0	.5	95.4	88	88.1	89.9
1000	4	2	2	0	.5	89.8	78.1	78.26087	82.7
5000	50	2	2	0	.5	99.2	93	93.3	93.4
2000	20	2	2	0	.5	98.2	89.6	89.9	91.1
1000	10	2	2	0	.5	96.5	88.8	88.9	90.2

SE of coverage

ssl	ssh	tsq0	tsq1	outc	beta	model1	model2	model3	model4
5000	10	1	0	0	.5	0	.6699142	.6338157	.6046156
2000	4	1	0	0	.5	0	.6692608	.5586959	.5480785
1000	2	1	0	0	.5	.2986469	.6892024	.6006234	.5811626
5000	20	1	0	0	.5	0	.6956939	.6568021	.6270486
2000	8	1	0	0	.5	0	.6196773	.5856205	.589101
1000	4	1	0	0	.5	0	.7154257	.6156348	.5969171
5000	50	1	0	0	.5	0	.7568289	.7282765	.7021111
2000	20	1	0	0	.5	0	.7147307	.6267318	.6122009
1000	10	1	0	0	.5	0	.6826346	.6377875	.6343185
5000	10	0	1	0	.5	1.001154	.9972763	1.021306	.9401915
2000	4	0	1	0	.5	1.255325	1.271971	1.294688	1.165144
1000	2	0	1	0	.5	1.550471	1.560253	1.586694	1.472671
5000	20	0	1	0	.5	.9094999	.9094999	.9110755	.8724162
2000	8	0	1	0	.5	1.034969	1.034969	1.037105	.9612024
1000	4	0	1	0	.5	1.262533	1.309962	1.30606	1.153412
5000	50	0	1	0	.5	.7209369	.7391617	.7238693	.7147307
2000	20	0	1	0	.5	.9094999	.9401915	.9237879	.8771773
1000	10	0	1	0	.5	.9444522	.981509	.9763421	.9094999
5000	10	1	1	0	.5	.6956939	1.005007	.9972763	.9359006
2000	4	1	1	0	.5	1.034969	1.260143	1.249669	1.162235
1000	2	1	1	0	.5	1.471431	1.567721	1.689454	1.506307
5000	20	1	1	0	.5	.5125524	.9004388	.8771773	.8676174

2000	8	1	1	0	.5	.7509993	1.126051	1.100541	1.056485
1000	4	1	1	0	.5	1.001154	1.332813	1.321161	1.209612
5000	50	1	1	0	.5	.3298333	.7391617	.7331507	.7270763
2000	20	1	1	0	.5	.4839835	.9139803	.9049862	.8676174
1000	10	1	1	0	.5	.5648982	1.052953	1.023909	.9653186
5000	10	.5	.5	0	.5	.6196773	.9972763	.973468	.9094999
2000	4	.5	.5	0	.5	1.001154	1.288022	1.26086	1.162235
1000	2	.5	.5	0	.5	1.514289	1.576071	1.638281	1.514147
5000	20	.5	.5	0	.5	.4317291	.8226239	.7851369	.7626008
2000	8	.5	.5	0	.5	.5480785	1.008836	.973468	.8819014
1000	4	.5	.5	0	.5	1.001154	1.312094	1.288963	1.209612
5000	50	.5	.5	0	.5	.3146427	.7391617	.7147307	.6956939
2000	20	.5	.5	0	.5	.4317291	.8226239	.7851369	.7568289
1000	10	.5	.5	0	.5	.6196773	1.077237	1.021129	.9775019
5000	10	2	2	0	.5	.5811626	1.045825	1.045825	1.01642
2000	4	2	2	0	.5	.993373	1.330789	1.337615	1.243057
1000	2	2	2	0	.5	1.461229	1.558124	1.760837	1.471431
5000	20	2	2	0	.5	.4839835	.8579044	.8529889	.8068457
2000	8	2	2	0	.5	.66245	1.027619	1.023909	.9528851
1000	4	2	2	0	.5	.957058	1.307819	1.311581	1.196123
5000	50	2	2	0	.5	.2817091	.8068457	.790639	.7851369
2000	20	2	2	0	.5	.4204284	.9653186	.9528851	.9004388
1000	10	2	2	0	.5	.5811626	.9972763	.993373	.9401915

Mean error

ssl	ssh	tsq0	tsq1	outc	beta	model1	model2	model3	model4
5000	10	1	0	0	.5	.1260697	.0227032	.0228591	.0226709
2000	4	1	0	0	.5	.1880775	.0356865	.0376084	.036511
1000	2	1	0	0	.5	.2559496	.0518778	.0614458	.0545991
5000	20	1	0	0	.5	.0823692	.0223473	.0226436	.0227418
2000	8	1	0	0	.5	.1309946	.0360754	.0365304	.0365466
1000	4	1	0	0	.5	.1686169	.0509979	.0534008	.0522491
5000	50	1	0	0	.5	.0397226	.0224024	.0224423	.0228053
2000	20	1	0	0	.5	.0605956	.0360304	.0360416	.0362223
1000	10	1	0	0	.5	.086842	.050928	.0509977	.0511052
5000	10	0	1	0	.5	.2550648	.2562082	.2550652	.2561395
2000	4	0	1	0	.5	.3956969	.3959701	.3951948	.3958203
1000	2	0	1	0	.5	.5620295	.5642526	.5648087	.5622553
5000	20	0	1	0	.5	.1848408	.185422	.1824277	.1854267
2000	8	0	1	0	.5	.2778434	.2778216	.2774616	.2778148
1000	4	0	1	0	.5	.3857421	.386042	.3883978	.3858549
5000	50	0	1	0	.5	.1110294	.1112976	.1111328	.1113013
2000	20	0	1	0	.5	.1842704	.1843931	.182162	.1844616
1000	10	0	1	0	.5	.24887	.2497477	.2489	.2497541
5000	10	1	1	0	.5	.2801669	.2516961	.251668	.2516807
2000	4	1	1	0	.5	.4541416	.401019	.4005178	.40088
1000	2	1	1	0	.5	.6467171	.5843129	.5793848	.5829425
5000	20	1	1	0	.5	.2049018	.1842332	.1842618	.184229
2000	8	1	1	0	.5	.3167602	.2916852	.2917287	.2917192
1000	4	1	1	0	.5	.449422	.4187749	.4185944	.4185092
5000	50	1	1	0	.5	.1191307	.1130826	.1131378	.1130824
2000	20	1	1	0	.5	.1992301	.1913465	.1912837	.1913727
1000	10	1	1	0	.5	.263641	.2567044	.2567461	.2566759
5000	10	.5	.5	0	.5	.1982397	.1782804	.1783384	.1782107
2000	4	.5	.5	0	.5	.3082441	.2769887	.2774022	.2768454
1000	2	.5	.5	0	.5	.4693262	.4234126	.4179544	.4233061
5000	20	.5	.5	0	.5	.1402102	.1301302	.1301303	.1301167
2000	8	.5	.5	0	.5	.2184563	.1992528	.1993592	.1992597
1000	4	.5	.5	0	.5	.3132129	.289554	.2899105	.2894055
5000	50	.5	.5	0	.5	.0836352	.0802659	.0802571	.0802733
2000	20	.5	.5	0	.5	.1296871	.125112	.1250927	.1252088
1000	10	.5	.5	0	.5	.1950142	.1913694	.1911295	.1913936
5000	10	2	2	0	.5	.3923104	.35269	.3527155	.3526828
2000	4	2	2	0	.5	.6346922	.5640359	.5650232	.5640234
1000	2	2	2	0	.5	.8978153	.8099144	.8268036	.8092205
5000	20	2	2	0	.5	.2788336	.2579366	.2579151	.2579287
2000	8	2	2	0	.5	.4228905	.389235	.3891265	.3892795
1000	4	2	2	0	.5	.6335591	.5875505	.5859274	.5875698
5000	50	2	2	0	.5	.1663305	.1595532	.1595703	.1595351
2000	20	2	2	0	.5	.2777085	.265881	.2659208	.2658558
1000	10	2	2	0	.5	.3758078	.3633347	.363298	.3632459

SE of mean error

ssl	ssh	tsq0	tsq1	outc	beta	model1	model2	model3	model4
5000	10	1	0	0	.5	.0001022	.0000172	.0000183	.0000173
2000	4	1	0	0	.5	.0001668	.0000277	.00003	.0000283
1000	2	1	0	0	.5	.0002913	.0000395	.0000494	.0000426
5000	20	1	0	0	.5	.0000647	.0000168	.0000178	.0000169
2000	8	1	0	0	.5	.0000996	.0000262	.0000276	.0000263
1000	4	1	0	0	.5	.0001518	.0000381	.0000407	.0000384
5000	50	1	0	0	.5	.0000314	.0000177	.0000182	.0000177
2000	20	1	0	0	.5	.0000464	.0000276	.0000288	.000028
1000	10	1	0	0	.5	.0000673	.0000377	.000039	.000038
5000	10	0	1	0	.5	.0001929	.0001924	.0002052	.0001924
2000	4	0	1	0	.5	.0002893	.0002897	.0003011	.0002897
1000	2	0	1	0	.5	.0004415	.0004431	.0004621	.0004424
5000	20	0	1	0	.5	.0001352	.0001351	.0001375	.0001351

2000	8	0	1	0	.5	.0002072	.000208	.0002149	.000208
1000	4	0	1	0	.5	.0002972	.0002967	.0003095	.0002969
5000	50	0	1	0	.5	.0000862	.0000862	.0000896	.0000862
2000	20	0	1	0	.5	.0001372	.0001373	.000141	.0001372
1000	10	0	1	0	.5	.0001914	.0001919	.0001981	.0001919
5000	10	1	1	0	.5	.0002194	.0001979	.0001979	.0001979
2000	4	1	1	0	.5	.0003451	.0003084	.0003116	.0003086
1000	2	1	1	0	.5	.0004983	.0004287	.0005014	.0004279
5000	20	1	1	0	.5	.0001512	.0001339	.0001341	.0001339
2000	8	1	1	0	.5	.0002407	.0002211	.000221	.0002211
1000	4	1	1	0	.5	.0003356	.0003098	.0003122	.0003096
5000	50	1	1	0	.5	.0000921	.0000889	.0000888	.0000888
2000	20	1	1	0	.5	.0001492	.0001478	.0001477	.0001478
1000	10	1	1	0	.5	.0002024	.0001972	.000197	.0001971
5000	10	.5	.5	0	.5	.000156	.0001341	.0001341	.000134
2000	4	.5	.5	0	.5	.0002422	.0002106	.0002121	.0002105
1000	2	.5	.5	0	.5	.0003611	.0003206	.0003379	.0003202
5000	20	.5	.5	0	.5	.0001025	.0000967	.0000965	.0000967
2000	8	.5	.5	0	.5	.000165	.0001472	.0001475	.0001472
1000	4	.5	.5	0	.5	.0002286	.000209	.0002115	.0002088
5000	50	.5	.5	0	.5	.000065	.000062	.000062	.000062
2000	20	.5	.5	0	.5	.0000979	.0000938	.0000939	.0000938
1000	10	.5	.5	0	.5	.0001462	.0001425	.0001429	.0001427
5000	10	2	2	0	.5	.0003015	.0002676	.0002676	.0002676
2000	4	2	2	0	.5	.000488	.0004354	.0004385	.0004354
1000	2	2	2	0	.5	.0006817	.0006003	.0007538	.0006003
5000	20	2	2	0	.5	.000206	.0001904	.0001903	.0001904
2000	8	2	2	0	.5	.0003144	.0002869	.000287	.000287
1000	4	2	2	0	.5	.0005072	.0004739	.0004784	.0004738
5000	50	2	2	0	.5	.0001251	.000121	.000121	.000121
2000	20	2	2	0	.5	.0002064	.0002028	.0002028	.0002028
1000	10	2	2	0	.5	.00028	.000267	.000267	.0002671

Mean bias

ssl	ssh	tsq0	tsq1	outc	beta	model1	model2	model3	model4
5000	10	1	0	0	.5	-.0036892	.0006466	.0011298	.0007115
2000	4	1	0	0	.5	.0127445	-.0023456	-.0017011	-.0026864
1000	2	1	0	0	.5	.0035604	.0029199	.0034378	.0035343
5000	20	1	0	0	.5	.0011787	-.0001871	-.0000353	-.0001251
2000	8	1	0	0	.5	-.0037587	.0023203	.0024609	.00231
1000	4	1	0	0	.5	.0106662	.0014477	.0015309	.0019516
5000	50	1	0	0	.5	-.0023174	-.0010384	-.0010551	-.0010905
2000	20	1	0	0	.5	.0000177	.0004309	.0004154	.0007378
1000	10	1	0	0	.5	.002777	.0009686	.0004962	.0013578
5000	10	0	1	0	.5	.029511	.0288614	.0319925	.0288559
2000	4	0	1	0	.5	-.019317	-.0208903	-.0252568	-.0208578
1000	2	0	1	0	.5	.0051972	.0070579	.0035156	.0070306
5000	20	0	1	0	.5	.0018357	.0017971	-.0003939	.0018271
2000	8	0	1	0	.5	-.0128543	-.0133958	-.0184118	-.0134553
1000	4	0	1	0	.5	-.0050969	-.0052295	-.0060354	-.0054135
5000	50	0	1	0	.5	-.0000249	-.0000193	.0002662	-.0000432
2000	20	0	1	0	.5	-.0010182	-.0011441	-.0014925	-.0012076
1000	10	0	1	0	.5	.0106383	.0109745	.0094562	.0109645
5000	10	1	1	0	.5	.0016192	.0009205	.0009103	.0009111
2000	4	1	1	0	.5	.0302687	.0186042	.0164597	.0185523
1000	2	1	1	0	.5	.0311305	.012236	-.0193155	.0128109
5000	20	1	1	0	.5	-.0094117	-.0029939	-.0030055	-.0030232
2000	8	1	1	0	.5	.0026108	-.0036642	-.0034025	-.0036848
1000	4	1	1	0	.5	.005238	.0010564	.0004036	.0014483
5000	50	1	1	0	.5	.0061683	.0016485	.0017658	.001671
2000	20	1	1	0	.5	.0064058	.0034198	.0035282	.0035186
1000	10	1	1	0	.5	.0059405	.005417	.0054589	.0054596
5000	10	.5	.5	0	.5	.0196803	.0118551	.0120303	.0119481
2000	4	.5	.5	0	.5	.0087769	.0060318	.0073648	.0060448
1000	2	.5	.5	0	.5	-.0097874	.0014899	-.0134606	.0013814
5000	20	.5	.5	0	.5	.0096628	.0091743	.0092341	.0091893
2000	8	.5	.5	0	.5	-.0003764	.0072902	.0074151	.007376
1000	4	.5	.5	0	.5	.0005957	.0011783	.0000403	.0014139
5000	50	.5	.5	0	.5	.0020852	.0009853	.0010595	.0009698
2000	20	.5	.5	0	.5	-.0014915	.0001775	.0001471	.0001089
1000	10	.5	.5	0	.5	.002062	.0032455	.0035286	.0029774
5000	10	2	2	0	.5	.003274	-.003496	-.0035339	-.0035183
2000	4	2	2	0	.5	-.0381191	-.0287809	-.0286399	-.0287752
1000	2	2	2	0	.5	.0605606	.0482318	.0078087	.0480607
5000	20	2	2	0	.5	-.0115633	-.0046999	-.0047677	-.0046915
2000	8	2	2	0	.5	.0067719	-.0046923	-.0044854	-.0047203
1000	4	2	2	0	.5	.0539484	.0663135	.0661982	.0662277
5000	50	2	2	0	.5	-.0012505	.0010713	.0010437	.0010852
2000	20	2	2	0	.5	-.0131256	-.0123849	-.0123951	-.0123677
1000	10	2	2	0	.5	.0153162	.0173807	.0173159	.0173848

SE of mean bias

ssl	ssh	tsq0	tsq1	outc	beta	model1	model2	model3	model4
5000	10	1	0	0	.5	.0001623	.0000285	.0000302	.0000285
2000	4	1	0	0	.5	.0002511	.0000452	.0000491	.0000461
1000	2	1	0	0	.5	.0003879	.0000652	.0000804	.0000692
5000	20	1	0	0	.5	.0001047	.0000279	.0000295	.0000283

2000	8	1	0	0	.5	.0001645	.0000446	.0000468	.000045
1000	4	1	0	0	.5	.0002267	.0000637	.0000685	.0000649
5000	50	1	0	0	.5	.0000506	.0000285	.0000294	.0000289
2000	20	1	0	0	.5	.0000763	.0000454	.0000472	.0000458
1000	10	1	0	0	.5	.0001099	.0000634	.0000654	.0000637
5000	10	0	1	0	.5	.0003185	.0003192	.0003373	.0003192
2000	4	0	1	0	.5	.00049	.0004903	.0005099	.0004902
1000	2	0	1	0	.5	.0007149	.0007177	.0007474	.0007156
5000	20	0	1	0	.5	.0002291	.0002295	.0002345	.0002295
2000	8	0	1	0	.5	.0003464	.0003469	.00036	.0003469
1000	4	0	1	0	.5	.0004871	.000487	.0005075	.000487
5000	50	0	1	0	.5	.0001406	.0001408	.0001464	.0001408
2000	20	0	1	0	.5	.0002298	.00023	.000236	.00023
1000	10	0	1	0	.5	.0003139	.0003148	.0003241	.0003149
5000	10	1	1	0	.5	.0003559	.0003203	.0003202	.0003203
2000	4	1	1	0	.5	.0005697	.0005057	.0005093	.0005057
1000	2	1	1	0	.5	.0008161	.0007248	.0008361	.0007233
5000	20	1	1	0	.5	.0002546	.0002278	.0002279	.0002278
2000	8	1	1	0	.5	.0003979	.0003661	.0003661	.0003661
1000	4	1	1	0	.5	.000561	.0005211	.0005251	.0005207
5000	50	1	1	0	.5	.0001505	.0001438	.0001439	.0001438
2000	20	1	1	0	.5	.0002489	.0002418	.0002417	.0002418
1000	10	1	1	0	.5	.0003324	.0003238	.0003237	.0003237
5000	10	.5	.5	0	.5	.0002515	.0002228	.0002229	.0002227
2000	4	.5	.5	0	.5	.000392	.000348	.0003504	.0003479
1000	2	.5	.5	0	.5	.0005927	.0005313	.0005678	.0005309
5000	20	.5	.5	0	.5	.0001735	.0001619	.0001618	.0001619
2000	8	.5	.5	0	.5	.0002739	.0002477	.000248	.0002477
1000	4	.5	.5	0	.5	.0003879	.0003572	.0003616	.000357
5000	50	.5	.5	0	.5	.0001059	.0001015	.0001015	.0001015
2000	20	.5	.5	0	.5	.0001625	.0001564	.0001565	.0001565
1000	10	.5	.5	0	.5	.0002438	.0002386	.0002388	.0002388
5000	10	2	2	0	.5	.0004949	.0004429	.0004428	.0004428
2000	4	2	2	0	.5	.0008	.0007122	.0007176	.0007122
1000	2	2	2	0	.5	.001126	.0010073	.0012787	.0010067
5000	20	2	2	0	.5	.0003466	.0003206	.0003206	.0003206
2000	8	2	2	0	.5	.0005271	.0004837	.0004837	.0004838
1000	4	2	2	0	.5	.00081	.0007522	.0007588	.0007521
5000	50	2	2	0	.5	.0002082	.0002003	.0002003	.0002003
2000	20	2	2	0	.5	.0003459	.0003343	.0003343	.0003342
1000	10	2	2	0	.5	.0004685	.0004507	.0004507	.0004507

Power

ssl	ssh	tsq0	tsq1	outc	beta	model1	model2	model3	model4
5000	10	1	0	0	.5	41.4	100	100	100
2000	4	1	0	0	.5	31.7	100	100	100
1000	2	1	0	0	.5	47.1	99.9	99.68976	99.2
5000	20	1	0	0	.5	72.4	100	100	100
2000	8	1	0	0	.5	34.2	100	100	100
1000	4	1	0	0	.5	28.4	100	100	100
5000	50	1	0	0	.5	100	100	100	100
2000	20	1	0	0	.5	73.6	100	100	100
1000	10	1	0	0	.5	38.3	100	100	100
5000	10	0	1	0	.5	40.9	40.3	40.16913	36.5
2000	4	0	1	0	.5	23.3	23.7	23.46194	18.3
1000	2	0	1	0	.5	30.9	32.1	31.18503	23.1
5000	20	0	1	0	.5	64.6	64.8	64.0999	62.8
2000	8	0	1	0	.5	29.3	30.2	29.61418	25.3
1000	4	0	1	0	.5	23.1	24.1	23.49896	17.8
5000	50	0	1	0	.5	96.2	96.4	96.45833	96.1
2000	20	0	1	0	.5	63.1	65.1	63.65524	60.6
1000	10	0	1	0	.5	40.3	42	40.20619	35.5
5000	10	1	1	0	.5	18.1	36.3	36	32.5
2000	4	1	1	0	.5	15.8	24.5	24.74849	17.3
1000	2	1	1	0	.5	26.3	31.3	31.17783	22.3
5000	20	1	1	0	.5	32.1	63.9	63.3	60.9
2000	8	1	1	0	.5	17.2	32.7	32	26.8
1000	4	1	1	0	.5	18.5	25	24.49597	17.6
5000	50	1	1	0	.5	79.3	96.1	96	95.8
2000	20	1	1	0	.5	34.2	65.6	64.4	61.8
1000	10	1	1	0	.5	19.5	37.3	36.4	31.7
5000	10	.5	.5	0	.5	42	70.9	70.4	66.9
2000	4	.5	.5	0	.5	26.8	38.5	38.29146	29.4
1000	2	.5	.5	0	.5	36.33634	42	39.08297	31.3
5000	20	.5	.5	0	.5	68.1	94.4	94.3	93.8
2000	8	.5	.5	0	.5	30.2	61	60.4	54.3
1000	4	.5	.5	0	.5	25.8	41.9	40.24267	30.1
5000	50	.5	.5	0	.5	98.9	100	100	100
2000	20	.5	.5	0	.5	66.5	93.6	93.4	92.6
1000	10	.5	.5	0	.5	36	67.3	64.96496	59.9
5000	10	2	2	0	.5	10.2	19.3	18.9	16.5
2000	4	2	2	0	.5	10.9	15.4	15.39235	11.1
1000	2	2	2	0	.5	20.8	24.7	25.34332	17.6
5000	20	2	2	0	.5	14.7	33.3	33.2	31.7
2000	8	2	2	0	.5	9.4	16.1	15.8	13.2
1000	4	2	2	0	.5	11.5	19	18.70576	14.1
5000	50	2	2	0	.5	39.8	72.6	72.5	71.9
2000	20	2	2	0	.5	15	33.7	33.3	31.4
1000	10	2	2	0	.5	8.4	21.9	21.2	18.1

SE of power

	ssl	ssh	tsq0	tsq1	outc	beta	model1	model2	model3	model4
	5000	10	1	0	0	.5	1.557575	0	0	0
	2000	4	1	0	0	.5	1.471431	0	0	0
	1000	2	1	0	0	.5	1.578477	.09995	.1788378	.2817091
	5000	20	1	0	0	.5	1.413591	0	0	0
	2000	8	1	0	0	.5	1.50012	0	0	0
	1000	4	1	0	0	.5	1.425987	0	0	0
	5000	50	1	0	0	.5	0	0	0	0
	2000	20	1	0	0	.5	1.39393	0	0	0
	1000	10	1	0	0	.5	1.537241	0	0	0
	5000	10	0	1	0	.5	1.554731	1.5511	1.593908	1.522416
	2000	4	0	1	0	.5	1.336828	1.344734	1.368395	1.222747
	1000	2	0	1	0	.5	1.461229	1.476343	1.493575	1.332813
	5000	20	0	1	0	.5	1.51223	1.510285	1.547444	1.52845
	2000	8	0	1	0	.5	1.439274	1.451882	1.474291	1.37474
	1000	4	0	1	0	.5	1.332813	1.352476	1.364173	1.209612
	5000	50	0	1	0	.5	.6046156	.589101	.596538	.6122009
	2000	20	0	1	0	.5	1.525906	1.507312	1.549975	1.545199
	1000	10	0	1	0	.5	1.5511	1.560769	1.574304	1.513192
	5000	10	1	1	0	.5	1.217534	1.520628	1.517893	1.481131
	2000	4	1	1	0	.5	1.153412	1.360055	1.368796	1.196123
	1000	2	1	1	0	.5	1.392232	1.466394	1.574085	1.316324
	5000	20	1	1	0	.5	1.476343	1.518812	1.524175	1.54311
	2000	8	1	1	0	.5	1.193382	1.483479	1.475127	1.400628
	1000	4	1	1	0	.5	1.227905	1.369306	1.365452	1.204259
	5000	50	1	1	0	.5	1.281214	.6122009	.6196773	.6343185
	2000	20	1	1	0	.5	1.50012	1.502212	1.514147	1.536476
	1000	10	1	1	0	.5	1.252897	1.529284	1.521526	1.471431
	5000	10	.5	.5	0	.5	1.560769	1.436381	1.443551	1.488083
	2000	4	.5	.5	0	.5	1.400628	1.538749	1.541033	1.440708
	1000	2	.5	.5	0	.5	1.521716	1.560769	1.612187	1.466394
	5000	20	.5	.5	0	.5	1.473903	.7270763	.7331507	.7626008
	2000	8	.5	.5	0	.5	1.451882	1.542401	1.546557	1.575281
	1000	4	.5	.5	0	.5	1.383604	1.560253	1.55934	1.450514
	5000	50	.5	.5	0	.5	.3298333	0	0	0
	2000	20	.5	.5	0	.5	1.492565	.7739767	.7851369	.8277922
	1000	10	.5	.5	0	.5	1.517893	1.483479	1.509413	1.549835
	5000	10	2	2	0	.5	.957058	1.248002	1.238059	1.173776
	2000	4	2	2	0	.5	.9854897	1.14142	1.144627	.993373
	1000	2	2	2	0	.5	1.283495	1.363785	1.536915	1.204259
	5000	20	2	2	0	.5	1.119781	1.490339	1.489215	1.471431
	2000	8	2	2	0	.5	.9228434	1.162235	1.153412	1.070402
	1000	4	2	2	0	.5	1.008836	1.240564	1.239994	1.100541
	5000	50	2	2	0	.5	1.547889	1.410404	1.412002	1.421404
	2000	20	2	2	0	.5	1.129159	1.494761	1.490339	1.467665
	1000	10	2	2	0	.5	.8771773	1.307819	1.292501	1.217534

Model 1: Fixed common intercept; random treatment effect; Fixed effect for baseline
Model 2: Fixed study-specific intercepts; random treatment effect; Fixed study-specific effects for baseline
Model 3: Random study intercept; random treatment effect; fixed study-specific effects for baseline
Model 4: two-stage IPD with ipdmetan

ss1: size for lower level unit (patients)
ssh: size for higher level unit (studies)
tsq0: between study variance for the intercept
tsq1: between study variance for the exposure
outc: outcome type (0=continuous)
beta: true effect size

Interaction effect (binary X continuous)

Convergence

ssl	ssh	tsq0	tsq1	outc	beta	model1	model2	model3	model4
5000	10	0	0	0	.4	98.9	100	99	100
2000	4	0	0	0	.4	99.3	100	97.9	100
1000	2	0	0	0	.4	100	100	98.9	100
5000	20	0	0	0	.4	98.8	100	98.6	100
2000	8	0	0	0	.4	99.7	100	99.1	100
1000	4	0	0	0	.4	99.8	100	98.3	100
5000	50	0	0	0	.4	98.8	100	98.8	100
2000	20	0	0	0	.4	99.4	100	98.9	100
1000	10	0	0	0	.4	99.2	100	98.8	100
5000	10	1	0	0	.4	100	100	96.1	100
2000	4	1	0	0	.4	100	99.8	96.5	100
1000	2	1	0	0	.4	99.9	100	96.9	100
5000	20	1	0	0	.4	100	99.9	96.5	100
2000	8	1	0	0	.4	100	100	96.1	100
1000	4	1	0	0	.4	100	100	96	100
5000	50	1	0	0	.4	100	100	96.5	100
2000	20	1	0	0	.4	100	100	97.5	100
1000	10	1	0	0	.4	100	100	96.8	100
5000	10	0	1	0	.4	100	100	94.9	100
2000	4	0	1	0	.4	100	100	95.4	100
1000	2	0	1	0	.4	99.9	100	94.2	100
5000	20	0	1	0	.4	100	100	96.5	100
2000	8	0	1	0	.4	100	100	95.3	100
1000	4	0	1	0	.4	100	100	95.8	100
5000	50	0	1	0	.4	100	100	95.9	100
2000	20	0	1	0	.4	100	100	96.6	100
1000	10	0	1	0	.4	100	100	97.9	100
5000	10	1	1	0	.4	100	100	100	100
2000	4	1	1	0	.4	100	100	99.5	100
1000	2	1	1	0	.4	100	100	84.2	100
5000	20	1	1	0	.4	100	100	100	100
2000	8	1	1	0	.4	100	100	100	100
1000	4	1	1	0	.4	100	100	98.9	100
5000	50	1	1	0	.4	100	100	100	100
2000	20	1	1	0	.4	100	100	100	100
1000	10	1	1	0	.4	100	100	100	100
5000	10	1	1	0	.4	100	100	100	100
2000	4	1	1	0	.4	100	100	99.5	100
1000	2	1	1	0	.4	100	100	84.2	100
5000	20	1	1	0	.4	100	100	100	100
2000	8	1	1	0	.4	100	100	100	100
1000	4	1	1	0	.4	100	100	98.9	100
5000	50	1	1	0	.4	100	100	100	100
2000	20	1	1	0	.4	100	100	100	100
1000	10	1	1	0	.4	100	100	100	100
5000	10	2	2	0	.4	100	100	100	100
2000	4	2	2	0	.4	100	100	99.7	100
1000	2	2	2	0	.4	100	100	78.9	100
5000	20	2	2	0	.4	100	100	100	100
2000	8	2	2	0	.4	100	100	100	100
1000	4	2	2	0	.4	100	100	99	100
5000	50	2	2	0	.4	100	100	100	100
2000	20	2	2	0	.4	100	100	100	100
1000	10	2	2	0	.4	100	100	100	100

SE of convergence

ssl	ssh	tsq0	tsq1	outc	beta	model1	model2	model3	model4
5000	10	0	0	0	.4	.3298333	0	.3146427	0
2000	4	0	0	0	.4	.2636475	0	.4534203	0
1000	2	0	0	0	.4	0	0	.3298333	0
5000	20	0	0	0	.4	.3443254	0	.3715373	0
2000	8	0	0	0	.4	.1729451	0	.2986469	0
1000	4	0	0	0	.4	.1412799	0	.4087909	0
5000	50	0	0	0	.4	.3443254	0	.3443254	0
2000	20	0	0	0	.4	.244213	0	.3298333	0
1000	10	0	0	0	.4	.2817091	0	.3443254	0
5000	10	1	0	0	.4	0	0	.6122009	0
2000	4	1	0	0	.4	0	.1412799	.5811626	0
1000	2	1	0	0	.4	.09995	0	.5480785	0
5000	20	1	0	0	.4	0	.09995	.5811626	0
2000	8	1	0	0	.4	0	0	.6122009	0
1000	4	1	0	0	.4	0	0	.6196773	0
5000	50	1	0	0	.4	0	0	.5811626	0
2000	20	1	0	0	.4	0	0	.4937104	0
1000	10	1	0	0	.4	0	0	.5565609	0
5000	10	0	1	0	.4	0	0	.6956939	0
2000	4	0	1	0	.4	0	0	.66245	0
1000	2	0	1	0	.4	.09995	0	.7391617	0
5000	20	0	1	0	.4	0	0	.5811626	0

	2000	8	0	1	0	.4	0	0	.6692608	0
	1000	4	0	1	0	.4	0	0	.6343185	0
	5000	50	0	1	0	.4	0	0	.6270486	0
	2000	20	0	1	0	.4	0	0	.5730969	0
	1000	10	0	1	0	.4	0	0	.4534203	0
	5000	10	1	1	0	.4	0	0	0	0
	2000	4	1	1	0	.4	0	0	.2230471	0
	1000	2	1	1	0	.4	0	0	1.153412	0
	5000	20	1	1	0	.4	0	0	0	0
	2000	8	1	1	0	.4	0	0	0	0
	1000	4	1	1	0	.4	0	0	.3298333	0
	5000	50	1	1	0	.4	0	0	0	0
	2000	20	1	1	0	.4	0	0	0	0
	1000	10	1	1	0	.4	0	0	0	0
	5000	10	1	1	0	.4	0	0	0	0
	2000	4	1	1	0	.4	0	0	.2230471	0
	1000	2	1	1	0	.4	0	0	1.153412	0
	5000	20	1	1	0	.4	0	0	0	0
	2000	8	1	1	0	.4	0	0	0	0
	1000	4	1	1	0	.4	0	0	.3298333	0
	5000	50	1	1	0	.4	0	0	0	0
	2000	20	1	1	0	.4	0	0	0	0
	1000	10	1	1	0	.4	0	0	0	0
	5000	10	2	2	0	.4	0	0	0	0
	2000	4	2	2	0	.4	0	0	.1729451	0
	1000	2	2	2	0	.4	0	0	1.290267	0
	5000	20	2	2	0	.4	0	0	0	0
	2000	8	2	2	0	.4	0	0	0	0
	1000	4	2	2	0	.4	0	0	.3146427	0
	5000	50	2	2	0	.4	0	0	0	0
	2000	20	2	2	0	.4	0	0	0	0
	1000	10	2	2	0	.4	0	0	0	0

Coverage

	ssl	ssh	tsq0	tsq1	outc	beta	model1	model2	model3	model4
	5000	10	0	0	0	.4	95.0455	94.7	94.84848	95.9
	2000	4	0	0	0	.4	95.06546	95.3	95.19918	96.1
	1000	2	0	0	0	.4	95.5	95.5	95.24772	96.7
	5000	20	0	0	0	.4	93.42105	94	93.61055	94.9
	2000	8	0	0	0	.4	95.08526	95	94.85368	96.3
	1000	4	0	0	0	.4	96.09218	95.8	95.72737	97.2
	5000	50	0	0	0	.4	95.44534	95	95.24291	96.3
	2000	20	0	0	0	.4	96.07646	95.5	95.44995	95.4
	1000	10	0	0	0	.4	94.95968	95.4	95.24291	96.2
	5000	10	1	0	0	.4	95.5	95.3	95.21332	96.8
	2000	4	1	0	0	.4	95.5	95.29058	95.02591	96.4
	1000	2	1	0	0	.4	95.1952	95.1	94.94324	97
	5000	20	1	0	0	.4	95.2	94.09409	95.02591	95.3
	2000	8	1	0	0	.4	95.8	95.4	95.31738	97.1
	1000	4	1	0	0	.4	94.8	95.2	95.10417	96.3
	5000	50	1	0	0	.4	96.5	94.4	94.50777	95.7
	2000	20	1	0	0	.4	94.7	94.6	94.76923	95.1
	1000	10	1	0	0	.4	95.5	95.6	95.45455	95.9
	5000	10	0	1	0	.4	94.2	94.4	94.20443	95.7
	2000	4	0	1	0	.4	95.3	95.3	95.07338	96.1
	1000	2	0	1	0	.4	94.89489	95	95.01062	96.6
	5000	20	0	1	0	.4	94.8	94.8	94.81865	96
	2000	8	0	1	0	.4	94.9	95.5	95.06821	96.8
	1000	4	0	1	0	.4	93.7	93.7	93.73695	95.4
	5000	50	0	1	0	.4	95.7	95.6	95.62044	96
	2000	20	0	1	0	.4	94.4	94.6	94.40994	95.4
	1000	10	0	1	0	.4	94.9	95.2	94.7906	95.9
	5000	10	1	1	0	.4	94.3	94.4	94.5	95.4
	2000	4	1	1	0	.4	94	93.9	93.96985	95.4
	1000	2	1	1	0	.4	94.4	93.7	93.94299	96
	5000	20	1	1	0	.4	94.8	94.4	94.5	95.6
	2000	8	1	1	0	.4	94.2	94.2	94.3	95.7
	1000	4	1	1	0	.4	96	95.2	95.24772	96.6
	5000	50	1	1	0	.4	94.9	95.2	95.3	95
	2000	20	1	1	0	.4	93.8	93.2	93.4	95.2
	1000	10	1	1	0	.4	95	94.5	94.5	95.5
	5000	10	1	1	0	.4	94.3	94.4	94.5	95.4
	2000	4	1	1	0	.4	94	93.9	93.96985	95.4
	1000	2	1	1	0	.4	94.4	93.7	93.94299	96
	5000	20	1	1	0	.4	94.8	94.4	94.5	95.6
	2000	8	1	1	0	.4	94.2	94.2	94.3	95.7
	1000	4	1	1	0	.4	96	95.2	95.24772	96.6
	5000	50	1	1	0	.4	94.9	95.2	95.3	95
	2000	20	1	1	0	.4	93.8	93.2	93.4	95.2
	1000	10	1	1	0	.4	95	94.5	94.5	95.5
	5000	10	2	2	0	.4	94.7	94.8	94.8	95.7
	2000	4	2	2	0	.4	94.9	95	94.88465	96.5
	1000	2	2	2	0	.4	95.2	94.8	94.04309	96
	5000	20	2	2	0	.4	94.7	95.2	95.3	96.1
	2000	8	2	2	0	.4	95.8	94.7	94.7	95.8
	1000	4	2	2	0	.4	94.9	95	95.15152	96.2
	5000	50	2	2	0	.4	94.8	94.9	94.8	95.3
	2000	20	2	2	0	.4	95.8	94.7	94.8	95.5
	1000	10	2	2	0	.4	94.3	94.1	94.2	95.4

SE of coverage

	ssl	ssh	tsq0	tsq1	outc	beta	model1	model2	model3	model4
	5000	10	0	0	0	.4	.6900293	.7084561	.7025304	.6270486
	2000	4	0	0	0	.4	.6873219	.6692608	.683255	.6122009
	1000	2	0	0	0	.4	.6555532	.6555532	.6765189	.5648982
	5000	20	0	0	0	.4	.7887187	.7509993	.7788535	.6956939
	2000	8	0	0	0	.4	.6846356	.6892024	.7018407	.5969171
	1000	4	0	0	0	.4	.6134029	.6343185	.6450438	.5216896
	5000	50	0	0	0	.4	.6633255	.6892024	.6771866	.5969171
	2000	20	0	0	0	.4	.6158209	.6555532	.6626708	.66245
	1000	10	0	0	0	.4	.6946131	.66245	.6771866	.6046156
	5000	10	1	0	0	.4	.6555532	.6692608	.6886592	.5565609
	2000	4	1	0	0	.4	.6555532	.6705689	.6998651	.589101
	1000	2	1	0	0	.4	.6766478	.6826346	.7038924	.5394442
	5000	20	1	0	0	.4	.6759882	.7458332	.6998651	.6692608
	2000	8	1	0	0	.4	.6343185	.66245	.6815048	.5306505
	1000	4	1	0	0	.4	.7021111	.6759882	.69643	.5969171
	5000	50	1	0	0	.4	.5811626	.7270763	.7334059	.6414905
	2000	20	1	0	0	.4	.7084561	.7147307	.7130404	.6826346
	1000	10	1	0	0	.4	.6555532	.6485677	.669498	.6270486
	5000	10	0	1	0	.4	.7391617	.7270763	.7584916	.6414905
	2000	4	0	1	0	.4	.6692608	.6692608	.7006966	.6122009
	1000	2	0	1	0	.4	.6963716	.6892024	.7093884	.5730969
	5000	20	0	1	0	.4	.7021111	.7021111	.7135174	.6196773
	2000	8	0	1	0	.4	.6956939	.6555532	.7014128	.5565609
	1000	4	0	1	0	.4	.7683163	.7683163	.7828265	.66245
	5000	50	0	1	0	.4	.6414905	.6485677	.6608173	.6196773
	2000	20	0	1	0	.4	.7270763	.7147307	.7391432	.66245
	1000	10	0	1	0	.4	.6956939	.6759882	.7102071	.6270486
	5000	10	1	1	0	.4	.7331507	.7270763	.7209369	.66245
	2000	4	1	1	0	.4	.7509993	.7568289	.7546521	.66245
	1000	2	1	1	0	.4	.7270763	.7683163	.8220632	.6196773
	5000	20	1	1	0	.4	.7021111	.7270763	.7209369	.6485677
	2000	8	1	1	0	.4	.7391617	.7391617	.7331507	.6414905
	1000	4	1	1	0	.4	.6196773	.6759882	.6765189	.5730969
	5000	50	1	1	0	.4	.6956939	.6759882	.6692608	.6892024
	2000	20	1	1	0	.4	.7626008	.7960904	.7851369	.6759882
	1000	10	1	1	0	.4	.6892024	.7209369	.7209369	.6555532
	5000	10	1	1	0	.4	.7331507	.7270763	.7209369	.66245
	2000	4	1	1	0	.4	.7509993	.7568289	.7546521	.66245
	1000	2	1	1	0	.4	.7270763	.7683163	.8220632	.6196773
	5000	20	1	1	0	.4	.7021111	.7270763	.7209369	.6485677
	2000	8	1	1	0	.4	.7391617	.7391617	.7331507	.6414905
	1000	4	1	1	0	.4	.6196773	.6759882	.6765189	.5730969
	5000	50	1	1	0	.4	.6956939	.6759882	.6692608	.6892024
	2000	20	1	1	0	.4	.7626008	.7960904	.7851369	.6759882
	1000	10	1	1	0	.4	.6892024	.7209369	.7209369	.6555532
	5000	10	2	2	0	.4	.7084561	.7021111	.7021111	.6414905
	2000	4	2	2	0	.4	.6956939	.6892024	.6977308	.5811626
	1000	2	2	2	0	.4	.6759882	.7021111	.842627	.6196773
	5000	20	2	2	0	.4	.7084561	.6759882	.6692608	.6122009
	2000	8	2	2	0	.4	.6343185	.7084561	.7084561	.6343185
	1000	4	2	2	0	.4	.6956939	.6892024	.6826424	.6046156
	5000	50	2	2	0	.4	.7021111	.6956939	.7021111	.6692608
	2000	20	2	2	0	.4	.6343185	.7084561	.7021111	.6555532
	1000	10	2	2	0	.4	.7331507	.7451107	.7391617	.66245

Mean error

	ssl	ssh	tsq0	tsq1	outc	beta	model1	model2	model3	model4
	5000	10	0	0	0	.4	.022452	.0224959	.0224697	.0225634
	2000	4	0	0	0	.4	.0365624	.0365902	.0365917	.0379082
	1000	2	0	0	0	.4	.050592	.0507723	.0506328	.055622
	5000	20	0	0	0	.4	.0237106	.0239878	.0238558	.0240456
	2000	8	0	0	0	.4	.0358867	.0362454	.0360664	.0365604
	1000	4	0	0	0	.4	.04945	.0497101	.0495973	.0518763
	5000	50	0	0	0	.4	.0228577	.0229943	.0230605	.0233415
	2000	20	0	0	0	.4	.0351512	.0359045	.0356709	.0367266
	1000	10	0	0	0	.4	.0496267	.0503176	.0502573	.0510083
	5000	10	1	0	0	.4	.0261775	.0219597	.0220115	.0220547
	2000	4	1	0	0	.4	.040019	.0349832	.0350581	.036025
	1000	2	1	0	0	.4	.0522616	.0486172	.0485807	.0535533
	5000	20	1	0	0	.4	.0273686	.0231053	.0229173	.023423
	2000	8	1	0	0	.4	.0425943	.0356562	.0355853	.0360333
	1000	4	1	0	0	.4	.0592056	.0505969	.0508601	.051272
	5000	50	1	0	0	.4	.0277514	.0235127	.02357	.0235683
	2000	20	1	0	0	.4	.0453367	.0363078	.036369	.0370768
	1000	10	1	0	0	.4	.0586726	.0487072	.0487614	.049379
	5000	10	0	1	0	.4	.0236468	.0236536	.023552	.0235997
	2000	4	0	1	0	.4	.0362813	.036537	.0366715	.0375254
	1000	2	0	1	0	.4	.0519089	.0519017	.0518591	.0546425
	5000	20	0	1	0	.4	.0234004	.0234772	.0234706	.0235765
	2000	8	0	1	0	.4	.0355867	.0357504	.0359006	.0363331
	1000	4	0	1	0	.4	.0514595	.051496	.0519437	.0519503
	5000	50	0	1	0	.4	.0223371	.022706	.022484	.0230018
	2000	20	0	1	0	.4	.0364314	.0368666	.0367925	.0369231
	1000	10	0	1	0	.4	.0509534	.0520401	.0520927	.052242
	5000	10	1	1	0	.4	.0271824	.0233208	.0233198	.0237342
	2000	4	1	1	0	.4	.0435515	.0382261	.0383384	.0391092
	1000	2	1	1	0	.4	.0567066	.053117	.0525726	.0563221
	5000	20	1	1	0	.4	.0274105	.0228595	.0228657	.0231223
	2000	8	1	1	0	.4	.044134	.0363561	.0363669	.0370792

	ssl	ssh	tsq0	tsq1	outc	beta	model1	model2	model3	model4
	1000	4	1	1	0	.4	.0570038	.0505305	.0507818	.0520281
	5000	50	1	1	0	.4	.0280546	.0228097	.0228096	.0229812
	2000	20	1	1	0	.4	.0440376	.0375185	.0375035	.0379946
	1000	10	1	1	0	.4	.0613091	.0511046	.0511221	.0517577
	5000	10	1	1	0	.4	.0271824	.0233208	.0233198	.0237342
	2000	4	1	1	0	.4	.0435515	.0382261	.0383384	.0391092
	1000	2	1	1	0	.4	.0567066	.053117	.0525726	.0563221
	5000	20	1	1	0	.4	.0274105	.0228595	.0228657	.0231223
	2000	8	1	1	0	.4	.044134	.0363561	.0363669	.0370792
	1000	4	1	1	0	.4	.0570038	.0505305	.0507818	.0520281
	5000	50	1	1	0	.4	.0280546	.0228097	.0228096	.0229812
	2000	20	1	1	0	.4	.0440376	.0375185	.0375035	.0379946
	1000	10	1	1	0	.4	.0613091	.0511046	.0511221	.0517577
	5000	10	2	2	0	.4	.0307331	.0229675	.0229676	.0232204
	2000	4	2	2	0	.4	.0463273	.0368708	.0368723	.0375545
	1000	2	2	2	0	.4	.0582594	.0515708	.0530906	.0557799
	5000	20	2	2	0	.4	.0308855	.0228003	.0227988	.0229118
	2000	8	2	2	0	.4	.0493733	.0357359	.0357384	.0364287
	1000	4	2	2	0	.4	.0667709	.051325	.0513244	.0522359
	5000	50	2	2	0	.4	.0318209	.022732	.0227268	.0231087
	2000	20	2	2	0	.4	.0503344	.0364088	.0363563	.0368362
	1000	10	2	2	0	.4	.0717104	.0525724	.0525746	.0535044

SE of mean error

	ssl	ssh	tsq0	tsq1	outc	beta	model1	model2	model3	model4
	5000	10	0	0	0	.4	.0000173	.0000171	.0000173	.0000171
	2000	4	0	0	0	.4	.0000275	.0000271	.0000279	.0000285
	1000	2	0	0	0	.4	.0000379	.000038	.0000384	.0000436
	5000	20	0	0	0	.4	.0000178	.0000174	.0000177	.0000175
	2000	8	0	0	0	.4	.0000262	.0000263	.0000265	.000027
	1000	4	0	0	0	.4	.0000368	.0000369	.0000375	.0000385
	5000	50	0	0	0	.4	.0000171	.0000172	.0000172	.0000174
	2000	20	0	0	0	.4	.0000273	.0000275	.0000278	.0000282
	1000	10	0	0	0	.4	.0000393	.0000397	.0000401	.0000404
	5000	10	1	0	0	.4	.00002	.0000167	.0000174	.0000167
	2000	4	1	0	0	.4	.0000314	.000027	.0000281	.0000274
	1000	2	1	0	0	.4	.0000422	.0000384	.0000397	.0000439
	5000	20	1	0	0	.4	.0000207	.0000171	.0000174	.0000169
	2000	8	1	0	0	.4	.0000316	.0000268	.0000279	.0000269
	1000	4	1	0	0	.4	.0000441	.0000369	.0000386	.0000378
	5000	50	1	0	0	.4	.0000204	.0000178	.0000184	.0000178
	2000	20	1	0	0	.4	.0000335	.0000278	.0000287	.0000283
	1000	10	1	0	0	.4	.0000452	.000037	.0000384	.0000384
	5000	10	0	1	0	.4	.0000176	.0000177	.0000186	.0000177
	2000	4	0	1	0	.4	.0000269	.0000269	.0000284	.0000282
	1000	2	0	1	0	.4	.0000388	.0000389	.0000411	.0000425
	5000	20	0	1	0	.4	.0000174	.0000174	.0000181	.0000175
	2000	8	0	1	0	.4	.0000266	.0000266	.0000281	.0000271
	1000	4	0	1	0	.4	.0000387	.0000388	.0000409	.0000405
	5000	50	0	1	0	.4	.0000165	.0000168	.0000174	.0000169
	2000	20	0	1	0	.4	.0000277	.0000279	.0000291	.0000282
	1000	10	0	1	0	.4	.0000382	.0000386	.0000394	.0000392
	5000	10	1	1	0	.4	.0000209	.0000173	.0000173	.0000174
	2000	4	1	1	0	.4	.0000335	.0000278	.000028	.0000281
	1000	2	1	1	0	.4	.000044	.0000396	.0000465	.0000435
	5000	20	1	1	0	.4	.0000207	.0000176	.0000176	.0000177
	2000	8	1	1	0	.4	.0000333	.0000274	.0000274	.0000279
	1000	4	1	1	0	.4	.0000422	.0000378	.0000382	.0000385
	5000	50	1	1	0	.4	.0000212	.0000174	.0000174	.0000176
	2000	20	1	1	0	.4	.000034	.0000284	.0000284	.0000289
	1000	10	1	1	0	.4	.0000465	.000039	.000039	.0000395
	5000	10	1	1	0	.4	.0000209	.0000173	.0000173	.0000174
	2000	4	1	1	0	.4	.0000335	.0000278	.000028	.0000281
	1000	2	1	1	0	.4	.000044	.0000396	.0000465	.0000435
	5000	20	1	1	0	.4	.0000207	.0000176	.0000176	.0000177
	2000	8	1	1	0	.4	.0000333	.0000274	.0000274	.0000279
	1000	4	1	1	0	.4	.0000422	.0000378	.0000382	.0000385
	5000	50	1	1	0	.4	.0000212	.0000174	.0000174	.0000176
	2000	20	1	1	0	.4	.000034	.0000284	.0000284	.0000289
	1000	10	1	1	0	.4	.0000465	.000039	.000039	.0000395
	5000	10	2	2	0	.4	.0000236	.0000175	.0000175	.0000177
	2000	4	2	2	0	.4	.0000351	.0000267	.0000268	.0000277
	1000	2	2	2	0	.4	.0000476	.0000387	.0000501	.0000443
	5000	20	2	2	0	.4	.0000244	.0000176	.0000176	.0000176
	2000	8	2	2	0	.4	.0000376	.0000277	.0000277	.0000284
	1000	4	2	2	0	.4	.0000512	.0000384	.0000388	.0000399
	5000	50	2	2	0	.4	.0000247	.0000175	.0000175	.0000179
	2000	20	2	2	0	.4	.0000369	.000028	.000028	.0000281
	1000	10	2	2	0	.4	.0000546	.0000398	.0000398	.0000405

Mean bias

	ssl	ssh	tsq0	tsq1	outc	beta	model1	model2	model3	model4
	5000	10	0	0	0	.4	-.0009484	-.0008172	-.0008071	-.0007877
	2000	4	0	0	0	.4	-.003555	-.0033961	-.0034208	-.0035244
	1000	2	0	0	0	.4	.0013214	.0013666	.0013152	.0013439
	5000	20	0	0	0	.4	-.0000643	-.0000389	-.0000404	-.0003775
	2000	8	0	0	0	.4	-.0008887	-.0010018	-.0008049	-.00106

1000	4	0	0	0	.4	.0034488	.0031497	.0030328	.003825
5000	50	0	0	0	.4	-.0007104	-.0007504	-.0008525	-.0009002
2000	20	0	0	0	.4	.0009986	.0008281	.0003946	.0009166
1000	10	0	0	0	.4	-.0024298	-.0025819	-.0026704	-.0022785
5000	10	1	0	0	.4	-.0012652	-.0004403	-.0004826	-.0005168
2000	4	1	0	0	.4	.0010852	.0009546	.0008752	.0006803
1000	2	1	0	0	.4	.0003941	.000757	.0013264	.0009878
5000	20	1	0	0	.4	.0006697	.0007857	.0006883	.0007883
2000	8	1	0	0	.4	-.0012277	-.0008488	-.0008528	-.0007862
1000	4	1	0	0	.4	.0013636	.0013163	.0006176	.001312
5000	50	1	0	0	.4	-.0035108	-.0014564	-.0014754	-.00145
2000	20	1	0	0	.4	-.0033529	-.0020131	-.0019165	-.0016541
1000	10	1	0	0	.4	-.0054051	-.0021891	-.0027432	-.0021719
5000	10	0	1	0	.4	-.001878	-.0019788	-.0018838	-.0021662
2000	4	0	1	0	.4	-.0020294	-.0019729	-.001526	-.0021735
1000	2	0	1	0	.4	-.0018287	-.0018457	-.0015135	-.0002254
5000	20	0	1	0	.4	.0000815	.0002096	.0004678	.0001958
2000	8	0	1	0	.4	.0017091	.0016752	.0017116	.0016899
1000	4	0	1	0	.4	.0006146	.0005186	.0012813	.0000294
5000	50	0	1	0	.4	-.0009233	-.0009636	-.0009291	-.0008925
2000	20	0	1	0	.4	-.0023013	-.0020888	-.0021836	-.0022079
1000	10	0	1	0	.4	.0052742	.0051562	.0053249	.00535
5000	10	1	1	0	.4	.0001975	-.0003083	-.0003091	-.0004111
2000	4	1	1	0	.4	-.001786	-.0001807	-.0002636	-.0001307
1000	2	1	1	0	.4	-.0019362	-.0019045	-.0018209	-.0021533
5000	20	1	1	0	.4	-.0003253	.0002885	.0002941	.0003841
2000	8	1	1	0	.4	-.0027043	-.0022251	-.0022346	-.0025067
1000	4	1	1	0	.4	-.0015036	-.0032315	-.0034935	-.0027553
5000	50	1	1	0	.4	-.0004939	-.0012347	-.0012265	-.0013883
2000	20	1	1	0	.4	-.0031528	-.0025448	-.0025765	-.0025325
1000	10	1	1	0	.4	.0038856	.002797	.0027821	.0028941
5000	10	1	1	0	.4	.0001975	-.0003083	-.0003091	-.0004111
2000	4	1	1	0	.4	-.001786	-.0001807	-.0002636	-.0001307
1000	2	1	1	0	.4	-.0019362	-.0019045	-.0018209	-.0021533
5000	20	1	1	0	.4	-.0003253	.0002885	.0002941	.0003841
2000	8	1	1	0	.4	-.0027043	-.0022251	-.0022346	-.0025067
1000	4	1	1	0	.4	-.0015036	-.0032315	-.0034935	-.0027553
5000	50	1	1	0	.4	-.0004939	-.0012347	-.0012265	-.0013883
2000	20	1	1	0	.4	-.0031528	-.0025448	-.0025765	-.0025325
1000	10	1	1	0	.4	.0038856	.002797	.0027821	.0028941
5000	10	2	2	0	.4	-.0018663	-.0000925	-.0001028	-.0002247
2000	4	2	2	0	.4	.0009758	-.0016635	-.0015452	-.0013774
1000	2	2	2	0	.4	-.0006789	.0002252	.0011234	.0006171
5000	20	2	2	0	.4	-.0001603	.0005559	.0005486	.000691
2000	8	2	2	0	.4	.0012458	.0015372	.0015358	.0014302
1000	4	2	2	0	.4	.0038678	.0022561	.0026796	.0034821
5000	50	2	2	0	.4	.0000627	-.0012679	-.001247	-.0010955
2000	20	2	2	0	.4	-.0017426	.000958	.0009321	.0009699
1000	10	2	2	0	.4	.0012114	.0012	.0011718	.0013892

SE of mean bias

ssl	ssh	tsq0	tsq1	outc	beta	model1	model2	model3	model4
5000	10	0	0	0	.4	.0000285	.0000283	.0000285	.0000283
2000	4	0	0	0	.4	.0000458	.0000454	.0000465	.0000473
1000	2	0	0	0	.4	.0000632	.0000634	.000064	.0000707
5000	20	0	0	0	.4	.0000299	.0000296	.00003	.0000298
2000	8	0	0	0	.4	.0000445	.0000448	.000045	.0000455
1000	4	0	0	0	.4	.0000617	.0000618	.0000628	.0000645
5000	50	0	0	0	.4	.0000288	.0000287	.000029	.0000291
2000	20	0	0	0	.4	.0000447	.0000453	.0000456	.0000463
1000	10	0	0	0	.4	.0000636	.0000641	.0000647	.000065
5000	10	1	0	0	.4	.0000329	.0000276	.0000288	.0000277
2000	4	1	0	0	.4	.0000508	.0000443	.0000459	.0000453
1000	2	1	0	0	.4	.0000672	.000062	.0000639	.0000693
5000	20	1	0	0	.4	.0000343	.0000288	.0000294	.0000289
2000	8	1	0	0	.4	.000053	.0000446	.0000464	.000045
1000	4	1	0	0	.4	.0000739	.0000626	.0000656	.0000637
5000	50	1	0	0	.4	.0000343	.0000294	.0000306	.0000295
2000	20	1	0	0	.4	.0000563	.0000457	.000047	.0000466
1000	10	1	0	0	.4	.0000739	.0000611	.0000633	.0000625
5000	10	0	1	0	.4	.0000294	.0000295	.000031	.0000294
2000	4	0	1	0	.4	.0000451	.0000453	.0000478	.0000469
1000	2	0	1	0	.4	.0000648	.0000648	.0000687	.0000692
5000	20	0	1	0	.4	.0000292	.0000292	.0000303	.0000294
2000	8	0	1	0	.4	.0000444	.0000446	.000047	.0000453
1000	4	0	1	0	.4	.0000644	.0000645	.000068	.0000659
5000	50	0	1	0	.4	.0000278	.0000283	.0000292	.0000285
2000	20	0	1	0	.4	.0000457	.0000462	.0000479	.0000464
1000	10	0	1	0	.4	.0000635	.0000646	.000066	.0000651
5000	10	1	1	0	.4	.0000343	.000029	.000029	.0000294
2000	4	1	1	0	.4	.000055	.0000473	.0000476	.0000482
1000	2	1	1	0	.4	.0000718	.0000663	.0000779	.0000711
5000	20	1	1	0	.4	.0000344	.0000288	.0000288	.0000291
2000	8	1	1	0	.4	.0000552	.0000455	.0000455	.0000464
1000	4	1	1	0	.4	.0000709	.0000631	.0000639	.0000647
5000	50	1	1	0	.4	.0000352	.0000287	.0000287	.0000289
2000	20	1	1	0	.4	.0000556	.000047	.000047	.0000477
1000	10	1	1	0	.4	.0000769	.0000642	.0000643	.0000651
5000	10	1	1	0	.4	.0000343	.000029	.000029	.0000294
2000	4	1	1	0	.4	.000055	.0000473	.0000476	.0000482
1000	2	1	1	0	.4	.0000718	.0000663	.0000779	.0000711
5000	20	1	1	0	.4	.0000344	.0000288	.0000288	.0000291
2000	8	1	1	0	.4	.0000552	.0000455	.0000455	.0000464
1000	4	1	1	0	.4	.0000709	.0000631	.0000639	.0000647

ssl	ssh	tsq0	tsq1	outc	beta	model1	model2	model3	model4
5000	50	1	1	0	.4	.0000352	.0000287	.0000287	.0000289
2000	20	1	1	0	.4	.0000556	.000047	.000047	.0000477
1000	10	1	1	0	.4	.0000769	.0000642	.0000643	.0000651
5000	10	2	2	0	.4	.0000387	.0000289	.0000289	.0000292
2000	4	2	2	0	.4	.0000581	.0000455	.0000457	.0000467
1000	2	2	2	0	.4	.0000752	.0000645	.0000839	.0000713
5000	20	2	2	0	.4	.0000394	.0000288	.0000288	.0000289
2000	8	2	2	0	.4	.0000621	.0000452	.0000452	.0000462
1000	4	2	2	0	.4	.0000841	.0000641	.0000647	.0000657
5000	50	2	2	0	.4	.0000403	.0000287	.0000287	.0000292
2000	20	2	2	0	.4	.0000624	.0000459	.0000459	.0000464
1000	10	2	2	0	.4	.0000901	.0000659	.0000659	.0000671

Power

ssl	ssh	tsq0	tsq1	outc	beta	model1	model2	model3	model4
5000	10	0	0	0	.4	100	100	100	100
2000	4	0	0	0	.4	99.29507	99.3	99.28498	95.5
1000	2	0	0	0	.4	89.4	89.1	89.38322	75.5
5000	20	0	0	0	.4	100	100	100	100
2000	8	0	0	0	.4	99.5988	99.6	99.59637	98.3
1000	4	0	0	0	.4	90.18036	89.8	90.23398	81.3
5000	50	0	0	0	.4	100	100	100	100
2000	20	0	0	0	.4	99.79879	99.6	99.69666	99.3
1000	10	0	0	0	.4	86.99597	86.4	86.74089	81.7
5000	10	1	0	0	.4	100	100	100	100
2000	4	1	0	0	.4	96.7	99.499	99.48187	95.6
1000	2	1	0	0	.4	84.18418	89	89.06089	75.1
5000	20	1	0	0	.4	100	100	100	100
2000	8	1	0	0	.4	96.6	99.7	99.58377	98.3
1000	4	1	0	0	.4	77	88.2	87.8125	80.3
5000	50	1	0	0	.4	99.9	100	100	100
2000	20	1	0	0	.4	95	98.9	98.76923	98.5
1000	10	1	0	0	.4	73.4	88.6	88.11983	82
5000	10	0	1	0	.4	100	100	100	100
2000	4	0	1	0	.4	99.1	98.9	98.95178	95.8
1000	2	0	1	0	.4	86.38639	86.7	86.6242	74.3
5000	20	0	1	0	.4	100	100	100	100
2000	8	0	1	0	.4	99.3	99.3	99.26548	97.9
1000	4	0	1	0	.4	88.7	88.5	88.62213	80.7
5000	50	0	1	0	.4	100	100	100	100
2000	20	0	1	0	.4	99	99.3	99.17184	98.9
1000	10	0	1	0	.4	89	88.3	88.25332	83.7
5000	10	1	1	0	.4	100	100	100	100
2000	4	1	1	0	.4	95.7	99.3	99.29648	96.7
1000	2	1	1	0	.4	81.9	87	86.93587	74.9
5000	20	1	1	0	.4	100	100	100	100
2000	8	1	1	0	.4	94.3	99.3	99.3	97.5
1000	4	1	1	0	.4	77.5	86.4	86.24874	77.3
5000	50	1	1	0	.4	100	100	100	100
2000	20	1	1	0	.4	95.1	98.8	98.8	97.9
1000	10	1	1	0	.4	75	89.2	88.6	82.7
5000	10	1	1	0	.4	100	100	100	100
2000	4	1	1	0	.4	95.7	99.3	99.29648	96.7
1000	2	1	1	0	.4	81.9	87	86.93587	74.9
5000	20	1	1	0	.4	100	100	100	100
2000	8	1	1	0	.4	94.3	99.3	99.3	97.5
1000	4	1	1	0	.4	77.5	86.4	86.24874	77.3
5000	50	1	1	0	.4	100	100	100	100
2000	20	1	1	0	.4	95.1	98.8	98.8	97.9
1000	10	1	1	0	.4	75	89.2	88.6	82.7
5000	10	2	2	0	.4	99.7	100	100	100
2000	4	2	2	0	.4	91.8	99.5	99.4985	97.1
1000	2	2	2	0	.4	81.1	87.4	87.07224	74.9
5000	20	2	2	0	.4	99.8	100	100	100
2000	8	2	2	0	.4	90.3	99	99	97.8
1000	4	2	2	0	.4	71.6	87.4	87.37374	80
5000	50	2	2	0	.4	99.8	100	100	100
2000	20	2	2	0	.4	88.8	99.5	99.5	98.8
1000	10	2	2	0	.4	64.8	87.9	87.8	82.9

SE of power

ssl	ssh	tsq0	tsq1	outc	beta	model1	model2	model3	model4
5000	10	0	0	0	.4	0	0	0	0
2000	4	0	0	0	.4	.2654994	.2636475	.2692825	.6555532
1000	2	0	0	0	.4	.973468	.9854897	.9795494	1.360055
5000	20	0	0	0	.4	0	0	0	0
2000	8	0	0	0	.4	.200199	.1995996	.2014086	.4087909
1000	4	0	0	0	.4	.9419731	.957058	.9468194	1.233009
5000	50	0	0	0	.4	0	0	0	0
2000	20	0	0	0	.4	.1421318	.1995996	.1748657	.2636475
1000	10	0	0	0	.4	1.067905	1.083993	1.078923	1.222747
5000	10	1	0	0	.4	0	0	0	0
2000	4	1	0	0	.4	.5648982	.2234929	.2311158	.6485677
1000	2	1	0	0	.4	1.154458	.9894443	1.002704	1.367476
5000	20	1	0	0	.4	0	0	0	0
2000	8	1	0	0	.4	.5730969	.1729451	.207683	.4087909
1000	4	1	0	0	.4	1.330789	1.020176	1.055844	1.25774

5000	50	1	0	0	.4	.09995	0	0	0
2000	20	1	0	0	.4	.6892024	.3298333	.3530993	.3843826
1000	10	1	0	0	.4	1.397297	1.005007	1.039945	1.214907
5000	10	0	1	0	.4	0	0	0	0
2000	4	0	1	0	.4	.2986469	.3298333	.3297338	.6343185
1000	2	0	1	0	.4	1.084992	1.07383	1.109057	1.38185
5000	20	0	1	0	.4	0	0	0	0
2000	8	0	1	0	.4	.2636475	.2636475	.2766019	.4534203
1000	4	0	1	0	.4	1.001154	1.008836	1.025933	1.248002
5000	50	0	1	0	.4	0	0	0	0
2000	20	0	1	0	.4	.3146427	.2636475	.2915829	.3298333
1000	10	0	1	0	.4	.9894443	1.01642	1.029039	1.168037
5000	10	1	1	0	.4	0	0	0	0
2000	4	1	1	0	.4	.6414905	.2636475	.2649677	.5648982
1000	2	1	1	0	.4	1.217534	1.063485	1.161405	1.371127
5000	20	1	1	0	.4	0	0	0	0
2000	8	1	1	0	.4	.7331507	.2636475	.2636475	.4937104
1000	4	1	1	0	.4	1.320511	1.083993	1.095089	1.324655
5000	50	1	1	0	.4	0	0	0	0
2000	20	1	1	0	.4	.6826346	.3443254	.3443254	.4534203
1000	10	1	1	0	.4	1.369306	.981509	1.005007	1.196123
5000	10	1	1	0	.4	0	0	0	0
2000	4	1	1	0	.4	.6414905	.2636475	.2649677	.5648982
1000	2	1	1	0	.4	1.217534	1.063485	1.161405	1.371127
5000	20	1	1	0	.4	0	0	0	0
2000	8	1	1	0	.4	.7331507	.2636475	.2636475	.4937104
1000	4	1	1	0	.4	1.320511	1.083993	1.095089	1.324655
5000	50	1	1	0	.4	0	0	0	0
2000	20	1	1	0	.4	.6826346	.3443254	.3443254	.4534203
1000	10	1	1	0	.4	1.369306	.981509	1.005007	1.196123
5000	10	2	2	0	.4	.1729451	0	0	0
2000	4	2	2	0	.4	.8676174	.2230471	.2237165	.5306505
1000	2	2	2	0	.4	1.238059	1.0494	1.194436	1.371127
5000	20	2	2	0	.4	.1412799	0	0	0
2000	8	2	2	0	.4	.9359006	.3146427	.3146427	.4638534
1000	4	2	2	0	.4	1.425987	1.0494	1.055626	1.264911
5000	50	2	2	0	.4	.1412799	0	0	0
2000	20	2	2	0	.4	.9972763	.2230471	.2230471	.3443254
1000	10	2	2	0	.4	1.510285	1.031305	1.034969	1.190626

Model 1: Fixed common intercept; random treatment effect; Fixed effect for baseline
Model 2: Fixed study-specific intercepts; random treatment effect; Fixed study-specific effects for baseline
Model 3: Random study intercept; random treatment effect; fixed study-specific effects for baseline
Model 4: two-stage IPD with ipdmetan

ss1: size for lower level unit (patients)
ssh: size for higher level unit (studies)
tsq0: between study variance for the intercept
tsq1: between study variance for the exposure
outc: outcome type (0=continuous)
beta: true effect size

Interaction effect (binary X binary)

Convergence

ssl	ssh	tsq0	tsq1	outc	beta	model1	model2	model3	model4
5000	10	0	0	0	.4	98.6	100	96.6	100
2000	4	0	0	0	.4	99.9	99.9	95.3	100
1000	2	0	0	0	.4	99.8	100	97.2	100
5000	20	0	0	0	.4	98.9	99.9	98.5	100
2000	8	0	0	0	.4	99.3	100	97.1	100
1000	4	0	0	0	.4	99.4	100	95.9	100
5000	50	0	0	0	.4	98.1	100	99.9	100
2000	20	0	0	0	.4	99.1	100	97.7	100
1000	10	0	0	0	.4	99.8	100	97.1	100
5000	10	1	0	0	.4	100	100	96.1	100
2000	4	1	0	0	.4	100	99.9	95.2	100
1000	2	1	0	0	.4	100	100	96.6	100
5000	20	1	0	0	.4	100	99.9	95.9	100
2000	8	1	0	0	.4	100	100	95.6	100
1000	4	1	0	0	.4	100	100	97.1	100
5000	50	1	0	0	.4	100	100	97.3	100
2000	20	1	0	0	.4	100	100	96.9	100
1000	10	1	0	0	.4	100	100	97.2	100
5000	10	0	1	0	.4	100	100	96	100
2000	4	0	1	0	.4	100	100	97.6	100
1000	2	0	1	0	.4	100	100	94	100
5000	20	0	1	0	.4	100	100	96.1	100
2000	8	0	1	0	.4	100	100	96	100
1000	4	0	1	0	.4	100	100	95.5	100
5000	50	0	1	0	.4	100	100	95.9	100
2000	20	0	1	0	.4	100	100	96.2	100
1000	10	0	1	0	.4	100	100	96.5	100
5000	10	1	1	0	.4	100	100	100	100
2000	4	1	1	0	.4	100	100	99.4	100
1000	2	1	1	0	.4	100	100	85.9	100
5000	20	1	1	0	.4	100	100	100	100
2000	8	1	1	0	.4	100	100	100	100
1000	4	1	1	0	.4	100	100	98.9	100
5000	50	1	1	0	.4	100	100	100	100
2000	20	1	1	0	.4	100	100	100	100
1000	10	1	1	0	.4	100	100	100	100
5000	10	1	1	0	.4	100	100	100	100
2000	4	1	1	0	.4	100	100	99.4	100
1000	2	1	1	0	.4	100	100	85.9	100
5000	20	1	1	0	.4	100	100	100	100
2000	8	1	1	0	.4	100	100	100	100
1000	4	1	1	0	.4	100	100	98.9	100
5000	50	1	1	0	.4	100	100	100	100
2000	20	1	1	0	.4	100	100	100	100
1000	10	1	1	0	.4	100	100	100	100
5000	10	2	2	0	.4	100	100	100	100
2000	4	2	2	0	.4	100	100	99.1	100
1000	2	2	2	0	.4	100	100	79.4	100
5000	20	2	2	0	.4	100	100	100	100
2000	8	2	2	0	.4	100	100	100	100
1000	4	2	2	0	.4	100	100	99.2	100
5000	50	2	2	0	.4	100	100	100	100
2000	20	2	2	0	.4	100	100	100	100
1000	10	2	2	0	.4	100	100	100	100

SE of convergence

ssl	ssh	tsq0	tsq1	outc	beta	model1	model2	model3	model4
5000	10	0	0	0	.4	.3715373	0	.5730969	0
2000	4	0	0	0	.4	.09995	.09995	.6692608	0
1000	2	0	0	0	.4	.1412799	0	.5216896	0
5000	20	0	0	0	.4	.3298333	.09995	.3843826	0
2000	8	0	0	0	.4	.2636475	0	.5306505	0
1000	4	0	0	0	.4	.244213	0	.6270486	0
5000	50	0	0	0	.4	.4317291	0	.09995	0
2000	20	0	0	0	.4	.2986469	0	.4740359	0
1000	10	0	0	0	.4	.1412799	0	.5306505	0
5000	10	1	0	0	.4	0	0	.6122009	0
2000	4	1	0	0	.4	0	.09995	.6759882	0
1000	2	1	0	0	.4	0	0	.5730969	0
5000	20	1	0	0	.4	0	.09995	.6270486	0
2000	8	1	0	0	.4	0	0	.6485677	0
1000	4	1	0	0	.4	0	0	.5306505	0
5000	50	1	0	0	.4	0	0	.5125524	0
2000	20	1	0	0	.4	0	0	.5480785	0
1000	10	1	0	0	.4	0	0	.5216896	0
5000	10	0	1	0	.4	0	0	.6196773	0
2000	4	0	1	0	.4	0	0	.4839835	0
1000	2	0	1	0	.4	0	0	.7509993	0
5000	20	0	1	0	.4	0	0	.6122009	0

2000	8	0	1	0	.4	0	0	.6196773	0
1000	4	0	1	0	.4	0	0	.6555532	0
5000	50	0	1	0	.4	0	0	.6270486	0
2000	20	0	1	0	.4	0	0	.6046156	0
1000	10	0	1	0	.4	0	0	.5811626	0
5000	10	1	1	0	.4	0	0	0	0
2000	4	1	1	0	.4	0	0	.244213	0
1000	2	1	1	0	.4	0	0	1.100541	0
5000	20	1	1	0	.4	0	0	0	0
2000	8	1	1	0	.4	0	0	0	0
1000	4	1	1	0	.4	0	0	.3298333	0
5000	50	1	1	0	.4	0	0	0	0
2000	20	1	1	0	.4	0	0	0	0
1000	10	1	1	0	.4	0	0	0	0
5000	10	1	1	0	.4	0	0	0	0
2000	4	1	1	0	.4	0	0	.244213	0
1000	2	1	1	0	.4	0	0	1.100541	0
5000	20	1	1	0	.4	0	0	0	0
2000	8	1	1	0	.4	0	0	0	0
1000	4	1	1	0	.4	0	0	.3298333	0
5000	50	1	1	0	.4	0	0	0	0
2000	20	1	1	0	.4	0	0	0	0
1000	10	1	1	0	.4	0	0	0	0
5000	10	2	2	0	.4	0	0	0	0
2000	4	2	2	0	.4	0	0	.2986469	0
1000	2	2	2	0	.4	0	0	1.278921	0
5000	20	2	2	0	.4	0	0	0	0
2000	8	2	2	0	.4	0	0	0	0
1000	4	2	2	0	.4	0	0	.2817091	0
5000	50	2	2	0	.4	0	0	0	0
2000	20	2	2	0	.4	0	0	0	0
1000	10	2	2	0	.4	0	0	0	0

Coverage

ssl	ssh	tsq0	tsq1	outc	beta	model1	model2	model3	model4
5000	10	0	0	0	.4	93.81339	94	94.09938	95.4
2000	4	0	0	0	.4	94.99499	94.89489	94.75341	96.8
1000	2	0	0	0	.4	93.88778	93.9	93.72428	95.7
5000	20	0	0	0	.4	95.55106	95.3954	95.32995	96.2
2000	8	0	0	0	.4	94.56193	94.4	94.43872	96.7
1000	4	0	0	0	.4	95.07042	94.8	95.09906	96.2
5000	50	0	0	0	.4	95.0051	94.7	95.0951	95.5
2000	20	0	0	0	.4	94.75277	94.7	94.77994	95.2
1000	10	0	0	0	.4	94.28858	94.5	94.6447	95.4
5000	10	1	0	0	.4	96.2	95.1	95.10926	96
2000	4	1	0	0	.4	93.6	94.29429	94.11765	95.6
1000	2	1	0	0	.4	94	94.5	94.51346	95.8
5000	20	1	0	0	.4	94.8	95.2953	95.41189	95.9
2000	8	1	0	0	.4	94.6	95.2	95.18828	96.2
1000	4	1	0	0	.4	94.2	95	95.26262	95.9
5000	50	1	0	0	.4	94.6	93.6	93.62795	95.1
2000	20	1	0	0	.4	95.2	93.9	93.80805	95
1000	10	1	0	0	.4	94.8	94.2	94.03292	95.3
5000	10	0	1	0	.4	95	95.2	94.79167	96.3
2000	4	0	1	0	.4	94.8	94.8	94.46721	96
1000	2	0	1	0	.4	95.2	95.5	95.10638	96.7
5000	20	0	1	0	.4	94.2	94.6	94.38085	95.1
2000	8	0	1	0	.4	95	94.8	95	96.4
1000	4	0	1	0	.4	94.6	94.6	94.55497	96
5000	50	0	1	0	.4	94.3	94.1	93.95203	94.4
2000	20	0	1	0	.4	94.7	94.2	94.38669	95.6
1000	10	0	1	0	.4	95.7	95.3	95.44041	96.2
5000	10	1	1	0	.4	94.8	94.8	94.9	96.2
2000	4	1	1	0	.4	95.3	95	94.96982	96.3
1000	2	1	1	0	.4	95.3	95.3	94.99418	97.1
5000	20	1	1	0	.4	94.5	94.9	94.9	95.5
2000	8	1	1	0	.4	94.5	94.7	94.6	95.6
1000	4	1	1	0	.4	95.5	95.1	95.14661	96.4
5000	50	1	1	0	.4	95.7	93.6	93.8	94
2000	20	1	1	0	.4	95.4	96.1	96.3	97.4
1000	10	1	1	0	.4	95.7	94.9	95.1	95.7
5000	10	1	1	0	.4	94.8	94.8	94.9	96.2
2000	4	1	1	0	.4	95.3	95	94.96982	96.3
1000	2	1	1	0	.4	95.3	95.3	94.99418	97.1
5000	20	1	1	0	.4	94.5	94.9	94.9	95.5
2000	8	1	1	0	.4	94.5	94.7	94.6	95.6
1000	4	1	1	0	.4	95.5	95.1	95.14661	96.4
5000	50	1	1	0	.4	95.7	93.6	93.8	94
2000	20	1	1	0	.4	95.4	96.1	96.3	97.4
1000	10	1	1	0	.4	95.7	94.9	95.1	95.7
5000	10	2	2	0	.4	95.7	95.7	95.7	96.8
2000	4	2	2	0	.4	95.5	95.3	95.35822	97
1000	2	2	2	0	.4	93.6	93.9	94.0806	95.4
5000	20	2	2	0	.4	94.5	95.8	95.8	96.7
2000	8	2	2	0	.4	96.1	94.5	94.5	95.2
1000	4	2	2	0	.4	95.8	94.6	94.95968	96
5000	50	2	2	0	.4	95.8	94.4	94.4	94.6
2000	20	2	2	0	.4	95.8	94.5	94.7	95.5
1000	10	2	2	0	.4	95.3	95.3	95.5	96.5

SE of coverage

ssl	ssh	tsq0	tsq1	outc	beta	model1	model2	model3	model4
5000	10	0	0	0	.4	.7672209	.7509993	.7581475	.66245
2000	4	0	0	0	.4	.6898742	.6963716	.7222533	.5565609
1000	2	0	0	0	.4	.7582962	.7568289	.7779017	.6414905
5000	20	0	0	0	.4	.6556134	.6630971	.6722911	.6046156
2000	8	0	0	0	.4	.7196243	.7270763	.7354492	.5648982
1000	4	0	0	0	.4	.6866483	.7021111	.6971375	.6046156
5000	50	0	0	0	.4	.6955086	.7084561	.6833003	.6555532
2000	20	0	0	0	.4	.708311	.7084561	.711621	.6759882
1000	10	0	0	0	.4	.7345755	.7209369	.7224879	.66245
5000	10	1	0	0	.4	.6046156	.6826346	.6957239	.6196773
2000	4	1	0	0	.4	.7739767	.7338624	.7625926	.6485677
1000	2	1	0	0	.4	.7509993	.7209369	.7326687	.6343185
5000	20	1	0	0	.4	.7021111	.6699142	.6756301	.6270486
2000	8	1	0	0	.4	.7147307	.6759882	.69217	.6046156
1000	4	1	0	0	.4	.7391617	.6892024	.6817434	.6270486
5000	50	1	0	0	.4	.7147307	.7739767	.7830433	.6826346
2000	20	1	0	0	.4	.6759882	.7568289	.7742334	.6892024
1000	10	1	0	0	.4	.7021111	.7391617	.7597797	.6692608
5000	10	0	1	0	.4	.6892024	.6759882	.7171316	.5969171
2000	4	0	1	0	.4	.7021111	.7021111	.7317919	.6196773
1000	2	0	1	0	.4	.6759882	.6555532	.7036487	.5648982
5000	20	0	1	0	.4	.7391617	.7147307	.7428745	.6826346
2000	8	0	1	0	.4	.6892024	.7021111	.7034143	.589101
1000	4	0	1	0	.4	.7147307	.7147307	.7342441	.6196773
5000	50	0	1	0	.4	.7331507	.7451107	.7697478	.7270763
2000	20	0	1	0	.4	.7084561	.7391617	.7421252	.6485677
1000	10	0	1	0	.4	.6414905	.6692608	.6715296	.6046156
5000	10	1	1	0	.4	.7021111	.7021111	.6956939	.6046156
2000	4	1	1	0	.4	.6692608	.6892024	.6932525	.5969171
1000	2	1	1	0	.4	.6692608	.6692608	.7440285	.5306505
5000	20	1	1	0	.4	.7209369	.6956939	.6956939	.6555532
2000	8	1	1	0	.4	.7209369	.7084561	.7147307	.6485677
1000	4	1	1	0	.4	.6555532	.6826346	.6833151	.589101
5000	50	1	1	0	.4	.6414905	.7739767	.7626008	.7509993
2000	20	1	1	0	.4	.66245	.6122009	.5969171	.5032296
1000	10	1	1	0	.4	.6414905	.6956939	.6826346	.6414905
5000	10	1	1	0	.4	.7021111	.7021111	.6956939	.6046156
2000	4	1	1	0	.4	.6692608	.6892024	.6932525	.5969171
1000	2	1	1	0	.4	.6692608	.6692608	.7440285	.5306505
5000	20	1	1	0	.4	.7209369	.6956939	.6956939	.6555532
2000	8	1	1	0	.4	.7209369	.7084561	.7147307	.6485677
1000	4	1	1	0	.4	.6555532	.6826346	.6833151	.589101
5000	50	1	1	0	.4	.6414905	.7739767	.7626008	.7509993
2000	20	1	1	0	.4	.66245	.6122009	.5969171	.5032296
1000	10	1	1	0	.4	.6414905	.6956939	.6826346	.6414905
5000	10	2	2	0	.4	.6414905	.6414905	.6414905	.5565609
2000	4	2	2	0	.4	.6555532	.6692608	.6683198	.5394442
1000	2	2	2	0	.4	.7739767	.7568289	.8374878	.66245
5000	20	2	2	0	.4	.7209369	.6343185	.6343185	.5648982
2000	8	2	2	0	.4	.6122009	.7209369	.7209369	.6759882
1000	4	2	2	0	.4	.6343185	.7147307	.6946131	.6196773
5000	50	2	2	0	.4	.6343185	.7270763	.7270763	.7147307
2000	20	2	2	0	.4	.6343185	.7209369	.7084561	.6555532
1000	10	2	2	0	.4	.6692608	.6692608	.6555532	.5811626

Mean error

ssl	ssh	tsq0	tsq1	outc	beta	model1	model2	model3	model4
5000	10	0	0	0	.4	.0463003	.0462324	.04622	.0468573
2000	4	0	0	0	.4	.071003	.0709317	.0708265	.072984
1000	2	0	0	0	.4	.1069869	.1069837	.1072175	.1151061
5000	20	0	0	0	.4	.0453649	.0454845	.0456171	.0457324
2000	8	0	0	0	.4	.0725527	.0729396	.0724745	.074201
1000	4	0	0	0	.4	.1009364	.1011238	.100809	.1024582
5000	50	0	0	0	.4	.0449434	.0449554	.0447695	.0458233
2000	20	0	0	0	.4	.0737366	.0734096	.0737541	.0747859
1000	10	0	0	0	.4	.1022139	.1025282	.1018517	.1034302
5000	10	1	0	0	.4	.0535428	.0446365	.0444501	.044978
2000	4	1	0	0	.4	.0862949	.0738564	.0738292	.0757891
1000	2	1	0	0	.4	.1140005	.1054668	.1049559	.117588
5000	20	1	0	0	.4	.0553041	.0453085	.0453495	.0462587
2000	8	1	0	0	.4	.0855416	.0713282	.0714235	.0727488
1000	4	1	0	0	.4	.1202345	.1009944	.1008567	.1020265
5000	50	1	0	0	.4	.0559231	.0449219	.0448827	.0454391
2000	20	1	0	0	.4	.0853211	.0699608	.070344	.0711232
1000	10	1	0	0	.4	.1202626	.1009068	.1013134	.1019966
5000	10	0	1	0	.4	.0446051	.0446916	.0447494	.0449963
2000	4	0	1	0	.4	.0723281	.0725015	.0725087	.0750557
1000	2	0	1	0	.4	.1011159	.101383	.1017353	.110539
5000	20	0	1	0	.4	.0466554	.046845	.0468886	.0473399
2000	8	0	1	0	.4	.0704392	.0704937	.070673	.0711335
1000	4	0	1	0	.4	.1027885	.1031167	.1022799	.1046345
5000	50	0	1	0	.4	.0456185	.0461877	.0458308	.0463961
2000	20	0	1	0	.4	.0731212	.0732183	.0738944	.073897
1000	10	0	1	0	.4	.1007479	.1013466	.1010916	.1013448
5000	10	1	1	0	.4	.0529274	.0440185	.0440251	.044492
2000	4	1	1	0	.4	.082438	.0720522	.0719323	.0742955
1000	2	1	1	0	.4	.1065791	.098331	.0971872	.1089653
5000	20	1	1	0	.4	.0555603	.0440334	.0440662	.044804
2000	8	1	1	0	.4	.0838531	.0727892	.0727801	.0739065

ssl	ssh	tsq0	tsq1	outc	beta	model1	model2	model3	model4
1000	4	1	1	0	.4	.1161469	.1024447	.1027559	.1039564
5000	50	1	1	0	.4	.0534039	.0446909	.0446607	.0451409
2000	20	1	1	0	.4	.0855363	.069468	.0694697	.070405
1000	10	1	1	0	.4	.1249455	.1036743	.1037839	.1057228
5000	10	1	1	0	.4	.0529274	.0440185	.0440251	.044492
2000	4	1	1	0	.4	.082438	.0720522	.0719323	.0742955
1000	2	1	1	0	.4	.1065791	.098331	.0971872	.1089653
5000	20	1	1	0	.4	.0555603	.0440334	.0440662	.044804
2000	8	1	1	0	.4	.0838531	.0727892	.0727801	.0739065
1000	4	1	1	0	.4	.1161469	.1024447	.1027559	.1039564
5000	50	1	1	0	.4	.0534039	.0446909	.0446607	.0451409
2000	20	1	1	0	.4	.0855363	.069468	.0694697	.070405
1000	10	1	1	0	.4	.1249455	.1036743	.1037839	.1057228
5000	10	2	2	0	.4	.0608403	.0453011	.0453024	.0455551
2000	4	2	2	0	.4	.0896579	.0706548	.0707647	.0715507
1000	2	2	2	0	.4	.1208776	.1042507	.1026702	.1150999
5000	20	2	2	0	.4	.0620642	.0453699	.0453613	.0459373
2000	8	2	2	0	.4	.0962979	.0712634	.0712528	.0720159
1000	4	2	2	0	.4	.1259452	.1009455	.1007075	.1034487
5000	50	2	2	0	.4	.0637109	.0473957	.0473845	.0477322
2000	20	2	2	0	.4	.0945936	.0709044	.0708502	.0708444
1000	10	2	2	0	.4	.13883	.1002222	.100223	.1017467

SE of mean error

ssl	ssh	tsq0	tsq1	outc	beta	model1	model2	model3	model4
5000	10	0	0	0	.4	.0000349	.0000343	.0000351	.000035
2000	4	0	0	0	.4	.0000525	.0000526	.0000553	.0000533
1000	2	0	0	0	.4	.0000788	.0000787	.0000808	.0000884
5000	20	0	0	0	.4	.0000334	.0000331	.0000335	.0000331
2000	8	0	0	0	.4	.0000543	.0000541	.0000558	.0000554
1000	4	0	0	0	.4	.0000792	.000079	.0000821	.0000803
5000	50	0	0	0	.4	.0000352	.0000348	.0000346	.0000346
2000	20	0	0	0	.4	.0000557	.0000553	.000057	.0000565
1000	10	0	0	0	.4	.000079	.0000786	.000081	.0000805
5000	10	1	0	0	.4	.0000402	.0000336	.000035	.0000338
2000	4	1	0	0	.4	.0000667	.0000561	.000059	.0000589
1000	2	1	0	0	.4	.000088	.0000793	.0000825	.0000929
5000	20	1	0	0	.4	.000042	.0000341	.0000355	.0000346
2000	8	1	0	0	.4	.0000639	.0000543	.0000571	.0000543
1000	4	1	0	0	.4	.0000924	.0000761	.0000784	.0000785
5000	50	1	0	0	.4	.0000424	.0000353	.0000361	.0000353
2000	20	1	0	0	.4	.0000665	.0000562	.0000582	.0000568
1000	10	1	0	0	.4	.0000925	.0000772	.0000799	.0000795
5000	10	0	1	0	.4	.0000337	.0000338	.0000352	.0000342
2000	4	0	1	0	.4	.0000565	.0000563	.0000579	.0000586
1000	2	0	1	0	.4	.0000734	.0000737	.0000786	.0000855
5000	20	0	1	0	.4	.0000339	.0000341	.0000354	.0000341
2000	8	0	1	0	.4	.0000542	.0000543	.0000564	.0000554
1000	4	0	1	0	.4	.0000772	.0000773	.0000804	.0000806
5000	50	0	1	0	.4	.000036	.0000362	.0000376	.0000364
2000	20	0	1	0	.4	.0000543	.0000546	.0000574	.0000555
1000	10	0	1	0	.4	.000075	.0000751	.0000779	.0000757
5000	10	1	1	0	.4	.0000406	.0000345	.0000345	.0000349
2000	4	1	1	0	.4	.0000613	.000053	.0000532	.0000548
1000	2	1	1	0	.4	.0000823	.0000756	.0000877	.0000876
5000	20	1	1	0	.4	.0000416	.0000347	.0000347	.0000347
2000	8	1	1	0	.4	.0000649	.0000541	.0000542	.000056
1000	4	1	1	0	.4	.0000898	.0000753	.0000762	.0000773
5000	50	1	1	0	.4	.0000412	.0000349	.0000349	.0000354
2000	20	1	1	0	.4	.0000634	.0000522	.0000522	.0000526
1000	10	1	1	0	.4	.0000926	.0000777	.0000778	.0000794
5000	10	1	1	0	.4	.0000406	.0000345	.0000345	.0000349
2000	4	1	1	0	.4	.0000613	.000053	.0000532	.0000548
1000	2	1	1	0	.4	.0000823	.0000756	.0000877	.0000876
5000	20	1	1	0	.4	.0000416	.0000347	.0000347	.0000347
2000	8	1	1	0	.4	.0000649	.0000541	.0000542	.000056
1000	4	1	1	0	.4	.0000898	.0000753	.0000762	.0000773
5000	50	1	1	0	.4	.0000412	.0000349	.0000349	.0000354
2000	20	1	1	0	.4	.0000634	.0000522	.0000522	.0000526
1000	10	1	1	0	.4	.0000926	.0000777	.0000778	.0000794
5000	10	2	2	0	.4	.0000462	.000034	.000034	.0000344
2000	4	2	2	0	.4	.0000682	.0000533	.0000537	.0000548
1000	2	2	2	0	.4	.0000962	.0000804	.0000991	.0000943
5000	20	2	2	0	.4	.0000479	.000033	.000033	.0000333
2000	8	2	2	0	.4	.0000709	.0000554	.0000554	.0000559
1000	4	2	2	0	.4	.0001012	.0000762	.0000766	.0000786
5000	50	2	2	0	.4	.0000476	.0000355	.0000355	.000036
2000	20	2	2	0	.4	.0000722	.0000544	.0000544	.0000556
1000	10	2	2	0	.4	.0001034	.0000743	.0000741	.0000749

Mean bias

ssl	ssh	tsq0	tsq1	outc	beta	model1	model2	model3	model4
5000	10	0	0	0	.4	.0014402	.001663	.0016478	.0015873
2000	4	0	0	0	.4	.0042304	.0041315	.0051056	.0055293
1000	2	0	0	0	.4	-.0012213	-.0008678	-.0007855	-.0038412
5000	20	0	0	0	.4	-.0018996	-.0016974	-.0014414	-.001766
2000	8	0	0	0	.4	.005975	.0058654	.0056572	.0065764

1000	4	0	0	0	.4	.0012158	.0015377	.0016303	.0012789
5000	50	0	0	0	.4	-.002063	-.0020892	-.0021821	-.0029914
2000	20	0	0	0	.4	.0040226	.0040736	.0041033	.0032166
1000	10	0	0	0	.4	-.0002745	.0000954	-.0003046	-.0004829
5000	10	1	0	0	.4	-.001947	-.0028523	-.0034069	-.0031894
2000	4	1	0	0	.4	-.0006784	-.001275	-.000692	-.0013703
1000	2	1	0	0	.4	.0033774	.0026963	.001986	.0013961
5000	20	1	0	0	.4	.0008377	.0005675	.0002246	.0004272
2000	8	1	0	0	.4	-.0051215	-.0053054	-.0058772	-.0050742
1000	4	1	0	0	.4	-.0091868	-.0108356	-.0111845	-.0115831
5000	50	1	0	0	.4	-.0016308	.0000779	-.0000548	-6.03e-06
2000	20	1	0	0	.4	.0004832	.00112	.0005022	.0008397
1000	10	1	0	0	.4	-.0041922	-.0047367	-.0046185	-.005503
5000	10	0	1	0	.4	-.0013295	-.0014068	-.0017381	-.0013784
2000	4	0	1	0	.4	-.0011715	-.0011333	-.0009101	-.0005191
1000	2	0	1	0	.4	.0032358	.0031485	.0028177	.0047438
5000	20	0	1	0	.4	-.0009457	-.000811	-.0008513	-.0008879
2000	8	0	1	0	.4	.0025543	.0026241	.0029106	.0019748
1000	4	0	1	0	.4	.00223	.0022137	.002604	.000468
5000	50	0	1	0	.4	-.003793	-.0038313	-.0038234	-.0035614
2000	20	0	1	0	.4	-.0020985	-.0019611	-.0019311	-.001676
1000	10	0	1	0	.4	.0011194	.0006339	.0014776	.0004633
5000	10	1	1	0	.4	-.0006215	-.0014985	-.0014917	-.0014361
2000	4	1	1	0	.4	.0002582	-.0002157	.0001214	-.00072
1000	2	1	1	0	.4	.0049799	.0032659	.0029216	.005389
5000	20	1	1	0	.4	.0011416	-.0005225	-.0005047	-.0002861
2000	8	1	1	0	.4	.0022185	.0007594	.0008058	.0003886
1000	4	1	1	0	.4	-.0036853	-.0041062	-.0042972	-.0036114
5000	50	1	1	0	.4	-.0006488	-.0006138	-.0006053	-.0005736
2000	20	1	1	0	.4	.0009577	.0000932	.0001438	.0004236
1000	10	1	1	0	.4	-.0080616	-.0061167	-.0061804	-.0053843
5000	10	1	1	0	.4	-.0006215	-.0014985	-.0014917	-.0014361
2000	4	1	1	0	.4	.0002582	-.0002157	.0001214	-.00072
1000	2	1	1	0	.4	.0049799	.0032659	.0029216	.005389
5000	20	1	1	0	.4	.0011416	-.0005225	-.0005047	-.0002861
2000	8	1	1	0	.4	.0022185	.0007594	.0008058	.0003886
1000	4	1	1	0	.4	-.0036853	-.0041062	-.0042972	-.0036114
5000	50	1	1	0	.4	-.0006488	-.0006138	-.0006053	-.0005736
2000	20	1	1	0	.4	.0009577	.0000932	.0001438	.0004236
1000	10	1	1	0	.4	-.0080616	-.0061167	-.0061804	-.0053843
5000	10	2	2	0	.4	.0004574	.0014246	.0014149	.0010704
2000	4	2	2	0	.4	-.0020208	.0035839	.0035375	.0026136
1000	2	2	2	0	.4	-.0046415	-.0057515	-.0076374	-.0061312
5000	20	2	2	0	.4	.0003094	-.000159	-.0001566	-.0002136
2000	8	2	2	0	.4	-.0017444	-.0004294	-.0004529	.0000407
1000	4	2	2	0	.4	-.006139	-.0047646	-.005361	-.0060814
5000	50	2	2	0	.4	-.0034114	.0004999	.0004367	.0003615
2000	20	2	2	0	.4	.0002602	.0009386	.000935	.0005772
1000	10	2	2	0	.4	-.0129671	-.0106985	-.0107694	-.0111761

SE of mean bias

ssl	ssh	tsq0	tsq1	outc	beta	model1	model2	model3	model4
5000	10	0	0	0	.4	.0000585	.0000576	.0000593	.0000585
2000	4	0	0	0	.4	.0000883	.0000883	.0000925	.0000903
1000	2	0	0	0	.4	.0001331	.0001328	.0001368	.0001451
5000	20	0	0	0	.4	.0000567	.0000563	.0000572	.0000564
2000	8	0	0	0	.4	.0000909	.0000907	.000093	.0000924
1000	4	0	0	0	.4	.0001288	.0001283	.0001334	.0001302
5000	50	0	0	0	.4	.0000578	.0000568	.0000566	.0000573
2000	20	0	0	0	.4	.0000929	.0000918	.0000945	.0000937
1000	10	0	0	0	.4	.0001294	.0001292	.0001326	.0001311
5000	10	1	0	0	.4	.0000669	.0000558	.0000579	.0000562
2000	4	1	0	0	.4	.0001091	.0000928	.0000975	.000096
1000	2	1	0	0	.4	.000144	.000132	.0001364	.0001499
5000	20	1	0	0	.4	.0000694	.0000568	.0000591	.0000578
2000	8	1	0	0	.4	.0001067	.0000895	.0000939	.0000907
1000	4	1	0	0	.4	.0001514	.0001261	.0001297	.0001282
5000	50	1	0	0	.4	.0000702	.0000571	.0000586	.0000576
2000	20	1	0	0	.4	.0001082	.0000897	.000093	.000091
1000	10	1	0	0	.4	.0001517	.000127	.0001313	.0001293
5000	10	0	1	0	.4	.0000559	.000056	.0000584	.0000565
2000	4	0	1	0	.4	.0000918	.0000918	.0000942	.0000952
1000	2	0	1	0	.4	.000125	.0001253	.0001338	.0001397
5000	20	0	1	0	.4	.0000577	.000058	.0000603	.0000584
2000	8	0	1	0	.4	.0000888	.000089	.0000927	.0000902
1000	4	0	1	0	.4	.0001286	.0001289	.0001339	.0001321
5000	50	0	1	0	.4	.000058	.0000586	.0000607	.0000589
2000	20	0	1	0	.4	.0000911	.0000914	.0000959	.0000924
1000	10	0	1	0	.4	.0001256	.0001262	.0001306	.0001265
5000	10	1	1	0	.4	.0000667	.0000559	.0000559	.0000565
2000	4	1	1	0	.4	.0001027	.0000895	.0000899	.0000923
1000	2	1	1	0	.4	.0001346	.000124	.0001431	.0001398
5000	20	1	1	0	.4	.0000694	.0000561	.0000561	.0000567
2000	8	1	1	0	.4	.000106	.0000907	.0000907	.0000928
1000	4	1	1	0	.4	.0001468	.0001271	.0001288	.0001295
5000	50	1	1	0	.4	.0000675	.0000567	.0000567	.0000574
2000	20	1	1	0	.4	.0001065	.0000869	.0000869	.0000879
1000	10	1	1	0	.4	.0001554	.0001295	.0001296	.0001321
5000	10	1	1	0	.4	.0000667	.0000559	.0000559	.0000565
2000	4	1	1	0	.4	.0001027	.0000895	.0000899	.0000923
1000	2	1	1	0	.4	.0001346	.000124	.0001431	.0001398
5000	20	1	1	0	.4	.0000694	.0000561	.0000561	.0000567
2000	8	1	1	0	.4	.000106	.0000907	.0000907	.0000928
1000	4	1	1	0	.4	.0001468	.0001271	.0001288	.0001295

5000	50	1	1	0	.4	.0000675	.0000567	.0000567	.0000574
2000	20	1	1	0	.4	.0001065	.0000869	.0000869	.0000879
1000	10	1	1	0	.4	.0001554	.0001295	.0001296	.0001321
5000	10	2	2	0	.4	.0000764	.0000566	.0000566	.0000571
2000	4	2	2	0	.4	.0001126	.0000885	.0000893	.0000901
1000	2	2	2	0	.4	.0001545	.0001316	.0001627	.0001487
5000	20	2	2	0	.4	.0000784	.0000561	.0000561	.0000567
2000	8	2	2	0	.4	.0001196	.0000903	.0000903	.0000912
1000	4	2	2	0	.4	.0001615	.0001264	.0001271	.0001298
5000	50	2	2	0	.4	.0000795	.0000592	.0000592	.0000598
2000	20	2	2	0	.4	.000119	.0000894	.0000893	.0000901
1000	10	2	2	0	.4	.0001727	.0001243	.0001242	.0001259

Power

ssl	ssh	tsq0	tsq1	outc	beta	model1	model2	model3	model4
5000	10	0	0	0	.4	94.72617	94.8	94.92754	91.2
2000	4	0	0	0	.4	63.36336	63.76376	64.53305	53.2
1000	2	0	0	0	.4	36.37275	36.8	36.93416	27.8
5000	20	0	0	0	.4	94.2366	94.39439	94.2132	91.7
2000	8	0	0	0	.4	63.84693	63.6	63.8517	57.3
1000	4	0	0	0	.4	36.31791	36.4	37.01773	30
5000	50	0	0	0	.4	93.67992	93.8	93.79379	92
2000	20	0	0	0	.4	62.46216	63.3	63.05015	57.9
1000	10	0	0	0	.4	34.96994	35.2	34.91246	30.2
5000	10	1	0	0	.4	84.2	94.4	94.2768	90.1
2000	4	1	0	0	.4	49.7	60.66066	61.02941	50.5
1000	2	1	0	0	.4	33	34.8	34.47205	27.7
5000	20	1	0	0	.4	82.9	94.39439	94.47341	92
2000	8	1	0	0	.4	45.1	60.3	59.83264	51.9
1000	4	1	0	0	.4	26.6	33	32.64676	26.1
5000	50	1	0	0	.4	80.9	94.5	94.45015	92.8
2000	20	1	0	0	.4	44.2	62.6	61.60991	56.8
1000	10	1	0	0	.4	24.2	34.8	34.77366	29.2
5000	10	0	1	0	.4	94	94.1	94.16667	90.5
2000	4	0	1	0	.4	62.4	62.2	62.39754	52.4
1000	2	0	1	0	.4	35.9	36.3	36.59574	29.7
5000	20	0	1	0	.4	93.3	93.5	93.44433	90.6
2000	8	0	1	0	.4	62.8	62.4	62.5	54.9
1000	4	0	1	0	.4	36.4	36.8	36.12565	29.4
5000	50	0	1	0	.4	92.3	92.4	92.07508	90.1
2000	20	0	1	0	.4	58.5	59.4	59.14761	54.2
1000	10	0	1	0	.4	35.7	36	36.26943	29.6
5000	10	1	1	0	.4	84.6	94.1	94.1	91.5
2000	4	1	1	0	.4	51.9	62.8	62.67606	52.2
1000	2	1	1	0	.4	32.1	34.8	34.45867	26.7
5000	20	1	1	0	.4	82.9	95.2	95.2	93.1
2000	8	1	1	0	.4	47.5	59.9	59.8	52.8
1000	4	1	1	0	.4	28	34.8	34.58038	28.8
5000	50	1	1	0	.4	81.9	94.1	94	91.9
2000	20	1	1	0	.4	45.6	61.8	61.6	56.2
1000	10	1	1	0	.4	25.2	35.3	35.1	29.1
5000	10	1	1	0	.4	84.6	94.1	94.1	91.5
2000	4	1	1	0	.4	51.9	62.8	62.67606	52.2
1000	2	1	1	0	.4	32.1	34.8	34.45867	26.7
5000	20	1	1	0	.4	82.9	95.2	95.2	93.1
2000	8	1	1	0	.4	47.5	59.9	59.8	52.8
1000	4	1	1	0	.4	28	34.8	34.58038	28.8
5000	50	1	1	0	.4	81.9	94.1	94	91.9
2000	20	1	1	0	.4	45.6	61.8	61.6	56.2
1000	10	1	1	0	.4	25.2	35.3	35.1	29.1
5000	10	2	2	0	.4	73.7	94.3	94.2	91
2000	4	2	2	0	.4	45.8	63.1	63.16852	53.9
1000	2	2	2	0	.4	27.6	32.6	31.48615	25.9
5000	20	2	2	0	.4	71.2	94.1	94	91.1
2000	8	2	2	0	.4	38.6	60.9	60.6	54.2
1000	4	2	2	0	.4	20.9	32.6	32.56048	26.5
5000	50	2	2	0	.4	67.5	94	93.7	91.8
2000	20	2	2	0	.4	35.6	61	61	55.5
1000	10	2	2	0	.4	20	32.9	32.6	28.1

SE of power

ssl	ssh	tsq0	tsq1	outc	beta	model1	model2	model3	model4
5000	10	0	0	0	.4	.7118029	.7021111	.70602	.8958571
2000	4	0	0	0	.4	1.524383	1.520813	1.549732	1.577897
1000	2	0	0	0	.4	1.522805	1.525044	1.548025	1.416743
5000	20	0	0	0	.4	.7410559	.7277825	.7439728	.8724162
2000	8	0	0	0	.4	1.524642	1.521526	1.541773	1.564196
1000	4	0	0	0	.4	1.525372	1.521526	1.55921	1.449138
5000	50	0	0	0	.4	.7768731	.7626008	.7633389	.8579044
2000	20	0	0	0	.4	1.538177	1.524175	1.544195	1.561278
1000	10	0	0	0	.4	1.509521	1.510285	1.529782	1.451882
5000	10	1	0	0	.4	1.153412	.7270763	.749308	.9444522
2000	4	1	0	0	.4	1.58111	1.545555	1.580591	1.58106
1000	2	1	0	0	.4	1.486943	1.506307	1.529179	1.415171
5000	20	1	0	0	.4	1.190626	.7277825	.7378603	.8579044
2000	8	1	0	0	.4	1.573528	1.547227	1.585538	1.579997
1000	4	1	0	0	.4	1.397297	1.486943	1.504838	1.388809

5000	50	1	0	0	.4	1.243057	.7209369	.7339819	.8174105
2000	20	1	0	0	.4	1.570465	1.530111	1.562331	1.566448
1000	10	1	0	0	.4	1.354385	1.506307	1.527579	1.437832
5000	10	0	1	0	.4	.7509993	.7451107	.7564346	.927227
2000	4	0	1	0	.4	1.531744	1.533349	1.550483	1.579316
1000	2	0	1	0	.4	1.516967	1.520628	1.571124	1.44496
5000	20	0	1	0	.4	.790639	.7795832	.7984052	.9228434
2000	8	0	1	0	.4	1.52845	1.531744	1.5625	1.573528
1000	4	0	1	0	.4	1.521526	1.525044	1.554424	1.440708
5000	50	0	1	0	.4	.8430362	.8379976	.8722867	.9444522
2000	20	0	1	0	.4	1.558124	1.552946	1.584856	1.575551
1000	10	0	1	0	.4	1.515094	1.517893	1.547678	1.443551
5000	10	1	1	0	.4	1.14142	.7451107	.7451107	.8819014
2000	4	1	1	0	.4	1.579997	1.52845	1.534092	1.579608
1000	2	1	1	0	.4	1.476343	1.506307	1.621475	1.398967
5000	20	1	1	0	.4	1.190626	.6759882	.6759882	.8014924
2000	8	1	1	0	.4	1.579161	1.549835	1.550471	1.578658
1000	4	1	1	0	.4	1.419859	1.506307	1.512414	1.431978
5000	50	1	1	0	.4	1.217534	.7451107	.7509993	.8627804
2000	20	1	1	0	.4	1.575005	1.536476	1.537999	1.568936
1000	10	1	1	0	.4	1.372938	1.511261	1.509301	1.436381
5000	10	1	1	0	.4	1.14142	.7451107	.7451107	.8819014
2000	4	1	1	0	.4	1.579997	1.52845	1.534092	1.579608
1000	2	1	1	0	.4	1.476343	1.506307	1.621475	1.398967
5000	20	1	1	0	.4	1.190626	.6759882	.6759882	.8014924
2000	8	1	1	0	.4	1.579161	1.549835	1.550471	1.578658
1000	4	1	1	0	.4	1.419859	1.506307	1.512414	1.431978
5000	50	1	1	0	.4	1.217534	.7451107	.7509993	.8627804
2000	20	1	1	0	.4	1.575005	1.536476	1.537999	1.568936
1000	10	1	1	0	.4	1.372938	1.511261	1.509301	1.436381
5000	10	2	2	0	.4	1.392232	.7331507	.7391617	.9049862
2000	4	2	2	0	.4	1.575551	1.525906	1.532227	1.576322
1000	2	2	2	0	.4	1.413591	1.482309	1.648309	1.385348
5000	20	2	2	0	.4	1.431978	.7451107	.7509993	.9004388
2000	8	2	2	0	.4	1.539493	1.54311	1.545199	1.575551
1000	4	2	2	0	.4	1.285764	1.482309	1.487808	1.395618
5000	50	2	2	0	.4	1.481131	.7509993	.7683163	.8676174
2000	20	2	2	0	.4	1.514147	1.542401	1.542401	1.571544
1000	10	2	2	0	.4	1.264911	1.485796	1.482309	1.421404

Model 1: Fixed common intercept; random treatment effect; Fixed effect for baseline
Model 2: Fixed study-specific intercepts; random treatment effect; Fixed study-specific effects for baseline
Model 3: Random study intercept; random treatment effect; fixed study-specific effects for baseline
Model 4: two-stage IPD with ipdmetan

ss1: size for lower level unit (patients)
ssh: size for higher level unit (studies)
tsq0: between study variance for the intercept
tsq1: between study variance for the exposure
outc: outcome type (0=continuous)
beta: true effect size

Interaction effect (binary X binary), with varying levels for the binary covariate

Convergence

	ssl	ssh	tsq0	tsq1	outc	beta	model1	model2	model3	model4
	5000	10	0	0	0	.4	99.1	99.9	96.7	100
	2000	4	0	0	0	.4	99.8	100	97.2	99.2
	1000	2	0	0	0	.4	100	100	98.9	88.8
	5000	20	0	0	0	.4	98.7	100	96.5	100
	2000	8	0	0	0	.4	99.6	100	97.2	99.9
	1000	4	0	0	0	.4	99.6	100	98.1	99
	5000	50	0	0	0	.4	99.2	100	97.5	100
	2000	20	0	0	0	.4	99.7	100	98.5	100
	1000	10	0	0	0	.4	99.6	100	97.6	100
	5000	10	1	0	0	.4	100	100	95.9	100
	2000	4	1	0	0	.4	100	100	97.1	99
	1000	2	1	0	0	.4	100	100	96.8	90.2
	5000	20	1	0	0	.4	100	100	97	100
	2000	8	1	0	0	.4	100	100	97.2	100
	1000	4	1	0	0	.4	100	100	96.7	99
	5000	50	1	0	0	.4	100	100	97.3	100
	2000	20	1	0	0	.4	100	100	96.1	100
	1000	10	1	0	0	.4	100	100	96.9	100
	5000	10	0	1	0	.4	100	100	93.7	100
	2000	4	0	1	0	.4	100	100	94.4	98.7
	1000	2	0	1	0	.4	100	100	94.6	89.8
	5000	20	0	1	0	.4	100	100	94.6	100
	2000	8	0	1	0	.4	100	100	94.4	100
	1000	4	0	1	0	.4	100	100	94.9	98.3
	5000	50	0	1	0	.4	100	100	94	100
	2000	20	0	1	0	.4	100	100	95.5	100
	1000	10	0	1	0	.4	100	100	95.6	100
	5000	10	1	1	0	.4	100	100	100	100
	2000	4	1	1	0	.4	100	100	93.6	99.7
	1000	2	1	1	0	.4	100	100	87.9	89.1
	5000	20	1	1	0	.4	100	100	100	100
	2000	8	1	1	0	.4	100	100	99.5	100
	1000	4	1	1	0	.4	100	100	94.4	99
	5000	50	1	1	0	.4	100	100	100	100
	2000	20	1	1	0	.4	100	100	100	100
	1000	10	1	1	0	.4	100	100	99.9	100
	5000	10	1	1	0	.4	100	100	100	100
	2000	4	1	1	0	.4	100	100	93.6	99.7
	1000	2	1	1	0	.4	100	100	87.9	89.1
	5000	20	1	1	0	.4	100	100	100	100
	2000	8	1	1	0	.4	100	100	99.5	100
	1000	4	1	1	0	.4	100	100	94.4	99
	5000	50	1	1	0	.4	100	100	100	100
	2000	20	1	1	0	.4	100	100	100	100
	1000	10	1	1	0	.4	100	100	99.9	100
	5000	10	2	2	0	.4	100	100	100	100
	2000	4	2	2	0	.4	100	100	93.3	98.8
	1000	2	2	2	0	.4	99.9	100	81.5	90.4
	5000	20	2	2	0	.4	100	100	100	100
	2000	8	2	2	0	.4	100	100	99.6	100
	1000	4	2	2	0	.4	99.9	100	92	98.2
	5000	50	2	2	0	.4	100	100	100	100
	2000	20	2	2	0	.4	100	100	100	100
	1000	10	2	2	0	.4	100	100	100	99.8

SE of convergence

	ssl	ssh	tsq0	tsq1	outc	beta	model1	model2	model3	model4
	5000	10	0	0	0	.4	.2986469	.09995	.5648982	0
	2000	4	0	0	0	.4	.1412799	0	.5216896	.2817091
	1000	2	0	0	0	.4	0	0	.3298333	.9972763
	5000	20	0	0	0	.4	.3582039	0	.5811626	0
	2000	8	0	0	0	.4	.1995996	0	.5216896	.09995
	1000	4	0	0	0	.4	.1995996	0	.4317291	.3146427
	5000	50	0	0	0	.4	.2817091	0	.4937104	0
	2000	20	0	0	0	.4	.1729451	0	.3843826	0
	1000	10	0	0	0	.4	.1995996	0	.4839835	0
	5000	10	1	0	0	.4	0	0	.6270486	0
	2000	4	1	0	0	.4	0	0	.5306505	.3146427
	1000	2	1	0	0	.4	0	0	.5565609	.9401915
	5000	20	1	0	0	.4	0	0	.5394442	0
	2000	8	1	0	0	.4	0	0	.5216896	0
	1000	4	1	0	0	.4	0	0	.5648982	.3146427
	5000	50	1	0	0	.4	0	0	.5125524	0
	2000	20	1	0	0	.4	0	0	.6122009	0
	1000	10	1	0	0	.4	0	0	.5480785	0
	5000	10	0	1	0	.4	0	0	.7683163	0
	2000	4	0	1	0	.4	0	0	.7270763	.3582039
	1000	2	0	1	0	.4	0	0	.7147307	.957058

5000	20	0	1	0	.4	0	0	.7147307	0
2000	8	0	1	0	.4	0	0	.7270763	0
1000	4	0	1	0	.4	0	0	.6956939	.4087909
5000	50	0	1	0	.4	0	0	.7509993	0
2000	20	0	1	0	.4	0	0	.6555532	0
1000	10	0	1	0	.4	0	0	.6485677	0
5000	10	1	1	0	.4	0	0	0	0
2000	4	1	1	0	.4	0	0	.7739767	.1729451
1000	2	1	1	0	.4	0	0	1.031305	.9854897
5000	20	1	1	0	.4	0	0	0	0
2000	8	1	1	0	.4	0	0	.2230471	0
1000	4	1	1	0	.4	0	0	.7270763	.3146427
5000	50	1	1	0	.4	0	0	0	0
2000	20	1	1	0	.4	0	0	0	0
1000	10	1	1	0	.4	0	0	.09995	0
5000	10	1	1	0	.4	0	0	0	0
2000	4	1	1	0	.4	0	0	.7739767	.1729451
1000	2	1	1	0	.4	0	0	1.031305	.9854897
5000	20	1	1	0	.4	0	0	0	0
2000	8	1	1	0	.4	0	0	.2230471	0
1000	4	1	1	0	.4	0	0	.7270763	.3146427
5000	50	1	1	0	.4	0	0	0	0
2000	20	1	1	0	.4	0	0	0	0
1000	10	1	1	0	.4	0	0	.09995	0
5000	10	2	2	0	.4	0	0	0	0
2000	4	2	2	0	.4	0	0	.790639	.3443254
1000	2	2	2	0	.4	.09995	0	1.227905	.9315793
5000	20	2	2	0	.4	0	0	0	0
2000	8	2	2	0	.4	0	0	.1995996	0
1000	4	2	2	0	.4	.09995	0	.8579044	.4204284
5000	50	2	2	0	.4	0	0	0	0
2000	20	2	2	0	.4	0	0	0	0
1000	10	2	2	0	.4	0	0	0	.1412799

Coverage

ssl	ssh	tsq0	tsq1	outc	beta	model1	model2	model3	model4
5000	10	0	0	0	.4	94.55096	94.39439	94.72596	96.1
2000	4	0	0	0	.4	95.29058	95.4	95.37037	96.57258
1000	2	0	0	0	.4	90	90.3	90.19211	95.27027
5000	20	0	0	0	.4	95.94732	95.4	95.54404	96.3
2000	8	0	0	0	.4	93.9759	94	94.34156	95.7958
1000	4	0	0	0	.4	93.27309	93.3	93.17023	96.16162
5000	50	0	0	0	.4	96.06855	95.7	95.89744	95.6
2000	20	0	0	0	.4	93.88164	93.6	94.11168	95.3
1000	10	0	0	0	.4	95.38153	95.1	95.08197	95.1
5000	10	1	0	0	.4	27.8	94.8	93.84776	96.3
2000	4	1	0	0	.4	34.8	95.6	93.92379	96.16162
1000	2	1	0	0	.4	53.2	90.3	87.29339	94.67849
5000	20	1	0	0	.4	35.2	96	96.28866	96.1
2000	8	1	0	0	.4	39.2	94.6	92.59259	95.8
1000	4	1	0	0	.4	45.1	95.7	92.86453	96.36364
5000	50	1	0	0	.4	55.6	95.5	95.68345	96.3
2000	20	1	0	0	.4	53.2	94	93.86056	95.2
1000	10	1	0	0	.4	58.4	94.1	94.53044	95.6
5000	10	0	1	0	.4	94.5	94.1	93.59658	95.4
2000	4	0	1	0	.4	94.9	93.6	93.32627	96.55522
1000	2	0	1	0	.4	83.5	81.5	82.34672	94.87751
5000	20	0	1	0	.4	93.6	94.8	95.66596	96.7
2000	8	0	1	0	.4	93.2	94	92.47881	95.8
1000	4	0	1	0	.4	93.7	91.4	92.41307	94.91353
5000	50	0	1	0	.4	94.9	94.8	95.31915	96.1
2000	20	0	1	0	.4	95.1	94.9	94.7644	96.7
1000	10	0	1	0	.4	94.5	95.3	93.93305	95.2
5000	10	1	1	0	.4	27.2	94.2	93.7	95.4
2000	4	1	1	0	.4	35.9	93.6	91.98718	96.18857
1000	2	1	1	0	.4	47.5	80.9	81.1149	96.74523
5000	20	1	1	0	.4	34.7	94.2	94	95.5
2000	8	1	1	0	.4	36.2	94.6	94.47236	96.5
1000	4	1	1	0	.4	41	92.1	90.7839	95.25253
5000	50	1	1	0	.4	55.2	95.8	95.7	96.2
2000	20	1	1	0	.4	57.1	95.1	95.6	96.2
1000	10	1	1	0	.4	57	94.8	94.09409	96.9
5000	10	1	1	0	.4	27.2	94.2	93.7	95.4
2000	4	1	1	0	.4	35.9	93.6	91.98718	96.18857
1000	2	1	1	0	.4	47.5	80.9	81.1149	96.74523
5000	20	1	1	0	.4	34.7	94.2	94	95.5
2000	8	1	1	0	.4	36.2	94.6	94.47236	96.5
1000	4	1	1	0	.4	41	92.1	90.7839	95.25253
5000	50	1	1	0	.4	55.2	95.8	95.7	96.2
2000	20	1	1	0	.4	57.1	95.1	95.6	96.2
1000	10	1	1	0	.4	57	94.8	94.09409	96.9
5000	10	2	2	0	.4	24	93.6	93.4	96
2000	4	2	2	0	.4	27.2	94	91.85423	96.86235
1000	2	2	2	0	.4	39.53954	82.4	82.45399	95.24336
5000	20	2	2	0	.4	29.6	92.8	93	94.9
2000	8	2	2	0	.4	30.8	95.6	95.78313	96.3
1000	4	2	2	0	.4	32.93293	92.2	91.08696	95.51935
5000	50	2	2	0	.4	48	94.9	94.8	95.4
2000	20	2	2	0	.4	48.2	94.5	94.1	95.7
1000	10	2	2	0	.4	49.7	94.8	94.7	96.49299

SE of coverage

ssl	ssh	tsq0	tsq1	outc	beta	model1	model2	model3	model4
5000	10	0	0	0	.4	.7210347	.7277825	.7187752	.6122009
2000	4	0	0	0	.4	.6705689	.66245	.6739796	.5776366
1000	2	0	0	0	.4	.9486833	.9359006	.9457446	.712345
5000	20	0	0	0	.4	.6276669	.66245	.6642151	.5969171
2000	8	0	0	0	.4	.7539187	.7509993	.7410826	.6349396
1000	4	0	0	0	.4	.7936998	.790639	.8053914	.610601
5000	50	0	0	0	.4	.6170371	.6414905	.6352269	.6485677
2000	20	0	0	0	.4	.759032	.7739767	.7500661	.6692608
1000	10	0	0	0	.4	.665046	.6826346	.6921813	.6826346
5000	10	1	0	0	.4	1.416743	.7021111	.7759243	.5969171
2000	4	1	0	0	.4	1.506307	.6485677	.7666455	.610601
1000	2	1	0	0	.4	1.577897	.9359006	1.070454	.747377
5000	20	1	0	0	.4	1.510285	.6196773	.6069698	.6122009
2000	8	1	0	0	.4	1.543813	.7147307	.8400171	.6343185
1000	4	1	0	0	.4	1.573528	.6414905	.8277954	.5949393
5000	50	1	0	0	.4	1.571191	.6555532	.6515237	.5969171
2000	20	1	0	0	.4	1.577897	.7509993	.7743623	.6759882
1000	10	1	0	0	.4	1.558666	.7451107	.730466	.6485677
5000	10	0	1	0	.4	.7209369	.7451107	.7997717	.66245
2000	4	0	1	0	.4	.6956939	.7739767	.8122697	.5805106
1000	2	0	1	0	.4	1.173776	1.227905	1.239625	.735672
5000	20	0	1	0	.4	.7739767	.7021111	.6620328	.5648982
2000	8	0	1	0	.4	.7960904	.7509993	.8583775	.6343185
1000	4	0	1	0	.4	.7683163	.886589	.8595413	.7008023
5000	50	0	1	0	.4	.6956939	.7021111	.6889514	.6122009
2000	20	0	1	0	.4	.6826346	.6956939	.7207825	.5648982
1000	10	0	1	0	.4	.7209369	.6692608	.7720854	.6759882
5000	10	1	1	0	.4	1.407182	.7391617	.7683163	.66245
2000	4	1	1	0	.4	1.516967	.7739767	.887398	.6063988
1000	2	1	1	0	.4	1.579161	1.243057	1.320126	.5944784
5000	20	1	1	0	.4	1.505294	.7391617	.7509993	.6555532
2000	8	1	1	0	.4	1.519724	.7147307	.7244537	.5811626
1000	4	1	1	0	.4	1.555313	.8529889	.9414387	.6758526
5000	50	1	1	0	.4	1.572565	.6343185	.6414905	.6046156
2000	20	1	1	0	.4	1.565117	.6826346	.6485677	.6046156
1000	10	1	1	0	.4	1.565567	.7021111	.7458332	.5480785
5000	10	1	1	0	.4	1.407182	.7391617	.7683163	.66245
2000	4	1	1	0	.4	1.516967	.7739767	.887398	.6063988
1000	2	1	1	0	.4	1.579161	1.243057	1.320126	.5944784
5000	20	1	1	0	.4	1.505294	.7391617	.7509993	.6555532
2000	8	1	1	0	.4	1.519724	.7147307	.7244537	.5811626
1000	4	1	1	0	.4	1.555313	.8529889	.9414387	.6758526
5000	50	1	1	0	.4	1.572565	.6343185	.6414905	.6046156
2000	20	1	1	0	.4	1.565117	.6826346	.6485677	.6046156
1000	10	1	1	0	.4	1.565567	.7021111	.7458332	.5480785
5000	10	2	2	0	.4	1.350555	.7739767	.7851369	.6196773
2000	4	2	2	0	.4	1.407182	.7509993	.8955189	.5546275
1000	2	2	2	0	.4	1.546923	1.204259	1.332344	.7079183
5000	20	2	2	0	.4	1.443551	.8174105	.8068457	.6956939
2000	8	2	2	0	.4	1.459918	.6485677	.6368099	.5969171
1000	4	2	2	0	.4	1.486918	.848033	.9393927	.6601772
5000	50	2	2	0	.4	1.579873	.6956939	.7021111	.66245
2000	20	2	2	0	.4	1.580114	.7209369	.7451107	.6414905
1000	10	2	2	0	.4	1.58111	.7021111	.7084561	.5823061

Mean error

ssl	ssh	tsq0	tsq1	outc	beta	model1	model2	model3	model4
5000	10	0	0	0	.4	.0468817	.046578	.0471109	.0690514
2000	4	0	0	0	.4	.0838852	.0835803	.0845923	.1298513
1000	2	0	0	0	.4	.1569825	.1565574	.1587338	.1917957
5000	20	0	0	0	.4	.045325	.0454296	.0452898	.06695
2000	8	0	0	0	.4	.0802613	.0805356	.080687	.1144916
1000	4	0	0	0	.4	.1186236	.119365	.1202812	.1705726
5000	50	0	0	0	.4	.0446434	.0445356	.0449606	.0667879
2000	20	0	0	0	.4	.0750771	.0743542	.0748114	.1117392
1000	10	0	0	0	.4	.1056726	.1050219	.1060052	.158411
5000	10	1	0	0	.4	.391248	.0474033	.048521	.0688671
2000	4	1	0	0	.4	.5464137	.0792746	.0846205	.1234945
1000	2	1	0	0	.4	.5186855	.1626729	.1754991	.1932326
5000	20	1	0	0	.4	.2882969	.0441567	.0455529	.065399
2000	8	1	0	0	.4	.4121262	.0777438	.0829089	.1136666
1000	4	1	0	0	.4	.5611664	.1154112	.1293241	.1803849
5000	50	1	0	0	.4	.1727585	.0449803	.0455213	.0674014
2000	20	1	0	0	.4	.2795669	.0740643	.0756763	.1116796
1000	10	1	0	0	.4	.3659798	.1049194	.1077055	.1551283
5000	10	0	1	0	.4	.0587531	.0675714	.0644583	.0701998
2000	4	0	1	0	.4	.109346	.1306213	.1241917	.1247369
1000	2	0	1	0	.4	.2298567	.2545096	.2504126	.2002108
5000	20	0	1	0	.4	.0586539	.0673166	.0611182	.0678562
2000	8	0	1	0	.4	.0930441	.110584	.1051697	.114198
1000	4	0	1	0	.4	.1563658	.180299	.1687583	.1796741
5000	50	0	1	0	.4	.056901	.0657675	.0626928	.0676968
2000	20	0	1	0	.4	.0904619	.1034175	.0960787	.1069124
1000	10	0	1	0	.4	.1319867	.1533783	.1422157	.1587591
5000	10	1	1	0	.4	.3966226	.0714871	.0722698	.0754774
2000	4	1	1	0	.4	.5508911	.1212066	.124417	.1264716
1000	2	1	1	0	.4	.5623321	.267	.2656914	.1907407
5000	20	1	1	0	.4	.2981418	.0677227	.0676204	.0689942

2000	8	1	1	0	.4	.4477367	.1081856	.1086016	.1121519
1000	4	1	1	0	.4	.5708357	.1742442	.1776282	.1739742
5000	50	1	1	0	.4	.1761885	.0663902	.0660694	.0682005
2000	20	1	1	0	.4	.2652475	.1001215	.1000741	.1046311
1000	10	1	1	0	.4	.3857114	.1515382	.1543822	.1551837
5000	10	1	1	0	.4	.3966226	.0714871	.0722698	.0754774
2000	4	1	1	0	.4	.5508911	.1212066	.124417	.1264716
1000	2	1	1	0	.4	.5623321	.267	.2656914	.1907407
5000	20	1	1	0	.4	.2981418	.0677227	.0676204	.0689942
2000	8	1	1	0	.4	.4477367	.1081856	.1086016	.1121519
1000	4	1	1	0	.4	.5708357	.1742442	.1776282	.1739742
5000	50	1	1	0	.4	.1761885	.0663902	.0660694	.0682005
2000	20	1	1	0	.4	.2652475	.1001215	.1000741	.1046311
1000	10	1	1	0	.4	.3857114	.1515382	.1543822	.1551837
5000	10	2	2	0	.4	.5631977	.0707342	.0704436	.0721684
2000	4	2	2	0	.4	.7688935	.1267078	.1323701	.1238041
1000	2	2	2	0	.4	.7663229	.2607963	.2627381	.179247
5000	20	2	2	0	.4	.3951585	.067307	.0673294	.0678957
2000	8	2	2	0	.4	.6076292	.1039022	.1035481	.1068884
1000	4	2	2	0	.4	.8127573	.1922295	.1945282	.1729289
5000	50	2	2	0	.4	.2405993	.064578	.0645518	.0665015
2000	20	2	2	0	.4	.3841884	.1054568	.1059403	.1080998
1000	10	2	2	0	.4	.5318211	.1518483	.1519564	.1557149

SE of mean error

ssl	ssh	tsq0	tsq1	outc	beta	model1	model2	model3	model4
5000	10	0	0	0	.4	.0000366	.0000362	.0000379	.0000571
2000	4	0	0	0	.4	.0000757	.0000756	.0000784	.0001318
1000	2	0	0	0	.4	.0001635	.0001639	.0001665	.000243
5000	20	0	0	0	.4	.0000345	.000034	.000036	.0000502
2000	8	0	0	0	.4	.0000589	.0000592	.000061	.0000945
1000	4	0	0	0	.4	.0000986	.0000981	.0001002	.000159
5000	50	0	0	0	.4	.0000346	.0000345	.0000353	.0000522
2000	20	0	0	0	.4	.0000573	.0000573	.0000585	.0000872
1000	10	0	0	0	.4	.0000773	.0000781	.0000798	.0001199
5000	10	1	0	0	.4	.0003171	.0000362	.0000391	.0000574
2000	4	1	0	0	.4	.0004832	.0000661	.0000741	.0001207
1000	2	1	0	0	.4	.0005856	.0001796	.0001952	.0002489
5000	20	1	0	0	.4	.0002195	.0000337	.0000355	.0000522
2000	8	1	0	0	.4	.0003285	.0000595	.0000674	.0000937
1000	4	1	0	0	.4	.0004805	.0000975	.0001098	.0001762
5000	50	1	0	0	.4	.0001284	.0000337	.0000351	.0000513
2000	20	1	0	0	.4	.0002068	.0000555	.0000588	.0000829
1000	10	1	0	0	.4	.0002847	.0000801	.0000849	.0001219
5000	10	0	1	0	.4	.0000472	.0000568	.0000566	.0000581
2000	4	0	1	0	.4	.0001525	.0001676	.0001752	.0001238
1000	2	0	1	0	.4	.0003358	.000366	.0003856	.0002664
5000	20	0	1	0	.4	.0000451	.000052	.0000502	.0000522
2000	8	0	1	0	.4	.0000799	.0001003	.0000989	.000104
1000	4	0	1	0	.4	.000145	.0001661	.0001667	.0001634
5000	50	0	1	0	.4	.0000423	.0000493	.0000486	.0000508
2000	20	0	1	0	.4	.0000688	.0000791	.0000772	.0000821
1000	10	0	1	0	.4	.0001031	.0001216	.0001168	.0001255
5000	10	1	1	0	.4	.0003095	.0000544	.0000557	.0001121
2000	4	1	1	0	.4	.0005175	.0001351	.0001404	.0001324
1000	2	1	1	0	.4	.0005623	.0003757	.0004339	.0002336
5000	20	1	1	0	.4	.0002322	.0000508	.0000512	.0000528
2000	8	1	1	0	.4	.0003554	.0000834	.0000855	.0000864
1000	4	1	1	0	.4	.0004542	.0001836	.0002032	.000169
5000	50	1	1	0	.4	.0001328	.0000492	.0000486	.0000499
2000	20	1	1	0	.4	.0002015	.0000792	.0000785	.0000821
1000	10	1	1	0	.4	.0003028	.0001179	.0001206	.0001188
5000	10	1	1	0	.4	.0003095	.0000544	.0000557	.0001121
2000	4	1	1	0	.4	.0005175	.0001351	.0001404	.0001324
1000	2	1	1	0	.4	.0005623	.0003757	.0004339	.0002336
5000	20	1	1	0	.4	.0002322	.0000508	.0000512	.0000528
2000	8	1	1	0	.4	.0003554	.0000834	.0000855	.0000864
1000	4	1	1	0	.4	.0004542	.0001836	.0002032	.000169
5000	50	1	1	0	.4	.0001328	.0000492	.0000486	.0000499
2000	20	1	1	0	.4	.0002015	.0000792	.0000785	.0000821
1000	10	1	1	0	.4	.0003028	.0001179	.0001206	.0001188
5000	10	2	2	0	.4	.00046	.0000553	.0000559	.0000557
2000	4	2	2	0	.4	.0006891	.000175	.0001966	.0001276
1000	2	2	2	0	.4	.0008583	.0004878	.0006202	.0002217
5000	20	2	2	0	.4	.0002966	.0000542	.0000544	.0000537
2000	8	2	2	0	.4	.0004865	.0000837	.0000834	.0000898
1000	4	2	2	0	.4	.0006971	.0002736	.0002982	.0001737
5000	50	2	2	0	.4	.0001781	.0000498	.0000498	.0000501
2000	20	2	2	0	.4	.0002997	.0000799	.0000798	.000082
1000	10	2	2	0	.4	.0004176	.0001253	.0001249	.0001205

Mean bias

ssl	ssh	tsq0	tsq1	outc	beta	model1	model2	model3	model4
5000	10	0	0	0	.4	-.00095	-.0011602	-.001257	-.0062837
2000	4	0	0	0	.4	-.0004766	-.0010908	-.0003878	-.001496
1000	2	0	0	0	.4	-.0178209	-.0182317	-.0167886	.0018813
5000	20	0	0	0	.4	-.0001219	.0004029	-.000246	.0018245

2000	8	0	0	0	.4	.0056315	.0057168	.0049832	.0042877
1000	4	0	0	0	.4	.0040672	.0041431	.002007	-.0025888
5000	50	0	0	0	.4	-.0002561	-.0006499	-.0003764	.0024965
2000	20	0	0	0	.4	-.0048009	-.0057151	-.0050005	-.0067749
1000	10	0	0	0	.4	-.0011116	-.0004935	-.0010366	.0002873
5000	10	1	0	0	.4	.0008126	.0002056	.0003202	.0039466
2000	4	1	0	0	.4	-.0006676	1.64e-06	.0026423	.0010884
1000	2	1	0	0	.4	-.0449639	-.0194626	-.024196	-.003669
5000	20	1	0	0	.4	-.0086506	.0007665	.0004094	.001046
2000	8	1	0	0	.4	.0276664	.0015886	.0024872	.0122846
1000	4	1	0	0	.4	.0064483	.0021882	.0039358	.0057896
5000	50	1	0	0	.4	.0001881	.000297	.0007488	.0027842
2000	20	1	0	0	.4	-.0030763	-.0053094	-.0050632	-.0074307
1000	10	1	0	0	.4	-.023117	-.0016568	-.0044321	-.0104995
5000	10	0	1	0	.4	-.001256	-.0028434	-.0031362	-.0036979
2000	4	0	1	0	.4	.0056557	.0072798	.0057605	.005865
1000	2	0	1	0	.4	-.0068401	.000921	-.0057337	.0071895
5000	20	0	1	0	.4	-.0016873	-.0032134	-.0009732	-.0038674
2000	8	0	1	0	.4	.0050613	.0024338	.0052175	.0018693
1000	4	0	1	0	.4	-.0083234	-.0090287	-.0128026	-.002297
5000	50	0	1	0	.4	.002039	.0026836	.0014865	.0028312
2000	20	0	1	0	.4	-.0054395	-.0063834	-.0072341	-.0037797
1000	10	0	1	0	.4	-.0043342	-.0034584	-.0025541	-.0044158
5000	10	1	1	0	.4	-.0130998	-.0026509	-.0026932	-.0071992
2000	4	1	1	0	.4	.01709	-.0056338	-.0070609	-.0081935
1000	2	1	1	0	.4	-.0102232	-.0192289	-.0118062	-.0111517
5000	20	1	1	0	.4	.0068999	.0036501	.0038678	.0036524
2000	8	1	1	0	.4	.0162013	.0039175	.0045686	.0044047
1000	4	1	1	0	.4	-.0277469	-.0003489	-.0019667	.0110677
5000	50	1	1	0	.4	-.0034299	.0036167	.0038116	.0032765
2000	20	1	1	0	.4	.0062226	-.0006826	.0003482	-.0023931
1000	10	1	1	0	.4	-.0056972	-.0025416	-.0036734	.0013115
5000	10	1	1	0	.4	-.0130998	-.0026509	-.0026932	-.0071992
2000	4	1	1	0	.4	.01709	-.0056338	-.0070609	-.0081935
1000	2	1	1	0	.4	-.0102232	-.0192289	-.0118062	-.0111517
5000	20	1	1	0	.4	.0068999	.0036501	.0038678	.0036524
2000	8	1	1	0	.4	.0162013	.0039175	.0045686	.0044047
1000	4	1	1	0	.4	-.0277469	-.0003489	-.0019667	.0110677
5000	50	1	1	0	.4	-.0034299	.0036167	.0038116	.0032765
2000	20	1	1	0	.4	.0062226	-.0006826	.0003482	-.0023931
1000	10	1	1	0	.4	-.0056972	-.0025416	-.0036734	.0013115
5000	10	2	2	0	.4	-.0072846	.002196	.0023729	.0022481
2000	4	2	2	0	.4	-.0395919	-.015772	-.0153938	-.0094319
1000	2	2	2	0	.4	.0165608	.0035917	.0039229	.0102104
5000	20	2	2	0	.4	.0080106	.0014817	.0015586	.00102
2000	8	2	2	0	.4	-.030395	-.0027986	-.0036191	-.001459
1000	4	2	2	0	.4	.0335374	.007155	.0101727	.0068247
5000	50	2	2	0	.4	-.0092004	-.0010127	-.0011561	-.000075
2000	20	2	2	0	.4	.0021659	.0045488	.0047373	.0047307
1000	10	2	2	0	.4	-.0157349	.0065967	.0037352	.0063733

SE of mean bias

ssl	ssh	tsq0	tsq1	outc	beta	model1	model2	model3	model4
5000	10	0	0	0	.4	.0000598	.000059	.0000617	.0000894
2000	4	0	0	0	.4	.0001132	.0001127	.0001172	.0001858
1000	2	0	0	0	.4	.000226	.000226	.0002307	.0003252
5000	20	0	0	0	.4	.0000574	.0000568	.0000592	.0000837
2000	8	0	0	0	.4	.0000997	.0000998	.0001029	.0001485
1000	4	0	0	0	.4	.0001546	.0001545	.0001584	.0002345
5000	50	0	0	0	.4	.0000568	.0000564	.0000581	.0000847
2000	20	0	0	0	.4	.0000945	.0000937	.0000958	.0001416
1000	10	0	0	0	.4	.0001313	.0001309	.0001348	.0001987
5000	10	1	0	0	.4	.0005038	.0000597	.000064	.0000896
2000	4	1	0	0	.4	.0007296	.0001032	.0001144	.0001736
1000	2	1	0	0	.4	.0007811	.0002416	.0002653	.0003284
5000	20	1	0	0	.4	.0003624	.0000555	.0000589	.0000837
2000	8	1	0	0	.4	.0005264	.0000979	.0001087	.0001469
1000	4	1	0	0	.4	.000739	.0001511	.0001731	.0002535
5000	50	1	0	0	.4	.0002153	.0000562	.0000585	.0000847
2000	20	1	0	0	.4	.0003478	.0000924	.0000981	.0001389
1000	10	1	0	0	.4	.0004632	.000132	.0001398	.0001971
5000	10	0	1	0	.4	.0000754	.0000882	.0000891	.0000911
2000	4	0	1	0	.4	.0001876	.0002124	.0002191	.0001768
1000	2	0	1	0	.4	.0004069	.0004459	.0004677	.0003474
5000	20	0	1	0	.4	.000074	.0000851	.0000819	.0000856
2000	8	0	1	0	.4	.0001226	.0001493	.0001489	.0001545
1000	4	0	1	0	.4	.0002131	.000245	.0002435	.0002452
5000	50	0	1	0	.4	.0000709	.0000822	.0000826	.0000846
2000	20	0	1	0	.4	.0001136	.0001301	.0001267	.0001348
1000	10	0	1	0	.4	.0001675	.0001958	.0001892	.0002024
5000	10	1	1	0	.4	.0005031	.0000898	.0000912	.000135
2000	4	1	1	0	.4	.0007559	.0001815	.0001932	.0001832
1000	2	1	1	0	.4	.0007954	.0004606	.0005287	.0003167
5000	20	1	1	0	.4	.0003779	.0000846	.0000848	.0000868
2000	8	1	1	0	.4	.0005716	.0001366	.0001386	.0001416
1000	4	1	1	0	.4	.0007292	.0002532	.000277	.0002436
5000	50	1	1	0	.4	.0002207	.0000826	.000082	.0000845
2000	20	1	1	0	.4	.0003332	.0001277	.0001272	.000133
1000	10	1	1	0	.4	.0004905	.000192	.0001961	.0001955
5000	10	1	1	0	.4	.0005031	.0000898	.0000912	.000135
2000	4	1	1	0	.4	.0007559	.0001815	.0001932	.0001832
1000	2	1	1	0	.4	.0007954	.0004606	.0005287	.0003167
5000	20	1	1	0	.4	.0003779	.0000846	.0000848	.0000868
2000	8	1	1	0	.4	.0005716	.0001366	.0001386	.0001416

1000	4	1	1	0	.4	.0007292	.0002532	.000277	.0002436
5000	50	1	1	0	.4	.0002207	.0000826	.000082	.0000845
2000	20	1	1	0	.4	.0003332	.0001277	.0001272	.000133
1000	10	1	1	0	.4	.0004905	.000192	.0001961	.0001955
5000	10	2	2	0	.4	.0007274	.0000898	.00009	.0000912
2000	4	2	2	0	.4	.0010321	.0002155	.0002419	.0001787
1000	2	2	2	0	.4	.0011513	.0005532	.0006991	.0002973
5000	20	2	2	0	.4	.0004942	.0000864	.0000865	.0000866
2000	8	2	2	0	.4	.000778	.0001335	.0001333	.0001396
1000	4	2	2	0	.4	.0010712	.0003344	.0003655	.0002474
5000	50	2	2	0	.4	.0002993	.0000816	.0000815	.0000833
2000	20	2	2	0	.4	.0004874	.0001323	.0001326	.0001356
1000	10	2	2	0	.4	.0006762	.0001968	.0001967	.0001971

Power

ssl	ssh	tsq0	tsq1	outc	beta	model1	model2	model3	model4
5000	10	0	0	0	.4	91.3219	91.69169	90.79628	56.5
2000	4	0	0	0	.4	52.90581	53.1	53.39506	28.02419
1000	2	0	0	0	.4	24.9	25.1	25.07583	18.80631
5000	20	0	0	0	.4	93.61702	93.9	92.95337	62.3
2000	8	0	0	0	.4	57.02811	58.2	57.09877	27.52753
1000	4	0	0	0	.4	30.12048	31.2	30.17329	15.35354
5000	50	0	0	0	.4	93.54839	94	93.33333	66.1
2000	20	0	0	0	.4	56.36911	57.9	55.63452	27.9
1000	10	0	0	0	.4	34.73896	35.1	33.09426	15.3
5000	10	1	0	0	.4	53.1	91.9	90.3024	59.6
2000	4	1	0	0	.4	47.2	51.7	50.25747	27.47475
1000	2	1	0	0	.4	33.6	25.6	26.13636	18.62528
5000	20	1	0	0	.4	53	93.9	92.78351	62.3
2000	8	1	0	0	.4	46.3	58.7	55.96708	29.4
1000	4	1	0	0	.4	40.9	31	32.05791	15.75758
5000	50	1	0	0	.4	56.8	95.3	94.96403	64
2000	20	1	0	0	.4	42.9	58.4	55.67118	27.8
1000	10	1	0	0	.4	34	31.8	29.92776	16.2
5000	10	0	1	0	.4	78.1	67.4	71.82497	58.5
2000	4	0	1	0	.4	41.4	37.2	40.57203	28.77406
1000	2	0	1	0	.4	22.3	23.1	22.83298	16.03563
5000	20	0	1	0	.4	80	68.1	74.84144	61.2
2000	8	0	1	0	.4	42	33.7	39.51271	28.1
1000	4	0	1	0	.4	22.9	20.6	21.49631	15.15768
5000	50	0	1	0	.4	80.3	68.6	74.78723	63.5
2000	20	0	1	0	.4	41.4	32.6	36.02094	26.2
1000	10	0	1	0	.4	24.3	21.8	23.11715	15.9
5000	10	1	1	0	.4	52	65	64.7	57.7
2000	4	1	1	0	.4	47.7	34.3	36.21795	27.98395
1000	2	1	1	0	.4	37.6	24.9	26.1661	17.62065
5000	20	1	1	0	.4	53.8	69.1	69	61.9
2000	8	1	1	0	.4	46.9	36	36.88442	30.1
1000	4	1	1	0	.4	39.2	19.4	21.39831	13.53535
5000	50	1	1	0	.4	54.3	68.9	69.8	62.9
2000	20	1	1	0	.4	42.9	33.6	34.5	28.3
1000	10	1	1	0	.4	36.4	20.2	20.62062	15.1
5000	10	1	1	0	.4	52	65	64.7	57.7
2000	4	1	1	0	.4	47.7	34.3	36.21795	27.98395
1000	2	1	1	0	.4	37.6	24.9	26.1661	17.62065
5000	20	1	1	0	.4	53.8	69.1	69	61.9
2000	8	1	1	0	.4	46.9	36	36.88442	30.1
1000	4	1	1	0	.4	39.2	19.4	21.39831	13.53535
5000	50	1	1	0	.4	54.3	68.9	69.8	62.9
2000	20	1	1	0	.4	42.9	33.6	34.5	28.3
1000	10	1	1	0	.4	36.4	20.2	20.62062	15.1
5000	10	2	2	0	.4	51.2	68.1	68.4	59.8
2000	4	2	2	0	.4	45.3	32	34.51233	25.20243
1000	2	2	2	0	.4	38.03804	25.4	27.48466	19.13717
5000	20	2	2	0	.4	51.8	69.1	68.4	62.8
2000	8	2	2	0	.4	44.1	32.9	33.53414	26.5
1000	4	2	2	0	.4	41.44144	21.2	22.5	14.86762
5000	50	2	2	0	.4	49	69.8	69.5	65.2
2000	20	2	2	0	.4	40.4	34.9	34.5	29.1
1000	10	2	2	0	.4	35.3	21.5	21.6	16.73347

SE of power

ssl	ssh	tsq0	tsq1	outc	beta	model1	model2	model3	model4
5000	10	0	0	0	.4	.8942584	.8732499	.9296138	1.567721
2000	4	0	0	0	.4	1.580047	1.578097	1.600049	1.425949
1000	2	0	0	0	.4	1.367476	1.371127	1.37829	1.311313
5000	20	0	0	0	.4	.7780912	.7568289	.8238719	1.53255
2000	8	0	0	0	.4	1.568581	1.559731	1.587505	1.413147
1000	4	0	0	0	.4	1.453705	1.465114	1.465506	1.145753
5000	50	0	0	0	.4	.7800033	.7509993	.7988596	1.496927
2000	20	0	0	0	.4	1.570616	1.561278	1.582984	1.418305
1000	10	0	0	0	.4	1.50871	1.509301	1.506202	1.13838
5000	10	1	0	0	.4	1.578097	.8627804	.955592	1.551722
2000	4	1	0	0	.4	1.578658	1.580225	1.604555	1.418711
1000	2	1	0	0	.4	1.493667	1.380087	1.412213	1.296262
5000	20	1	0	0	.4	1.57829	.7568289	.830831	1.53255
2000	8	1	0	0	.4	1.576804	1.55702	1.592289	1.440708

1000	4	1	0	0	.4	1.554731	1.462532	1.500803	1.157957
5000	50	1	0	0	.4	1.566448	.6692608	.7010755	1.517893
2000	20	1	0	0	.4	1.565117	1.558666	1.602495	1.416743
1000	10	1	0	0	.4	1.497999	1.472671	1.47112	1.165144
5000	10	0	1	0	.4	1.307819	1.482309	1.469603	1.558124
2000	4	0	1	0	.4	1.557575	1.52845	1.598169	1.44099
1000	2	0	1	0	.4	1.316324	1.332813	1.364745	1.224482
5000	20	0	1	0	.4	1.264911	1.473903	1.41081	1.540961
2000	8	0	1	0	.4	1.560769	1.494761	1.591162	1.421404
1000	4	0	1	0	.4	1.328755	1.278921	1.333503	1.143789
5000	50	0	1	0	.4	1.25774	1.467665	1.416316	1.522416
2000	20	0	1	0	.4	1.557575	1.482309	1.553441	1.390525
1000	10	0	1	0	.4	1.356285	1.305665	1.363493	1.156369
5000	10	1	1	0	.4	1.579873	1.50831	1.511261	1.562277
2000	4	1	1	0	.4	1.579465	1.50117	1.570989	1.421745
1000	2	1	1	0	.4	1.531744	1.367476	1.482528	1.276384
5000	20	1	1	0	.4	1.576566	1.461229	1.462532	1.535705
2000	8	1	1	0	.4	1.578097	1.517893	1.529601	1.450514
1000	4	1	1	0	.4	1.543813	1.250456	1.334811	1.087268
5000	50	1	1	0	.4	1.575281	1.463827	1.451882	1.527609
2000	20	1	1	0	.4	1.565117	1.493667	1.503246	1.424468
1000	10	1	1	0	.4	1.521526	1.26963	1.280035	1.13225
5000	10	1	1	0	.4	1.579873	1.50831	1.511261	1.562277
2000	4	1	1	0	.4	1.579465	1.50117	1.570989	1.421745
1000	2	1	1	0	.4	1.531744	1.367476	1.482528	1.276384
5000	20	1	1	0	.4	1.576566	1.461229	1.462532	1.535705
2000	8	1	1	0	.4	1.578097	1.517893	1.529601	1.450514
1000	4	1	1	0	.4	1.543813	1.250456	1.334811	1.087268
5000	50	1	1	0	.4	1.575281	1.463827	1.451882	1.527609
2000	20	1	1	0	.4	1.565117	1.493667	1.503246	1.424468
1000	10	1	1	0	.4	1.521526	1.26963	1.280035	1.13225
5000	10	2	2	0	.4	1.580683	1.473903	1.470184	1.550471
2000	4	2	2	0	.4	1.574138	1.475127	1.556417	1.381295
1000	2	2	2	0	.4	1.535992	1.376532	1.5638	1.308366
5000	20	2	2	0	.4	1.580114	1.461229	1.470184	1.52845
2000	8	2	2	0	.4	1.570092	1.485796	1.495937	1.395618
1000	4	2	2	0	.4	1.558583	1.292501	1.376728	1.135304
5000	50	2	2	0	.4	1.580823	1.451882	1.455936	1.506307
2000	20	2	2	0	.4	1.551722	1.507312	1.503246	1.436381
1000	10	2	2	0	.4	1.511261	1.299134	1.301322	1.181579

Model 1: Fixed common intercept; random treatment effect; Fixed effect for baseline
Model 2: Fixed study-specific intercepts; random treatment effect; Fixed study-specific effects for baseline
Model 3: Random study intercept; random treatment effect; fixed study-specific effects for baseline
Model 4: two-stage IPD with ipdmetan

ss1: size for lower level unit (patients)
ssh: size for higher level unit (studies)
tsq0: between study variance for the intercept
tsq1: between study variance for the exposure
outc: outcome type (0=continuous)
beta: true effect size

Interaction effect (bXb), with heterogeneity for both covariate & interaction (same analytic models)

Convergence

ssl	ssh	tsq0	tsq1	outc	beta	model1	model2	model3	model4
5000	10	0	0	0	.4	100	100	88.7	100
2000	4	0	0	0	.4	100	100	86.9	100
1000	2	0	0	0	.4	100	100	93.8	100
5000	20	0	0	0	.4	100	100	95.5	100
2000	8	0	0	0	.4	100	100	87.8	100
1000	4	0	0	0	.4	100	100	87.9	100
5000	50	0	0	0	.4	100	100	97	100
2000	20	0	0	0	.4	100	100	93.4	100
1000	10	0	0	0	.4	100	100	88	100
5000	10	1	0	0	.4	100	100	100	100
2000	4	1	0	0	.4	100	100	99.1	100
1000	2	1	0	0	.4	100	100	91.2	100
5000	20	1	0	0	.4	100	100	100	100
2000	8	1	0	0	.4	100	100	100	100
1000	4	1	0	0	.4	100	100	98.8	100
5000	50	1	0	0	.4	100	100	100	100
2000	20	1	0	0	.4	100	100	100	100
1000	10	1	0	0	.4	100	100	99.9	100
5000	10	0	1	0	.4	100	100	99.9	100
2000	4	0	1	0	.4	100	100	98.1	100
1000	2	0	1	0	.4	100	100	91	100
5000	20	0	1	0	.4	100	100	99.9	100
2000	8	0	1	0	.4	100	100	99.4	100
1000	4	0	1	0	.4	100	100	96.1	100
5000	50	0	1	0	.4	100	100	100	100
2000	20	0	1	0	.4	100	100	99.9	100
1000	10	0	1	0	.4	100	100	97.5	100
5000	10	1	1	0	.4	100	100	100	100
2000	4	1	1	0	.4	100	100	99.3	100
1000	2	1	1	0	.4	100	100	86	100
5000	20	1	1	0	.4	100	100	100	100
2000	8	1	1	0	.4	100	100	100	100
1000	4	1	1	0	.4	100	100	98.7	100
5000	50	1	1	0	.4	100	100	100	100
2000	20	1	1	0	.4	100	100	100	100
1000	10	1	1	0	.4	100	100	100	100
5000	10	1	1	0	.4	100	100	100	100
2000	4	1	1	0	.4	100	100	99.3	100
1000	2	1	1	0	.4	100	100	86	100
5000	20	1	1	0	.4	100	100	100	100
2000	8	1	1	0	.4	100	100	100	100
1000	4	1	1	0	.4	100	100	98.7	100
5000	50	1	1	0	.4	100	100	100	100
2000	20	1	1	0	.4	100	100	100	100
1000	10	1	1	0	.4	100	100	100	100
5000	10	2	2	0	.4	100	100	100	100
2000	4	2	2	0	.4	100	100	99.7	100
1000	2	2	2	0	.4	100	100	77.6	100
5000	20	2	2	0	.4	100	100	100	100
2000	8	2	2	0	.4	100	100	100	100
1000	4	2	2	0	.4	100	100	99	100
5000	50	2	2	0	.4	100	100	100	100
2000	20	2	2	0	.4	100	100	100	100
1000	10	2	2	0	.4	100	100	100	100

SE of convergence

ssl	ssh	tsq0	tsq1	outc	beta	model1	model2	model3	model4
5000	10	0	0	0	.4	0	0	1.001154	0
2000	4	0	0	0	.4	0	0	1.066954	0
1000	2	0	0	0	.4	0	0	.7626008	0
5000	20	0	0	0	.4	0	0	.6555532	0
2000	8	0	0	0	.4	0	0	1.034969	0
1000	4	0	0	0	.4	0	0	1.031305	0
5000	50	0	0	0	.4	0	0	.5394442	0
2000	20	0	0	0	.4	0	0	.7851369	0
1000	10	0	0	0	.4	0	0	1.027619	0
5000	10	1	0	0	.4	0	0	0	0
2000	4	1	0	0	.4	0	0	.2986469	0
1000	2	1	0	0	.4	0	0	.8958571	0
5000	20	1	0	0	.4	0	0	0	0
2000	8	1	0	0	.4	0	0	0	0
1000	4	1	0	0	.4	0	0	.3443254	0
5000	50	1	0	0	.4	0	0	0	0
2000	20	1	0	0	.4	0	0	0	0
1000	10	1	0	0	.4	0	0	.09995	0
5000	10	0	1	0	.4	0	0	.09995	0
2000	4	0	1	0	.4	0	0	.4317291	0
1000	2	0	1	0	.4	0	0	.9049862	0

5000	20	0	1	0	.4	0	0	.09995	0
2000	8	0	1	0	.4	0	0	.244213	0
1000	4	0	1	0	.4	0	0	.6122009	0
5000	50	0	1	0	.4	0	0	0	0
2000	20	0	1	0	.4	0	0	.09995	0
1000	10	0	1	0	.4	0	0	.4937104	0
5000	10	1	1	0	.4	0	0	0	0
2000	4	1	1	0	.4	0	0	.2636475	0
1000	2	1	1	0	.4	0	0	1.097269	0
5000	20	1	1	0	.4	0	0	0	0
2000	8	1	1	0	.4	0	0	0	0
1000	4	1	1	0	.4	0	0	.3582039	0
5000	50	1	1	0	.4	0	0	0	0
2000	20	1	1	0	.4	0	0	0	0
1000	10	1	1	0	.4	0	0	0	0
5000	10	1	1	0	.4	0	0	0	0
2000	4	1	1	0	.4	0	0	.2636475	0
1000	2	1	1	0	.4	0	0	1.097269	0
5000	20	1	1	0	.4	0	0	0	0
2000	8	1	1	0	.4	0	0	0	0
1000	4	1	1	0	.4	0	0	.3582039	0
5000	50	1	1	0	.4	0	0	0	0
2000	20	1	1	0	.4	0	0	0	0
1000	10	1	1	0	.4	0	0	0	0
5000	10	2	2	0	.4	0	0	0	0
2000	4	2	2	0	.4	0	0	.1729451	0
1000	2	2	2	0	.4	0	0	1.318423	0
5000	20	2	2	0	.4	0	0	0	0
2000	8	2	2	0	.4	0	0	0	0
1000	4	2	2	0	.4	0	0	.3146427	0
5000	50	2	2	0	.4	0	0	0	0
2000	20	2	2	0	.4	0	0	0	0
1000	10	2	2	0	.4	0	0	0	0

Coverage

ssl	ssh	tsq0	tsq1	outc	beta	model1	model2	model3	model4
5000	10	0	0	0	.4	29.5	25.5	25.81736	91.7
2000	4	0	0	0	.4	26.3	23.7	22.78481	84.2
1000	2	0	0	0	.4	23.6	22.8	23.13433	68.7
5000	20	0	0	0	.4	42.4	36.8	36.23037	93.3
2000	8	0	0	0	.4	42.3	36.1	35.19362	91.8
1000	4	0	0	0	.4	37.6	33.2	33.21957	84.7
5000	50	0	0	0	.4	61.2	53.7	54.3299	93.4
2000	20	0	0	0	.4	63.8	55.7	56.20985	92.9
1000	10	0	0	0	.4	62.7	55.4	54.43182	92.3
5000	10	1	0	0	.4	33.7	26.1	26.1	92.8
2000	4	1	0	0	.4	30.4	25	24.92432	86.6
1000	2	1	0	0	.4	30.4	26.4	26.86404	69.3
5000	20	1	0	0	.4	49	36.9	36.8	93.6
2000	8	1	0	0	.4	46.3	36.1	36.3	91.2
1000	4	1	0	0	.4	43.1	34.9	34.81781	85.6
5000	50	1	0	0	.4	68.1	55.2	55.3	96.1
2000	20	1	0	0	.4	64.7	52.2	52.1	93.2
1000	10	1	0	0	.4	62.4	50.7	51.15115	91.2
5000	10	0	1	0	.4	31.7	26.6	26.62663	93.1
2000	4	0	1	0	.4	29.7	26.4	26.09582	85.1
1000	2	0	1	0	.4	28.2	25.9	26.26374	70
5000	20	0	1	0	.4	38.9	33.6	33.63363	92.3
2000	8	0	1	0	.4	40	36	35.91549	90.8
1000	4	0	1	0	.4	41.1	36.2	36.4204	85.5
5000	50	0	1	0	.4	61.7	53.2	53.7	93.2
2000	20	0	1	0	.4	59.7	53	53.15315	92.7
1000	10	0	1	0	.4	60.1	51.7	52.41026	92.4
5000	10	1	1	0	.4	33.9	25.6	25.8	91.7
2000	4	1	1	0	.4	31.7	25.3	25.27694	86.6
1000	2	1	1	0	.4	27.4	24.4	24.4186	73.5
5000	20	1	1	0	.4	47.5	36.9	36.9	94.1
2000	8	1	1	0	.4	47.3	36.5	36.8	91.1
1000	4	1	1	0	.4	40.7	34.9	35.15704	84.1
5000	50	1	1	0	.4	66.9	53.7	54.1	95
2000	20	1	1	0	.4	65.6	51.2	51.3	94.1
1000	10	1	1	0	.4	68.6	56.3	56.9	93.2
5000	10	1	1	0	.4	33.9	25.6	25.8	91.7
2000	4	1	1	0	.4	31.7	25.3	25.27694	86.6
1000	2	1	1	0	.4	27.4	24.4	24.4186	73.5
5000	20	1	1	0	.4	47.5	36.9	36.9	94.1
2000	8	1	1	0	.4	47.3	36.5	36.8	91.1
1000	4	1	1	0	.4	40.7	34.9	35.15704	84.1
5000	50	1	1	0	.4	66.9	53.7	54.1	95
2000	20	1	1	0	.4	65.6	51.2	51.3	94.1
1000	10	1	1	0	.4	68.6	56.3	56.9	93.2
5000	10	2	2	0	.4	35.7	23.4	23.4	91.6
2000	4	2	2	0	.4	34	27.1	27.08124	87.8
1000	2	2	2	0	.4	30	26.3	27.57732	71.5
5000	20	2	2	0	.4	49.3	33.5	33.6	93.5
2000	8	2	2	0	.4	49	35.9	36	90.3
1000	4	2	2	0	.4	44.2	33.5	33.43434	85.4
5000	50	2	2	0	.4	68.9	55.3	55.4	95.3
2000	20	2	2	0	.4	70	53.4	53.6	93
1000	10	2	2	0	.4	68.8	52.5	52.5	90.7

SE of coverage

ssl	ssh	tsq0	tsq1	outc	beta	model1	model2	model3	model4
5000	10	0	0	0	.4	1.442134	1.378314	1.469418	.8724162
2000	4	0	0	0	.4	1.392232	1.344734	1.422867	1.153412
1000	2	0	0	0	.4	1.342773	1.32671	1.376871	1.466394
5000	20	0	0	0	.4	1.562767	1.525044	1.555398	.790639
2000	8	0	0	0	.4	1.562277	1.518812	1.611735	.8676174
1000	4	0	0	0	.4	1.531744	1.489215	1.588646	1.13838
5000	50	0	0	0	.4	1.540961	1.576804	1.599372	.7851369
2000	20	0	0	0	.4	1.519724	1.570831	1.623383	.8121515
1000	10	0	0	0	.4	1.529284	1.571891	1.678866	.8430362
5000	10	1	0	0	.4	1.494761	1.388809	1.388809	.8174105
2000	4	1	0	0	.4	1.454593	1.369306	1.374119	1.077237
1000	2	1	0	0	.4	1.454593	1.39393	1.467755	1.458599
5000	20	1	0	0	.4	1.580823	1.525906	1.525044	.7739767
2000	8	1	0	0	.4	1.576804	1.518812	1.520628	.8958571
1000	4	1	0	0	.4	1.566011	1.507312	1.515607	1.110243
5000	50	1	0	0	.4	1.473903	1.572565	1.572231	.6122009
2000	20	1	0	0	.4	1.511261	1.579608	1.579744	.7960904
1000	10	1	0	0	.4	1.531744	1.580984	1.581511	.8958571
5000	10	0	1	0	.4	1.471431	1.397297	1.398442	.8014924
2000	4	0	1	0	.4	1.44496	1.39393	1.402121	1.126051
1000	2	0	1	0	.4	1.422941	1.385348	1.458808	1.449138
5000	20	0	1	0	.4	1.541684	1.493667	1.494783	.8430362
2000	8	0	1	0	.4	1.549193	1.517893	1.521683	.9139803
1000	4	0	1	0	.4	1.555889	1.519724	1.552278	1.113441
5000	50	0	1	0	.4	1.537241	1.577897	1.576804	.7960904
2000	20	0	1	0	.4	1.5511	1.57829	1.578781	.8226239
1000	10	0	1	0	.4	1.548544	1.580225	1.59942	.8379976
5000	10	1	1	0	.4	1.496927	1.380087	1.383604	.8724162
2000	4	1	1	0	.4	1.471431	1.37474	1.379161	1.077237
1000	2	1	1	0	.4	1.410404	1.358175	1.464936	1.395618
5000	20	1	1	0	.4	1.579161	1.525906	1.525906	.7451107
2000	8	1	1	0	.4	1.578832	1.522416	1.525044	.9004388
1000	4	1	1	0	.4	1.553548	1.507312	1.519774	1.156369
5000	50	1	1	0	.4	1.488083	1.576804	1.575814	.6892024
2000	20	1	1	0	.4	1.502212	1.580683	1.580604	.7451107
1000	10	1	1	0	.4	1.467665	1.568538	1.566011	.7960904
5000	10	1	1	0	.4	1.496927	1.380087	1.383604	.8724162
2000	4	1	1	0	.4	1.471431	1.37474	1.379161	1.077237
1000	2	1	1	0	.4	1.410404	1.358175	1.464936	1.395618
5000	20	1	1	0	.4	1.579161	1.525906	1.525906	.7451107
2000	8	1	1	0	.4	1.578832	1.522416	1.525044	.9004388
1000	4	1	1	0	.4	1.553548	1.507312	1.519774	1.156369
5000	50	1	1	0	.4	1.488083	1.576804	1.575814	.6892024
2000	20	1	1	0	.4	1.502212	1.580683	1.580604	.7451107
1000	10	1	1	0	.4	1.467665	1.568538	1.566011	.7960904
5000	10	2	2	0	.4	1.515094	1.33882	1.33882	.8771773
2000	4	2	2	0	.4	1.497999	1.405557	1.407364	1.034969
1000	2	2	2	0	.4	1.449138	1.392232	1.604288	1.427498
5000	20	2	2	0	.4	1.580984	1.492565	1.493667	.7795832
2000	8	2	2	0	.4	1.580823	1.516967	1.517893	.9359006
1000	4	2	2	0	.4	1.570465	1.492565	1.499353	1.11662
5000	50	2	2	0	.4	1.463827	1.572231	1.571891	.6692608
2000	20	2	2	0	.4	1.449138	1.577479	1.577035	.8068457
1000	10	2	2	0	.4	1.465114	1.579161	1.579161	.918428

Mean error

ssl	ssh	tsq0	tsq1	outc	beta	model1	model2	model3	model4
5000	10	0	0	0	.4	.2768576	.2770768	.2770046	.2501287
2000	4	0	0	0	.4	.4495579	.4491865	.4504685	.410152
1000	2	0	0	0	.4	.6503547	.6496542	.6459233	.5907899
5000	20	0	0	0	.4	.1963984	.1954346	.1943976	.1828043
2000	8	0	0	0	.4	.3172627	.3177304	.3177753	.2894014
1000	4	0	0	0	.4	.459035	.4598473	.4582614	.4263124
5000	50	0	0	0	.4	.1256846	.1246828	.1241855	.1212339
2000	20	0	0	0	.4	.1917446	.1932554	.1929311	.1928265
1000	10	0	0	0	.4	.2730686	.2698389	.2714526	.2650661
5000	10	1	0	0	.4	.2884816	.2865958	.2865997	.2537803
2000	4	1	0	0	.4	.4448521	.4415586	.4427732	.4031823
1000	2	1	0	0	.4	.6284109	.6246718	.6229984	.5576118
5000	20	1	0	0	.4	.1963065	.1955093	.1955065	.1793482
2000	8	1	0	0	.4	.3117647	.3062757	.3062283	.2848025
1000	4	1	0	0	.4	.4506125	.4483995	.4465163	.4088002
5000	50	1	0	0	.4	.1254375	.121214	.1212033	.1162067
2000	20	1	0	0	.4	.2098148	.2026067	.2025521	.1987167
1000	10	1	0	0	.4	.2994568	.2848573	.2849102	.2776634
5000	10	0	1	0	.4	.2798957	.2799109	.2794222	.2557012
2000	4	0	1	0	.4	.4543568	.454717	.4540237	.4025763
1000	2	0	1	0	.4	.6379631	.6381269	.6404678	.5810531
5000	20	0	1	0	.4	.2082949	.2078244	.2078745	.1921208
2000	8	0	1	0	.4	.3255003	.324312	.3250019	.2969464
1000	4	0	1	0	.4	.4251269	.4247454	.424348	.394504
5000	50	0	1	0	.4	.1261943	.1256125	.1255837	.1230963
2000	20	0	1	0	.4	.2034418	.201772	.201863	.1955194
1000	10	0	1	0	.4	.2844841	.2815804	.282154	.2758419
5000	10	1	1	0	.4	.2918757	.288422	.2884197	.2608642
2000	4	1	1	0	.4	.4505562	.4499117	.4506548	.4184877
1000	2	1	1	0	.4	.6429309	.6384649	.6390778	.5782692
5000	20	1	1	0	.4	.1975572	.1945517	.1945558	.1759154

2000	8	1	1	0	.4	.3108371	.3060171	.3060684	.285201
1000	4	1	1	0	.4	.4509239	.447803	.4465411	.4211639
5000	50	1	1	0	.4	.1310783	.124788	.1248314	.1215536
2000	20	1	1	0	.4	.2059219	.1992126	.1992116	.1946649
1000	10	1	1	0	.4	.2742498	.2642665	.2641223	.2569796
5000	10	1	1	0	.4	.2918757	.288422	.2884197	.2608642
2000	4	1	1	0	.4	.4505562	.4499117	.4506548	.4184877
1000	2	1	1	0	.4	.6429309	.6384649	.6390778	.5782692
5000	20	1	1	0	.4	.1975572	.1945517	.1945558	.1759154
2000	8	1	1	0	.4	.3108371	.3060171	.3060684	.285201
1000	4	1	1	0	.4	.4509239	.447803	.4465411	.4211639
5000	50	1	1	0	.4	.1310783	.124788	.1248314	.1215536
2000	20	1	1	0	.4	.2059219	.1992126	.1992116	.1946649
1000	10	1	1	0	.4	.2742498	.2642665	.2641223	.2569796
5000	10	2	2	0	.4	.289833	.2865679	.2865707	.2605984
2000	4	2	2	0	.4	.447093	.4407944	.4408085	.3937332
1000	2	2	2	0	.4	.6212268	.6162465	.6064694	.5624045
5000	20	2	2	0	.4	.2070937	.2005246	.2005289	.1870829
2000	8	2	2	0	.4	.3228975	.3144401	.3144403	.2980716
1000	4	2	2	0	.4	.4554837	.4436382	.444479	.4096263
5000	50	2	2	0	.4	.1355719	.1242861	.1243091	.1200297
2000	20	2	2	0	.4	.2126918	.1996374	.1996037	.1921658
1000	10	2	2	0	.4	.3041757	.2889633	.2888519	.2801915

SE of mean error

ssl	ssh	tsq0	tsq1	outc	beta	model1	model2	model3	model4
5000	10	0	0	0	.4	.0002139	.0002144	.0002455	.0001896
2000	4	0	0	0	.4	.000336	.0003367	.0003865	.0003048
1000	2	0	0	0	.4	.000481	.0004803	.0005135	.0004278
5000	20	0	0	0	.4	.000155	.0001539	.0001609	.0001433
2000	8	0	0	0	.4	.0002468	.0002475	.0002768	.0002184
1000	4	0	0	0	.4	.0003509	.0003474	.0003956	.0003236
5000	50	0	0	0	.4	.0000948	.0000935	.0000966	.0000934
2000	20	0	0	0	.4	.0001544	.0001529	.0001652	.0001486
1000	10	0	0	0	.4	.0002052	.0002069	.0002369	.0001996
5000	10	1	0	0	.4	.0002183	.000219	.000219	.0001909
2000	4	1	0	0	.4	.0003345	.0003325	.0003361	.0003041
1000	2	1	0	0	.4	.0004784	.0004752	.0005152	.0004257
5000	20	1	0	0	.4	.0001535	.0001509	.0001509	.0001426
2000	8	1	0	0	.4	.0002404	.0002391	.0002391	.0002155
1000	4	1	0	0	.4	.0003437	.0003393	.0003391	.0003151
5000	50	1	0	0	.4	.0000956	.0000919	.0000919	.0000879
2000	20	1	0	0	.4	.0001632	.0001544	.0001545	.0001493
1000	10	1	0	0	.4	.0002181	.0002078	.0002079	.0002018
5000	10	0	1	0	.4	.0002148	.0002139	.0002136	.0001878
2000	4	0	1	0	.4	.0003575	.0003578	.0003629	.0003165
1000	2	0	1	0	.4	.0004925	.0004922	.0005418	.0004387
5000	20	0	1	0	.4	.0001556	.0001548	.0001551	.0001443
2000	8	0	1	0	.4	.0002537	.0002534	.0002554	.0002246
1000	4	0	1	0	.4	.0003204	.0003191	.0003293	.0002957
5000	50	0	1	0	.4	.0000977	.0000969	.0000967	.0000956
2000	20	0	1	0	.4	.0001536	.0001518	.0001522	.0001493
1000	10	0	1	0	.4	.0002101	.0002101	.0002162	.0002034
5000	10	1	1	0	.4	.0002302	.0002278	.0002278	.0002068
2000	4	1	1	0	.4	.0003452	.0003429	.0003453	.0003151
1000	2	1	1	0	.4	.0004918	.000489	.0005676	.0004361
5000	20	1	1	0	.4	.000151	.0001493	.0001493	.0001366
2000	8	1	1	0	.4	.0002391	.0002366	.0002366	.0002238
1000	4	1	1	0	.4	.0003342	.0003348	.0003392	.0003159
5000	50	1	1	0	.4	.0000991	.0000951	.0000951	.0000921
2000	20	1	1	0	.4	.0001565	.0001457	.0001458	.0001416
1000	10	1	1	0	.4	.0002158	.00021	.0002098	.0001991
5000	10	1	1	0	.4	.0002302	.0002278	.0002278	.0002068
2000	4	1	1	0	.4	.0003452	.0003429	.0003453	.0003151
1000	2	1	1	0	.4	.0004918	.000489	.0005676	.0004361
5000	20	1	1	0	.4	.000151	.0001493	.0001493	.0001366
2000	8	1	1	0	.4	.0002391	.0002366	.0002366	.0002238
1000	4	1	1	0	.4	.0003342	.0003348	.0003392	.0003159
5000	50	1	1	0	.4	.0000991	.0000951	.0000951	.0000921
2000	20	1	1	0	.4	.0001565	.0001457	.0001458	.0001416
1000	10	1	1	0	.4	.0002158	.00021	.0002098	.0001991
5000	10	2	2	0	.4	.0002214	.0002197	.0002197	.0002008
2000	4	2	2	0	.4	.000333	.0003336	.000335	.0002985
1000	2	2	2	0	.4	.0004781	.0004794	.0006266	.0004274
5000	20	2	2	0	.4	.0001575	.0001523	.0001523	.0001415
2000	8	2	2	0	.4	.0002509	.0002457	.0002457	.0002251
1000	4	2	2	0	.4	.0003343	.000323	.0003264	.0003051
5000	50	2	2	0	.4	.0000997	.0000935	.0000935	.0000906
2000	20	2	2	0	.4	.0001616	.0001523	.0001523	.0001452
1000	10	2	2	0	.4	.0002371	.000219	.000219	.0002148

Mean bias

ssl	ssh	tsq0	tsq1	outc	beta	model1	model2	model3	model4
5000	10	0	0	0	.4	-.0063261	-.0056937	-.0144767	-.0058766
2000	4	0	0	0	.4	.0065653	.0051554	.0185464	.0030955
1000	2	0	0	0	.4	-.0264864	-.0267726	-.0249351	-.014512
5000	20	0	0	0	.4	-.0081237	-.0088302	-.0099947	-.0055067

2000	8	0	0	0	.4	.0009263	.0019451	-.0055319	.0050563
1000	4	0	0	0	.4	.0136162	.0128647	.0030821	.0192494
5000	50	0	0	0	.4	-.0022109	-.000977	-.0003256	.0006957
2000	20	0	0	0	.4	.0013309	.0018749	-.0005765	.0012073
1000	10	0	0	0	.4	-.0124987	-.0122031	-.005972	-.0125046
5000	10	1	0	0	.4	-.0138634	-.0148778	-.0148876	-.0165769
2000	4	1	0	0	.4	.0185994	.0191364	.0191419	.012349
1000	2	1	0	0	.4	-.0003734	-.0022547	-.0130556	-.0088019
5000	20	1	0	0	.4	.0129073	.0090739	.0090862	.0089127
2000	8	1	0	0	.4	.0477436	.0489783	.0490134	.0447897
1000	4	1	0	0	.4	-.0020797	.0039051	-.00029	-.0014017
5000	50	1	0	0	.4	-.0010072	-7.44e-06	-8.05e-07	-.0020484
2000	20	1	0	0	.4	.0172583	.020471	.0204393	.0198763
1000	10	1	0	0	.4	.0076876	.009461	.0094828	.0083833
5000	10	0	1	0	.4	-.013814	-.0139668	-.0132073	-.0180584
2000	4	0	1	0	.4	-.0201986	-.0193132	-.0163273	-.0119111
1000	2	0	1	0	.4	-.0043126	-.0049638	.0004141	.0029497
5000	20	0	1	0	.4	-.0059443	-.0062651	-.0064855	-.0025363
2000	8	0	1	0	.4	-.0035703	-.0040487	-.0033673	.0016763
1000	4	0	1	0	.4	-.0292873	-.0282247	-.0346938	-.0168992
5000	50	0	1	0	.4	-.0056449	-.0053295	-.0054013	-.0051262
2000	20	0	1	0	.4	.0043363	.0046724	.0047111	.0026809
1000	10	0	1	0	.4	-.0134838	-.0142168	-.0163567	-.0077928
5000	10	1	1	0	.4	.0101728	.0100686	.0100674	.0101064
2000	4	1	1	0	.4	-.0380839	-.0348198	-.0356286	-.0250738
1000	2	1	1	0	.4	.0474622	.0421632	.0541809	.025347
5000	20	1	1	0	.4	-.0018053	-.0008897	-.0008872	-.0002472
2000	8	1	1	0	.4	-.0063667	-.0050255	-.0050369	-.0050643
1000	4	1	1	0	.4	-.0215176	-.024209	-.0260335	-.0290066
5000	50	1	1	0	.4	-.0049312	-.0047339	-.0047519	-.0029504
2000	20	1	1	0	.4	-.0010472	-.005239	-.005184	-.0037276
1000	10	1	1	0	.4	-.0008213	-.0058233	-.0058595	-.0070626
5000	10	1	1	0	.4	.0101728	.0100686	.0100674	.0101064
2000	4	1	1	0	.4	-.0380839	-.0348198	-.0356286	-.0250738
1000	2	1	1	0	.4	.0474622	.0421632	.0541809	.025347
5000	20	1	1	0	.4	-.0018053	-.0008897	-.0008872	-.0002472
2000	8	1	1	0	.4	-.0063667	-.0050255	-.0050369	-.0050643
1000	4	1	1	0	.4	-.0215176	-.024209	-.0260335	-.0290066
5000	50	1	1	0	.4	-.0049312	-.0047339	-.0047519	-.0029504
2000	20	1	1	0	.4	-.0010472	-.005239	-.005184	-.0037276
1000	10	1	1	0	.4	-.0008213	-.0058233	-.0058595	-.0070626
5000	10	2	2	0	.4	-.0103922	-.0097007	-.0096979	-.016026
2000	4	2	2	0	.4	-.0014339	-.0014391	-.0013941	.004774
1000	2	2	2	0	.4	.0204244	.0176878	.0209104	.0273876
5000	20	2	2	0	.4	.0160866	.0161753	.0161751	.0098697
2000	8	2	2	0	.4	.0164263	.0170856	.0171043	.0163915
1000	4	2	2	0	.4	.0263423	.0228037	.0227706	.0116932
5000	50	2	2	0	.4	-.0017519	-.0006775	-.0006763	-.0015243
2000	20	2	2	0	.4	-.0120891	-.0071114	-.0071537	-.0077583
1000	10	2	2	0	.4	.0286049	.0219259	.0219993	.021896

SE of mean bias

ssl	ssh	tsq0	tsq1	outc	beta	model1	model2	model3	model4
5000	10	0	0	0	.4	.0003499	.0003504	.0003971	.0003139
2000	4	0	0	0	.4	.0005614	.0005615	.0006465	.0005112
1000	2	0	0	0	.4	.0008087	.0008078	.0008589	.0007295
5000	20	0	0	0	.4	.0002501	.0002487	.0002594	.0002323
2000	8	0	0	0	.4	.0004021	.0004029	.0004558	.0003626
1000	4	0	0	0	.4	.0005778	.0005764	.0006547	.000535
5000	50	0	0	0	.4	.0001574	.0001559	.0001604	.0001531
2000	20	0	0	0	.4	.0002463	.0002465	.0002646	.0002435
1000	10	0	0	0	.4	.0003414	.0003399	.000389	.0003317
5000	10	1	0	0	.4	.0003616	.0003605	.0003605	.0003173
2000	4	1	0	0	.4	.0005565	.0005526	.000559	.000505
1000	2	1	0	0	.4	.00079	.0007851	.0008558	.0007017
5000	20	1	0	0	.4	.000249	.0002469	.0002469	.000229
2000	8	1	0	0	.4	.0003909	.0003856	.0003855	.0003544
1000	4	1	0	0	.4	.0005669	.0005625	.0005652	.0005163
5000	50	1	0	0	.4	.0001577	.0001522	.0001522	.0001457
2000	20	1	0	0	.4	.0002653	.000254	.000254	.0002479
1000	10	1	0	0	.4	.0003705	.0003526	.0003529	.0003433
5000	10	0	1	0	.4	.0003527	.0003521	.0003518	.0003169
2000	4	0	1	0	.4	.0005779	.0005784	.0005881	.0005121
1000	2	0	1	0	.4	.0008062	.0008062	.0008885	.0007283
5000	20	0	1	0	.4	.00026	.0002592	.0002595	.0002404
2000	8	0	1	0	.4	.0004128	.0004117	.000415	.0003725
1000	4	0	1	0	.4	.0005317	.0005307	.0005498	.0004929
5000	50	0	1	0	.4	.0001596	.0001586	.0001585	.0001558
2000	20	0	1	0	.4	.000255	.0002525	.000253	.0002461
1000	10	0	1	0	.4	.0003535	.0003511	.000361	.0003428
5000	10	1	1	0	.4	.0003717	.0003675	.0003675	.0003329
2000	4	1	1	0	.4	.0005665	.0005648	.0005693	.0005234
1000	2	1	1	0	.4	.0008083	.0008033	.0009333	.000724
5000	20	1	1	0	.4	.0002487	.0002453	.0002453	.0002228
2000	8	1	1	0	.4	.0003922	.0003869	.0003869	.0003626
1000	4	1	1	0	.4	.0005611	.0005588	.000565	.0005259
5000	50	1	1	0	.4	.0001643	.0001569	.0001569	.0001525
2000	20	1	1	0	.4	.0002587	.0002469	.0002469	.0002408
1000	10	1	1	0	.4	.0003491	.0003376	.0003374	.0003251
5000	10	1	1	0	.4	.0003717	.0003675	.0003675	.0003329
2000	4	1	1	0	.4	.0005665	.0005648	.0005693	.0005234
1000	2	1	1	0	.4	.0008083	.0008033	.0009333	.000724
5000	20	1	1	0	.4	.0002487	.0002453	.0002453	.0002228
2000	8	1	1	0	.4	.0003922	.0003869	.0003869	.0003626

1000	4	1	1	0	.4	.0005611	.0005588	.000565	.0005259
5000	50	1	1	0	.4	.0001643	.0001569	.0001569	.0001525
2000	20	1	1	0	.4	.0002587	.0002469	.0002469	.0002408
1000	10	1	1	0	.4	.0003491	.0003376	.0003374	.0003251
5000	10	2	2	0	.4	.0003647	.0003611	.0003611	.0003287
2000	4	2	2	0	.4	.0005576	.000553	.0005549	.0004942
1000	2	2	2	0	.4	.0007839	.0007808	.0010017	.0007061
5000	20	2	2	0	.4	.0002597	.0002514	.0002514	.0002344
2000	8	2	2	0	.4	.0004087	.0003988	.0003988	.0003732
1000	4	2	2	0	.4	.0005645	.0005485	.0005548	.0005108
5000	50	2	2	0	.4	.0001684	.0001556	.0001556	.0001504
2000	20	2	2	0	.4	.0002669	.0002511	.0002511	.0002408
1000	10	2	2	0	.4	.0003847	.000362	.0003619	.0003525

Power

ssl	ssh	tsq0	tsq1	outc	beta	model1	model2	model3	model4
5000	10	0	0	0	.4	56.7	58.8	57.8354	11.8
2000	4	0	0	0	.4	51.2	52.9	53.16456	15.5
1000	2	0	0	0	.4	46.2	47.5	47.54797	21.2
5000	20	0	0	0	.4	59.2	62.3	62.30366	13.9
2000	8	0	0	0	.4	51.5	54.3	53.53075	10.5
1000	4	0	0	0	.4	44.5	47.3	46.18885	13.7
5000	50	0	0	0	.4	65.1	69.9	70	25.9
2000	20	0	0	0	.4	46.8	52.8	52.46253	13.8
1000	10	0	0	0	.4	37.6	43.2	43.52273	11.4
5000	10	1	0	0	.4	53.6	57.7	57.7	10.2
2000	4	1	0	0	.4	49.7	53.2	53.0777	13.9
1000	2	1	0	0	.4	44.3	46.5	45.50439	21.2
5000	20	1	0	0	.4	57.4	64.1	64.1	15.7
2000	8	1	0	0	.4	50	57.1	57	12.7
1000	4	1	0	0	.4	41.3	46.2	46.05263	13.2
5000	50	1	0	0	.4	60.5	71.9	71.6	25.6
2000	20	1	0	0	.4	45.6	58.1	57.7	17
1000	10	1	0	0	.4	37.1	45.2	45.24525	12.7
5000	10	0	1	0	.4	57.1	59.9	59.95996	11.4
2000	4	0	1	0	.4	46.9	48.4	48.21611	12.3
1000	2	0	1	0	.4	44.9	46	45.82418	19.5
5000	20	0	1	0	.4	60.7	62.6	62.56256	15.8
2000	8	0	1	0	.4	49.4	51.8	51.81087	10.2
1000	4	0	1	0	.4	41.7	45.2	44.22477	13
5000	50	0	1	0	.4	65.3	69.5	69.3	24.4
2000	20	0	1	0	.4	49.1	53.8	53.95395	14.5
1000	10	0	1	0	.4	39.1	43.9	43.28205	10.7
5000	10	1	1	0	.4	56.8	61.5	61.5	12
2000	4	1	1	0	.4	45.9	49.4	49.34542	12.9
1000	2	1	1	0	.4	47.3	48.9	49.30233	20.5
5000	20	1	1	0	.4	58.1	64.2	64.2	14.6
2000	8	1	1	0	.4	47.1	51.9	51.8	12.1
1000	4	1	1	0	.4	39.6	43.3	43.26241	12.7
5000	50	1	1	0	.4	58.4	69	68.9	24
2000	20	1	1	0	.4	42.3	52.2	52.3	14.2
1000	10	1	1	0	.4	33.5	41.1	40.9	9.7
5000	10	1	1	0	.4	56.8	61.5	61.5	12
2000	4	1	1	0	.4	45.9	49.4	49.34542	12.9
1000	2	1	1	0	.4	47.3	48.9	49.30233	20.5
5000	20	1	1	0	.4	58.1	64.2	64.2	14.6
2000	8	1	1	0	.4	47.1	51.9	51.8	12.1
1000	4	1	1	0	.4	39.6	43.3	43.26241	12.7
5000	50	1	1	0	.4	58.4	69	68.9	24
2000	20	1	1	0	.4	42.3	52.2	52.3	14.2
1000	10	1	1	0	.4	33.5	41.1	40.9	9.7
5000	10	2	2	0	.4	51.9	57.8	57.8	10.5
2000	4	2	2	0	.4	47	52	51.85557	12.5
1000	2	2	2	0	.4	47.2	49.3	48.96907	20.8
5000	20	2	2	0	.4	57.6	64.6	64.6	16.9
2000	8	2	2	0	.4	45.2	54.6	54.5	10.8
1000	4	2	2	0	.4	41.1	47.4	47.47475	12.8
5000	50	2	2	0	.4	54.1	71	70.8	24.4
2000	20	2	2	0	.4	36.8	52.5	52.5	12.7
1000	10	2	2	0	.4	33.7	46.7	46.6	13.5

SE of power

ssl	ssh	tsq0	tsq1	outc	beta	model1	model2	model3	model4
5000	10	0	0	0	.4	1.566879	1.556458	1.658094	1.020176
2000	4	0	0	0	.4	1.580683	1.578477	1.692733	1.144443
1000	2	0	0	0	.4	1.576566	1.579161	1.630594	1.292501
5000	20	0	0	0	.4	1.554143	1.53255	1.568212	1.093979
2000	8	0	0	0	.4	1.580427	1.575281	1.683206	.969407
1000	4	0	0	0	.4	1.571544	1.578832	1.681552	1.087341
5000	50	0	0	0	.4	1.507312	1.450514	1.471376	1.385348
2000	20	0	0	0	.4	1.577897	1.578658	1.634065	1.09067
1000	10	0	0	0	.4	1.531744	1.566448	1.671297	1.005007
5000	10	1	0	0	.4	1.577035	1.562277	1.562277	.957058
2000	4	1	0	0	.4	1.58111	1.577897	1.585291	1.093979
1000	2	1	0	0	.4	1.570831	1.57726	1.648959	1.292501
5000	20	1	0	0	.4	1.563726	1.516967	1.516967	1.150439
2000	8	1	0	0	.4	1.581139	1.565117	1.565567	1.052953

1000	4	1	0	0	.4	1.55702	1.576566	1.585747	1.070402
5000	50	1	0	0	.4	1.545882	1.421404	1.425987	1.380087
2000	20	1	0	0	.4	1.575005	1.560253	1.562277	1.187855
1000	10	1	0	0	.4	1.527609	1.573836	1.574761	1.052953
5000	10	0	1	0	.4	1.565117	1.549835	1.550227	1.005007
2000	4	0	1	0	.4	1.578097	1.580329	1.595361	1.03861
1000	2	0	1	0	.4	1.572892	1.576071	1.651693	1.252897
5000	20	0	1	0	.4	1.54451	1.530111	1.531185	1.153412
2000	8	0	1	0	.4	1.581025	1.580114	1.584863	.957058
1000	4	0	1	0	.4	1.559202	1.573836	1.602108	1.063485
5000	50	0	1	0	.4	1.505294	1.455936	1.458599	1.358175
2000	20	0	1	0	.4	1.580883	1.576566	1.576976	1.113441
1000	10	0	1	0	.4	1.54311	1.569328	1.586762	.9775019
5000	10	1	1	0	.4	1.566448	1.538749	1.538749	1.027619
2000	4	1	1	0	.4	1.575814	1.581025	1.586566	1.059995
1000	2	1	1	0	.4	1.578832	1.580756	1.70482	1.276617
5000	20	1	1	0	.4	1.560253	1.516034	1.516034	1.11662
2000	8	1	1	0	.4	1.578477	1.579997	1.580114	1.031305
1000	4	1	1	0	.4	1.546557	1.566879	1.577002	1.052953
5000	50	1	1	0	.4	1.558666	1.462532	1.463827	1.350555
2000	20	1	1	0	.4	1.562277	1.579608	1.579465	1.103793
1000	10	1	1	0	.4	1.492565	1.555889	1.554731	.9359006
5000	10	1	1	0	.4	1.566448	1.538749	1.538749	1.027619
2000	4	1	1	0	.4	1.575814	1.581025	1.586566	1.059995
1000	2	1	1	0	.4	1.578832	1.580756	1.70482	1.276617
5000	20	1	1	0	.4	1.560253	1.516034	1.516034	1.11662
2000	8	1	1	0	.4	1.578477	1.579997	1.580114	1.031305
1000	4	1	1	0	.4	1.546557	1.566879	1.577002	1.052953
5000	50	1	1	0	.4	1.558666	1.462532	1.463827	1.350555
2000	20	1	1	0	.4	1.562277	1.579608	1.579465	1.103793
1000	10	1	1	0	.4	1.492565	1.555889	1.554731	.9359006
5000	10	2	2	0	.4	1.579997	1.561781	1.561781	.969407
2000	4	2	2	0	.4	1.57829	1.579873	1.582425	1.045825
1000	2	2	2	0	.4	1.578658	1.580984	1.794514	1.283495
5000	20	2	2	0	.4	1.562767	1.51223	1.51223	1.18507
2000	8	2	2	0	.4	1.573836	1.574433	1.574722	.981509
1000	4	2	2	0	.4	1.555889	1.579	1.587076	1.056485
5000	50	2	2	0	.4	1.575814	1.434922	1.437832	1.358175
2000	20	2	2	0	.4	1.525044	1.579161	1.579161	1.052953
1000	10	2	2	0	.4	1.494761	1.577691	1.577479	1.080625

Model 1: Fixed common intercept; random treatment effect; Fixed effect for baseline
Model 2: Fixed study-specific intercepts; random treatment effect; Fixed study-specific effects for baseline
Model 3: Random study intercept; random treatment effect; fixed study-specific effects for baseline
Model 4: two-stage IPD with ipdmetan

ss1: size for lower level unit (patients)
ssh: size for higher level unit (studies)
tsq0: between study variance for the intercept
tsq1: between study variance for the exposure
outc: outcome type (0=continuous)
beta: true effect size

Interaction effect (bXb), with heterogeneity for both covariate & interaction (different models 1-3)

Convergence

ssl	ssh	tsq0	tsq1	outc	beta	model1	model2	model3	model4
5000	10	0	0	0	.4	99.9	98.2	97.6	100
2000	4	0	0	0	.4	100	99.5	99.1	100
1000	2	0	0	0	.4	100	100	99.9	100
5000	20	0	0	0	.4	100	98.4	97.5	100
2000	8	0	0	0	.4	100	98.9	99.3	100
1000	4	0	0	0	.4	100	99.8	99.5	100
5000	50	0	0	0	.4	100	98.8	96.5	100
2000	20	0	0	0	.4	100	99.6	98.8	100
1000	10	0	0	0	.4	100	99.7	99.3	100
5000	10	1	0	0	.4	100	99.7	99.2	100
2000	4	1	0	0	.4	100	99.6	99.7	100
1000	2	1	0	0	.4	100	99.9	99.9	100
5000	20	1	0	0	.4	100	99.5	99	100
2000	8	1	0	0	.4	100	99.6	99.6	100
1000	4	1	0	0	.4	100	99.9	99.6	100
5000	50	1	0	0	.4	100	99.2	99.1	100
2000	20	1	0	0	.4	100	99.7	99.7	100
1000	10	1	0	0	.4	100	99.9	100	100
5000	10	0	1	0	.4	100	99.7	99	100
2000	4	0	1	0	.4	100	100	99.3	100
1000	2	0	1	0	.4	100	100	99.7	100
5000	20	0	1	0	.4	100	100	99	100
2000	8	0	1	0	.4	100	100	98.4	100
1000	4	0	1	0	.4	100	99.9	99.9	100
5000	50	0	1	0	.4	100	100	98.7	100
2000	20	0	1	0	.4	100	99.9	98.8	100
1000	10	0	1	0	.4	100	100	99	100
5000	10	1	1	0	.4	100	100	100	100
2000	4	1	1	0	.4	100	100	99.8	100
1000	2	1	1	0	.4	100	100	99.8	100
5000	20	1	1	0	.4	100	100	100	100
2000	8	1	1	0	.4	100	100	100	100
1000	4	1	1	0	.4	100	100	99.9	100
5000	50	1	1	0	.4	100	100	100	100
2000	20	1	1	0	.4	100	100	100	100
1000	10	1	1	0	.4	100	100	100	100
5000	10	1	1	0	.4	100	100	100	100
2000	4	1	1	0	.4	100	100	99.8	100
1000	2	1	1	0	.4	100	100	99.8	100
5000	20	1	1	0	.4	100	100	100	100
2000	8	1	1	0	.4	100	100	100	100
1000	4	1	1	0	.4	100	100	99.9	100
5000	50	1	1	0	.4	100	100	100	100
2000	20	1	1	0	.4	100	100	100	100
1000	10	1	1	0	.4	100	100	100	100
5000	10	2	2	0	.4	100	100	100	100
2000	4	2	2	0	.4	100	99.9	99.9	100
1000	2	2	2	0	.4	100	100	99.6	100
5000	20	2	2	0	.4	100	100	100	100
2000	8	2	2	0	.4	100	100	100	100
1000	4	2	2	0	.4	100	100	100	100
5000	50	2	2	0	.4	100	100	100	100
2000	20	2	2	0	.4	100	100	100	100
1000	10	2	2	0	.4	99.9	100	100	100

SE of convergence

ssl	ssh	tsq0	tsq1	outc	beta	model1	model2	model3	model4
5000	10	0	0	0	.4	.09995	.4204284	.4839835	0
2000	4	0	0	0	.4	0	.2230471	.2986469	0
1000	2	0	0	0	.4	0	0	.09995	0
5000	20	0	0	0	.4	0	.3967871	.4937104	0
2000	8	0	0	0	.4	0	.3298333	.2636475	0
1000	4	0	0	0	.4	0	.1412799	.2230471	0
5000	50	0	0	0	.4	0	.3443254	.5811626	0
2000	20	0	0	0	.4	0	.1995996	.3443254	0
1000	10	0	0	0	.4	0	.1729451	.2636475	0
5000	10	1	0	0	.4	0	.1729451	.2817091	0
2000	4	1	0	0	.4	0	.1995996	.1729451	0
1000	2	1	0	0	.4	0	.09995	.09995	0
5000	20	1	0	0	.4	0	.2230471	.3146427	0
2000	8	1	0	0	.4	0	.1995996	.1995996	0
1000	4	1	0	0	.4	0	.09995	.1995996	0
5000	50	1	0	0	.4	0	.2817091	.2986469	0
2000	20	1	0	0	.4	0	.1729451	.1729451	0
1000	10	1	0	0	.4	0	.09995	0	0
5000	10	0	1	0	.4	0	.1729451	.3146427	0
2000	4	0	1	0	.4	0	0	.2636475	0
1000	2	0	1	0	.4	0	0	.1729451	0

5000	20	0	1	0	.4	0	0	.3146427	0
2000	8	0	1	0	.4	0	0	.3967871	0
1000	4	0	1	0	.4	0	.09995	.09995	0
5000	50	0	1	0	.4	0	0	.3582039	0
2000	20	0	1	0	.4	0	.09995	.3443254	0
1000	10	0	1	0	.4	0	0	.3146427	0
5000	10	1	1	0	.4	0	0	0	0
2000	4	1	1	0	.4	0	0	.1412799	0
1000	2	1	1	0	.4	0	0	.1412799	0
5000	20	1	1	0	.4	0	0	0	0
2000	8	1	1	0	.4	0	0	0	0
1000	4	1	1	0	.4	0	0	.09995	0
5000	50	1	1	0	.4	0	0	0	0
2000	20	1	1	0	.4	0	0	0	0
1000	10	1	1	0	.4	0	0	0	0
5000	10	1	1	0	.4	0	0	0	0
2000	4	1	1	0	.4	0	0	.1412799	0
1000	2	1	1	0	.4	0	0	.1412799	0
5000	20	1	1	0	.4	0	0	0	0
2000	8	1	1	0	.4	0	0	0	0
1000	4	1	1	0	.4	0	0	.09995	0
5000	50	1	1	0	.4	0	0	0	0
2000	20	1	1	0	.4	0	0	0	0
1000	10	1	1	0	.4	0	0	0	0
5000	10	2	2	0	.4	0	0	0	0
2000	4	2	2	0	.4	0	.09995	.09995	0
1000	2	2	2	0	.4	0	0	.1995996	0
5000	20	2	2	0	.4	0	0	0	0
2000	8	2	2	0	.4	0	0	0	0
1000	4	2	2	0	.4	0	0	0	0
5000	50	2	2	0	.4	0	0	0	0
2000	20	2	2	0	.4	0	0	0	0
1000	10	2	2	0	.4	.09995	0	0	0

Coverage

ssl	ssh	tsq0	tsq1	outc	beta	model1	model2	model3	model4
5000	10	0	0	0	.4	91.39139	91.0387	91.4959	91.7
2000	4	0	0	0	.4	80.2	80.30151	80.82745	84.2
1000	2	0	0	0	.4	58.7	58.8	59.35936	68.7
5000	20	0	0	0	.4	92.1	92.07317	92.41026	93.3
2000	8	0	0	0	.4	89.9	89.78766	90.43303	91.8
1000	4	0	0	0	.4	80.8	80.96192	81.60804	84.7
5000	50	0	0	0	.4	93	93.11741	93.67876	93.4
2000	20	0	0	0	.4	92.1	91.96787	92.71255	92.9
1000	10	0	0	0	.4	90.7	90.37111	91.44008	92.3
5000	10	1	0	0	.4	91.7	91.47442	91.73387	92.8
2000	4	1	0	0	.4	81.5	81.4257	81.74524	86.6
1000	2	1	0	0	.4	60.5	60.36036	61.36136	69.3
5000	20	1	0	0	.4	92.8	92.86432	93.0303	93.6
2000	8	1	0	0	.4	89.1	89.35743	90.06024	91.2
1000	4	1	0	0	.4	79.9	80.18018	81.62651	85.6
5000	50	1	0	0	.4	94.9	95.06048	95.56004	96.1
2000	20	1	0	0	.4	92.7	92.97894	93.58074	93.2
1000	10	1	0	0	.4	88.8	88.98899	89.9	91.2
5000	10	0	1	0	.4	90.9	90.97292	91.0101	93.1
2000	4	0	1	0	.4	79.8	80.8	81.36959	85.1
1000	2	0	1	0	.4	59.4	60.8	61.18355	70
5000	20	0	1	0	.4	91.6	91.5	91.51515	92.3
2000	8	0	1	0	.4	88.6	88.8	90.04065	90.8
1000	4	0	1	0	.4	80.1	81.38138	83.28328	85.5
5000	50	0	1	0	.4	91.7	92.4	92.9078	93.2
2000	20	0	1	0	.4	91	92.19219	92.61134	92.7
1000	10	0	1	0	.4	89.4	90.5	91.0101	92.4
5000	10	1	1	0	.4	89.7	89.8	90.6	91.7
2000	4	1	1	0	.4	80.7	80.4	81.76353	86.6
1000	2	1	1	0	.4	60.3	60.9	61.82365	73.5
5000	20	1	1	0	.4	92.7	93	93.5	94.1
2000	8	1	1	0	.4	87.8	88	88.7	91.1
1000	4	1	1	0	.4	78.6	78.8	80.88088	84.1
5000	50	1	1	0	.4	94	94	95.3	95
2000	20	1	1	0	.4	92.7	92.8	93.8	94.1
1000	10	1	1	0	.4	90.7	90.6	92.1	93.2
5000	10	1	1	0	.4	89.7	89.8	90.6	91.7
2000	4	1	1	0	.4	80.7	80.4	81.76353	86.6
1000	2	1	1	0	.4	60.3	60.9	61.82365	73.5
5000	20	1	1	0	.4	92.7	93	93.5	94.1
2000	8	1	1	0	.4	87.8	88	88.7	91.1
1000	4	1	1	0	.4	78.6	78.8	80.88088	84.1
5000	50	1	1	0	.4	94	94	95.3	95
2000	20	1	1	0	.4	92.7	92.8	93.8	94.1
1000	10	1	1	0	.4	90.7	90.6	92.1	93.2
5000	10	2	2	0	.4	89.2	89.2	89.7	91.6
2000	4	2	2	0	.4	82.7	82.78278	83.58358	87.8
1000	2	2	2	0	.4	59.4	59.6	61.44578	71.5
5000	20	2	2	0	.4	92.7	92.7	93.2	93.5
2000	8	2	2	0	.4	87	87.1	88.4	90.3
1000	4	2	2	0	.4	78.5	78.7	80.6	85.4
5000	50	2	2	0	.4	93.7	93.5	95	95.3
2000	20	2	2	0	.4	91.6	91.5	92.4	93
1000	10	2	2	0	.4	86.98699	87	89.2	90.7

SE of coverage

	ssl	ssh	tsq0	tsq1	outc	beta	model1	model2	model3	model4
	5000	10	0	0	0	.4	.8874347	.911471	.8928737	.8724162
	2000	4	0	0	0	.4	1.260143	1.26086	1.250497	1.153412
	1000	2	0	0	0	.4	1.55702	1.556458	1.553968	1.466394
	5000	20	0	0	0	.4	.8529889	.8612292	.8481474	.790639
	2000	8	0	0	0	.4	.9528851	.9628813	.9334178	.8676174
	1000	4	0	0	0	.4	1.245536	1.242758	1.2282	1.13838
	5000	50	0	0	0	.4	.8068457	.8054025	.7833542	.7851369
	2000	20	0	0	0	.4	.8529889	.8611994	.8269487	.8121515
	1000	10	0	0	0	.4	.918428	.9342332	.8878273	.8430362
	5000	10	1	0	0	.4	.8724162	.8844314	.8742993	.8174105
	2000	4	1	0	0	.4	1.227905	1.232274	1.223409	1.077237
	1000	2	1	0	0	.4	1.545882	1.547597	1.540549	1.458599
	5000	20	1	0	0	.4	.8174105	.8160759	.8092851	.7739767
	2000	8	1	0	0	.4	.9854897	.9771448	.948036	.8958571
	1000	4	1	0	0	.4	1.267277	1.261248	1.227105	1.110243
	5000	50	1	0	0	.4	.6956939	.6879967	.654321	.6122009
	2000	20	1	0	0	.4	.8226239	.8091819	.7762258	.7960904
	1000	10	1	0	0	.4	.9972763	.9903735	.9528851	.8958571
	5000	10	0	1	0	.4	.9094999	.9075742	.9090852	.8014924
	2000	4	0	1	0	.4	1.26963	1.245536	1.235571	1.126051
	1000	2	0	1	0	.4	1.552946	1.543813	1.543397	1.449138
	5000	20	0	1	0	.4	.8771773	.8819014	.8856272	.8430362
	2000	8	0	1	0	.4	1.005007	.9972763	.9546348	.9139803
	1000	4	0	1	0	.4	1.262533	1.231554	1.180515	1.113441
	5000	50	0	1	0	.4	.8724162	.8379976	.8170675	.7960904
	2000	20	0	1	0	.4	.9049862	.848846	.832217	.8226239
	1000	10	0	1	0	.4	.973468	.927227	.9090852	.8379976
	5000	10	1	1	0	.4	.9612024	.957058	.9228434	.8724162
	2000	4	1	1	0	.4	1.248002	1.255325	1.22232	1.077237
	1000	2	1	1	0	.4	1.547227	1.54311	1.537833	1.395618
	5000	20	1	1	0	.4	.8226239	.8068457	.7795832	.7451107
	2000	8	1	1	0	.4	1.034969	1.027619	1.001154	.9004388
	1000	4	1	1	0	.4	1.296935	1.292501	1.244154	1.156369
	5000	50	1	1	0	.4	.7509993	.7509993	.6692608	.6892024
	2000	20	1	1	0	.4	.8226239	.8174105	.7626008	.7451107
	1000	10	1	1	0	.4	.918428	.9228434	.8529889	.7960904
	5000	10	1	1	0	.4	.9612024	.957058	.9228434	.8724162
	2000	4	1	1	0	.4	1.248002	1.255325	1.22232	1.077237
	1000	2	1	1	0	.4	1.547227	1.54311	1.537833	1.395618
	5000	20	1	1	0	.4	.8226239	.8068457	.7795832	.7451107
	2000	8	1	1	0	.4	1.034969	1.027619	1.001154	.9004388
	1000	4	1	1	0	.4	1.296935	1.292501	1.244154	1.156369
	5000	50	1	1	0	.4	.7509993	.7509993	.6692608	.6892024
	2000	20	1	1	0	.4	.8226239	.8174105	.7626008	.7451107
	1000	10	1	1	0	.4	.918428	.9228434	.8529889	.7960904
	5000	10	2	2	0	.4	.981509	.981509	.9612024	.8771773
	2000	4	2	2	0	.4	1.196123	1.194452	1.171971	1.034969
	1000	2	2	2	0	.4	1.552946	1.551722	1.542241	1.427498
	5000	20	2	2	0	.4	.8226239	.8226239	.7960904	.7795832
	2000	8	2	2	0	.4	1.063485	1.059995	1.01264	.9359006
	1000	4	2	2	0	.4	1.299134	1.294724	1.250456	1.11662
	5000	50	2	2	0	.4	.7683163	.7795832	.6892024	.6692608
	2000	20	2	2	0	.4	.8771773	.8819014	.8379976	.8068457
	1000	10	2	2	0	.4	1.06447	1.063485	.981509	.918428

Mean error

	ssl	ssh	tsq0	tsq1	outc	beta	model1	model2	model3	model4
	5000	10	0	0	0	.4	.2475001	.2466385	.2463089	.2501287
	2000	4	0	0	0	.4	.4104255	.4101493	.4091904	.410152
	1000	2	0	0	0	.4	.5911744	.5913178	.5911638	.5907899
	5000	20	0	0	0	.4	.1812661	.1813982	.1807761	.1828043
	2000	8	0	0	0	.4	.289682	.2902108	.2893774	.2894014
	1000	4	0	0	0	.4	.4243564	.4242516	.4251862	.4263124
	5000	50	0	0	0	.4	.1211108	.1216788	.1214363	.1212339
	2000	20	0	0	0	.4	.1918963	.1920662	.1924524	.1928265
	1000	10	0	0	0	.4	.2642888	.2639699	.2640595	.2650661
	5000	10	1	0	0	.4	.2517398	.2523715	.2517203	.25378
	2000	4	1	0	0	.4	.4008844	.401007	.4006355	.4031823
	1000	2	1	0	0	.4	.5593449	.5582449	.5606426	.5576118
	5000	20	1	0	0	.4	.1783822	.1785286	.1787391	.1793482
	2000	8	1	0	0	.4	.282119	.2831175	.282139	.2848025
	1000	4	1	0	0	.4	.4082713	.4084689	.4074734	.4088002
	5000	50	1	0	0	.4	.1158676	.1163313	.1158242	.1162067
	2000	20	1	0	0	.4	.1969526	.1964836	.1967598	.1987167
	1000	10	1	0	0	.4	.2762	.2765037	.2762629	.2776634
	5000	10	0	1	0	.4	.2558799	.2555295	.255059	.2557012
	2000	4	0	1	0	.4	.4032306	.4026604	.4032299	.4025763
	1000	2	0	1	0	.4	.5880683	.5839637	.5848958	.5810531
	5000	20	0	1	0	.4	.192246	.1919108	.1920282	.1921208
	2000	8	0	1	0	.4	.2976462	.2969887	.2973285	.2969464
	1000	4	0	1	0	.4	.3942061	.3942796	.3930009	.394504
	5000	50	0	1	0	.4	.1226993	.1229336	.1226	.1230973
	2000	20	0	1	0	.4	.1951312	.1943031	.1938756	.1955194
	1000	10	0	1	0	.4	.275909	.2752616	.2761141	.2758419
	5000	10	1	1	0	.4	.2607467	.260951	.2610733	.2608642
	2000	4	1	1	0	.4	.4192602	.4193449	.4203531	.4184877
	1000	2	1	1	0	.4	.5820929	.579644	.5847842	.5782692
	5000	20	1	1	0	.4	.1762268	.1761397	.1756578	.1759154

	ssl	ssh	tsq0	tsq1	outc	beta	model1	model2	model3	model4
	2000	8	1	1	0	.4	.2841426	.2841369	.28429	.285201
	1000	4	1	1	0	.4	.4215855	.4217363	.4202212	.4211554
	5000	50	1	1	0	.4	.1215073	.1216626	.1215995	.1215536
	2000	20	1	1	0	.4	.1944543	.1945051	.1945562	.1946649
	1000	10	1	1	0	.4	.2577293	.2577093	.2575584	.2569796
	5000	10	1	1	0	.4	.2607467	.260951	.2610733	.2608642
	2000	4	1	1	0	.4	.4192602	.4193449	.4203531	.4184877
	1000	2	1	1	0	.4	.5820929	.579644	.5847842	.5782692
	5000	20	1	1	0	.4	.1762268	.1761397	.1756578	.1759154
	2000	8	1	1	0	.4	.2841426	.2841369	.28429	.285201
	1000	4	1	1	0	.4	.4215855	.4217363	.4202212	.4211554
	5000	50	1	1	0	.4	.1215073	.1216626	.1215995	.1215536
	2000	20	1	1	0	.4	.1944543	.1945051	.1945562	.1946649
	1000	10	1	1	0	.4	.2577293	.2577093	.2575584	.2569796
	5000	10	2	2	0	.4	.2604881	.2604058	.2602523	.2605984
	2000	4	2	2	0	.4	.3944111	.394388	.3949793	.3937332
	1000	2	2	2	0	.4	.5643734	.5644164	.564118	.5624045
	5000	20	2	2	0	.4	.1871853	.1872084	.1871234	.1870829
	2000	8	2	2	0	.4	.2977061	.2975673	.2977646	.2980716
	1000	4	2	2	0	.4	.4095406	.4097106	.4097142	.4096263
	5000	50	2	2	0	.4	.1199405	.119949	.1198637	.1200297
	2000	20	2	2	0	.4	.1924767	.1925224	.1927734	.1921658
	1000	10	2	2	0	.4	.2806006	.2802	.2798756	.2801915

SE of mean error

	ssl	ssh	tsq0	tsq1	outc	beta	model1	model2	model3	model4
	5000	10	0	0	0	.4	.0001881	.0001914	.0001919	.0001896
	2000	4	0	0	0	.4	.0003037	.0003052	.0003069	.0003048
	1000	2	0	0	0	.4	.0004237	.0004235	.0004237	.0004278
	5000	20	0	0	0	.4	.0001408	.0001427	.0001436	.0001433
	2000	8	0	0	0	.4	.0002181	.0002206	.0002194	.0002184
	1000	4	0	0	0	.4	.000323	.0003237	.0003254	.0003236
	5000	50	0	0	0	.4	.0000929	.0000942	.0000968	.0000934
	2000	20	0	0	0	.4	.0001487	.0001496	.0001505	.0001486
	1000	10	0	0	0	.4	.000199	.0001993	.0002003	.0001996
	5000	10	1	0	0	.4	.0001886	.0001891	.0001903	.0001909
	2000	4	1	0	0	.4	.0003018	.0003039	.0003037	.0003041
	1000	2	1	0	0	.4	.0004316	.0004321	.0004327	.0004257
	5000	20	1	0	0	.4	.0001418	.0001429	.0001434	.0001426
	2000	8	1	0	0	.4	.0002112	.0002122	.0002125	.0002155
	1000	4	1	0	0	.4	.0003146	.0003145	.0003162	.0003151
	5000	50	1	0	0	.4	.0000879	.0000887	.0000885	.0000879
	2000	20	1	0	0	.4	.000148	.0001484	.0001487	.0001493
	1000	10	1	0	0	.4	.000203	.0002028	.0002024	.0002018
	5000	10	0	1	0	.4	.0001878	.0001886	.0001899	.0001878
	2000	4	0	1	0	.4	.0003173	.0003149	.0003178	.0003165
	1000	2	0	1	0	.4	.0004453	.000442	.0004428	.0004387
	5000	20	0	1	0	.4	.0001441	.0001439	.0001456	.0001443
	2000	8	0	1	0	.4	.0002254	.0002243	.0002279	.0002246
	1000	4	0	1	0	.4	.0002957	.0002934	.0002929	.0002957
	5000	50	0	1	0	.4	.0000955	.0000954	.0000963	.0000956
	2000	20	0	1	0	.4	.0001491	.0001497	.0001515	.0001493
	1000	10	0	1	0	.4	.0002036	.0002033	.0002051	.0002034
	5000	10	1	1	0	.4	.0002073	.0002074	.0002073	.0002068
	2000	4	1	1	0	.4	.0003137	.0003131	.000314	.0003151
	1000	2	1	1	0	.4	.0004404	.0004402	.0004422	.0004361
	5000	20	1	1	0	.4	.0001364	.0001365	.0001363	.0001366
	2000	8	1	1	0	.4	.0002234	.0002234	.0002234	.0002238
	1000	4	1	1	0	.4	.0003142	.0003148	.0003145	.0003159
	5000	50	1	1	0	.4	.0000922	.0000922	.0000918	.0000921
	2000	20	1	1	0	.4	.0001415	.0001415	.0001417	.0001416
	1000	10	1	1	0	.4	.0001995	.0001998	.0001996	.0001991
	5000	10	1	1	0	.4	.0002073	.0002074	.0002073	.0002068
	2000	4	1	1	0	.4	.0003137	.0003131	.000314	.0003151
	1000	2	1	1	0	.4	.0004404	.0004402	.0004422	.0004361
	5000	20	1	1	0	.4	.0001364	.0001365	.0001363	.0001366
	2000	8	1	1	0	.4	.0002234	.0002234	.0002234	.0002238
	1000	4	1	1	0	.4	.0003142	.0003148	.0003145	.0003159
	5000	50	1	1	0	.4	.0000922	.0000922	.0000918	.0000921
	2000	20	1	1	0	.4	.0001415	.0001415	.0001417	.0001416
	1000	10	1	1	0	.4	.0001995	.0001998	.0001996	.0001991
	5000	10	2	2	0	.4	.0002011	.000201	.0002013	.0002008
	2000	4	2	2	0	.4	.0002987	.0002993	.000299	.0002985
	1000	2	2	2	0	.4	.0004322	.0004337	.0004324	.0004274
	5000	20	2	2	0	.4	.0001413	.0001411	.000141	.0001415
	2000	8	2	2	0	.4	.0002252	.0002252	.0002261	.0002251
	1000	4	2	2	0	.4	.0003054	.0003053	.0003048	.0003051
	5000	50	2	2	0	.4	.0000906	.0000907	.0000909	.0000906
	2000	20	2	2	0	.4	.0001456	.0001456	.0001456	.0001452
	1000	10	2	2	0	.4	.0002148	.0002146	.000215	.0002148

Mean bias

	ssl	ssh	tsq0	tsq1	outc	beta	model1	model2	model3	model4
	5000	10	0	0	0	.4	-.0057864	-.0044076	-.0057635	-.0058766
	2000	4	0	0	0	.4	.0016908	.0028302	.0000143	.0030955
	1000	2	0	0	0	.4	-.0129901	-.0131437	-.0126387	-.014512
	5000	20	0	0	0	.4	-.0055679	-.007534	-.0069286	-.0055067

2000	8	0	0	0	.4	.0043049	.0032097	.0041381	.0050563
1000	4	0	0	0	.4	.0192566	.017999	.018877	.0192494
5000	50	0	0	0	.4	.0010895	.0007024	.0019671	.0006957
2000	20	0	0	0	.4	.0018065	.0013169	.001827	.0012073
1000	10	0	0	0	.4	-.0129062	-.0136017	-.0117589	-.0125046
5000	10	1	0	0	.4	-.0158979	-.0159673	-.0163212	-.0165766
2000	4	1	0	0	.4	.0121556	.0113969	.0126386	.012349
1000	2	1	0	0	.4	-.0101307	-.0112174	-.0101848	-.0088019
5000	20	1	0	0	.4	.0101066	.010047	.0103844	.0089127
2000	8	1	0	0	.4	.0451075	.0454261	.0465178	.0447897
1000	4	1	0	0	.4	-.0016718	-.0014279	-.0013366	-.0014017
5000	50	1	0	0	.4	-.0020602	-.002213	-.0023705	-.0020484
2000	20	1	0	0	.4	.0206899	.0201412	.0201205	.0198763
1000	10	1	0	0	.4	.0084103	.0083375	.008231	.0083833
5000	10	0	1	0	.4	-.0184825	-.0178371	-.0171163	-.0180584
2000	4	0	1	0	.4	-.0118524	-.0115875	-.0115282	-.0119111
1000	2	0	1	0	.4	.0069857	.0052055	.0094367	.0029497
5000	20	0	1	0	.4	-.0025715	-.0015912	-.0013022	-.0025363
2000	8	0	1	0	.4	.0014044	.0017373	.0006113	.0016763
1000	4	0	1	0	.4	-.0180867	-.0184811	-.0180338	-.0168992
5000	50	0	1	0	.4	-.0051168	-.00548	-.0052368	-.0051272
2000	20	0	1	0	.4	.0027252	.0023383	.0040948	.0026809
1000	10	0	1	0	.4	-.0084366	-.008561	-.009076	-.0077928
5000	10	1	1	0	.4	.0098094	.0097623	.0101698	.0101064
2000	4	1	1	0	.4	-.0256393	-.0256479	-.0252341	-.0250738
1000	2	1	1	0	.4	.0272392	.0299158	.0252448	.025347
5000	20	1	1	0	.4	.0000102	.0000196	.0000194	-.0002472
2000	8	1	1	0	.4	-.0050522	-.005144	-.0051338	-.0050643
1000	4	1	1	0	.4	-.0300817	-.0307527	-.0314858	-.0290151
5000	50	1	1	0	.4	-.0030406	-.0029851	-.0030559	-.0029504
2000	20	1	1	0	.4	-.0037539	-.0037901	-.0041923	-.0037276
1000	10	1	1	0	.4	-.0075452	-.0076699	-.0074516	-.0070626
5000	10	1	1	0	.4	.0098094	.0097623	.0101698	.0101064
2000	4	1	1	0	.4	-.0256393	-.0256479	-.0252341	-.0250738
1000	2	1	1	0	.4	.0272392	.0299158	.0252448	.025347
5000	20	1	1	0	.4	.0000102	.0000196	.0000194	-.0002472
2000	8	1	1	0	.4	-.0050522	-.005144	-.0051338	-.0050643
1000	4	1	1	0	.4	-.0300817	-.0307527	-.0314858	-.0290151
5000	50	1	1	0	.4	-.0030406	-.0029851	-.0030559	-.0029504
2000	20	1	1	0	.4	-.0037539	-.0037901	-.0041923	-.0037276
1000	10	1	1	0	.4	-.0075452	-.0076699	-.0074516	-.0070626
5000	10	2	2	0	.4	-.0158895	-.015677	-.0157381	-.016026
2000	4	2	2	0	.4	.0042096	.0048201	.0045889	.004774
1000	2	2	2	0	.4	.0248037	.02578	.0214329	.0273876
5000	20	2	2	0	.4	.0095953	.0094584	.0095151	.0098697
2000	8	2	2	0	.4	.0164911	.0164863	.0164629	.0163915
1000	4	2	2	0	.4	.0116954	.0121099	.0128391	.0116932
5000	50	2	2	0	.4	-.0016354	-.0016013	-.0016437	-.0015243
2000	20	2	2	0	.4	-.0080169	-.0079891	-.00821	-.0077583
1000	10	2	2	0	.4	.0226057	.0225029	.0226989	.021896

SE of mean bias

ssl	ssh	tsq0	tsq1	outc	beta	model1	model2	model3	model4
5000	10	0	0	0	.4	.0003111	.0003158	.0003171	.0003139
2000	4	0	0	0	.4	.0005107	.0005131	.0005147	.0005112
1000	2	0	0	0	.4	.0007275	.0007274	.0007279	.0007295
5000	20	0	0	0	.4	.0002295	.0002331	.0002345	.0002323
2000	8	0	0	0	.4	.0003627	.0003672	.0003649	.0003626
1000	4	0	0	0	.4	.0005331	.0005342	.000537	.000535
5000	50	0	0	0	.4	.0001527	.0001551	.0001588	.0001531
2000	20	0	0	0	.4	.0002429	.0002441	.0002462	.0002435
1000	10	0	0	0	.4	.0003307	.0003312	.0003328	.0003317
5000	10	1	0	0	.4	.0003142	.0003157	.0003168	.0003173
2000	4	1	0	0	.4	.0005018	.0005044	.0005037	.000505
1000	2	1	0	0	.4	.0007067	.0007065	.0007088	.0007017
5000	20	1	0	0	.4	.0002277	.0002292	.0002304	.000229
2000	8	1	0	0	.4	.0003496	.0003519	.0003512	.0003544
1000	4	1	0	0	.4	.0005156	.000516	.0005172	.0005163
5000	50	1	0	0	.4	.0001455	.0001471	.0001467	.0001457
2000	20	1	0	0	.4	.0002456	.0002459	.0002463	.0002479
1000	10	1	0	0	.4	.0003428	.0003431	.0003425	.0003433
5000	10	0	1	0	.4	.000317	.0003178	.0003197	.0003169
2000	4	0	1	0	.4	.0005131	.0005112	.0005157	.0005121
1000	2	0	1	0	.4	.0007379	.0007326	.0007352	.0007283
5000	20	0	1	0	.4	.0002403	.00024	.0002426	.0002404
2000	8	0	1	0	.4	.0003735	.0003723	.0003786	.0003725
1000	4	0	1	0	.4	.0004926	.0004916	.0004903	.0004929
5000	50	0	1	0	.4	.0001555	.0001556	.0001571	.0001558
2000	20	0	1	0	.4	.0002457	.0002455	.000248	.0002461
1000	10	0	1	0	.4	.0003429	.0003422	.0003462	.0003428
5000	10	1	1	0	.4	.000333	.0003333	.0003333	.0003329
2000	4	1	1	0	.4	.0005232	.0005229	.0005249	.0005234
1000	2	1	1	0	.4	.0007296	.0007275	.0007339	.000724
5000	20	1	1	0	.4	.0002229	.0002229	.0002224	.0002228
2000	8	1	1	0	.4	.0003615	.0003615	.0003616	.0003626
1000	4	1	1	0	.4	.0005251	.0005256	.0005244	.0005258
5000	50	1	1	0	.4	.0001525	.0001527	.0001524	.0001525
2000	20	1	1	0	.4	.0002405	.0002406	.0002408	.0002408
1000	10	1	1	0	.4	.0003259	.0003261	.0003258	.0003251
5000	10	1	1	0	.4	.000333	.0003333	.0003333	.0003329
2000	4	1	1	0	.4	.0005232	.0005229	.0005249	.0005234
1000	2	1	1	0	.4	.0007296	.0007275	.0007339	.000724
5000	20	1	1	0	.4	.0002229	.0002229	.0002224	.0002228
2000	8	1	1	0	.4	.0003615	.0003615	.0003616	.0003626

1000	4	1	1	0	.4	.0005251	.0005256	.0005244	.0005258
5000	50	1	1	0	.4	.0001525	.0001527	.0001524	.0001525
2000	20	1	1	0	.4	.0002405	.0002406	.0002408	.0002408
1000	10	1	1	0	.4	.0003259	.0003261	.0003258	.0003251
5000	10	2	2	0	.4	.0003288	.0003287	.0003287	.0003287
2000	4	2	2	0	.4	.0004949	.0004955	.0004958	.0004942
1000	2	2	2	0	.4	.0007106	.0007116	.0007125	.0007061
5000	20	2	2	0	.4	.0002344	.0002343	.0002342	.0002344
2000	8	2	2	0	.4	.000373	.0003729	.0003736	.0003732
1000	4	2	2	0	.4	.0005109	.000511	.0005107	.0005108
5000	50	2	2	0	.4	.0001504	.0001504	.0001504	.0001504
2000	20	2	2	0	.4	.0002413	.0002413	.0002415	.0002408
1000	10	2	2	0	.4	.000353	.0003523	.0003523	.0003525

Power

ssl	ssh	tsq0	tsq1	outc	beta	model1	model2	model3	model4
5000	10	0	0	0	.4	13.51351	13.64562	13.52459	11.8
2000	4	0	0	0	.4	19	18.89447	18.16347	15.5
1000	2	0	0	0	.4	27.8	27.9	26.62663	21.2
5000	20	0	0	0	.4	15.1	14.73577	14.15385	13.9
2000	8	0	0	0	.4	13.1	13.04348	12.78953	10.5
1000	4	0	0	0	.4	17.9	17.13427	16.38191	13.7
5000	50	0	0	0	.4	28.5	28.23887	26.94301	25.9
2000	20	0	0	0	.4	15.9	15.96386	14.87854	13.8
1000	10	0	0	0	.4	12.8	12.73821	11.58107	11.4
5000	10	1	0	0	.4	12.8	12.43731	11.89516	10.2
2000	4	1	0	0	.4	17.8	17.97189	17.65296	13.9
1000	2	1	0	0	.4	27.7	27.32733	27.32733	21.2
5000	20	1	0	0	.4	16.8	16.78392	16.66667	15.7
2000	8	1	0	0	.4	15.3	15.06024	14.55823	12.7
1000	4	1	0	0	.4	17.4	17.31732	16.56627	13.2
5000	50	1	0	0	.4	27.1	26.71371	26.23613	25.6
2000	20	1	0	0	.4	19.4	18.65597	18.25476	17
1000	10	1	0	0	.4	15.8	15.71572	15	12.7
5000	10	0	1	0	.4	13.4	12.73821	12.62626	11.4
2000	4	0	1	0	.4	17.1	16.1	15.60926	12.3
1000	2	0	1	0	.4	27.5	26.5	26.68004	19.5
5000	20	0	1	0	.4	17.4	17.2	16.86869	15.8
2000	8	0	1	0	.4	13.9	13.2	13.00813	10.2
1000	4	0	1	0	.4	17.2	17.11712	16.71672	13
5000	50	0	1	0	.4	27.6	25.3	24.11348	24.4
2000	20	0	1	0	.4	17.4	16.71672	15.68826	14.5
1000	10	0	1	0	.4	14.3	13.2	11.51515	10.7
5000	10	1	1	0	.4	13.8	13.7	13.1	12
2000	4	1	1	0	.4	17.4	17.2	16.73347	12.9
1000	2	1	1	0	.4	29.3	29.4	28.85772	20.5
5000	20	1	1	0	.4	16	15.8	15.2	14.6
2000	8	1	1	0	.4	14.8	14.6	13.4	12.1
1000	4	1	1	0	.4	17.2	17.3	15.71572	12.7
5000	50	1	1	0	.4	27.5	27.8	24.3	24
2000	20	1	1	0	.4	18	17.8	15.2	14.2
1000	10	1	1	0	.4	12.6	12.7	11.1	9.7
5000	10	1	1	0	.4	13.8	13.7	13.1	12
2000	4	1	1	0	.4	17.4	17.2	16.73347	12.9
1000	2	1	1	0	.4	29.3	29.4	28.85772	20.5
5000	20	1	1	0	.4	16	15.8	15.2	14.6
2000	8	1	1	0	.4	14.8	14.6	13.4	12.1
1000	4	1	1	0	.4	17.2	17.3	15.71572	12.7
5000	50	1	1	0	.4	27.5	27.8	24.3	24
2000	20	1	1	0	.4	18	17.8	15.2	14.2
1000	10	1	1	0	.4	12.6	12.7	11.1	9.7
5000	10	2	2	0	.4	12.3	12.4	11.8	10.5
2000	4	2	2	0	.4	17.3	17.01702	15.71572	12.5
1000	2	2	2	0	.4	29.9	30	28.01205	20.8
5000	20	2	2	0	.4	19	18.9	18	16.9
2000	8	2	2	0	.4	14.6	14.3	13.2	10.8
1000	4	2	2	0	.4	17.5	17.8	17	12.8
5000	50	2	2	0	.4	28.5	28.4	25.3	24.4
2000	20	2	2	0	.4	16.5	16.4	13.8	12.7
1000	10	2	2	0	.4	17.41742	17.3	15.1	13.5

SE of power

ssl	ssh	tsq0	tsq1	outc	beta	model1	model2	model3	model4
5000	10	0	0	0	.4	1.081622	1.095426	1.094671	1.020176
2000	4	0	0	0	.4	1.240564	1.241027	1.224718	1.144443
1000	2	0	0	0	.4	1.416743	1.418305	1.398442	1.292501
5000	20	0	0	0	.4	1.13225	1.129984	1.116337	1.093979
2000	8	0	0	0	.4	1.066954	1.070902	1.059832	.969407
1000	4	0	0	0	.4	1.212266	1.192765	1.173332	1.087341
5000	50	0	0	0	.4	1.427498	1.432154	1.428204	1.385348
2000	20	0	0	0	.4	1.156369	1.160573	1.132195	1.09067
1000	10	0	0	0	.4	1.056485	1.05589	1.015482	1.005007
5000	10	1	0	0	.4	1.056485	1.045142	1.027849	.957058
2000	4	1	0	0	.4	1.209612	1.216602	1.207495	1.093979
1000	2	1	0	0	.4	1.415171	1.409942	1.409942	1.292501
5000	20	1	0	0	.4	1.182269	1.184783	1.184448	1.150439
2000	8	1	0	0	.4	1.13838	1.133292	1.117532	1.052953

1000	4	1	0	0	.4	1.198849	1.197195	1.178022	1.070402
5000	50	1	0	0	.4	1.405557	1.404826	1.397446	1.380087
2000	20	1	0	0	.4	1.250456	1.233741	1.223409	1.187855
1000	10	1	0	0	.4	1.153412	1.151483	1.129159	1.052953
5000	10	0	1	0	.4	1.077237	1.05589	1.055626	1.005007
2000	4	0	1	0	.4	1.190626	1.162235	1.151765	1.03861
1000	2	0	1	0	.4	1.412002	1.395618	1.400738	1.252897
5000	20	0	1	0	.4	1.198849	1.193382	1.19016	1.153412
2000	8	0	1	0	.4	1.093979	1.070402	1.072381	.957058
1000	4	0	1	0	.4	1.193382	1.191695	1.180515	1.063485
5000	50	0	1	0	.4	1.413591	1.37474	1.361613	1.358175
2000	20	0	1	0	.4	1.198849	1.180515	1.157052	1.113441
1000	10	0	1	0	.4	1.107028	1.070402	1.014499	.9775019
5000	10	1	1	0	.4	1.09067	1.087341	1.066954	1.027619
2000	4	1	1	0	.4	1.198849	1.193382	1.181579	1.059995
1000	2	1	1	0	.4	1.439274	1.440708	1.434266	1.276617
5000	20	1	1	0	.4	1.15931	1.153412	1.135324	1.11662
2000	8	1	1	0	.4	1.122925	1.11662	1.077237	1.031305
1000	4	1	1	0	.4	1.193382	1.196123	1.151483	1.052953
5000	50	1	1	0	.4	1.412002	1.416743	1.356285	1.350555
2000	20	1	1	0	.4	1.214907	1.209612	1.135324	1.103793
1000	10	1	1	0	.4	1.0494	1.052953	.993373	.9359006
5000	10	1	1	0	.4	1.09067	1.087341	1.066954	1.027619
2000	4	1	1	0	.4	1.198849	1.193382	1.181579	1.059995
1000	2	1	1	0	.4	1.439274	1.440708	1.434266	1.276617
5000	20	1	1	0	.4	1.15931	1.153412	1.135324	1.11662
2000	8	1	1	0	.4	1.122925	1.11662	1.077237	1.031305
1000	4	1	1	0	.4	1.193382	1.196123	1.151483	1.052953
5000	50	1	1	0	.4	1.412002	1.416743	1.356285	1.350555
2000	20	1	1	0	.4	1.214907	1.209612	1.135324	1.103793
1000	10	1	1	0	.4	1.0494	1.052953	.993373	.9359006
5000	10	2	2	0	.4	1.03861	1.042228	1.020176	.969407
2000	4	2	2	0	.4	1.196123	1.188922	1.151483	1.045825
1000	2	2	2	0	.4	1.447753	1.449138	1.422894	1.283495
5000	20	2	2	0	.4	1.240564	1.238059	1.214907	1.18507
2000	8	2	2	0	.4	1.11662	1.107028	1.070402	.981509
1000	4	2	2	0	.4	1.201561	1.209612	1.187855	1.056485
5000	50	2	2	0	.4	1.427498	1.425987	1.37474	1.358175
2000	20	2	2	0	.4	1.173776	1.170914	1.09067	1.052953
1000	10	2	2	0	.4	1.199923	1.196123	1.13225	1.080625

Model 1: Fixed study-specific intercepts; random treatment effect; Fixed study-specific effects for baseline; random effect for interaction
Model 2: Random study intercept; random treatment effect; Fixed study-specific effects for baseline; random effect for interaction
Model 3: Random study intercept; random treatment effect; random effect for baseline; random effect for interaction
Model 4: two-stage IPD with ipdmetan

ss1: size for lower level unit (patients)
ssh: size for higher level unit (studies)
tsq0: between study variance for the intercept
tsq1: between study variance for the exposure
outc: outcome type (0=continuous)
beta: true effect size
